# Olefination with Sulfonyl Halides and Esters: Synthesis of Unsaturated Sulfonyl Fluorides

**DOI:** 10.1021/acs.orglett.2c01604

**Published:** 2022-06-02

**Authors:** Michał Tryniszewski, Dariusz Basiak, Michał Barbasiewicz

**Affiliations:** † Faculty of Chemistry, University of Warsaw, Pasteura 1, 02-093 Warsaw, Poland

## Abstract

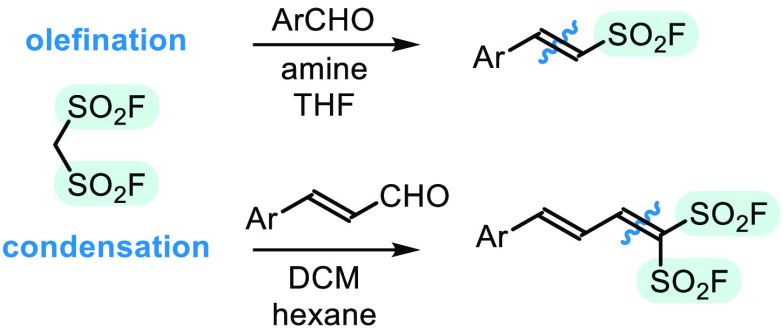

Methanedisulfonyl fluoride, CH_2_(SO_2_F)_2_, transforms aromatic aldehydes into β-arylethenesulfonyl fluorides, useful substrates for the SuFEx “click”-type transformations. The reaction mimics mechanism of the Horner–Wadsworth–Emmons olefination, which runs via addition of the carbanion, followed by cyclization–fragmentation of the four-membered ring intermediate. In the absence of base, electron-rich aldehydes follow an alternative pathway of the Knoevenagel condensation to provide unsaturated 1,1-disulfonyl fluorides. We demonstrate also trapping of elusive ethene-1,1-disulfonyl fluoride, CH_2_=C(SO_2_F)_2_, with 4-(dimethylamino)pyridine (DMAP) that forms zwitterionic adduct, characterized with X-ray studies.

Sulfur Fluoride Exchange reaction (SuFEx) is a valuable tool for “click”-type formation of the S–O, S–N, and S–C bonds, applied in organic synthesis, drug-discovery, molecular biology, and material science.^1,2^ Unique substrates for the transformations are sulfonyl fluorides, which display unprecedented combination of stability and reactivity.^3^ The contradictory features of the reagents inspired the term ‘*sleeping beauties*’, which, apparently intact, awake on demand to react in the most desired way.^2^ Among them β-arylethenesulfonyl fluorides, ArCH=CHSO_2_F, are recognized as selectively addressable bis-electrophiles, able to react as Michael acceptors, or via sulfur substitution, depending on the reaction conditions.^4^ Historical methods of their preparation consist of chlorosulfonation–fluorination of styrenes,^5^ and Horner–Wadsworth–Emmons olefination of arylaldehydes, followed by scission of intermediate sulfonate, chlorination, and halogen exchange.^6^ More efficient, one-step procedures developed recently by Qin, Sharpless, Arvidsson, and others, utilize Heck–Matsuda couplings of ethenesulfonyl fluoride, CH_2_=CHSO_2_F (ESF),^7^ with arenediazonium salts,^4^ aryl boronates,^8^ and iodoarenes.^9^ In the follow-up studies, similar approach was demonstrated also for the C–H alkenylation of arenes, in processes directed by functional groups,^10^ or governed by π-electron distribution of the aromatic substrates.^11^ Only recently, mechanistically distinct radical fluorosulfonation of alkenes with SO_2_ClF under blue LED irradiation was developed by Liao (Scheme 1, top).^12^

**Scheme 1:**
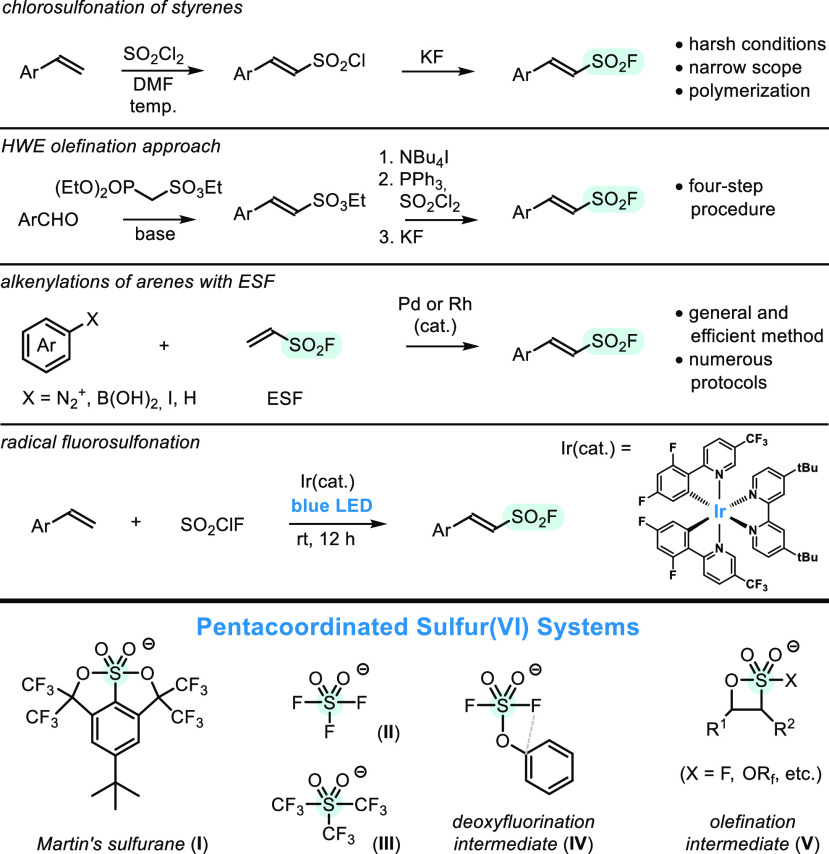
Literature Methods of Synthesis of β-Arylethenesulfonyl Fluorides (Top), and Selected Pentacoordinated Sulfur(VI) Systems (Bottom)

Our research group explores organic transformations of sulfonyl- and carbonyl-containing substrates, demonstrated on functionalization of nitroarenes,^13^ synthesis and transformations of sulfonyl fluorides,^14^ and carbonyl olefination reactions.^15^ Recently, we developed *olefination with sulfonyl halides and esters*, which mimics the Horner-Wadsworth-Emmons reaction of alkanephosphonates.^16^ Accordingly, sulfonyl-stabilized carbanions add to the carbonyl groups of aldehydes or ketones, and so-formed aldol-type adducts cyclize to four-membered ring intermediates, which fragment to alkenes. Although the reaction was reported for the first time in 1990 by Hawkins,^17^ and in 1991 by Kagabu,^18^ the preliminary results remained practically unknown in the chemical literature. Inspiration of the Hawkins’ pioneered studies was a report on tricyclic sulfurane **I**, synthesized by Martin (Scheme 1, bottom).^19^ The pentacoordinated, trigonal bipyramidal sulfur atom present in the structure was stabilized by chelation with electronegative hexafluoroalkoxide ligands, whereas more donating analogues underwent rapid degenerate rearrangement between tetracoordinated sulfonates.^20^ Later, unchelated SO_2_X_3_(−) anions bearing strongly electron-withdrawing ligands (X = F and CF_3_) were reported as moderately stable species (**II**^21^ and **III**,^22^ respectively), and postulated as intermediates in deoxyfluorination of phenols via aryl fluorosulfonates (**IV**).^23^ Importantly, similar structural motif can be recognized in transient four-membered ring intermediates of the sulfonyl-based olefination (**V**). Indeed, observations of Hawkins^17^ and us^15a,15b^ fully confirmed that only sulfonates of fluorinated alcohols and phenols are able to give alkenes, whereas nonactivated neopentyl esters fail to undergo second step of the reaction (only initial aldol-type adducts are formed). In turn more electrophilic sulfonyl fluorides were reported as precursors for the synthesis of stilbenes and cinnamyl-type products;^18^ e.g., Kagabu demonstrated that ethyl fluorosulfonylacetate, FSO_2_CH_2_CO_2_Et, reacts with benzaldehyde in the presence of NEt_3_ to afford ethyl cinnamate, isolated in 78% yield.^18b^ Based on this precedent we reckoned that methanedisulfonyl fluoride, CH_2_(SO_2_F)_2_ (MDSF, **1**), may act as a symmetrical precursor, in which one of the SO_2_F groups reacts in the olefination process,^24^ and the latter remains installed on the newly formed C=C bond. In our report we present direct, one-step transformation of arylaldehydes into β-arylethenesulfonyl fluorides, and spontaneous Knoevenagel-type condensation of **1** with electron-rich aldehydes.

Our studies began from the preparation of methanedisulfonyl fluoride (**1**) in two steps, starting from inexpensive acetic acid, POCl_3_, and HSO_3_Cl,^25^ and followed by double halogen exchange (SO_2_Cl → SO_2_F) with KHF_2_ in dry acetonitrile.^26^ After short optimization the synthesis was carried out on a 1 mol scale, and **1** was isolated by distillation in 67% yield over two steps.^27^ Attempts at model reaction of **1** with 2-naphthaldehyde started from conditions described by Kagabu.^18b^ We observed that the process runs rather slowly, thus requires prolonged heating in boiling THF, and displays strong effect of structure of the amine base on the reaction course. After testing 11 low molecular weight tertiary amines, we selected *N*-methylpyrrolidine, as a reagent of choice, able to yield the expected 2-(2-naphthyl)ethenesulfonyl fluoride (**2u**, 70%), as an exclusive *E*-isomer.^27^ Results of reactions with other aldehydes are presented in Scheme 2.

**Scheme 2:**
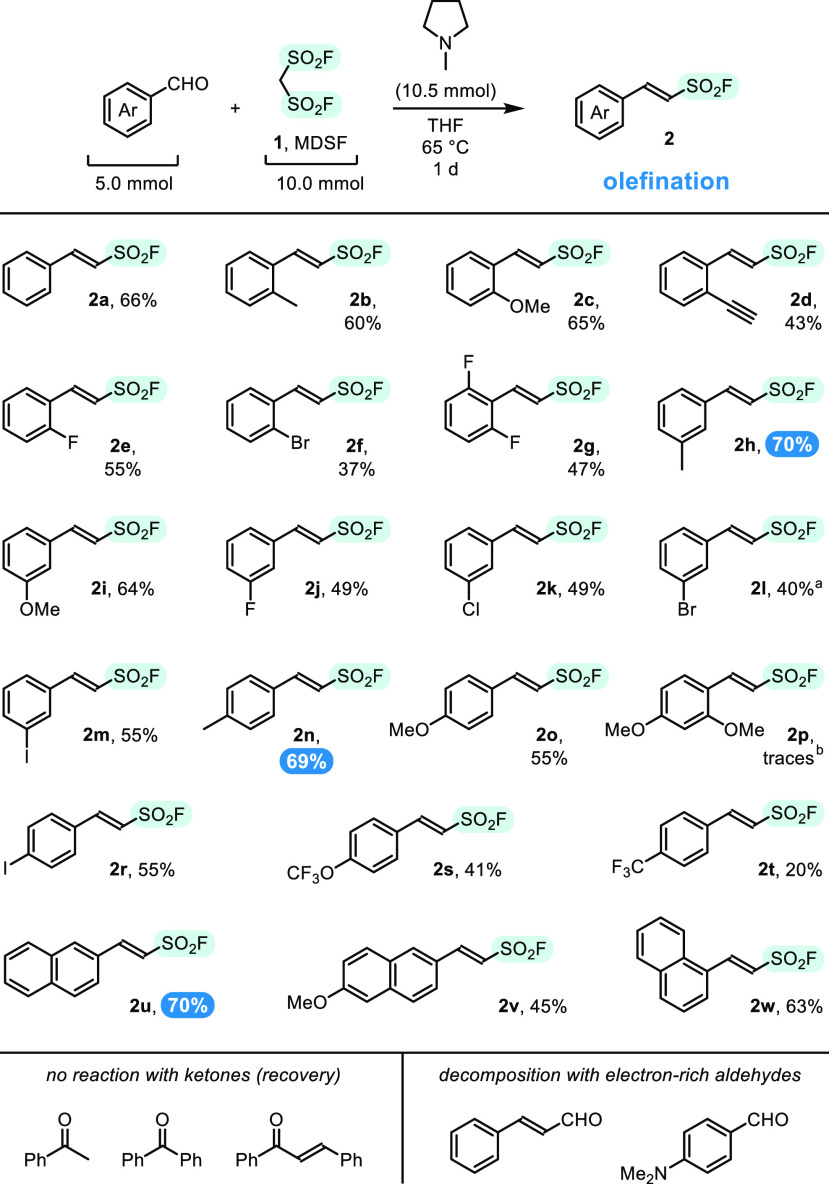
Synthesis of β-Arylethenesulfonyl Fluorides in the Reaction of **1** with Arylaldehydes The reaction carried out with 1,2,2,6,6-pentamethylpiperidine, as a base, gave 17% of **2l**. The reaction carried out at rt gave 25% of **2p**, accompanied by byproduct.

Surprisingly, the scope and limitation studies revealed a rather disappointing observation that the highest yields of **2** are obtained for arylaldehydes with neutral substitution pattern, whereas presence of donors and acceptors decreases product yields. Origins of the effect were partially revealed, when pure samples of isolated sulfonyl fluorides, bearing naphthyl (**2u**) and 4-trifluoromethylphenyl group (**2t**), were subjected to standard olefination conditions. In the first case most of the product remained intact and was recovered in 95%, whereas *more electrophilic* CF_3_-substituted sulfonyl fluoride partially decomposed and was recovered in only 42%. Accordingly, prolonged heating of the products with amine may cause slow degradation, likely due to Michael-type addition and polymerization events.

Additionally, a useful hint regarding cause of lower yields obtained for *electron-rich* substrates was given from reaction of 4-methylsalicylaldehyde, which unexpectedly led to sulfocoumarin, substituted with the SO_2_F group (**3a**, 29%). The reaction mimicked process described recently by Yang for ethyl chlorosulfonyl acetate, ClSO_2_CH_2_CO_2_Et, in which analogous sulfocoumarin-3-carboxylates were formed in good yields.^28^ On the basis of the reported procedure and our own experimentation,^27^ we chose pyridine in 1,2-dichloroethane (DCE) at 65 °C as optimal conditions and performed a few reactions with salicylaldehydes (Scheme 3).

**Scheme 3:**
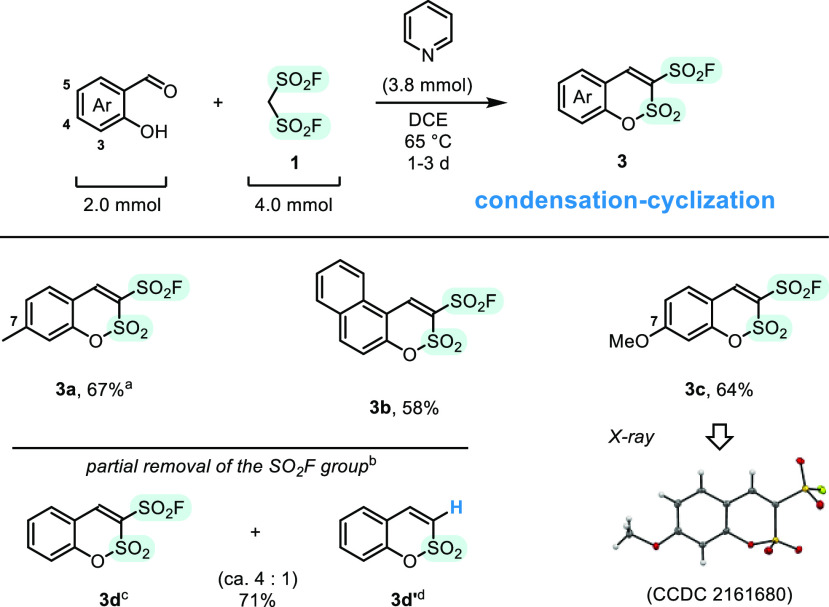
Synthesis of Sulfocoumarins in the Reaction of **1** with Salicylaldehydes The reaction carried out under olefination conditions (*cf*. Scheme 2) gave 29% of **3a**. The reaction with salicylaldehyde was carried out for 7 d. Similar de(fluorosulfonylation) process was observed for 3-methyl- and 5-methylsalicylaldehydes. Analytical sample of **3d** was isolated in 33% of yield. Analytical sample of **3d′** was isolated in 6% of yield.

7-Methyl- (**3a**), naphthyl- (**3b**), and 7-methoxysulfocoumarin (**3c**) were isolated in 58–67% yield, and structure of the latter was confirmed with X-ray studies. Importantly, 6- and 7-alkoxysubstituted sulfocoumarins are potent and selective inhibitors of human carbonic anhydrases (hCA);^29^ thus, their methods of preparation are in great demand. Unfortunately, under these conditions, parent salicylaldehyde and its 3- and 5-methyl derivatives formed hardly separable mixtures of products, resulted from partial removal of the fluorosulfonyl group. Interestingly, similar removal of the ester function was demonstrated on sulfocoumarin-3-carboxylates under Happer’s decarboxylation conditions (LiI, DMF, reflux).^28^ Mechanism of formation of the sulfocoumarins, proposed by Yang, consisted of initial generation of sulfene, which adds to the phenoxide, and so-formed aryl sulfonate cyclizes by condensation with the carbonyl group.^28^ However, we supposed that for MDSF (**1**) the order of events is plausibly reversed: namely, carbonyl group of the aldehyde condenses to the Knoevenagel-type adduct, and then one of the fluorosulfonyl groups is forced toward substitution with proximal phenoxide anion. The idea has been supported by isolation of condensation product with donor-substituted 4-(dimethylamino)benzaldehyde, when the olefination was attempted at rt (**4n**, 39%).^27^ Interestingly, the same reaction was already reported in 1979 by Yagupolskii et al., who heated the substrates in acetic anhydride at 50 °C for 3 h (yield 84%).^30^ Based on this, we reasoned that under olefination conditions more electron-rich aldehydes form styrenes bearing *two* fluorosulfonyl groups, which likely decompose in the presence of amine at higher temperature. Similar obstacles were considered by Qin et al. in studies of condensation of halomethanesulfonyl fluorides, HalCH_2_SO_2_F, with cinnamaldehydes, promoted by pyrrolidine.^31^ Yet further support, based on literature data, arose from report on condensation of *cinnamaldehydes* with close analog of **1**: bis(trifluoromethanesulfonyl)methane, CH_2_(SO_2_CF_3_)_2_. Yanai et al. reported that the condensation runs spontaneously in DCE at rt for 3–10 h, giving crystalline, yellowish-colored products, stable on air,^32^ and the process is promoted by the substrate, which is strong Brønsted acid. Surprisingly, our literature search revealed that both CH_2_(SO_2_CF_3_)_2_ and **1** display essentially the same acidity in DMSO (p*K*_a_ = 2.4),^33^ being stronger than, e.g., trifluoroacetic acid (p*K*_a_ = 3.45).^32a^ Based on this we attempted synthesis of the Knoevenagel-type adducts with electron-rich aldehydes. To facilitate separation of the expected products we applied conditions of Yanai, but concentrated DCM solutions of substrates were additionally layered with hexane and left at rt overnight. After slow diffusional mixing of the organic phases, we observed formation of yellowish, millimeter-size block crystals of cinnamaldehyde derivative **4a**, isolated in two crops in 85% yield, and characterized with X-ray studies. Analogously, set of products **4b**–**o** was obtained in excellent yields, as shown at Scheme 4.^27^

**Scheme 4:**
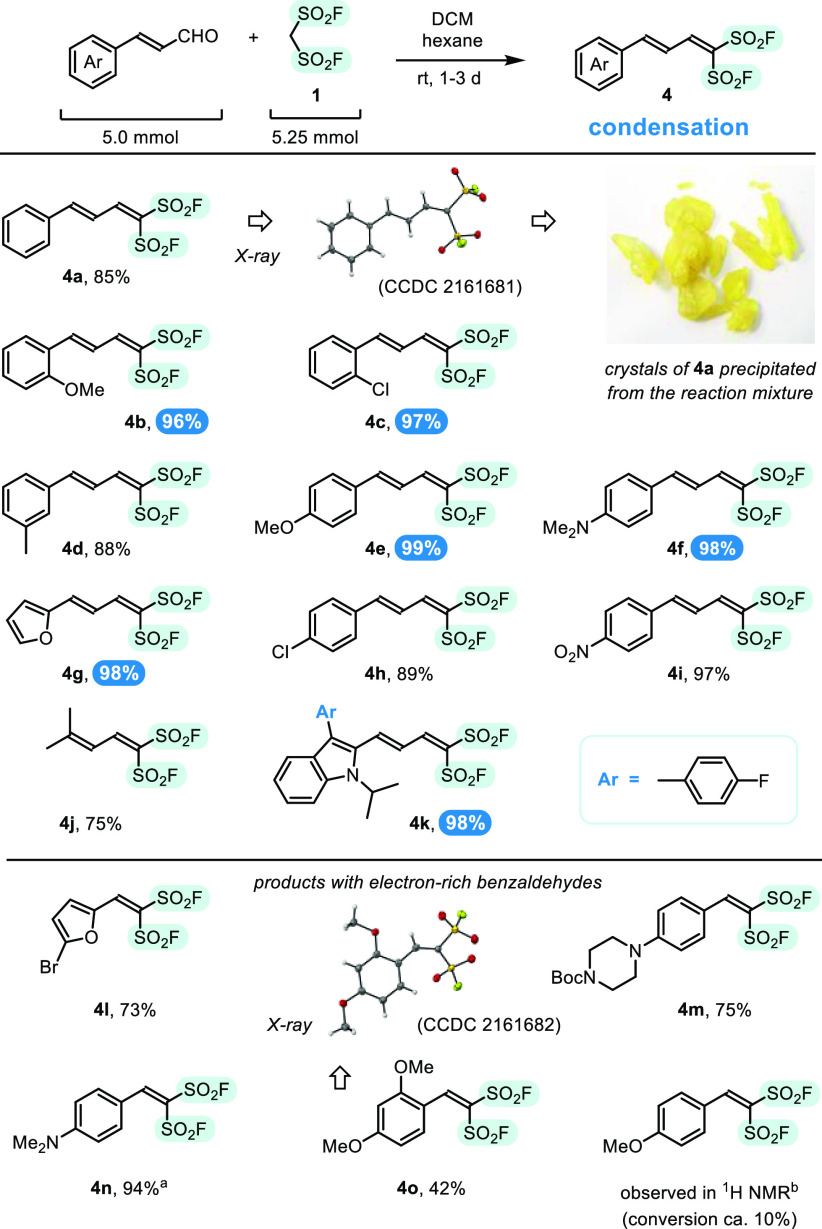
Synthesis of Unsaturated 1,1-Disulfonyl Fluorides via Knoevenagel Condensation of **1** with Electron-Rich Aldehydes Product **4n** was reported in the literature.^30^ No precipitate was formed.

Interestingly, when donor properties of benzaldehyde substituents decreased in the series from 4-Me_2_N, to 2,4-diMeO, and to 4-MeO, yields of the condensation products also decreased from 94% for **4n**, to 42% for **4o**, and to only 10% of conversion, observed by ^1^H NMR (Scheme 4, bottom).^27^ The trend was consistent with observations by Yanai, who isolated benzaldehyde adduct with CH_2_(SO_2_CF_3_)_2_ in only 7% yield, and earlier report by Zhu,^34^ who forced dehydration reaction with acetic anhydride, but after isolation observed fast decomposition to substrates. The facts taken together lead to the rather unusual conclusion that formation of the condensation products is thermodynamically controlled with electronic (push–pull) stabilization between π-electron system and bissulfonyl center, and thus counterintuitively more electrophilic aldehydes give lower yields of **4**, than electron-rich ones.

Following our inspiration with reactivity of the CH_2_(SO_2_CF_3_)_2_, we considered generation of condensation product of **1** with formaldehyde. As ethenesulfonyl fluoride (ESF)^7^ is considered to be *the most prefect Michael acceptor ever found*,^35^ one would expect that analogue bearing two fluorosulfonyl groups may supersede its electrophilic properties and become another useful hub for the SuFEx processes.^2,3b^ On the basis of literature data we heated **1** with paraformaldehyde and substituted pyridines in DCE.^32b^ Although 2-fluoropyridine, pyridine, and 2-fluoro-4-(dimethylamino)pyridine gave complex mixtures of products, reaction with nucleophilic 4-(dimethylamino)pyridine (DMAP) led to the formation of white precipitate **5**, isolated in 81% yield (Scheme 5, top).^27^

**Scheme 5:**
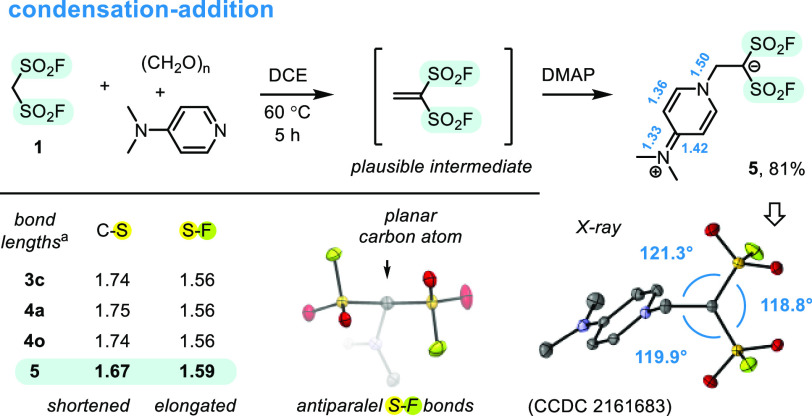
Follow-up Studies: Generation of Transient Ethene-1,1-disulfonyl Fluoride, and X-ray Structure of Its Zwitterionic Adduct with DMAP, **5** The bond lengths were taken from X-ray structures of compounds **3c**, **4a**, **4o**, and **5**, averaged for each molecule, and given in Å. Hydrogen atoms were omitted for clarity.

Structure of **5**, established with X-ray studies, revealed a unique zwitterionic form, which paralleled structures of related bis(trifluoromethanesulfonyl) derivatives, reported in the literature.^32b,36^ Stabilization of negative charge with two SO_2_F groups caused planarization of the carbanionic center and resulted in alternations of the C–S (−0.07 Å) and S–F (+0.03 Å) bond lengths, as compared with neutral structures of **3c**, **4a**, and **4o** (Scheme 5, bottom). To the best of our knowledge, **5** represents one of the very few examples of stable carbanions of sulfonyl fluorides,^37^ which usually eliminate to sulfenes.

In conclusion, we presented one-step transformation of arylaldehydes into β-arylethenesulfonyl fluorides, using easily accessible methanedisulfonyl fluoride (**1**). With electron-rich aldehydes (e.g., cinnamaldehydes) the precursor spontaneously condenses, to afford Knoevenagel-type products, isolated in excellent yields. Transient ethene-1,1-bissulfonyl fluoride, formed in reaction of **1** with paraformaldehyde in the presence of DMAP, gives stable zwitterionic adduct, with planar carbanionic center, stabilized with two SO_2_F groups. The presented results expand armory of synthetic methods for preparation of valuable SuFEx reagents, and understanding of their activation and reactivity.

## Supporting Information Available

Experimental procedures, characterization data, and NMR spectra reproductions of the synthesized compounds (PDF)

## Accession Codes

CCDC 2161680–2161683 contain the supplementary crystallographic data for this paper. These data can be obtained free of charge via www.ccdc.cam.ac.uk/data_request/cif, or by emailing data_request@ccdc.cam.ac.uk, or by contacting The Cambridge Crystallographic Data Centre, 12 Union Road, Cambridge CB2 1EZ, UK; fax: +44 1223 336033.
