# Supplementary material for: Olefination with Sulfonyl Halides and Esters: Synthesis of Unsaturated Sulfonyl Fluorides
Source: Org Lett. 2022 Jun 2;24(23):4270–4. doi: 10.1021/acs.orglett.2c01604 (PMC9490844; doi:10.1021/acs.orglett.2c01604)
Supplement: Supplementary file 1 — ol2c01604_si_001.pdf [file ol2c01604_si_001.pdf]

Olefination with Sulfonyl Halides and Esters: Synthesis of Unsaturated Sulfonyl Fluorides

Michał Tryniszewski,[1] Dariusz Basiak[1] and Michał Barbasiewicz[1,*]

[1]Faculty of Chemistry, University of Warsaw, Pasteura 1, 02-093 Warsaw, Poland

*barbasiewicz@chem.uw.edu.pl, www.aromaticity.pl

Supporting Information

Table of content

1. General Informations ... 3
2. Synthesis of Methanedisulfonyl Fluoride (**1**, MDSF) ... 5
   2.1. Optimization ... 5
   2.2. Preparation of Methanedisulfonyl Chloride (Optimal Conditions) ... 5
   2.3. Preparation of Methanedisulfonyl Fluoride (Optimal Conditions) ... 6
3. Synthesis of β-Arylethenesulfonyl Fluorides, **2** ... 7
   3.1. Optimization ... 7
   3.2. Other Processes Occurring During Reaction ... 8
   3.3. Preparation of β-Arylethenesulfonyl Fluorides (General Procedure) ... 9
   3.4. Stability Studies of 2t and 2u under the Reaction Conditions ... 16
4. Synthesis of Sulfocoumarines, **3** ... 17
   4.1. Optimization ... 17
   4.2. Preparation of Sulfocoumarines (Optimal Conditions) ... 17
   4.3. Reactions with Other Salicylaldehydes ... 19
5. Synthesis of Unsaturated 1,1-Disulfonyl Fluorides, **4** ... 20
   5.1. General procedure ... 20
   5.2. Condensations of Electron-rich Benzaldehydes Under Various Conditions ... 25
   5.3. ^{1}H NMR Spectrum of *p*-Anisaldehyde Condensation ... 26
   5.4. NMR Assignments of Diene 4j ... 27
6. Reactions of 1 with Paraformaldehyde and Pyridines ... 30
7. Synthesis of 2a, 2n, and 2u by Chlorosulfonation of Styrenes ... 33
8. Single-crystal X-Ray Diffraction Analysis ... 34
   8.1. Experimental ... 34
   8.2. Results ... 35
   8.3. Plots of X-ray structures of 3c, 4a, 4o and 5 ... 36
9. ^{1}H, ^{13}C and ^{19}F NMR Spectra of Characterized Compounds ... 38

1. General Informations

^{1}H, ^{19}F, and ^{13}C **NMR** spectra were recorded on Agilent 400 MHz NMR spectrometer. Chemical shifts (δ) are given in parts per million (ppm) with solvent resonance as the internal standard (for $CDCl_3$: 7.24 and 77.0 ppm, for CD_3CN: 1.96 and 118.3 ppm, for acetone-d_3: 2.05 and 206.3 ppm) or with $CFCl_3$ in $CDCl_3$ (0.0 ppm for ^{19}F NMR). Spin multiplicity was abbreviated as follows: s, singlet; d, doublet; t, triplet; q, quartet; hept, heptet; and m, multiplet.

Melting points were uncorrected.

Analytical gas-liquid chromatography (**GLC**) was performed on a PerkinElmer Clarus 580 chromatograph equipped with a flame ionization detector, and a GL Sciences InertCap 5MS/Sil column with helium as a carrier gas (column 0.25 mm × 30 m, carrier flow 1.5 mL/min, method parameters 50 °C, +10 °C/min to 300 °C, then 15 min at 300 °C).

Electron ionization (**EI**) high-resolution mass spectra were recorded using the narrow-range high-voltage scan technique with low-boiling perfluorokerosene (PFK) as internal standard. Measurements were carried out with a magnetic sector mass spectrometer *AutoSpec Premier* (*Waters, USA)* equipped with an electron impact (EI) ion source and the EBE double focusing geometry mass analyzer. Samples were introduced by using a heated direct insertion probe. The instrument was controlled and recorded data were processed using *MassLynx 4.1* software package (*Waters, USA*).

Mass spectrometry analysis was performed using Synapt G2-S mass spectrometer (Waters) equipped with the electrospray (**ESI**) ion source and quadrupole-Time-of-flight (qTOF) mass analyzer. Methanol (Honeywell, LC-MS Chromasolv™, purity ≥ 99.9%) was used as a solvent and mobile phase with the flow rate 100 µl/min. Sample was dissolved and injected directly into the ESI source. Injection volume was 1 µl. The measurement was performed in the positive ion mode with the resolving power of TOF analyzer 20000 FWHM. The lock-spray spectrum of Leucine-enkephalin was generated by the lock-spray source and the correction was performed for the recorded spectrum in the mass range of *m/z* = 50-1200. The exact mass measurement was performed within 3 mDa mass error. Nitrogen was used as desolvation and cone gas, and their flow values were set to 600 L/h and 100 L/h respectively. Desolvation gas temperature was set to 350°C. Nebulizer gas pressure was set to 5.0 bar. Capillary voltage was set to 3.0 kV, and sampling cone voltage and source offset were set to 20 V. The instrument was controlled and data were processed using the MassLynx V4.1 software package (Waters).

Thin layer chromatography (**TLC**) was performed on Supelco silica gel on TLC Al foils with fluorescent indicator 254 nm and was visualized under UV lamp. Column chromatography was performed on silica gel (high-purity grade, pore size 60 Å, 230-400 mesh particle size, 40-63 m, 60737).

Commercial suppliers of the reagents: Benzaldehyde, *o*-tolualdehyde, 2-ethynyl-benzaldehyde, 2-fluorobenzaldehyde, 2-bromobenzaldehyde, 2,6-difluorobenzaldehyde, *p*-tolualdehyde, *p*-anisaldehyde, 2,4-dimethoxybenzaldehyde, 4-(dimethylamino)benzalde-hyde, 1-naphthaldehyde, 2-hydroxy-4-methylbenzaldehyde, 2-hydroxy-1-naphthaldehyde, *trans*-cinnamaldehyde, acetophenone, benzophenone, *trans*-chalcone, 3-methyl-2-butenal, *trans*-4-nitrocinnamaldehyde, 1,5,7-triazabicyclo[4.4.0]dec-5-ene, 1,4-diazabicyclo[2.2.2]-octane, *N,N,N′,N′*-tetramethylethylenediamine, pyridine, 4-(dimethylamino)pyridine, phosphorus(V) oxychloride, tetrabutylammonium chloride, acetonitrile (anhydrous), THF (anhydrous), DCM (anhydrous), DCE (anhydrous), 1,4-dioxane (anhydrous) and hexane (anhydrous) were purchased from Sigma-Aldrich. *m*-Tolualdehyde, *m*-anisaldehyde, 3-bromobenzaldehyde, (2*E*)-3-(furan-2-yl)acrylaldehyde, *N*-methylpiperidine, and 1,8-diaza-bicyclo[5.4.0]undec-7-ene were purchased from AlfaAesar. 3-Iodobenzaldehyde and 4-iodobenzaldehyde were purchased from Apollo Scientific. 3-Fluorobenzaldehyde, 4-(trifluoromethoxy)benzaldehyde, 5-bromo-2-furaldehyde, 4-trifluoromethylbenzaldehyde, 2-naphthaldehyde, salicylaldehyde, *trans*-4-methoxycinnamaldehyde, trimethylamine (THF solution), dimethylamine (40 %wt solution in water), 1,2,2,6,6-pentamethylpiperidine, *N*-methylpyrrolidine, and chlorosulfuric acid were purchased from Acros Organics. 3-Chlorobenzaldehyde, 6-methoxy-2-naphthaldehyde, 2-fluoropyridine, 2,4-difluoropyridine, 2-hydroxy-4-methoxybenzaldehyde, *trans*-2-methoxycinnamaldehyde, *trans*-2-chlorocinnam-aldehyde, *trans*-3-methylcinnamaldehyde, 1-boc-4-(4-formylphenyl)-piperazine, *trans*-4-chlorocinnamaldehyde and (*E*)-3-(3-(4-fluorophenyl)-1-isopropyl-1*H*-indol-2-yl)acryl-aldehyde were purchased from Ambeed. *Trans*-4-(dimethylamino)-cinnamaldehyde was purchased from Angene. Potassium bifluoride was purchased from Honeywell. *o*-Anisaldehyde was purchased from Koch-Light. Quinuclidine was purchased from Chemat. Acetic acid was purchased from Chempur. Paraformaldehyde, acetonitrile and triethylamine were purchased from POCh. Commercially available solvents and materials were used without further purification.

2. Synthesis of Methanedisulfonyl Fluoride (1, MDSF)

2.1. Optimization

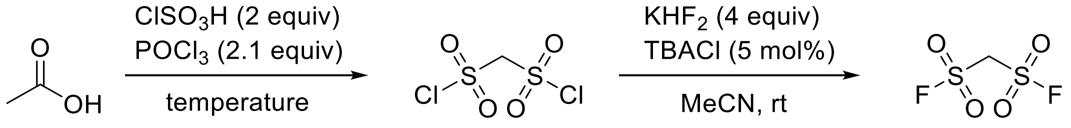

acetic acid *methanedisulfonyl chloride* **1**, MDSF

	yield of the 1st step		yield of the 2nd step
**1st step conditions:**	ClO_2S SO_2Cl	**2nd step conditions:**	FO_2S SO_2F
1+3 h; 105+145 °C, 0.5 mol of AcOH,	**53 %**	KHF_2 4 eq, TBACl 5%, 2 mol/l in MeCN	**75 %**
1+17 h; 105+145 °C, 0.5 mol of AcOH	**67 %**	KHF_2 4 eq, TBACl 5%, 2 mol/l in MeCN	**75 %**
1+7 h; 105+145 °C, 0.5 mol of AcOH	**66 %**	KHF_2 4 eq, TBACl 5%, 2 mol/l in MeCN	**78 %**
1+7 h; 105+145 °C, 0.5 mol of AcOH	**76 %**	KHF_2 4 eq, TBACl 5%, 2 mol/l in MeCN	**89%**
1+7 h; 105+145 °C, 0.5 mol of AcOH	**73 %***	**-**	**-**
1+7 h; 105+145 °C, 1 mol of AcOH	**77 %**	KHF_2 4 eq, TBACl 2.5%, 2.8 mol/l in MeCN	**87 %**

* - synthesis of methanedisulfonyl chloride was repeated under the same conditions

2.2. Preparation of Methanedisulfonyl Chloride (Optimal Conditions)

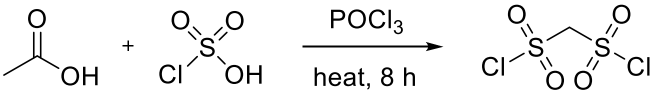

A 1 l round-bottomed flask fitted with reflux condenser was charged with chlorosulfuric acid (135 ml, 2.0 mol), phosphorus(V) oxychloride (195 ml, 2.1 mol), and acetic acid (58 ml, 1.0 mol). The flask was immersed in oil bath and heated at 105 °C for 1 h. Then, temperature was raised to 145 °C. After total time of 8 h the mixture was cooled to rt, and distilled directly

from the reaction flask equipped with Vigreux column. Organic fraction was collected as the second one (first fraction contained mainly inorganic acid) to obtain **methanedisulfonyl chloride**, as a slightly yellowish oil, b.p. 95-105 °C at 2-8×10^{-2} mbar (164.1 g; 0.77 mol; 77%). ^{1}H NMR (400 MHz, $CDCl_3$) δ 5.58 (s, 2H). ^{13}C NMR (100 MHz, $CDCl_3$) δ 84.4. The ^{1}H NMR spectrum was consistent with that described in the literature: A. Castro, S. K. Erickson, I. Shechter, T. A. Spencer, *Bioorg. Chem.* **1996**, *24*, 242-250.

2.3. Preparation of Methanedisulfonyl Fluoride (Optimal Conditions)

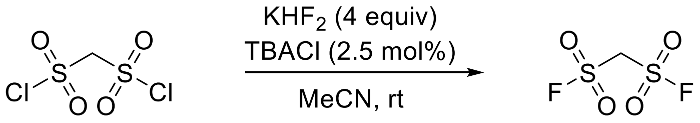

1, MDSF

A 1 l round-bottomed flask was charged with KHF_2 (242 g; 3.1 mol), tetrabutylammonium chloride (5.44 g; 19.4 mmol; 2.5 mol%), and dried for 1 h at rt *in vacuo* (2-5×10^{-2} mbar). Then, freshly distilled acetonitrile (270 ml) and methanedisulfonyl chloride (164 g; 0.77 mol) were added. The mixture was stirred mechanically (500 rpm) for 5 h at rt (water cooling bath was applied to avoid overheating), and DCM (100 ml) was added. Precipitate was filtered off using Schott funnel with 1 cm celite layer bed, and washed with DCM (300 ml). Combined filtrates were evaporated and crude product was distilled under reduced pressure with Vigreux column to obtain **methanedisulfonyl fluoride** (**1**, MDSF) as a colorless liquid, b.p. 69-71 °C (8 mbar) (129.5 g; 0.67 mol; 86%). ^{1}H NMR (400 MHz, $CDCl_3$) δ 5.18 (t, *J*=3.8 Hz, 2H). ^{13}C NMR (100 MHz, $CDCl_3$) δ 63.8 (t, *J*=26.3 Hz). ^{19}F NMR (376 MHz, $CDCl_3$) δ 63.8 (t, *J*=4.1 Hz).

3. Synthesis of β-Arylethenesulfonyl Fluorides, 2

3.1. Optimization

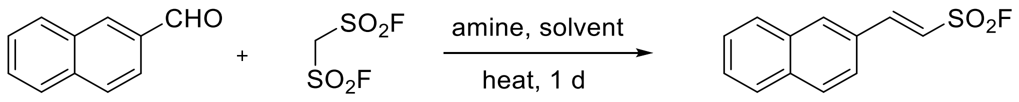

1 **2u**

A 30 ml Schlenk flask was charged with 2-naphthaldehyde (312 mg; 2 mmol), methanedisulfonyl fluoride (**1**; 720 mg; 4 mmol) and flushed with argon. Then, dry solvent (4 ml) was added. The reaction flask was immersed in oil bath, heated to appropriate temperature, and amine was added. After 24 h the reaction mixture was cooled to rt, poured on water (25 ml), and extracted with ethyl acetate (3×25 ml). Combined organic phases were washed with brine (25 ml), dried with $MgSO_4$, filtered off and evaporated. The product was purified by column chromatography (eluent: cyclohexane/toluene 4:1, 2:1, 1:1). Results of the experiments are collected in the table below.

Entry	T(°C)	Solvent	Amine	Molar ratio CHO:MDSF:amine	Isolated yield of 2u
**1**	65	THF	NEt_3	1.0:2.0:2.1	54
**2**	65	THF	NMe_3	1.0:2.0:2.1	56
**3**	65	THF	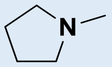	1.0:2.0:2.1	70
**4**	65	THF	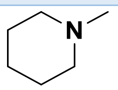	1.0:2.0:2.1	63
**5**	65	THF	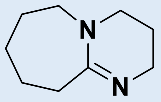	1.0:2.0:2.1	36
**6**	65	THF	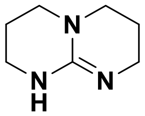	1.0:2.0:2.1	47
**7**	65	THF	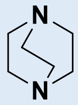	1.0:2.0:1.05	45
**8**	65	THF	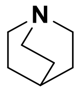	1.0:2.0:2.1	61
**9**	65	THF	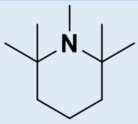	1.0:2.0:2.1	43

**10**	65	THF	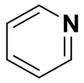	1.0:2.0:2.1	19
**11**	65	THF	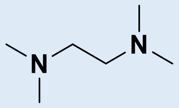	1.0:2.0:2.1	36
**12**	65	toluene	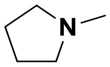	1.0:2.0:2.1	68
**13**	65	acetonitrile	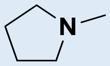	1.0:2.0:2.1	71
**14**	100	1,4-dioxane	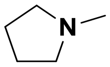	1.0:2.0:2.1	55
**15**	80	toluene	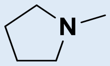	1.0:2.0:2.1	63
**16**	80	acetonitrile	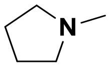	1.0:2.0:2.1	72
**17**	40	acetonitrile	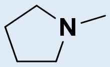	1.0:2.0:2.1	63
**18**	40	acetonitrile (0.8 ml)	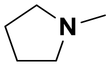	1.0:2.0:2.1	61
**19**	65	THF	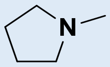	1.0:2.0:1.9	60
**20**	65	THF	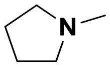	1.0:3.5:3.6	69
**21**	65	THF	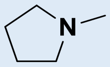	1.0:5.0:5.1	65
**22**	65	THF	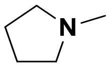	1.0:2.0:2.1	60[a]

[a] - Aldehyde was added to the reaction mixture of **1** and amine in THF after 1 h of heating

3.2. Other Processes Occurring During Reaction

In one of the optimization experiments presented in the table above (entry 14, dioxane, 100 °C) we isolated olefination product **2u** (260 mg, 1.10 mmol, 55%), accompanied with recovered 2-naphthaldehyde (18 mg, 0.12 mmol, 6%) and unexpected

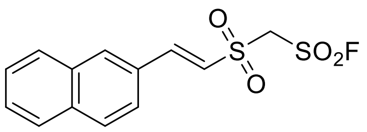

(E)-[2-(2-naphthyl)ethensulfonyl]methylsulfonyl fluoride (14.0 mg; 0.04 mmol; 2%); white solid; 155-171°C (dec). ^{1}H NMR (400 MHz, $CDCl_3$) δ 8.06-7.80 (m, 5H), 7.67-7.51 (m, 3H), 7.13 (d, *J*=15.3 Hz, 1H), 4.91 (d, *J*=3.4 Hz, 2H). ^{13}C NMR (100 MHz, $CDCl_3$) δ 149.8, 135.2, 133.0, 132.6, 129.4, 129.0, 128.6, 128.6, 127.9, 127.3, 123.2, 122.3, 69.3 (d, *J*=19.5 Hz). ^{19}F NMR (376 MHz, $CDCl_3$) δ 63.6 (t, *J*=3.7 Hz). MS (EI) *m/z*: (%) 314 (95, [M^+]), 217 (31), 168 (100), 152 (100), 127 (14). HRMS (EI) *m/z*: Calc. for $C_{13}H_{11}O_4FS_2$ [M^+] 314.0079. Found 314.0083.

3.3. Preparation of β-Arylethenesulfonyl Fluorides (General Procedure)

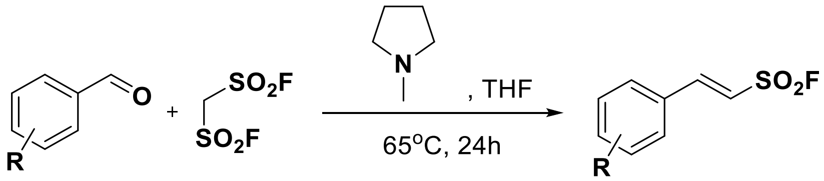

1 2a-2w

A 30 ml Schlenk flask was charged with arylaldehyde (5.0 mmol) and flushed with argon. Then, dry THF (10 ml) and methanedisulfonyl fluoride (**1**; 1.801 g; 10 mmol) were added. The reaction mixture was placed in oil bath at 65 °C and after warming *N*-methylpyrrolidine (1.1 ml; 10.5 mmol) was added via syringe. After 24 h the reaction mixture was cooled to rt, poured on water (50 ml), and extracted with ethyl acetate (3×50 ml). Combined organic phases were washed with brine (50 ml), dried with $MgSO_4$, filtered off and evaporated. The products were purified by column chromatography (eluent: cyclohexane/toluene 4:1, 2:1, 1:1).

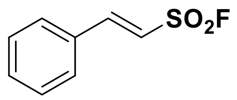

2a, (E)-2-phenylethenesulfonyl fluoride (368.0 mg; 1.98 mmol at 3 mmol scale; 66%); white solid; m.p. 96.0-97.0°C (lit. 96.5-97.5°C: A. Talko, D. Antoniak, M. Barbasiewicz, *Synthesis* **2019**, *51*, 2278-2286). ^{1}H NMR (400 MHz, $CDCl_3$) δ 7.79 (d, *J*=15.5 Hz, 1H), 7.58–7.41 (m, 5H), 6.86 (dd, *J*=15.5, 2.6 Hz, 1H). ^{13}C NMR (100 MHz, $CDCl_3$) δ 148.8 (d, *J*=2.7 Hz), 132.6, 130.9 (d, *J*=1.4 Hz), 129.4, 129.0, 117.8 (d, *J*=28.0 Hz). ^{19}F NMR (376 MHz, $CDCl_3$) δ 61.8. The ^{1}H, ^{13}C and ^{19}F NMR spectra were consistent with those described in the literature: A. Talko, D. Antoniak, M. Barbasiewicz, *Synthesis* **2019**, *51*, 2278-2286.

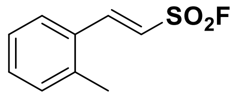

2b, (E)-2-(2-methylphenyl)ethenesulfonyl fluoride (597.9 mg; 2.99 mmol; 60%); white solid; m.p. 35.5-36.5°C (lit. 27-28°C: G.-F. Zha, Q. Zheng, J. Leng, P. Wu, H.-L. Qin, K. B. Sharpless, *Angew. Chem. Int. Ed.* **2017**, *56*, 4849-4852.). ^{1}H NMR (400 MHz, $CDCl_3$) δ 8.08 (d, *J*=15.4 Hz, 1H), 7.52 (dd, *J*=8.1, 1.3 Hz, 1H), 7.39 (td, *J*=7.5, 1.4 Hz, 1H), 7.31–7.21 (m, 2H), 6.78 (dd, *J*=15.4, 2.5 Hz, 1H), 2.46 (s, 3H). ^{13}C NMR (100 MHz, $CDCl_3$) δ 146.5 (d, *J*=2.7 Hz), 139.0, 132.4, 131.4, 129.9, 127.2, 126.8, 118.7 (d, *J*=27.8 Hz), 19.7. ^{19}F NMR (376 MHz, $CDCl_3$) δ 61.6. The ^{1}H, ^{13}C and ^{19}F NMR spectra were consistent with those described in the literature: G.-F. Zha, Q. Zheng, J. Leng, P. Wu, H.-L. Qin, K. B. Sharpless, *Angew. Chem. Int. Ed.* **2017**, *56*, 4849-4852.

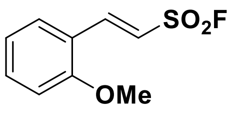

2c, (E)-2-(2-methoxyphenyl)ethenesulfonyl fluoride (703.9 mg; 3.26 mmol; 65%); white solid; m.p. 48.0-49.5°C. ^{1}H NMR (400 MHz, $CDCl_3$) δ 7.91 (d, *J*=15.5 Hz, 1H), 7.46 (ddd, *J*=8.3, 7.4, 1.7 Hz, 1H), 7.42 (dd, *J*=7.6, 1.8 Hz, 1H), 7.14 (dd, *J*=15.5, 2.4 Hz, 1H), 7.01 (td, *J*=7.5, 1.0 Hz, 1H), 6.97 (d, *J*=8.4 Hz, 1H), 3.93 (s, 4H). ^{13}C NMR (100 MHz, $CDCl_3$) δ 159.5, 144.9 (d, *J*=2.8 Hz), 133.9, 132.1, 121.0, 119.9, 118.6 (d, *J*=26.7 Hz), 111.4, 55.6. ^{19}F NMR (376 MHz, $CDCl_3$) δ 61.8. The ^{1}H, ^{13}C and ^{19}F NMR spectra were consistent with those described in the literature: P. K. Chinthakindi, K. B. Govender, A. S. Kumar, H. G. Kruger, T. Govender, T. Naicker, P. I. Arvidsson, *Org. Lett.* **2017**, *19*, 480-483.

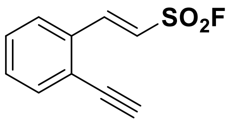

2d, (E)-2-(2-ethynylphenyl)ethenesulfonyl fluoride (451.9 mg; 2.15 mmol; 43%); white solid; m.p. 85.5-87.0°C. ^{1}H NMR (400 MHz, $CDCl_3$) δ 8.24 (dd, *J*=15.7, 1.1 Hz, 1H), 7.59 (ddd, *J*=7.2, 6.4, 2.0 Hz, 3H), 7.48–7.39 (m, 3H), 7.05 (dd, *J*=15.6, 2.5 Hz, 1H), 3.50 (s, 1H). ^{13}C NMR (100 MHz, $CDCl_3$) δ 146.2 (d, *J*=3.0 Hz), 134.1, 132.5, 131.9, 129.4, 127.5, 123.8, 119.9 (d, *J*=28.3 Hz), 85.0, 80.1. ^{19}F NMR (376 MHz, $CDCl_3$) δ 61.7. MS (EI) *m/z*: (%) 210 (21, [M^+]), 127 (100), 115 (62), 77 (12). HRMS (EI) *m/z*: Calc. for $C_{10}H_7O_2FS$ [M^+] 210.0151. Found 210.0154

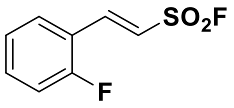

2e, (E)-2-(2-fluorophenyl)ethenesulfonyl fluoride (565.3 mg; 2.77 mmol; 55%); white solid; m.p. 55.5-57.5°C (lit. 37-38°C: G.-F. Zha, G. A. L. Bare, J. Leng, Z.-P. Shang, Z. Luo, H.-L. Qin, *Adv. Synth. Catal.* **2017**, *359*, 3237-3242). ^{1}H NMR (400 MHz, $CDCl_3$) δ 7.84 (d, *J*=15.7 Hz, 1H), 7.54–7.44 (m, 2H), 7.24 (td, *J*=7.6, 1.1 Hz, 2H), 7.21–7.14 (m, 1H), 7.04 (ddd, *J*=15.7, 2.5, 0.8 Hz, 1H). ^{13}C NMR (100 MHz, $CDCl_3$) δ 161.9 (d, *J*=257.1 Hz), 141.9 (dd, *J*=3.1, 1.9 Hz), 134.3 (d, *J*=9.2 Hz), 131.0 (d, *J*=2.2 Hz), 125.0 (d, *J*=3.6 Hz), 121.0 (dd, *J*=28.3, 10.4 Hz), 119.3 (d, *J*=11.1 Hz), 116.7 (d, *J*=21.4 Hz). ^{19}F NMR (376 MHz, $CDCl_3$) δ 61.3, -111.5 (ddd, *J*=10.9, 7.2, 5.4 Hz). The ^{1}H, ^{13}C and ^{19}F NMR spectra were consistent with those described in the literature: G.-F. Zha, G. A. L. Bare, J. Leng, Z.-P. Shang, Z. Luo, H.-L. Qin, *Adv. Synth. Catal.* **2017**, *359*, 3237-3242.

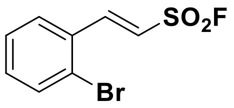

2f, (E)-2-(2-bromophenyl)ethenesulfonyl fluoride (491.0 mg; 1.85 mmol; 37%); white solid; m.p. 31.5-32.5°C. ^{1}H NMR (400 MHz, $CDCl_3$) δ 8.19 (dd, *J*=15.5, 0.6 Hz, 1H), 7.67 (dd, *J*=7.8, 1.5 Hz, 1H), 7.58 (dd, *J*=7.6, 1.9 Hz, 1H), 7.40

(td, *J*=7.7, 1.5 Hz, 1H), 7.37–7.32 (m, 1H), 6.86 (dd, *J*=15.5, 2.4 Hz, 1H). ^{13}C NMR (100 MHz, $CDCl_3$) δ 147.1 (d, *J*=3.0 Hz), 134.0, 133.4, 131.1, 128.5, 128.1, 126.1, 120.6 (d, *J*=28.7 Hz). ^{19}F NMR (376 MHz, $CDCl_3$) δ 61.5. The ^{1}H, ^{13}C and ^{19}F NMR spectra were consistent with those described in the literature: P. K. Chinthakindi, K. B. Govender, A. S. Kumar, H. G. Kruger, T. Govender, T. Naicker, P. I. Arvidsson, *Org. Lett.* **2017**, *19*, 480-483.

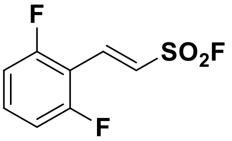

2g, (E)-2-(2,6-difluorophenyl)ethenesulfonyl fluoride (521.5 mg; 2.35 mmol; 47%); white solid; m.p. 76.0-76.5°C. ^{1}H NMR (400 MHz, $CDCl_3$) δ 7.90 (d, *J*=15.9 Hz, 1H), 7.47 (tt, *J*=8.4, 6.4 Hz, 1H), 7.21 (dd, *J*=15.9, 2.4 Hz, 1H), 7.07–6.96 (m, 2H). ^{13}C NMR (100 MHz, $CDCl_3$) δ 161.9 (dd, *J*=258.6, 5.8 Hz), 134.7 (q, *J*=3.1 Hz), 134.2 (t, *J*=11.3 Hz), 123.6 (dt, *J*=28.6, 9.8 Hz), 112.6–111.9 (m), 109.5 (td, *J*=14.5, 1.2 Hz). ^{19}F NMR (376 MHz, $CDCl_3$) δ 60.9 (d, *J*=2.6 Hz), -108.8 (dd, *J*=9.1, 6.4 Hz). MS (EI) *m/z*: (%) 222 (80, [M^+]), 138 (100), 119 (14). HRMS (EI) *m/z*: Calc. for $C_8H_5O_2F_3S$ [M^+] 221.9962. Found 221.9954.

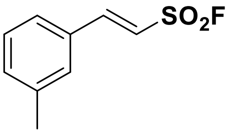

2h, (E)-2-(3-methylphenyl)ethenesulfonyl fluoride (696.5 mg; 3.48 mmol; 70%); white solid; m.p. 33.0-33.5°C (lit. 29-30°C: G.-F. Zha, Q. Zheng, J. Leng, P. Wu, H. L. Qin, K. B. Sharpless, *Angew. Chem. Int. Ed.* **2017**, *56*, 4849-4852). ^{1}H NMR (400 MHz, $CDCl_3$) δ 7.76 (dd, *J*=15.5, 1.2 Hz, 1H), 7.39–7.27 (m, 4H), 6.83 (dd, *J*=15.5, 2.5 Hz, 1H), 2.39 (s, 3H). ^{13}C NMR (100 MHz, $CDCl_3$) δ 149.1 (d, *J*=2.7 Hz), 139.3, 133.5, 130.9, 129.6, 129.3, 126.3, 117.6 (d, *J*=27.9 Hz), 21.3. ^{19}F NMR (376 MHz, $CDCl_3$) δ 61.8. The ^{1}H, ^{13}C and ^{19}F NMR spectra were consistent with those described in the literature: G.-F. Zha, Q. Zheng, J. Leng, P. Wu, H. L. Qin, K. B. Sharpless, *Angew. Chem. Int. Ed.* **2017**, *56*, 4849-4852.

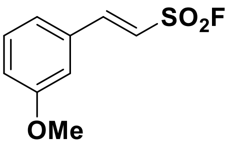

2i, (E)-2-(3-methoxyphenyl)ethenesulfonyl fluoride (694.6 mg; 3.21 mmol; 64%); white solid; m.p. 72.0-72.5°C (lit. 71-72°C: G. F. Zha, Q. Zheng, J. Leng, P. Wu, H. L. Qin, K. B. Sharpless, *Angew. Chem. Int. Ed.* **2017**, *56*, 4849-4852). ^{1}H NMR (400 MHz, $CDCl_3$) δ 7.76 (dd, *J*=15.4, 1.2 Hz, 1H), 7.37 (dd, *J*=8.1, 7.7 Hz, 1H), 7.12 (d, *J*=7.6 Hz, 1H), 7.07–7.00 (m, 2H), 6.83 (dd, *J*=15.5, 2.5 Hz, 1H), 3.83 (s, 3H). ^{13}C NMR (100 MHz, $CDCl_3$) δ 160.1, 148.8 (d, *J*=2.7 Hz), 132.2, 130.4, 121.6, 118.5, 118.2 (d, *J*=28.1 Hz), 113.8, 55.5. ^{19}F NMR (376 MHz, $CDCl_3$) δ 61.8. The ^{1}H, ^{13}C and

^{19}F NMR spectra were consistent with those described in the literature: P. K. Chinthakindi, K. B. Govender, A. S. Kumar, H. G. Kruger, T. Govender, T. Naicker, P. I. Arvidsson, *Org. Lett.* **2017**, *19*, 480-483.

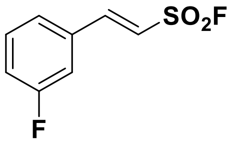

2j, (E)-2-(3-fluorophenyl)ethenesulfonyl fluoride (498.5 mg; 2.44 mmol; 49%); white solid; m.p. 74.0-74.5°C (lit. 69-70°C: G.-F. Zha, Q. Zheng, J. Leng, P. Wu, H.-L. Qin, K. B. Sharpless, *Angew. Chem. Int. Ed.* **2017**, *56*, 4849-4852). ^{1}H NMR (400 MHz, $CDCl_3$) δ 7.76 (d, *J*=15.5 Hz, 1H), 7.50–7.40 (m, 4H), 7.33 (d, *J*=7.8 Hz, 1H), 7.24 (s, 3H), 6.86 (dd, *J*=15.5, 2.5 Hz, 1H). ^{13}C NMR (100 MHz, $CDCl_3$) δ 163.0 (d, *J*=249.0 Hz), 147.3 (t, *J*=2.8 Hz), 133.0 (d, *J*=8.9 Hz), 131.1 (d, *J*=8.2 Hz), 125.1 (d, *J*=3.1 Hz), 119.6 (d, *J*=21.2 Hz), 119.4, 115.3 (d, *J*=22.4 Hz). ^{19}F NMR (376 MHz, $CDCl_3$) δ 61.6, -111.4 (td, *J*=8.7, 5.7 Hz). The ^{1}H, ^{13}C and ^{19}F NMR spectra were consistent with those described in the literature: G. F. Zha, Q. Zheng, J. Leng, P. Wu, H. L. Qin, K. B. Sharpless, *Angew. Chem. Int. Ed.* **2017**, *56*, 4849-4852.

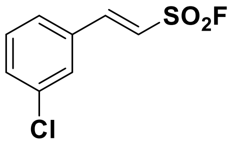

2k, (E)-2-(3-chlorophenyl)ethenesulfonyl fluoride (538.0 mg; 2.44 mmol; 49%); white solid; m.p. 65.0-67.0°C (lit. 68-70°C: X.-Y. Chen, Y. Wu, J. Zhou, P. Wang, and J.-Q. Yu, *Org. Lett.* **2019**, *21*, 1426-1429). ^{1}H NMR (400 MHz, $CDCl_3$) δ 7.73 (dd, *J*=15.5, 1.2 Hz, 1H), 7.56–7.50 (m, 1H), 7.50–7.45 (m, 1H), 7.44–7.37 (m, 2H), 6.87 (dd, *J*=15.6, 2.6 Hz, 1H). ^{13}C NMR (100 MHz, $CDCl_3$) δ 147.1 (d, *J*=2.8 Hz), 135.6, 132.6, 132.5, 130.7, 128.6, 127.2, 119.6 (d, *J*=28.7 Hz). ^{19}F NMR (376 MHz, $CDCl_3$) δ 61.6. The ^{1}H, ^{13}C and ^{19}F NMR spectra were consistent with those described in the literature: X.-Y. Chen, Y. Wu, J. Zhou, P. Wang, and J.-Q. Yu, *Org. Lett.* **2019**, *21*, 1426-1429.

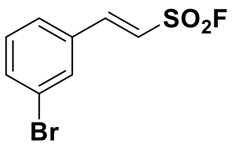

2l, (E)-2-(3-bromophenyl)ethenesulfonyl fluoride (524.3 mg; 1.98 mmol; 40%); white solid; m.p. 79.0-79.5°C (lit. 81-82°C: P. K. Chinthakindi, K. B. Govender, A. S. Kumar, H. G. Kruger, T. Govender, T. Naicker, P. I. Arvidsson, *Org. Lett.* **2017**, *19*, 480-483). ^{1}H NMR (400 MHz, $CDCl_3$) δ 7.75–7.67 (m, 2H), 7.63 (ddd, *J*=8.0, 1.9, 1.0 Hz, 1H), 7.46 (d, *J*=7.8 Hz, 1H), 7.34 (t, *J*=7.9 Hz, 1H), 6.87 (dd, *J*=15.5, 2.5 Hz, 1H). ^{13}C NMR (100 MHz, $CDCl_3$) δ 147.0 (d, *J*=2.9 Hz), 135.4, 132.9 (d, *J*=1.2 Hz), 131.6, 130.9, 127.6, 123.5, 119.6 (d, *J*=28.7 Hz).^{19}F NMR (376 MHz, $CDCl_3$) δ

61.7. The ^{1}H, ^{13}C and ^{19}F NMR spectra were consistent with those described in the literature: P. K. Chinthakindi, K. B. Govender, A. S. Kumar, H. G. Kruger, T. Govender, T. Naicker, P. I. Arvidsson, *Org. Lett.* **2017**, *19*, 480-483.

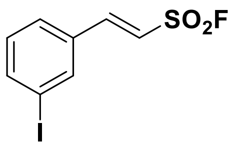

2m, (E)-2-(3-iodophenyl)ethenesulfonyl fluoride (743.4 mg; 2.38 mmol at 4.3 mmol scale; 55%); white solid; m.p. 96.0-97.0°C (lit. 87-88°C: G.-F. Zha, Q. Zheng, J. Leng, P. Wu, H.-L. Qin, K. B. Sharpless, *Angew. Chem. Int. Ed.* **2017**, *56*, 4849-4852). ^{1}H NMR (400 MHz, $CDCl_3$) δ 7.89 (t, *J*=1.8 Hz, 1H), 7.83 (ddd, *J*=7.9, 1.7, 1.0 Hz, 1H), 7.69 (d, *J*=15.5 Hz, 1H), 7.49 (d, *J*=7.9 Hz, 1H), 7.20 (t, *J*=7.8 Hz, 1H), 6.85 (dd, *J*=15.5, 2.5 Hz, 1H). ^{13}C NMR (100 MHz, $CDCl_3$) δ 146.9 (d, *J*=2.8 Hz), 141.3, 137.5, 132.9 (d, *J*=1.1 Hz), 130.9, 128.1, 119.4 (d, *J*=28.7 Hz), 94.9. ^{19}F NMR (376 MHz, $CDCl_3$) δ 61.7. The ^{1}H, ^{13}C and ^{19}F NMR spectra were consistent with those described in the literature: G. F. Zha, Q. Zheng, J. Leng, P. Wu, H. L. Qin, K. B. Sharpless, *Angew. Chem. Int. Ed.* **2017**, *56*, 4849-4852.

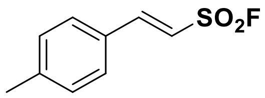

2n, (E)-2-(4-methylphenyl)ethenesulfonyl fluoride (413.0 mg; 2.06 mmol at 3 mmol scale; 69%); white solid; m.p. 132.0-133.0°C (131-133°C: X.-Y. Chen, Y. Wu, J. Zhou, P. Wang, and J.-Q. Yu, *Org. Lett.* **2019**, *21*, 1426-1429). ^{1}H NMR (400 MHz, $CDCl_3$) δ 7.76 (d, *J*=15.5 Hz, 1H), 7.43 (d, *J*=8.2 Hz, 2H), 7.25 (d, *J*=8.0 Hz, 2H), 6.79 (dd, *J*=15.5, 2.6 Hz, 1H), 2.40 (s, 3H). ^{13}C NMR (100 MHz, $CDCl_3$) δ 148.9 (d, *J*=2.7 Hz), 143.6, 130.1, 129.1, 128.2 (d, *J*=1.3 Hz), 116.5 (d, *J*=27.8 Hz), 21.7. ^{19}F NMR (376 MHz, $CDCl_3$) δ 62.1. The ^{1}H, ^{13}C and ^{19}F NMR spectra were consistent with those described in the literature: X.-Y. Chen, Y. Wu, J. Zhou, P. Wang, and J.-Q. Yu, *Org. Lett.* **2019**, *21*, 1426-1429.

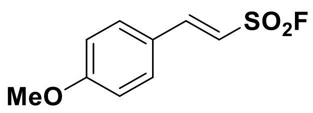

2o, (E)-2-(4-methoxyphenyl)ethenesulfonyl fluoride (355 mg; 1.64 mmol at 3 mmol scale; 55%); white solid; m.p. 76.0-77.0°C (lit. 70-71°C: G.-F. Zha, Q. Zheng, J. Leng, P. Wu, H.-L. Qin, K. B. Sharpless, *Angew. Chem. Int. Ed.* **2017**, *56*, 4849-4852). ^{1}H NMR (400 MHz, $CDCl_3$) δ 7.73 (d, *J*=15.4 Hz, 1H), 7.49 (d, *J*=8.8 Hz, 2H), 6.95 (d, *J*=8.8 Hz, 2H), 6.68 (dd, *J*=15.4, 2.6 Hz, 1H), 3.86 (s, 3H). ^{13}C NMR (100 MHz, $CDCl_3$) δ 163.2, 148.6 (d, *J*=2.5 Hz), 131.1, 123.5 (d, *J*=1.2 Hz), 114.8, 114.6 (d, *J*=27.5 Hz), 55.5. ^{19}F NMR (376 MHz, $CDCl_3$) δ 63.0. The ^{1}H, ^{13}C and ^{19}F NMR spectra were

consistent with those described in the literature: G. F. Zha, Q. Zheng, J. Leng, P. Wu, H. L. Qin, K. B. Sharpless, *Angew. Chem. Int. Ed.* **2017**, *56*, 4849-4852.

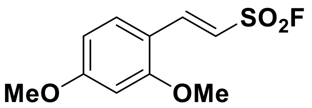

2p, (E)-2-(2,4-(dimethoxy)phenyl)ethenesulfonyl fluoride reaction was carried out at rt, (313.2 mg; 1.27 mmol; 25%); white solid; m.p. 81.5-83.5°C. ^{1}H NMR (400 MHz, $CDCl_3$) δ 7.80 (dd, *J*=15.4, 1.0 Hz, 1H), 7.34 (d, *J*=8.6 Hz, 1H), 6.99 (dd, *J*=15.4, 2.5 Hz, 1H), 6.53 (dd, *J*=8.6, 2.3 Hz, 1H), 6.46 (d, *J*=2.4 Hz, 1H), 3.90 (s, 3H), 3.85 (s, 3H). ^{13}C NMR (100 MHz, $CDCl_3$) δ 164.6, 161.2, 144.8 (d, *J*=2.4 Hz), 134.1, 115.2 (d, *J*=26.1 Hz), 113.3 (d, *J*=1.4 Hz), 105.8, 98.6, 55.6. ^{19}F NMR (376 MHz, $CDCl_3$) δ 62.6. MS (EI) *m/z*: (%) 246 (100, [M^+]), 162 (24), 149 (49), 121 (60), 77 (19). HRMS (EI) *m/z*: Calc. for $C_{10}H_{11}O_4FS$ [M^+] 246.0362. Found 246.0370.

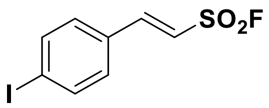

2r, (E)-2-(4-iodophenyl)ethenesulfonyl fluoride (822.5 mg; 2.64 mmol at 4.8 mmol scale; 55%); white solid; m.p. 166.0-167.0°C (lit. 152-153°C: G.-F. Zha, G. A. L. Bare, J. Leng, Z.-P. Shang, Z. Luo, H.-L. Qin, *Adv. Synth. Catal.* **2017**, *359*, 3237-3242). ^{1}H NMR (400 MHz, $CDCl_3$) δ 7.83 (d, *J*=8.5 Hz, 2H), 7.73 (d, *J*=15.5 Hz, 1H), 7.27 (d, *J*=8.1 Hz, 2H), 6.89 (dd, *J*=15.5, 2.5 Hz, 1H). ^{13}C NMR (100 MHz, $CDCl_3$) δ 147.6 (d, *J*=2.9 Hz), 138.7, 130.3, 130.2, 118.6 (d, *J*=28.6 Hz). 99.7. ^{19}F NMR (376 MHz, $CDCl_3$) δ 61.8. The ^{1}H, ^{13}C and ^{19}F NMR spectra were consistent with those described in the literature: G.-F. Zha, G. A. L. Bare, J. Leng, Z.-P. Shang, Z. Luo, H.-L. Qin, *Adv. Synth. Catal.* **2017**, *359*, 3237-3242.

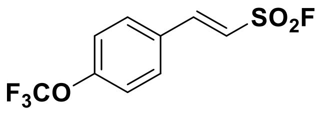

2s, (E)-2-(4-(trifluoromethoxy)phenyl)ethenesulfonyl fluoride (458.8 mg; 1.70 mmol at 4.1 mmol scale; 41%); white solid; m.p. 116.0-117.0°C (lit. 118-119°C: G.-F. Zha, Q. Zheng, J. Leng, P. Wu, H.-L. Qin, K. B. Sharpless, *Angew. Chem. Int. Ed.* **2017**, *56*, 4849-4852). ^{1}H NMR (400 MHz, $CDCl_3$) δ 7.78 (d, *J*=15.7 Hz, 1H), 7.59 (d, *J*=8.8 Hz, 2H), 7.30 (dd, *J*=8.9, 1.1 Hz, 2H), 6.85 (dd, *J*=15.5, 2.5 Hz, 1H). ^{13}C NMR (100 MHz, $CDCl_3$) δ 152.1 (q, *J*=1.7 Hz), 147.0 (d, *J*=2.9 Hz), 130.7, 129.4 (d, *J*=1.2 Hz), 121.4 (d, *J*=1.1 Hz), 120.3 (q, *J*=259.0 Hz), 118.9 (d, *J*=28.6 Hz). ^{19}F NMR (376 MHz, $CDCl_3$) δ 61.7, -58.2. The ^{1}H and ^{19}F NMR spectra were consistent with those described in the literature: G. F. Zha, Q. Zheng, J. Leng, P. Wu, H. L. Qin, K. B. Sharpless, *Angew. Chem. Int. Ed.* **2017**, *56*, 4849-4852.

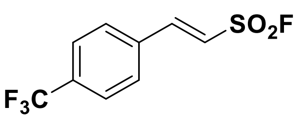

2t, (E)-2-(4-(trifluoromethyl)phenyl)ethenesulfonyl fluoride (151.0 mg; 0.59 mmol at 3 mmol scale; 20%); white solid; m.p. 128.5-129.5°C (lit. 133-134°C: G.-F. Zha, Q. Zheng, J. Leng, P. Wu, H.-L. Qin, K. B. Sharpless, *Angew. Chem. Int. Ed.* **2017**, *56*, 4849-4852). ^{1}H NMR (400 MHz, $CDCl_3$) δ 7.83 (d, *J*=15.6 Hz, 1H), 7.73 (d, *J*=8.3 Hz, 2H), 7.66 (d, *J*=8.3 Hz, 2H), 6.95 (dd, *J*=15.6, 2.5 Hz, 1H). ^{13}C NMR (100 MHz, $CDCl_3$) δ 146.8 (d, *J*=2.9 Hz), 134.2, 133.9 (q, *J*=33.1 Hz), 129.2, 126.4 (q, *J*=3.8 Hz), 123.4 (q, *J*=272.7 Hz), 120.8 (d, *J*=29.0 Hz). ^{19}F NMR (376 MHz, $CDCl_3$) δ 61.5, -63.7. The ^{1}H, ^{13}C and ^{19}F NMR spectra were consistent with those described in the literature: X. Nie, T. Xu, J. Song, A. Devaraj, B. Zhang, Y. Chen, S. Liao, *Angew. Chem. Int. Ed.* **2021**, *60*, 3956-3960.

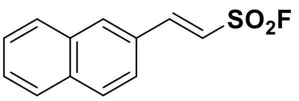

2u, (E)-2-(naphthalene-2-yl)ethane-1-sulfonyl fluoride (329.0 mg; 1.39 mmol at 2 mmol scale; 70%); white solid; m.p. 149.0-150.0°C (lit. 145-146°C: G.-F. Zha, Q. Zheng, J. Leng, P. Wu, H.-L. Qin, K. B. Sharpless, *Angew. Chem. Int. Ed.* **2017**, *56*, 4849-4852). ^{1}H NMR (400 MHz, $CDCl_3$) δ 7.98 (d, *J*=0.9 Hz, 1H), 7.94 (dd, *J*=15.5, 0.7 Hz, 1H), 7.91–7.84 (m, 3H), 7.63–7.53 (m, 3H), 6.94 (dd, *J*=15.4, 2.5 Hz, 1H). ^{13}C NMR (100 MHz, $CDCl_3$) δ 148.9 (d, *J*=2.7 Hz), 135.1, 133.0, 132.3, 129.4, 129.0, 128.6, 128.4 (d, *J*=1.1 Hz), 127.9, 127.4, 123.0, 117.7 (d, *J*=28.0 Hz). ^{19}F NMR (376 MHz, $CDCl_3$) δ 62.1 (d, *J*=2.5 Hz). The ^{1}H, ^{13}C and ^{19}F NMR spectra were consistent with those described in the literature: X. Nie, T. Xu, J. Song, A. Devaraj, B. Zhang, Y. Chen, S. Liao, *Angew. Chem. Int. Ed.* **2021**, *60*, 3956-3960.

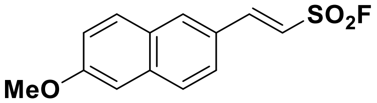

2v, (E)-2-(6-methoxynaphthalene-2-yl)ethane-1-sulfonyl fluoride (603.7 mg; 2.27 mmol; 45%); white solid; m.p. 123.0-124.0°C. ^{1}H NMR (400 MHz, $CDCl_3$) δ 7.92–7.87 (m, 2H), 7.76 (dd, *J*=8.7, 4.4 Hz, 2H), 7.55 (dd, *J*=8.6, 1.9 Hz, 1H), 7.20 (dd, *J*=9.0, 2.5 Hz, 1H), 7.14 (d, *J*=2.5 Hz, 1H), 6.86 (dd, *J*=15.4, 2.5 Hz, 1H), 3.94 (s, 3H). ^{13}C NMR (100 MHz, $CDCl_3$) δ 159.9, 149.1 (d, *J*=2.6 Hz), 136.9, 132.1, 130.6, 128.3, 128.1, 126.2 (d, *J*=1.1 Hz), 123.7, 120.2, 116.2 (d, *J*=27.8 Hz), 106.1, 55.5. ^{19}F NMR (376 MHz, $CDCl_3$) δ 62.4. MS (EI) *m/z*: (%) 266 (100, [M^+]), 223 (13), 182 (15), 139 (13). HRMS (EI) *m/z*: Calc. for $C_{13}H_{11}O_3FS$ [M^+] 266.0413. Found 266.0408.

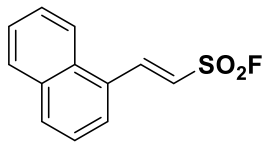

2w, (E)-2-(naphthalene-1-yl)ethane-1-sulfonyl fluoride (740.9 mg; 3.14 mmol; 63%); white solid; m.p. 94.5-95.5°C (lit. 66-67°C: G.-F. Zha, Q. Zheng, J. Leng, P. Wu, H.-L. Qin, K. B. Sharpless, *Angew. Chem. Int. Ed.* **2017**, *56*, 4849-4852). ^{1}H NMR (400 MHz, $CDCl_3$) δ 8.63 (d, *J*=15.3 Hz, 1H), 8.09 (dt, *J*=8.5, 1.0 Hz, 1H), 8.01 (dd, *J*=8.3, 1.1 Hz, 1H), 7.94–7.88 (m, 1H), 7.79–7.73 (m, 1H), 7.67–7.49 (m, 3H), 6.97 (dd, *J*=15.3, 2.5 Hz, 1H). ^{13}C NMR (100 MHz, $CDCl_3$) δ 146.0 (d, *J*=3.0 Hz), 133.7, 133.0, 131.2, 129.1, 128.0 (d, *J*=1.1 Hz), 127.9, 126.9, 126.5, 125.3, 122.6, 120.0 (d, *J*=27.7 Hz). ^{19}F NMR (376 MHz, $CDCl_3$) δ 61.5. The ^{1}H, ^{13}C and ^{19}F NMR spectra were consistent with those described in the literature: X. Nie, T. Xu, J. Song, A. Devaraj, B. Zhang, Y. Chen, S. Liao, *Angew. Chem. Int. Ed.* **2021**, *60*, 3956-3960.

3.4. Stability Studies of 2t and 2u under the Reaction Conditions

β-Arylethenesulfonyl fluorides **2u** and **2t** were separately subjected to the standard olefination conditions. A 30 ml Schlenk flask was charged with **2u** (236.1 mg) or **2u** (250.3 mg, 1 mmol scale), **1** (360 mg, 2 mmol), *N*-methylpyrrolidine (0.22 ml, 2.1 mmol) and dry THF (2 ml), and the reaction mixtures were heated at 65 °C for 24 hours according to the General Procedure. After chromatography we received recovered **2u** (223.7 mg, 95%), and **2t** (106.8 mg, 42%), respectively.

4. Synthesis of Sulfocoumarines, 3

4.1. Optimization

The experiments were carried out according to the General Procedure for the olefination reaction, at 2 mmol scale of 4-methylsalicylaldehyde at temperatures given in the table below (reaction time: 24 h).

Entry	T(°C)	Solvent	Concentration of aldehyde (M)	Molar ratio aldehyde: MDSF:amine	Amine	Yield (%)
**1**	65	THF	0.5	1 : 2 : 2.1	*N*-methyl-pyrrolidine	29
**2**	20	THF	0.5	1 : 2 : 2.2	*N*-methyl-pyrrolidine	0
**3**	65	THF	0.5	1 : 1.2 : 1.3	*N*-methyl-pyrrolidine	0[a]
**4**	20	MeCN	0.5	1 : 1.2 : 1.3	Cs_2CO_3	0
**5**	65	DCE	0.5	1 : 1.2 : 1.3	pyridine	25
**6**	65	DCE	0.5	1 : 2 : 2.1	pyridine	49
**7**	65	DCE	1	1 : 2 : 2.1	pyridine	67
**8**	85	DCE	0.5	1 : 2 : 2.1	pyridine	41
**9**	85	DCE	1	1 : 2 : 2.1	pyridine	53
**10**	85	DCE	1	1 : 2 : 4	pyridine	17

[a] - 5% of **3a** was observed by ^{1}H NMR

In further studies it occurred that for the synthesis of sulfocoumarins it is beneficial to use 1 : 2 : 1.9 reagents ratio and prolonged reaction time (up to 7 d).

4.2. Preparation of Sulfocoumarines (Optimal Conditions)

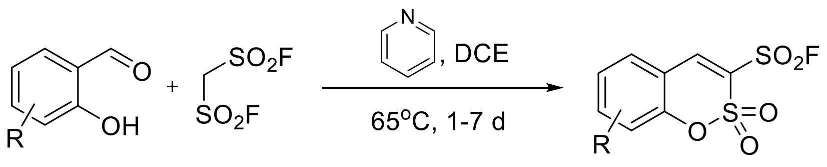

1 **3a-3d**

A 30 ml Schlenk flask was charged with salicylaldehyde (2 mmol) and flushed with argon. Then dry DCE (2 ml) and methanedisulfonyl fluoride (**1**; 720.6 mg; 4 mmol) were added. The reaction flask was immersed in oil bath at 65 °C and after warming, pyridine (0.31 ml; 3.8

mmol) was added via syringe. After 1-7 d the reaction mixture was cooled to rt, poured on water (25 ml) and extracted with ethyl acetate (3×25 ml). Combined organic phases were washed with brine (25 ml), dried with $MgSO_4$, filtered off and evaporated. The products were purified by column chromatography (eluent: cyclohexane/toluene 2:1, 1:1)

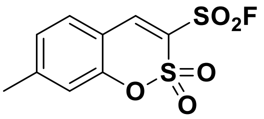

3a, 3-fluorosulfonyl-7-methylbenzo[e][1,2]oxathiane-2,2-dioxide, reaction time: 1 d, reagents ratio 1 : 2 : 2.1 (373.3 mg; 1.34 mmol; 67%); white crystals; m.p. 128.5-129.0°C. ^{1}H NMR (400 MHz, $CDCl_3$) δ 8.19 (s, 1H), 7.55 (d, *J*=7.9 Hz, 1H), 7.29 (d, *J*=7.9 Hz, 1H), 7.21 (s, 1H), 2.51 (s, 3H). ^{13}C NMR (100 MHz, $CDCl_3$) δ 152.7, 149.9, 146.6 (d, *J*=1.1 Hz), 132.2, 128.5, 127.1 (d, *J*=32.4 Hz), 119.9, 115.3, 22.3. ^{19}F NMR (376 MHz, $CDCl_3$) δ 65.2. MS (EI) *m/z*: (%) 278 (100, [M^+]), 214 (12), 131 (25), 103 (19). HRMS (EI) *m/z*: Calc. for $C_9H_7O_5FS_2$ [M^+] 277.9719. Found 277.9716.

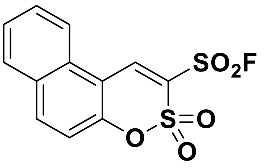

3b, 2-(fluorosulfonyl)naphtho[1,2-e][1,2]oxathiine-3,3-dioxide, reaction time 3 d, reagents ratio 1 : 2 : 1.9 (366.4 mg; 1.17 mmol; 58%), or reaction time: 1 d, reagents ratio 1 : 2 : 2.1 (136.2 mg; 0.43 mmol; 22%); yellow needles; m.p. 175.5-176.0°C. ^{1}H NMR (400 MHz, $CDCl_3$) δ 8.94 (s, 1H), 8.24 (d, *J*=9.0 Hz, 1H), 8.17 (d, *J*=8.3 Hz, 1H), 7.99 (d, *J*=8.2 Hz, 1H), 7.83 (ddd, *J*=8.5, 7.0, 1.3 Hz, 1H), 7.70 (ddd, *J*=8.1, 7.0, 1.1 Hz, 1H), 7.48 (d, *J*=9.0 Hz, 1H). ^{13}C NMR (100 MHz, $CDCl_3$) δ 153.7, 142.6 (d, *J*=1.0 Hz), 139.0, 131.2, 130.7, 130.2, 129.8, 127.8, 126.8 (d, *J*=32.4 Hz), 121.9, 117.5, 112.7. ^{19}F NMR (376 MHz, $CDCl_3$) δ 65.5. MS (EI) *m/z*: (%) 314 (100, [M^+]), 250 (18), 139 (47), 127 (12). HRMS (EI) *m/z*: Calc. for $C_{12}H_7O_5FS_2$ [M^+] 313.9719. Found 313.9715.

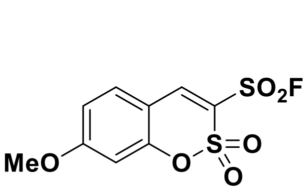

3c, 3-fluorosulfonyl-7-methoxybenzo[e][1,2]oxathiane-2,2-dioxide, reaction time: 3 d, reagents ratio 1 : 2 : 1.9 (377.1 mg; 1.28 mmol; 64%), or reaction time: 1 d, reagents ratio 1 : 2 : 2.1 (288.7 mg; 0.98 mmol; 48%); white solid; m.p. 128.5-129.5°C. ^{1}H NMR (400 MHz, $CDCl_3$) δ 8.15 (s, 1H), 7.57 (d, *J*=8.7 Hz, 1H), 6.98 (dd, *J*=8.7, 2.4 Hz, 1H), 6.87 (dd, *J*=2.4, 0.6 Hz, 1H), 3.95 (s, 3H). ^{13}C NMR (100 MHz, $CDCl_3$) δ 166.9, 155.0, 146.6, 134.0, 124.0 (d, *J*=32.4 Hz), 114.5, 110.9 (d, *J*=1.1 Hz), 104.7, 56.6. ^{19}F NMR (376 MHz, $CDCl_3$) δ 65.7. MS (EI) *m/z*: (%) 294 (100, [M^+]), 230 (24), 215 (20), 119 (4). HRMS (EI) *m/z*: Calc. for $C_9H_7O_6FS_2$ [M^+] 293.9668.

Found 293.9665. Crystals suitable for X-ray analysis were grown by slow evaporation of ethyl acetate : cyclohexane solution of **3c**.

Reaction with Salicylaldehyde

Reaction time: 7 d, reagents ratio 1 : 2.1 : 2.1, scale 5 mmol. Attempts at quantitative separation of the mixture (71% by weight; ca. 4 : 1, according to ^{1}H NMR) with column chromatography has failed, thus fractions enriched with selected products were crystallized by slow evaporation of cyclohexane : ethyl acetate solutions of **3d** and **3d'** to obtain:

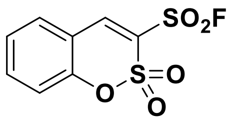

3d, 3-(fluorosulfonyl)benzo[e][1,2]oxathiane-2,2-dioxide (438.2 mg; 1.66 mmol at 5 mmol scale; 33%); colorless crystals; m.p. 95.5-97.0°C. ^{1}H NMR (400 MHz, $CDCl_3$) δ 8.24 (s, 1H), 7.76 (ddd, *J*=8.3, 7.5, 1.7 Hz, 1H), 7.70 (dd, *J*=7.8, 1.6 Hz, 1H), 7.50 (td, *J*=7.6, 1.1 Hz, 1H), 7.40 (ddt, *J*=8.4, 1.1, 0.5 Hz, 1H). ^{13}C NMR (100 MHz, $CDCl_3$) δ 152.6, 146.7 (d, *J*=1.1 Hz), 137.0, 132.5, 128.5 (d, *J*=32.6 Hz), 127.5, 119.5, 117.8 (d, *J*=1.1 Hz). ^{19}F NMR (376 MHz, $CDCl_3$) δ 65.1. MS (EI) *m/z*: (%) 264 (100, [M^+]), 200 (13), 181 (6), 133 (28), 108 (10), 105 (30), 89 (62). HRMS (EI) *m/z*: Calc. for $C_8H_5O_5FS_2$ [M^+] 263.9562. Found 263.9565.

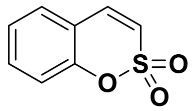

3d', benzo[e][1,2]oxathiane-2,2-dioxide (52.5 mg; 0.29 mmol at 5 mmol scale; 6%); colorless crystals; m.p. 85.0-85.5°C (lit. 82-83°C: A. Grandane, S. Belyakov, P. Trapencieris, R. Zalubovskis, *Tetrahedron* **2012**, *68*, 5541-5546). ^{1}H NMR (400 MHz, $CDCl_3$) δ 7.49 (ddd, *J*=8.2, 7.4, 1.7 Hz, 1H), 7.43 (dd, *J*=7.7, 1.7 Hz, 1H), 7.31 (td, *J*=7.6, 1.2 Hz, 1H), 7.29–7.23 (m, 2H), 6.78 (d, *J*=10.3 Hz, 1H). ^{13}C NMR (100 MHz, $CDCl_3$) δ 151.5, 135.9, 132.3, 129.4, 126.0, 122.2, 118.9, 118.9. The ^{1}H and ^{13}C NMR spectra were consistent with those described in the literature: T. Nishimura, Y. Takiguchi, T. Hayashi *J. Am. Chem. Soc.* **2012**, *134*, 9086-9089.

4.3. Reactions with Other Salicylaldehydes

Reactions with 3-methyl-2-hydroxybenzaldehyde and 5-methyl-2-hydroxybenzaldehyde gave mixtures of products, similar as with salicylaldehyde (see above).

5. Synthesis of Unsaturated 1,1-Disulfonyl Fluorides, 4

5.1. General procedure

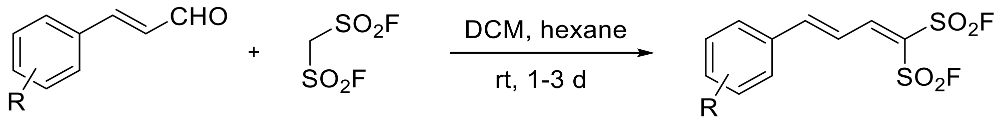

1 **4a-4o**

A 20 ml Schlenk flask was charged with aldehyde (5 mmol) and flushed with argon. Then, DCM (2 ml) and **1** (946 mg, 5.25 mmol) were added. After dissolution, hexane (10 ml) was layered on the top of the DCM phase. After 1-3 d at ambient temperature resulted crystalline products were filtered off, washed with DCM/cyclohexane mixture 1:5 (2×3ml) and dried. In selected cases combined liquid phases (aliquots) were left on air for slow, partial evaporation over 1 d, that resulted in a second crop of crystals, which was washed and dried in the same manner as the first one. In some cases, after 3 d of the reaction course diffusional mixing was not complete thus the flask content was carefully mixed and placed in a refrigerator to induce crystallization.

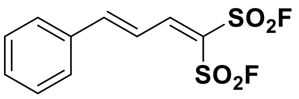

4a, (E)-(4,4-bis(fluorosulfonyl)buta-1,3-dienyl)benzene, reaction time: 3 d (1.258 g; 4.27 mmol; 85%, in two crops: 78 + 7%); yellow crystals; m.p. 124.5-125.5°C. ^{1}H NMR (400 MHz, $CDCl_3$) δ 8.26 (dd, *J*=11.8, 5.6 Hz, 1H), 7.76 (ddd, *J*=15.0, 11.8, 1.0 Hz, 1H), 7.70–7.65 (m, 2H), 7.60 (d, *J*=15.1 Hz, 1H), 7.58–7.53 (m, 1H), 7.52–7.46 (m, 2H). ^{13}C NMR (100 MHz, $CDCl_3$) δ 159.9, 158.7, 133.7, 133.6, 130.2, 129.6, 121.7 (t, *J*=29.9 Hz), 119.4 (d, *J*=1.5 Hz). ^{19}F NMR (376 MHz, $CDCl_3$) δ 70.3 (t, *J*=5.9 Hz), 63.3 (d, *J*=6.2 Hz). MS (EI) *m/z*: (%) 294 (45, [M^+]), 210 (22), 146 (21), 128 (100), 115 (46), 102 (25). HRMS (EI) *m/z*: Calc. for $C_{10}H_8O_4F_2S_2$ [M^+] 293.9832. Found 293.9828. Crystals suitable for X-ray analysis were grown by slow diffusional mixing of cyclohexane with toluene solution of **4a**.

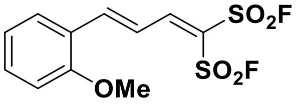

4b, (E)-(4,4-bis(fluorosulfonyl)buta-1,3-dienyl)-2-methoxybenze-ne, reaction time: 1 d (1.551 g; 4.78 mmol; 96%, in two crops: 79 + 17%); brown-orange needles; m.p. 137°C (dec). ^{1}H NMR (400 MHz, $CDCl_3$) δ 8.24 (dd, *J*=11.7, 5.5 Hz, 1H), 7.95 (d, *J*=15.1 Hz, 1H), 7.85 (ddd, *J*=15.1, 11.7, 0.9 Hz, 1H), 7.64 (dd,

J=7.8, 1.7 Hz, 1H), 7.52 (ddd, J=8.4, 7.4, 1.7 Hz, 1H), 7.07–7.01 (m, 1H), 6.97 (dd, J=8.5, 1.0 Hz, 1H), 3.94 (s, 3H). ^{13}C NMR (100 MHz, $CDCl_3$) δ 160.1, 159.9, 155.9, 135.7, 130.8, 122.8, 121.3, 119.9 (d, J=1.5 Hz), 119.8 (t, J=29.4 Hz), 111.7, 55.8. ^{19}F NMR (376 MHz, $CDCl_3$) δ 70.2 (t, J=5.9 Hz), 63.4 (d, J=6.2 Hz). MS (EI) *m/z*: (%) 324 (96, [M^+]), 240 (34), 158 (100), 142 (30), 127 (41), 115 (38), 91 (23). HRMS (EI) *m/z*: Calc. for $C_{11}H_{10}O_5F_2S_2$ [M^+] 323.9938. Found 323.9925.

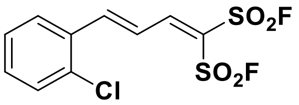

4c, (E)-(4,4-bis(fluorosulfonyl)buta-1,3-dienyl)-2-chlorobenzene, reaction time: 1 d, the product did not crystalize, so the mixture was mixed and placed in refrigerator for 3 h, second crop was obtained by slow evaporation of the solvent (1.589 g; 4.83 mmol; 97%, in two crops: 52 + 45%); yellow crystals; m.p. 131.0-132.5°C. ^{1}H NMR (400 MHz, $CDCl_3$) δ 8.30 (ddd, J=11.8, 5.7, 0.6 Hz, 1H), 8.06 (d, J=15.2 Hz, 1H), 7.84–7.71 (m, 2H), 7.52–7.43 (m, 2H), 7.38 (ddd, J=8.7, 7.0, 1.8 Hz, 1H). ^{13}C NMR (100 MHz, $CDCl_3$) δ 158.2, 154.3, 136.9, 134.1, 131.6, 130.8, 128.9, 127.7, 123.3 (t, J=30.1 Hz), 121.2 (d, J=1.5 Hz). ^{19}F NMR (376 MHz, $CDCl_3$) δ 70.4 (t, J=6.0 Hz), 63.2 (d, J=6.2 Hz). MS (EI) *m/z*: (%) 328 (69, [M^+]), 244 (30), 180 (19), 162 (100), 149 (39), 126 (35). HRMS (EI) *m/z*: Calc. for $C_{10}H_7O_4F_2S_2Cl$ [M^+] 327.9442. Found 327.9436.

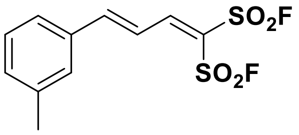

4d, (E)-(4,4-bis(fluorosulfonyl)buta-1,3-dienyl)-3-methylbenzene, reaction time: 1 d, the product did not crystalize, so the mixture was mixed and placed in refrigerator for 3 h, second crop was obtained by slow evaporation of the solvent (1.364 g; 4.42 mmol; 88%, in two crops: 61 + 27%); yellow needles; m.p. 117.5-120.0°C. ^{1}H NMR (400 MHz, $CDCl_3$) δ 8.25 (dd, J=11.8, 5.6 Hz, 1H), 7.73 (dd, J=15.0, 11.8 Hz, 1H), 7.57 (d, J=15.0 Hz, 1H), 7.50–7.45 (m, 2H), 7.42–7.34 (m, 2H), 2.41 (s, 3H). ^{13}C NMR (100 MHz, $CDCl_3$) δ 160.4, 158.9, 139.5, 134.8, 133.6, 130.6, 129.4, 127.7, 121.2 (t, J=29.9 Hz), 119.2 (d, J=1.5 Hz), 21.2. ^{19}F NMR (376 MHz, $CDCl_3$) δ 70.3 (t, J=5.9 Hz), 63.3 (d, J=6.1 Hz). MS (EI) *m/z*: (%) 308 (34, [M^+]), 224 (13), 142 (100), 115 (56). HRMS (EI) *m/z*: Calc. for $C_{11}H_{10}O_4F_2S_2$ [M^+] 307.9989. Found 307.9985.

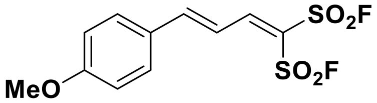

4e, (E)-(4,4-bis(fluorosulfonyl)buta-1,3-dienyl)-4-methoxy-benzene, reaction time: 1 d (1.613 g; 4.97 mmol; 99%, in two crops: 87 + 12%); red needles; m.p. 164°C (dec). ^{1}H NMR (400 MHz, $CDCl_3$) δ 8.20 (ddd,

J=10.6, 5.5, 0.9 Hz, 1H), 7.68–7.62 (m, 2H), 7.61–7.51 (m, 2H), 7.02–6.95 (m, 2H), 3.89 (s, 3H). ^{13}C NMR (100 MHz, $CDCl_3$) δ 164.7, 160.3, 159.0, 132.9, 126.7, 118.7 (t, J=29.6 Hz), 117.2 (d, J=1.5 Hz), 115.3, 55.8. ^{19}F NMR (376 MHz, $CDCl_3$) δ 70.2 (t, J=5.9 Hz), 63.6 (d, J=6.3 Hz). MS (EI) *m/z*: (%) 324 (77, [M^+]), 240 (27), 158 (100), 115 (38). HRMS (EI) *m/z*: Calc. for $C_{11}H_{10}O_5F_2S_2$ [M^+] 323.9938. Found 323.9922.

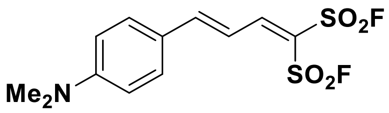

4f, (E)-N,N-dimethyl-4-(4,4-bis(fluorosulfonyl)buta-1,3-dienyl)aniline, reaction time: 3 d, 3 ml of DCM was used as a reaction solvent (1.656 g; 4.91 mmol; 98%); dark-violet solid; m.p. 150-180°C (dec). ^{1}H NMR (400 MHz, acetone-d_6) δ 8.30 (dd, J=12.7, 3.5 Hz, 1H), 8.05 (dd, J=14.0, 2.9 Hz, 1H), 7.78 (d, J=8.6 Hz, 2H), 7.44 (t, J=13.4 Hz, 1H), 6.95 (d, J=9.0 Hz, 2H), 3.27 (s, 6H). ^{13}C NMR (100 MHz, acetone-d_6) δ 165.4, 159.7, 156.9, 136.3, 123.5, 114.3, 113.9, 40.7. ^{19}F NMR (376 MHz, acetone-d_6) δ 70.3, 65.5. MS (EI) *m/z*: (%) 337 (100, [M^+]), 252 (37), 171 (81), 128 (10). HRMS (EI) *m/z*: Calc. for $C_{12}H_{13}NO_4F_2S_2$ [M^+] 337.0254. Found 337.0242.

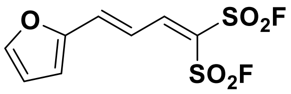

4g, (E)-2-(4,4-bis(fluorosulfonyl)buta-1,3-dien-1-yl)furan, reaction time: 1 d (1.395 g; 4.91 mmol; 98%, in two crops: 78 + 20%); red needles; m.p. 94°C (dec). ^{1}H NMR (400 MHz, $CDCl_3$) δ 8.15 (dd, J=12.3, 5.3 Hz, 1H), 7.73 (d, J=1.7 Hz, 1H), 7.58–7.48 (m, 1H), 7.33 (d, J=14.6 Hz, 1H), 7.07 (d, J=3.6 Hz, 1H), 6.66 (dd, J=3.7, 1.8 Hz, 1H). ^{13}C NMR (100 MHz, $CDCl_3$) δ 158.1, 150.9, 149.6, 143.2, 123.2, 119.4 (t, J=29.6 Hz), 117.0 (d, J=1.5 Hz), 114.6. ^{19}F NMR (376 MHz, $CDCl_3$) δ 70.3 (t, J=5.8 Hz), 63.5 (d, J=6.1 Hz). MS (EI) *m/z*: (%) 284 (100, [M^+]), 200 (42), 118 (74), 89 (14). HRMS (EI) *m/z*: Calc. for $C_8H_6O_5F_2S_2$ [M^+] 283.9625. Found 283.9624.

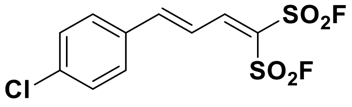

4h, (E)-(4,4-bis(fluorosulfonyl)buta-1,3-dienyl)-4-chlorobenzene, reaction time: 1 d, the product did not crystalize, so the mixture was mixed and placed in refrigerator for 3 h; second crop was obtained by slow evaporation of the solvent (1.463 g; 4.45 mmol; 89% in two crops: 57 + 32%); yellow crystals; m.p. 113.0-114.5°C. ^{1}H NMR (400 MHz, $CDCl_3$) δ 8.24 (dd, J=11.7, 5.7 Hz, 1H), 7.72 (ddd, J=15.1, 11.8, 1.0 Hz, 1H), 7.63–7.58 (m, 2H), 7.54 (d, J=15.1 Hz, 1H), 7.48–7.43 (m, 2H). ^{13}C NMR (100 MHz, $CDCl_3$) δ 158.2, 157.8, 140.1, 132.0, 131.1, 130.0, 122.4 (t, J=29.9 Hz), 119.8

(d, J=1.5 Hz). ^{19}F NMR (376 MHz, $CDCl_3$) δ 70.4 (t, J=5.9 Hz), 63.3 (d, J=6.2 Hz). MS (EI) *m/z*: (%) 328 (73, [M^+]), 244 (29), 180 (14), 162 (100), 149 (30), 126 (27). HRMS (EI) *m/z*: Calc. for $C_{10}H_7O_4F_2S_2Cl$ [M^+] 327.9442. Found 327.9445.

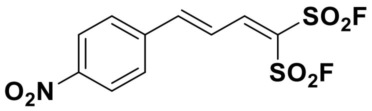

4i, (E)-(4,4-bis(fluorosulfonyl)buta-1,3-dienyl)-4-nitrobenzene, reaction time: 1 d, 4 ml of DCM was used as a reaction solvent, DCM was added to obtain homogenous mixture and product crystalised by slow evaporation of the solvent (1.657 g; 4.88 mmol; 97%, in two crops: 95 + 2%); brown crystals; m.p. 142°C (dec). ^{1}H NMR (400 MHz, $CDCl_3$) δ 8.34–8.30 (m, 2H), 8.28 (ddd, J=11.6, 5.8, 0.6 Hz, 1H), 7.90–7.80 (m, 3H), 7.63 (d, J=15.3 Hz, 1H). ^{13}C NMR (100 MHz, $CDCl_3$) δ 156.9, 154.8, 149.9, 139.0, 130.3, 125.4 (t, J=30.4 Hz), 124.6, 122.7 (d, J=1.4 Hz). ^{19}F NMR (376 MHz, $CDCl_3$) δ 70.5 (t, J=6.0 Hz), 63.2 (d, J=6.2 Hz). MS (EI) *m/z*: (%) 339 (100, [M^+]), 255 (17), 225 (25), 210 (54), 173 (33), 126 (35), 115 (56). HRMS (EI) *m/z*: Calc. for $C_{10}H_7NO_6F_2S_2$ [M^+] 338.9683. Found 338.9696.

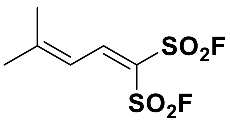

4j, 1,1-bis(fluorosulfonyl)-4-methylpenta-1,3-diene, reaction time: 3 d, 1 ml of DCM was used as a reaction solvent; the product did not crystalize, so the mixture was placed in refrigerator for 3 h; second crop was obtained by slow evaporation of the solvent (1.014 g; 4.12 mmol at 5.5 mmol scale; 75%); brown crystals; m.p. 80.0-80.5°C. ^{1}H NMR (400 MHz, $CDCl_3$) δ 8.41 (dd, J=12.6, 5.5 Hz, 1H), 6.99 (ddq, J=12.6, 2.5, 1.3 Hz, 1H), 2.22 (s, 3H), 2.19 (d, J=1.3 Hz, 3H). ^{13}C NMR (100 MHz, $CDCl_3$) δ 173.8, 153.4, 120.5 (t, J=29.6 Hz), 119.6 (d, J=1.4 Hz), 29.0, 20.7. ^{19}F NMR (376 MHz, $CDCl_3$) δ 70.05 (t, J=5.8 Hz), 62.58 (d, J=6.1 Hz). MS (EI) *m/z*: (%) 246 (19, [M^+]), 162 (8), 79 (100), 67 (14). HRMS (EI) *m/z*: Calc. for $C_6H_8O_4F_2S_2$ [M^+] 245.9832. Found 245.9830.

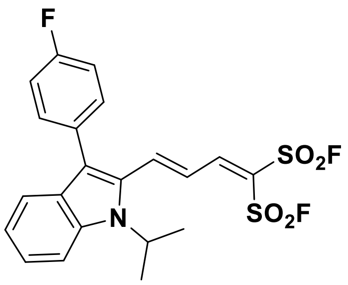

4k, (E)-2-(4,4-bis(fluorosulfonyl)buta-1,3-dienyl)-1-isopropyl-3-(4-fluorophenyl)indole, reaction time: 3 d (1.570 g; 3.34 mmol at 3.4 mmol scale; 98%); red needles; m.p. 123°C (dec). ^{1}H NMR (400 MHz, $CDCl_3$) δ 8.07 (dd, J=12.2, 5.3 Hz, 1H), 7.67–7.58 (m, 2H), 7.51 (d, J=8.1 Hz, 1H), 7.43–7.29 (m, 4H), 7.27–7.20 (m, 2H), 7.14 (ddd, J=8.0, 6.9, 0.8 Hz, 1H), 5.00 (hept, J=7.0 Hz, 1H), 1.76 (d, J=7.0 Hz, 6H). ^{13}C NMR (100 MHz, $CDCl_3$) δ 162.9 (d, J=248.7 Hz), 158.0, 146.6,

139.7, 132.0 (d, *J*=8.2 Hz), 131.5, 129.9, 128.9 (d, *J*=3.5 Hz), 128.5, 127.6, 122.3, 121.6, 119.4, 117.6 (t, *J*=29.5 Hz), 116.4 (d, *J*=21.7 Hz), 112.9, 48.9, 21.8. ^{19}F NMR (376 MHz, $CDCl_3$) δ 70.2 (t, *J*=5.8 Hz), 63.9 (d, *J*=6.2 Hz), -113.47 (tt, *J*=8.7, 5.4 Hz). MS (EI) *m/z*: (%) 469 (100, [M^+]), 427 (18), 343 (11), 264 (25), 261 (33), 236 (5). HRMS (EI) *m/z*: Calc. for $C_{21}H_{18}NO_4F_3S_2$ [M^+] 469.0629. Found 469.0629.

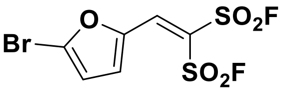

4l, 2-bromo-5-[2,2-bis(fluorosulfonyl)vinyl]-furane, reaction time: 5 d, the product did not crystalize, so the mixture was evaporated, dissolved in toluene/heptane and crystallized by slow evaporation of the solvent (0.692 g; 2.05 mmol at 2.8 mmol scale; 73%, in two crops: 51 + 22%); slightly yellowish crystals; m.p. 121.5-123.5°C. ^{1}H NMR (400 MHz, $CDCl_3$) δ 8.18 (d, *J*=3.4 Hz, 1H), 7.75 (d, *J*=4.0 Hz, 1H), 6.81 (dd, *J*=4.0, 0.5 Hz, 1H). ^{13}C NMR (100 MHz, $CDCl_3$) δ 146.7 (d, *J*=2.4 Hz), 138.4, 138.1, 133.3, 118.8, 118.0 (t, *J*=32.2 Hz). ^{19}F NMR (376 MHz, $CDCl_3$) δ 66.8, 62.1 (d, *J*=5.8 Hz). MS (EI) *m/z*: (%) 336 (62, [M^+]), 254 (100), 145 (50), 63 (53). HRMS (EI) *m/z*: Calc. for $C_6H_3O_5F_2S_2Br$ [M^+] 335.8573. Found 335.8574.

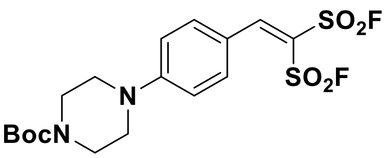

4m, *tert*-butyl 4-{4-[2,2-bis(fluorosulfonyl)vinyl] phenyl}-piperazine-1-carboxylate, reaction time: 5 d, the product did not crystalize, so the mixture was evaporated, dissolved in toluene, filtrated and crystallized from toluene/cyclohexane (1 d in refrigerator) (1.252 g; 2.77 mmol at 3.7 mmol scale; 75% in two crops: 72 + 3%); brown solid; m.p. 138-158°C decomposition. ^{1}H NMR (400 MHz, $CDCl_3$) δ 8.17 (d, *J*=4.4 Hz, 1H), 7.80 (d, *J*=9.3 Hz, 2H), 6.84 (d, *J*=9.3 Hz, 2H), 3.60 (s, 8H), 1.47 (s, 9H). ^{13}C NMR (100 MHz, $CDCl_3$) δ 155.9, 155.4, 154.4, 139.3 (d, *J*=1.3 Hz), 116.6 (d, *J*=2.1 Hz), 113.9 (t, *J*=29.8 Hz), 112.8, 80.7, 46.1, 28.3. ^{19}F NMR (376 MHz, $CDCl_3$) δ 67.7 (dd, *J*=6.7, 4.6 Hz), 61.8 (d, *J*=6.8 Hz). MS (EI) *m/z*: (%) 452 (10, [M^+]), 396 (31), 370 (9), 314 (25), 240 (13), 64 (69), 57 (75), 41 (100). HRMS (EI) *m/z*: Calc. for $C_{17}H_{22}N_2O_6F_2S_2$ [M^+] 452.0887. Found 452.0874.

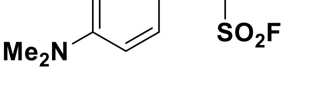

4n, 4-[2,2-bis(fluorosulfonyl)vinyl]-*N,N*-dimethylaniline, react-ion time: 2 d (1.470 g; 4.73 mmol; 94%), or (601.1 mg; 1.93 mmol; 39% under olefination conditions, at rt); orange solid; m.p. 184°C

(dec) (lit. 183-184°C: I. I. Maletina, A. A. Mironova, T. I. Savina, Y. L. Yagupolskii, *Zh. Org. Khim.* **1979**, *15*, 2416-2417). ^{1}H NMR (400 MHz, $CDCl_3$) δ 8.16 (d, *J*=4.6 Hz, 1H), 7.80 (d, *J*=9.3 Hz, 2H), 6.72 (d, *J*=9.4 Hz, 2H), 3.21 (s, 6H). ^{13}C NMR (100 MHz, $CDCl_3$) δ 156.0, 155.8, 139.4, 115.6 (d, *J*=2.3 Hz), 112.1, 111.7 (t, *J*=30.4 Hz), 40.3. ^{19}F NMR (376 MHz, $CDCl_3$) δ 67.7 (dd, *J*=6.9, 4.5 Hz), 62.0 (d, *J*=7.0 Hz). MS (EI) *m/z*: (%) 311 (100, [M^+]), 227 (33), 145 (32). HRMS (EI) *m/z*: Calc. for $C_{10}H_{11}NO_4F_2S_2$ [M^+] 311.0098. Found 311.0092.

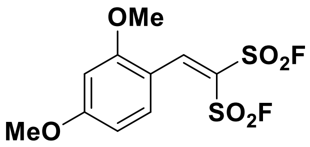

4o, 1,2-dimethoxy-4-[2,2-bis(fluorosulfonyl)vinyl]-benzene, reaction time: 7 d, DCM was added to obtain homogenous mixture and product crystalised by slow evaporation of the solvent (694.3 mg; 2.11 mmol; 42%); yellow crystals; m.p. 112.5-113.5°C. ^{1}H NMR (400 MHz, $CDCl_3$) δ 8.92 (d, *J*=5.6 Hz, 1H), 7.90 (d, *J*=9.1 Hz, 1H), 6.63 (dd, *J*=9.1, 2.4 Hz, 1H), 6.44 (d, *J*=2.4 Hz, 1H), 3.93 (s, 3H), 3.92 (s, 3H). ^{13}C NMR (100 MHz, $CDCl_3$) δ 168.9, 163.6, 152.5, 135.7 (d, *J*=3.4 Hz), 119.8 (dd, *J*=31.1, 29.3 Hz), 110.7 (d, *J*=2.0 Hz), 107.5, 97.7, 56.3, 56.1. ^{19}F NMR (376 MHz, $CDCl_3$) δ 67.5 (dd, *J*=8.6, 5.6 Hz), 61.4 (d, *J*=8.7 Hz). MS (EI) *m/z*: (%) 328 (70, [M^+]), 244 (100), 161 (52), 148 (50), 119 (25), 76 (21). HRMS (EI) *m/z*: Calc. for $C_{10}H_{10}O_6F_2S_2$ [M^+] 327.9887. Found 327.9889. Crystals suitable for X-ray analysis were grown by slow cooling of toluene : cyclohexane solution of **4o**.

5.2. Condensations of Electron-rich Benzaldehydes Under Various Conditions

Aldehyde	Olefination conditions (65°C)	Olefination conditions (rt)	Condensation conditions
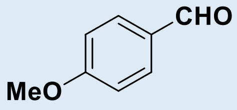	55 % of **2o**	not tested	0% (11%)[a]
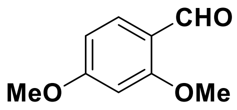	decomposition (traces of **2p**)[b]	25% of **2v** + 200 mg of unidentified product	42% of **4o**
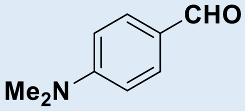	decomposition (traces of **4n**)[b]	1% of the olefination product, and 39% of **4n**	96% of **4n**

[a] – ^{1}H NMR yield of the condensation product; [b] – **4n** was detected with ^{1}H, ^{19}F NMR spectra

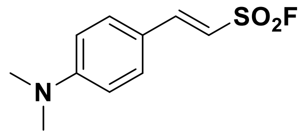

(E)-2-(4-(dimethylamine)phenyl)ethenesulfonyl fluoride, was isolated from olefination reaction carried out for 1 d at rt (15.6 mg; 0.07 mmol; 1%), **4n** (501 mg, 1.93 mmol, 39%) was also formed; yellow solid; m.p. 159-165°C (dec.). ^{1}H NMR (400 MHz, $CDCl_3$) δ 7.67 (d, *J*=15.2 Hz, 1H), 7.42–7.36 (m, 2H), 6.68–6.63 (m, 2H), 6.50 (dd, *J*=15.2, 2.6 Hz, 1H), 3.05 (s, 6H). ^{13}C NMR (100 MHz, $CDCl_3$) δ 153.1, 149.5 (d, *J* = 2.2 Hz), 131.2, 118.3, 111.7, 109.9 (d, *J*=26.4 Hz), 40.0. ^{19}F NMR (376 MHz, $CDCl_3$) δ 63.8 (d, *J*=2.6 Hz). MS (EI) *m/z*: (%) 229 (100, [M^+]), 145 (11). HRMS (EI) *m/z*: Calc. for $C_{10}H_{12}NO_2FS$ [M^+] 229.0573. Found 229.0571.

5.3. ^{1}H NMR Spectrum of *p*-Anisaldehyde Condensation

p-Anisaldehyde under condensation conditions with **1** gave ca. 10% of conversion (^{1}H NMR).

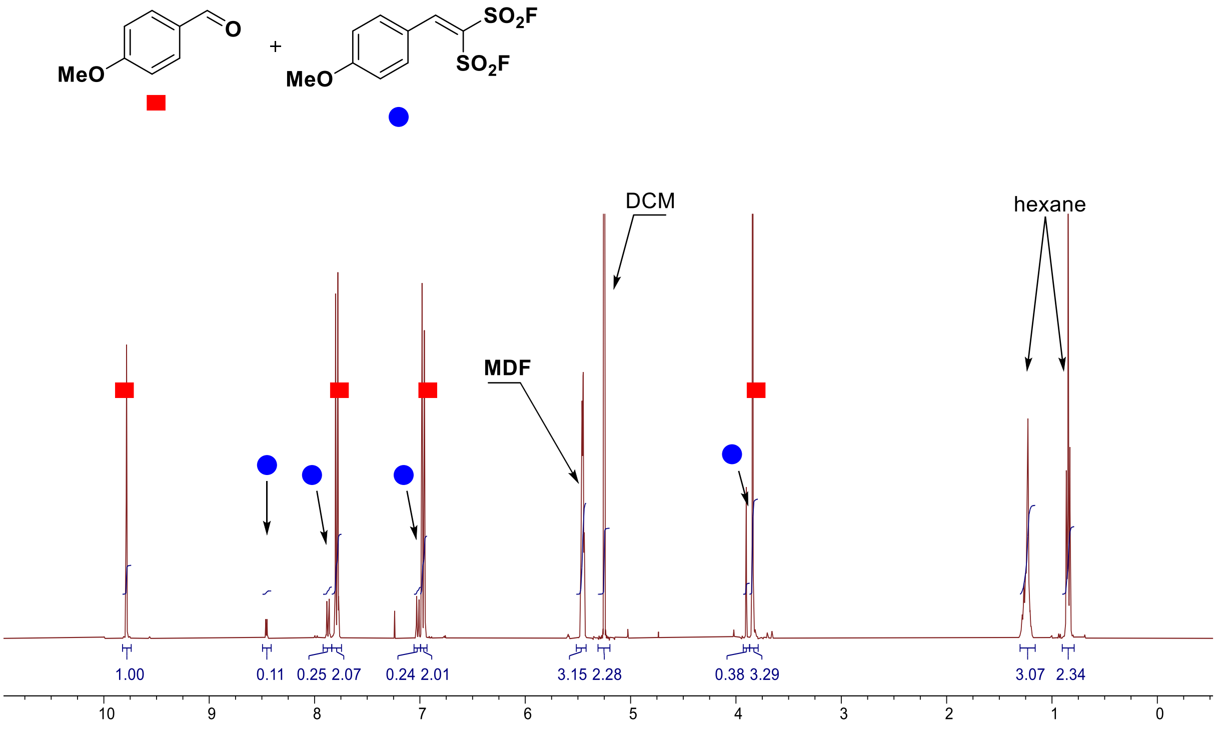

5.4. NMR Assignments of Diene 4j

For diene **4j** we assigned ^{1}H, ^{13}C and ^{19}F all peak resonances at NMR spectra, using correlation experiments (COSY, HSQC, HMBC, 1D NOESY).

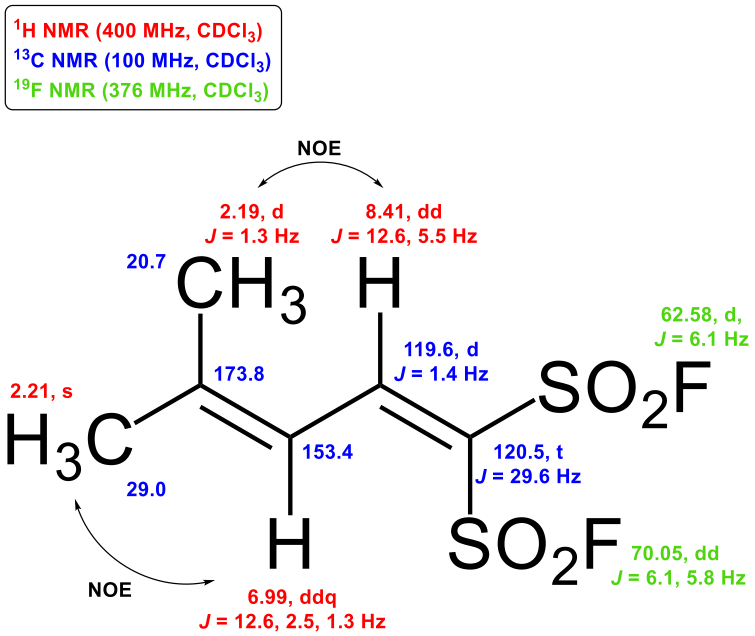

COSY experiment:

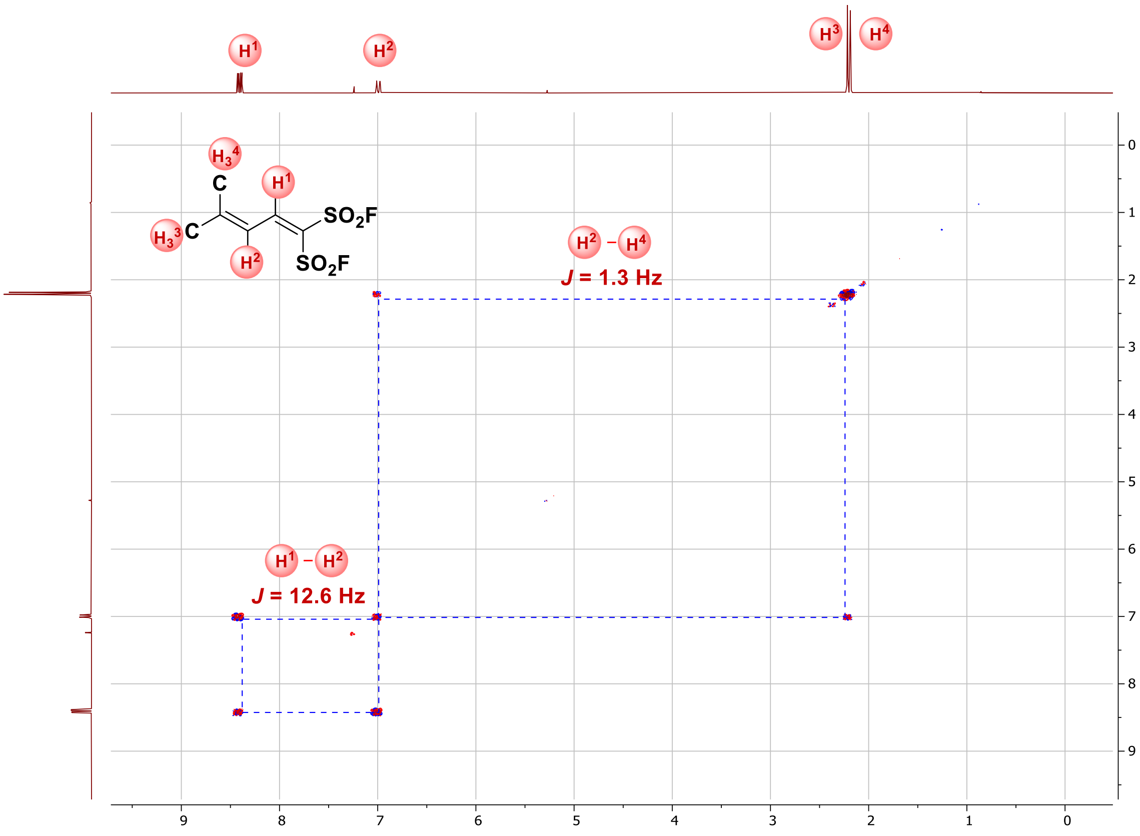

HSQC experiment

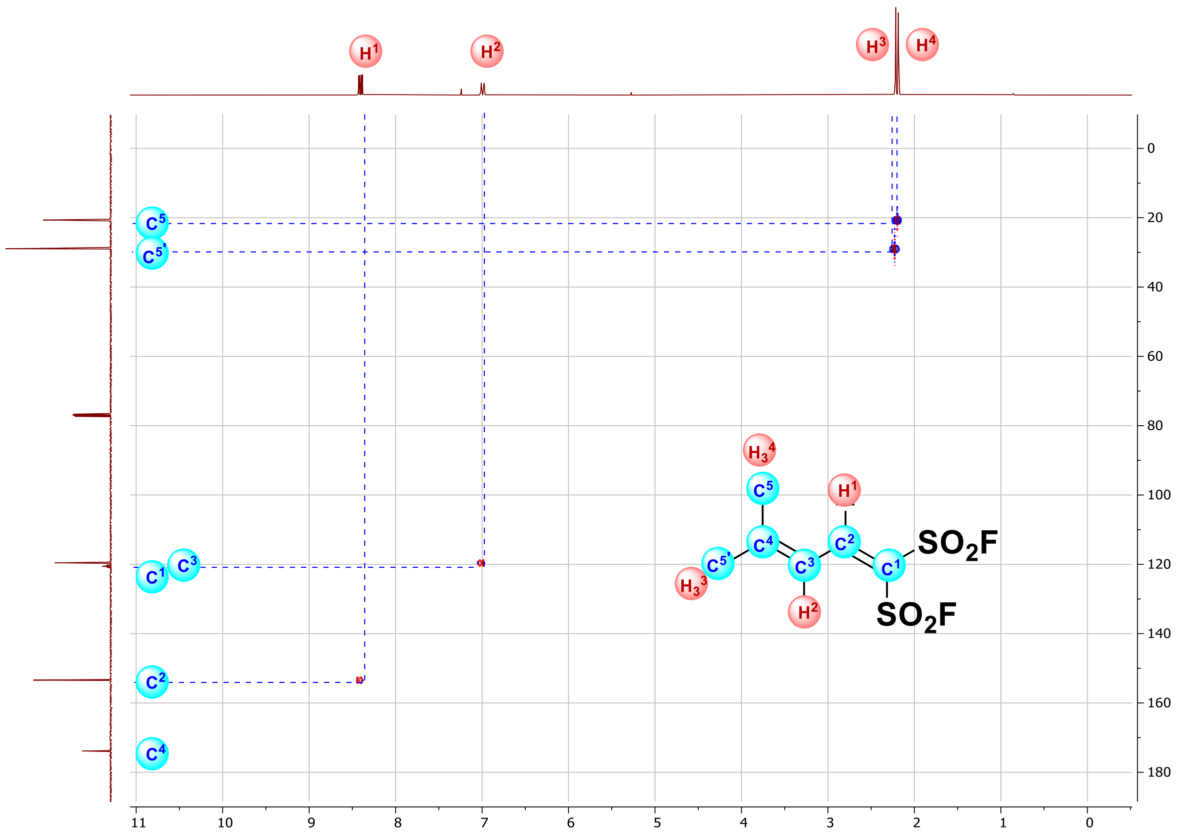

HMBC experiment:

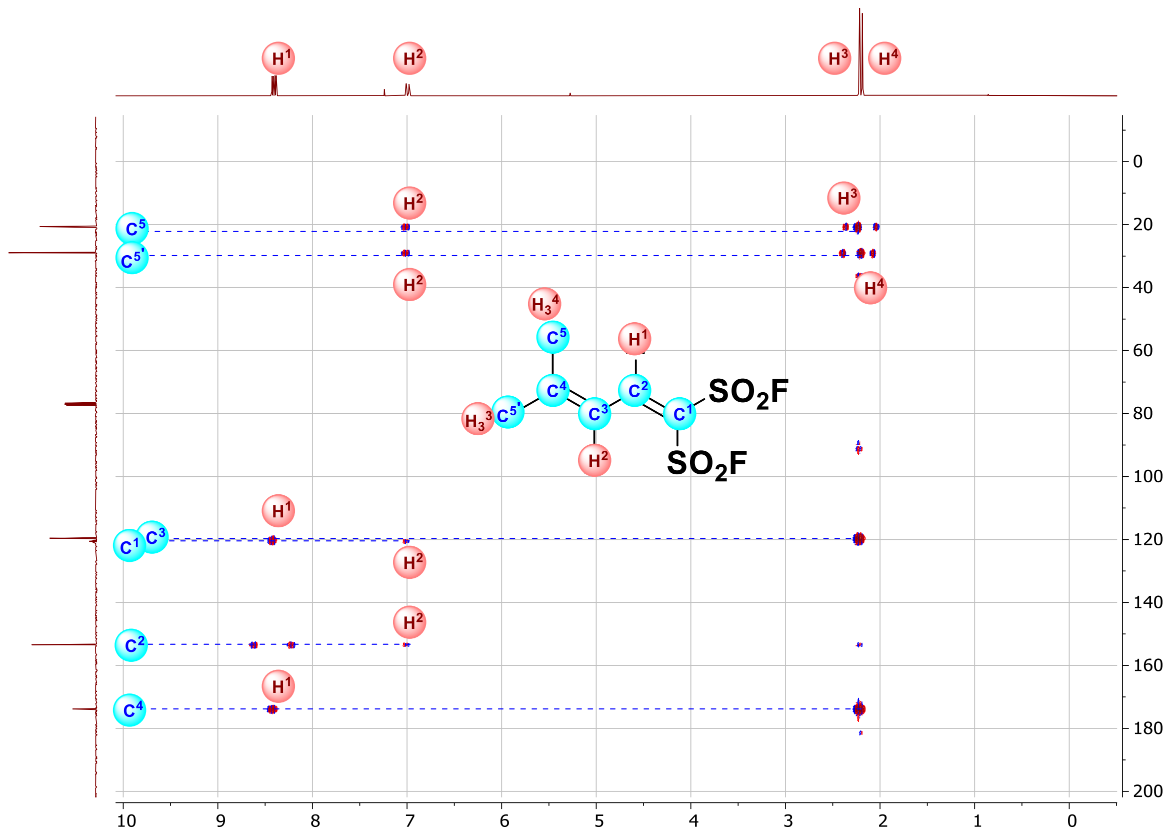

1D NOE experiment:

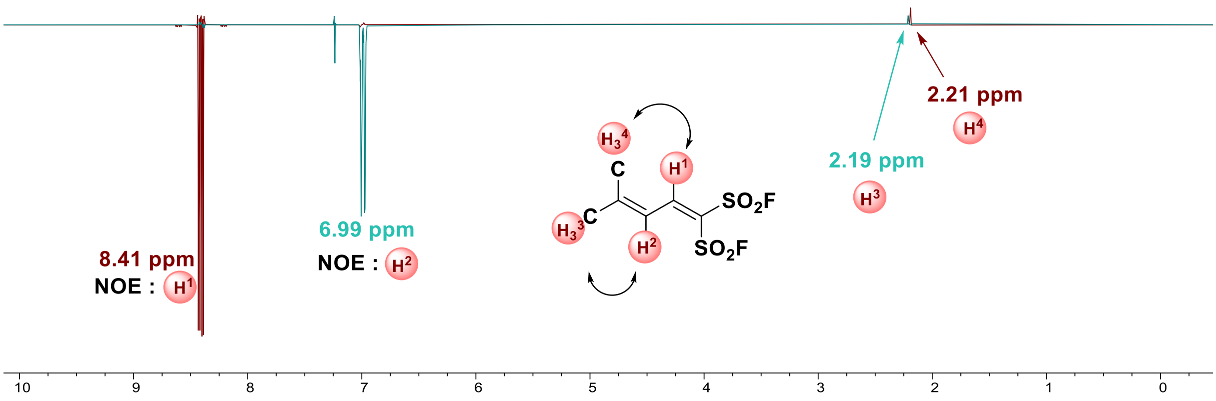

^{19}**F NMR** spectrum

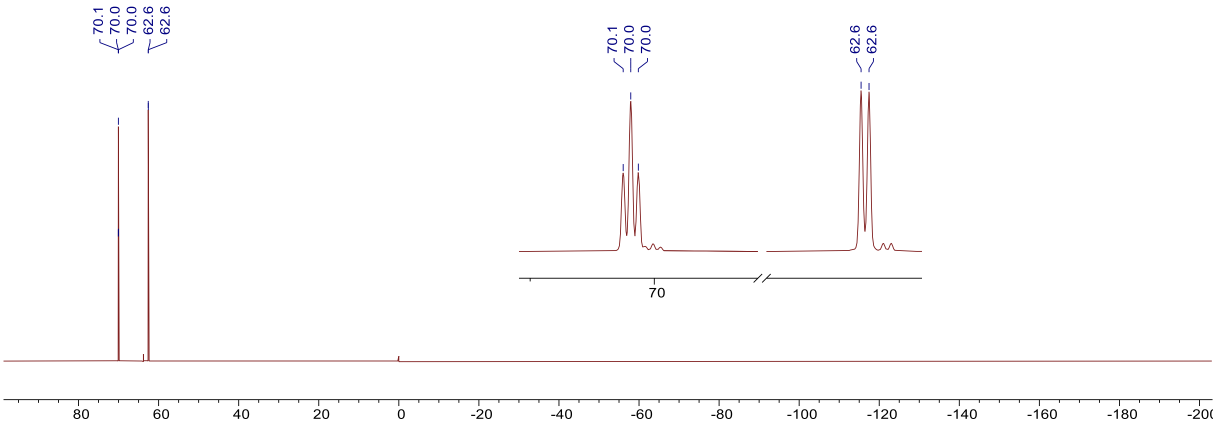

6. Reactions of 1 with Paraformaldehyde and Pyridines

Reactions of **1,** paraformaldehyde and different pyridines were carried out to obtain zwitterionic adducts. However, only reaction DMAP (4-(dimethylamino)pyridine) gave desired product **5,** as a white precipitate in a pure form.

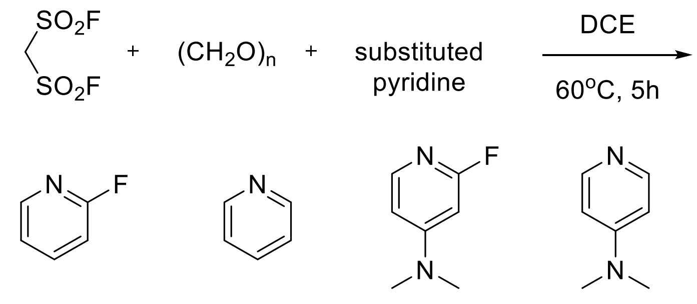

1H NMR spectra of crude reaction precipitates are shown below

complex mixture | *complex mixture* | *complex mixture* | **5** (72%)

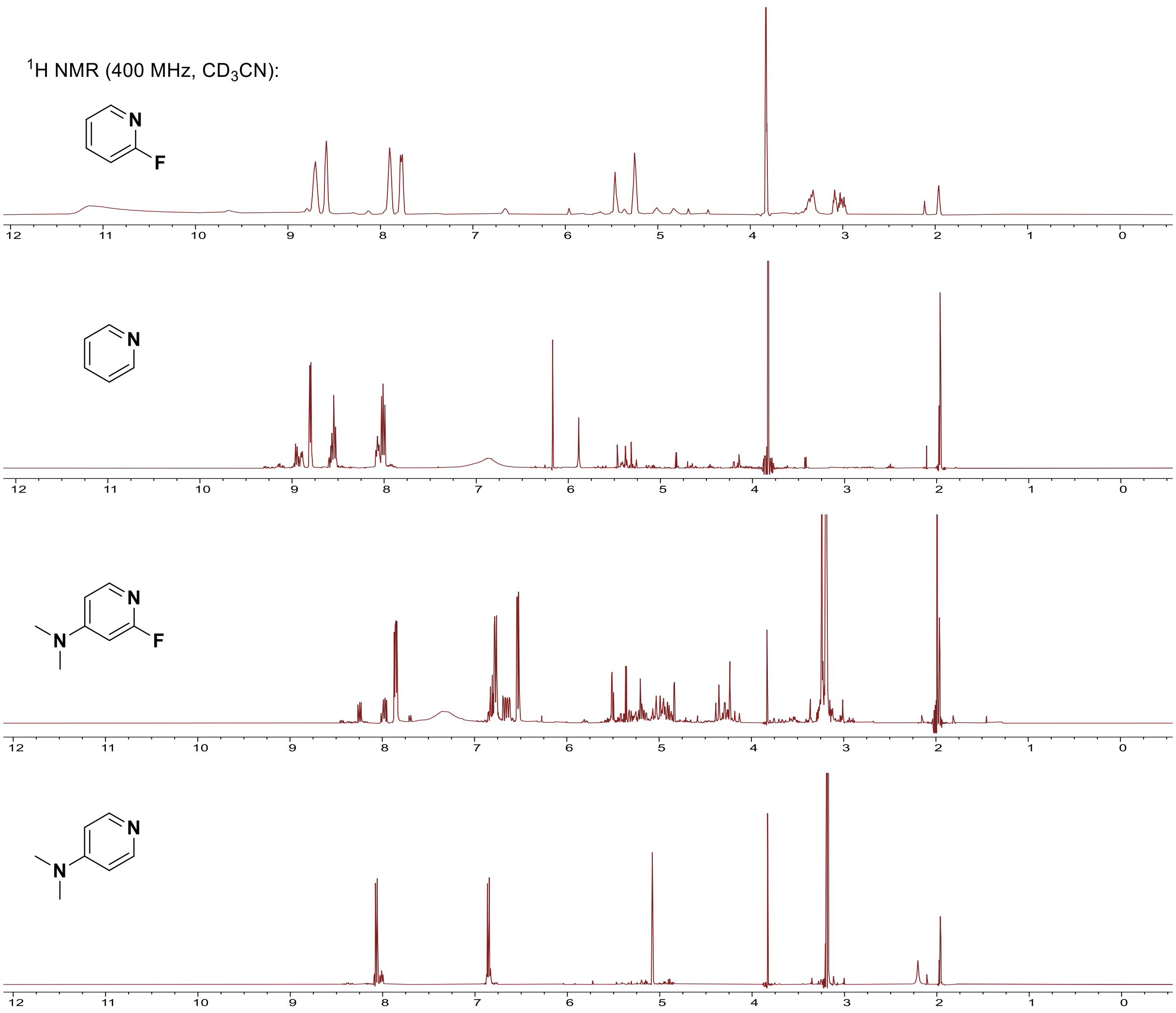

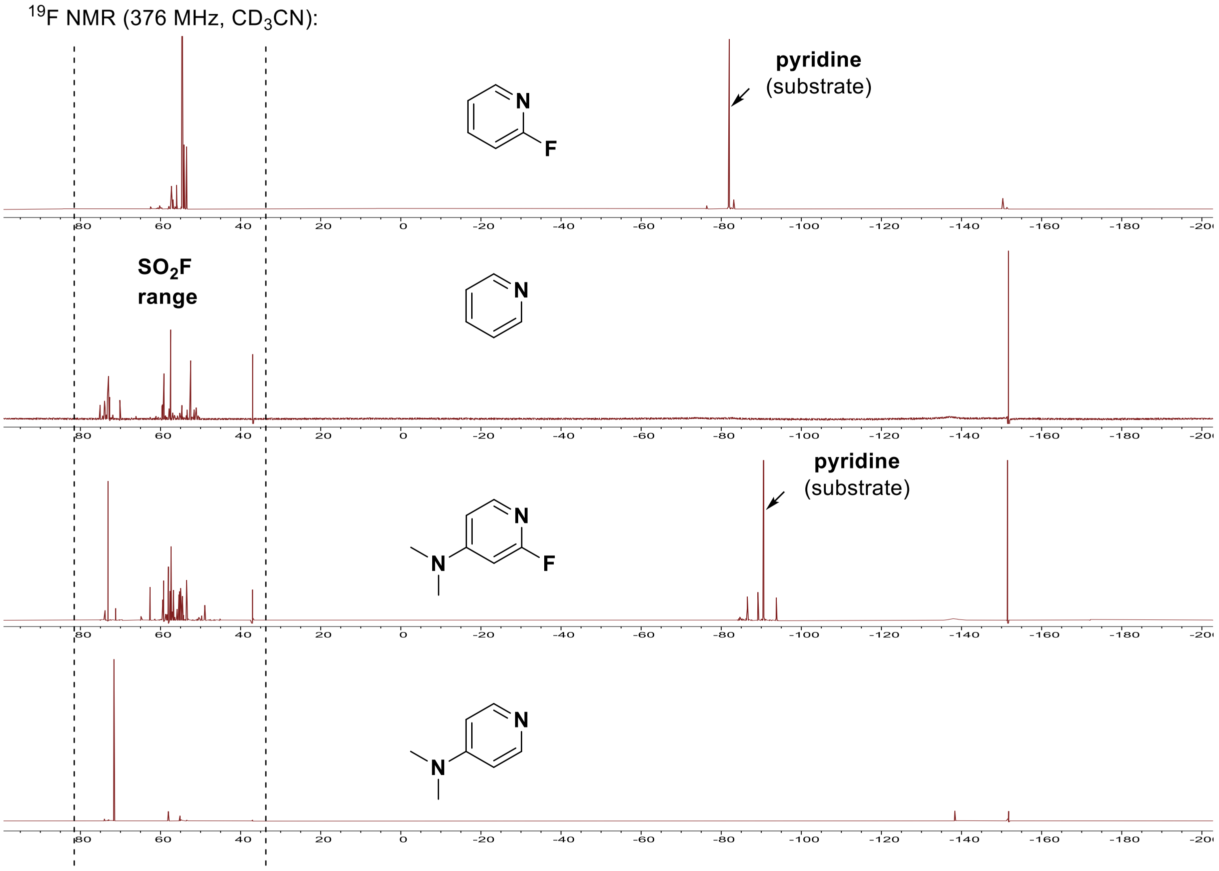

With less nucleophilic pyridines (2-fluoropyridine, pyridine, 4-(dimethylamino)-2-fluoropyridine), we observed complex mixtures of products (^{1}H and ^{19}F NMR).

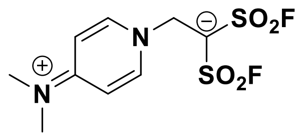

A 25 ml round-bottomed flask was charged with paraformaldehyde (210 mg, 7 mmol), DMAP (611 mg, 5 mmol) and flushed with argon. Next DCE (8 ml) and **1** (901 mg, 5 mmol) were added and the mixture was placed in oil bath 60 °C. After 5 h the mixture was cooled to rt, precipitate was filtered off, washed with DCM (3×1 ml) and dissolved in acetonitrile (10 ml). Unsolvable residue was filtered off and the acetonitrile solution was evaporated, and residue was dried *in vacuo* to give **2-(4-(dimethylamino)pyridin-1-ium-1-yl)-1,1-bis(fluorosulfonyl)ethan-1-ide** (**5**; 1.275 g; 4.06 mmol; 81%); white crystals; m.p. 170°C (dec). ^{1}H NMR (400 MHz, CD_3CN) δ 8.12–8.01 (m, 2H), 6.88–6.82 (m, 2H), 5.08 (t, *J*=2.4 Hz, 2H), 3.18 (s, 6H). ^{13}C NMR (100 MHz, CD_3CN) δ 157.6, 141.6, 108.4, 67.5 (t, *J*=24.2 Hz), 58.5, 40.5. ^{19}F NMR (376 MHz, CD_3CN) δ 71.6 (t, *J*=2.4 Hz). HRMS (ESI) *m/z*: Calc. for $C_9H_{13}N_2O_4F_2S_2$ $[M+H]^+$ 315.0285. Found 315.0287. Crystals suitable for X-ray analysis were grown by slow evaporation of acetonitrile : heptane solution of **5**.

4-(Dimethylamino)-2-fluoropyridine was synthesized according to the procedure described in: M. Schlosser, C. Bobbio, T. Rausis, *J. Org. Chem.* **2005**, *70*, 2494–2502. Yield: 90%. White crystals; m.p. 55.2-56.3°C. ^{1}H NMR (400 MHz, acetone-d_6) δ 7.77 (d, *J*=6.1 Hz, 1H), 6.52 (dt, *J*=6.1, 2.0 Hz, 1H), 6.10 (dd, *J*=2.1, 1.1 Hz, 1H), 3.03 (s, 6H). ^{13}C NMR (100 MHz, acetone-d_6) δ 165.8 (d, *J*=227 Hz), 158.5 (d, *J*=12.1 Hz), 146.9 (d, *J*=20.2 Hz), 105.4 (d, *J*=2.5 Hz), 89.2 (d, *J*=44.3 Hz), 38.6. ^{19}F NMR (376 MHz, acetone-d_6) δ -71.8.

7. Synthesis of 2a, 2n, and 2u by Chlorosulfonation of Styrenes

At the beginning of the project we attempted also synthesis of some β-arylethenesulfonyl fluorides, by chlorosulfonation, followed by halogen exchange. First step was conducted at 25 mmol scale, according to the procedure: M. V. R. Reddy, M. R. Mallireddigari, V. R. Pallela, S. C. Cosenza, V. K. Billa, B. Akula, D. R. C. V. Subbaiah, E. V. Bharathi, A. Padgaonkar, H. Lv, J. M. Gallo, E. P. Reddy *J. Med. Chem.* **2013**, *56*, 5562-5586.

Second step was conducted according to the procedure: J. Dong, L. Krasnova, M. G. Finn, K. B. Sharpless *Angew. Chem. Int. Ed.* **2014**, *53*, 9430-9448.

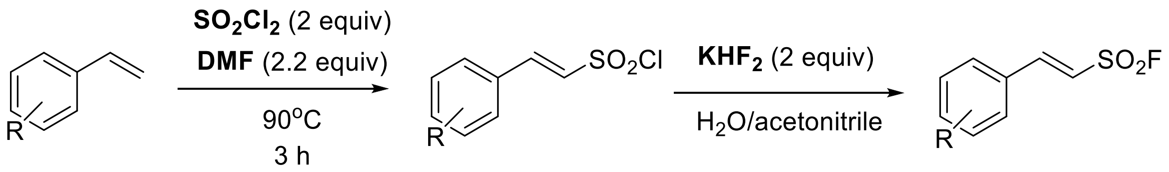

1st step product	Sulfonation yield (%)	2nd step product	Fluorination yield (%)	Overall yield (%)
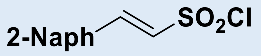	9	**2u**	94	8
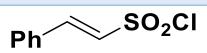	41	**2a**	98	40
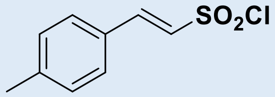	36	**2n**	97	35
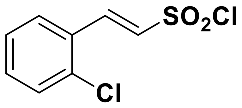	48	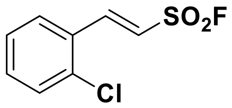	97	47
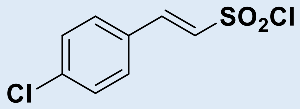	54	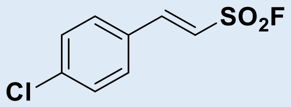	97	52

8. Single-crystal X-Ray Diffraction Analysis

8.1. Experimental

Good-quality single-crystals of **3c**, **4a**, **4o** and **5**, were mounted on a MiTeGen micro-mounts using paratone-N oil. Diffraction data were collected at 100(2) K on the Agilent Technologies SuperNova Dual Source diffractometer with Cu*K*α radiation (λ = 1.54184 Å) (**4o** and **5**) and SuperNova Single Source diffractometer with Mo*K*α radiation (λ = 0.71073 Å) (**3c** and **4a**), using CrysAlis Pro software (*CrysAlis CCD and CrysAlis RED*, Oxford Diffraction, Oxford Diffraction Ltd: Yarnton, **2008**). The analytical absorption correction using a multifaceted crystal model based on expressions derived by Clark and Reid (R. C. Clark, J. S. Reid. *Acta Cryst. Sect. A* **1994**, *51*, 887-897), implemented in SCALE3 ABSPACK scaling algorithm was applied (*CrysAlis CCD and CrysAlis RED*, Oxford Diffraction, Oxford Diffraction Ltd: Yarnton, **2008**). In the case of **4a**, **4o** and **5** the structural determination procedure was carried out using the SHELX package (G. M. Sheldrick, *Acta Cryst. Sect. C* **2015**, *71*, 3-8), while for **3c** the Superflip (L. Palatinus, G. Chapuis, *J. Appl. Cryst.* **2007**, *40*, 786-790) was used. The structures were solved with direct methods and then successive least-square refinement was carried out based on the full-matrix least-squares method on F^2 using the SHELXL program (G. M. Sheldrick, *Acta Cryst. Sect. C* **2015**, *71*, 3-8). All H-atoms were positioned geometrically, with C–H equal to 0.93, 0.96 and 0.98 Å for the aromatic/methene, methyl and methylene H-atoms, respectively, and constrained to ride on their parent atoms with U_{iso}(H) = xU_{eq}(C), where x = 1.2 for the aromatic, methine and methylene, and 1.5 for the methyl H-atoms, respectively. The figures presented below were prepared using Olex2 program (O.V. Dolomanov, L. J. Bourhis, R. J. Gildea, J.A.K. Howard, H. Puschmann, *J. Appl. Cryst.* **2009**, *42*, 339-341).

8.2. Results

Table 1. Crystal data and structure refinement details for investigated compounds.

**Compound**	**3c**	**4a**	**4o**	**5**
Empirical formula	$C_9H_7FO_6S_2$	$C_{10}H_8F_2O_4S_2$	$C_{10}H_{10}F_2O_6S_2$	$C_9H_{12}F_2N_2O_4S_2$
Formula weight	294.27	294.28	328.30	314.33
Temperature/K	100(2)	100(2)	100(2)	100(2)
Crystal system	monoclinic	monoclinic	triclinic	monoclinic
Space group	$P2_1/c$	$P2_1/c$	P-1	$P2_1/c$
a/Å	7.05444(19)	14.9098(7)	5.1189(4)	9.93918(12)
b/Å	8.4578(3)	11.9852(6)	9.9945(5)	10.51160(13)
c/Å	18.7175(5)	13.6860(6)	12.7428(5)	12.41653(16)
α/°	90	90	88.874(4)	90
β/°	100.128(3)	107.084(5)	87.593(5)	102.5299(13)
γ/°	90	90	77.039(5)	90
Volume/Å^3	1099.38(6)	2337.7(2)	634.73(6)	1266.34(3)
Z	4	8	2	4
ρ_{calc}g/cm^3	1.778	1.672	1.718	1.649
μ/mm^{-1}	0.516	0.485	4.306	4.206
F(000)	600.0	1200.0	336.0	648.0
Crystal size/mm^3	0.40 × 0.19 × 0.14	0.58 × 0.44 × 0.17	0.45 × 0.13 × 0.10	0.16 × 0.15 × 0.12
Radiation	Mo$K\alpha$ (λ = 0.71073)	Mo$K\alpha$ (λ = 0.71073)	Cu$K\alpha$ (λ = 1.54184)	Cu$K\alpha$ (λ = 1.54184)
2Θ range for data collection/°	4.422 to 52.72	4.61 to 52.744	6.944 to 134.078	9.114 to 134.112
Index ranges	$-7 \leq h \leq 8$, $-10 \leq k \leq 8$, $-22 \leq l \leq 23$	$-18 \leq h \leq 18$, $-14 \leq k \leq 8$, $-17 \leq l \leq 16$	$-5 \leq h \leq 6$, $-11 \leq k \leq 11$, $-15 \leq l \leq 15$	$-11 \leq h \leq 11$, $-12 \leq k \leq 12$, $-14 \leq l \leq 14$
Reflections collected	4073	9795	9811	23251
Independent reflections	2250 [R_{int} = 0.0271, R_{sigma} = 0.0551]	4772 [R_{int} = 0.0363, R_{sigma} = 0.0686]	2253 [R_{int} = 0.0566, R_{sigma} = 0.0417]	2261 [R_{int} = 0.0411, R_{sigma} = 0.0177]
Data/restraints/parameters	2250/0/164	4772/0/325	2253/0/183	2261/0/174
Goodness-of-fit on F^2	1.089	1.056	1.058	1.049
Final R indexes [I>=2σ (I)]	R_1 = 0.0407, wR_2 = 0.0870	R_1 = 0.0453, wR_2 = 0.0865	R_1 = 0.0427, wR_2 = 0.1204	R_1 = 0.0285, wR_2 = 0.0735
Final R indexes [all data]	R_1 = 0.0542, wR_2 = 0.0981	R_1 = 0.0644, wR_2 = 0.0999	R_1 = 0.0462, wR_2 = 0.1242	R_1 = 0.0295, wR_2 = 0.0743
Largest diff. peak/hole / e Å^{-3}	0.40/-0.54	0.49/-0.51	0.65/-0.46	0.39/-0.49

8.3. Plots of X-ray structures of 3c, 4a, 4o and 5

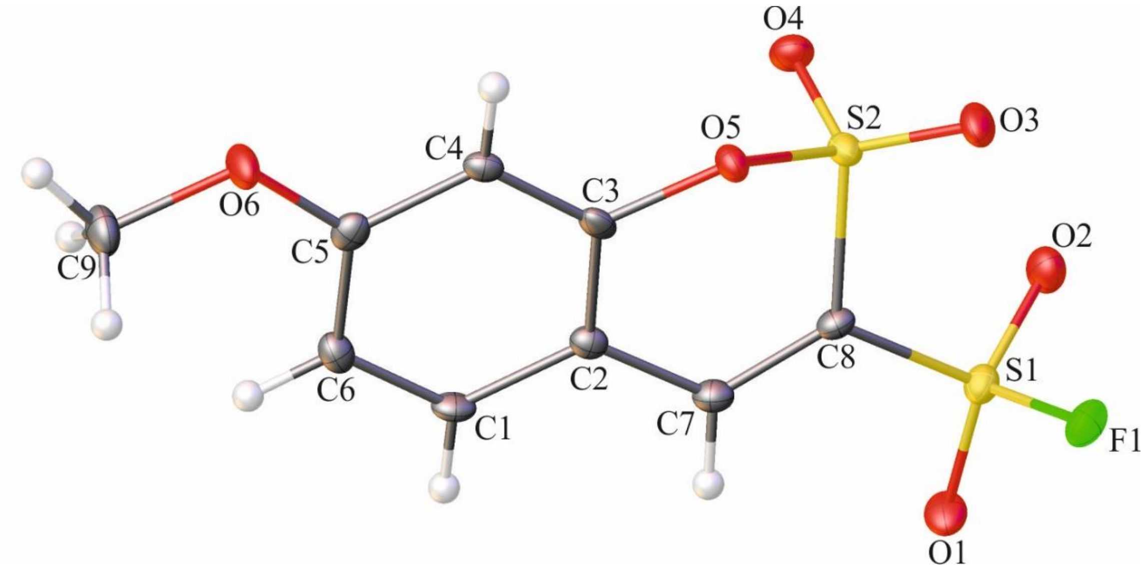

Figure 1. Asymmetric unit of the crystal lattice of **3c** with the crystallographic atom numbering. Displacement ellipsoids are drawn at the 50% probability level and the H atoms are shown as small spheres of arbitrary radius.

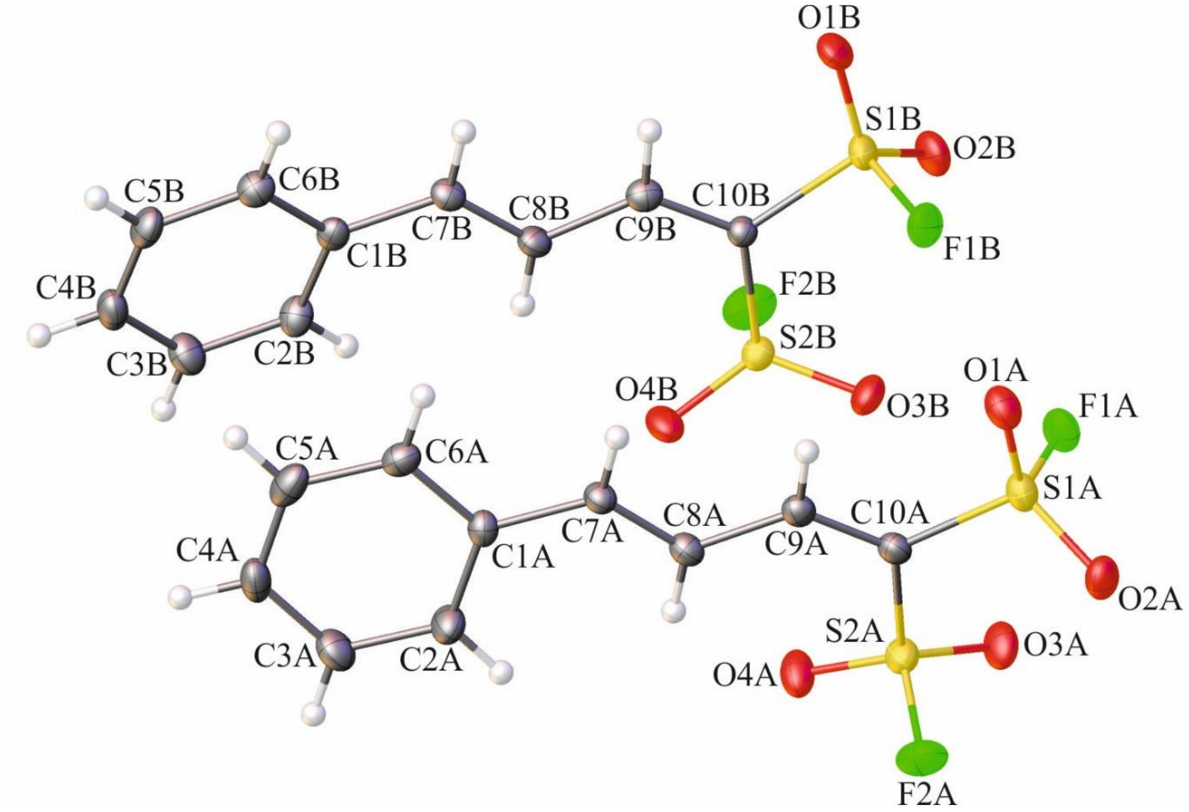

Figure 2. Asymmetric unit of the crystal lattice of **4a** with the crystallographic atom numbering. Displacement ellipsoids are drawn at the 50% probability level and the H atoms are shown as small spheres of arbitrary radius.

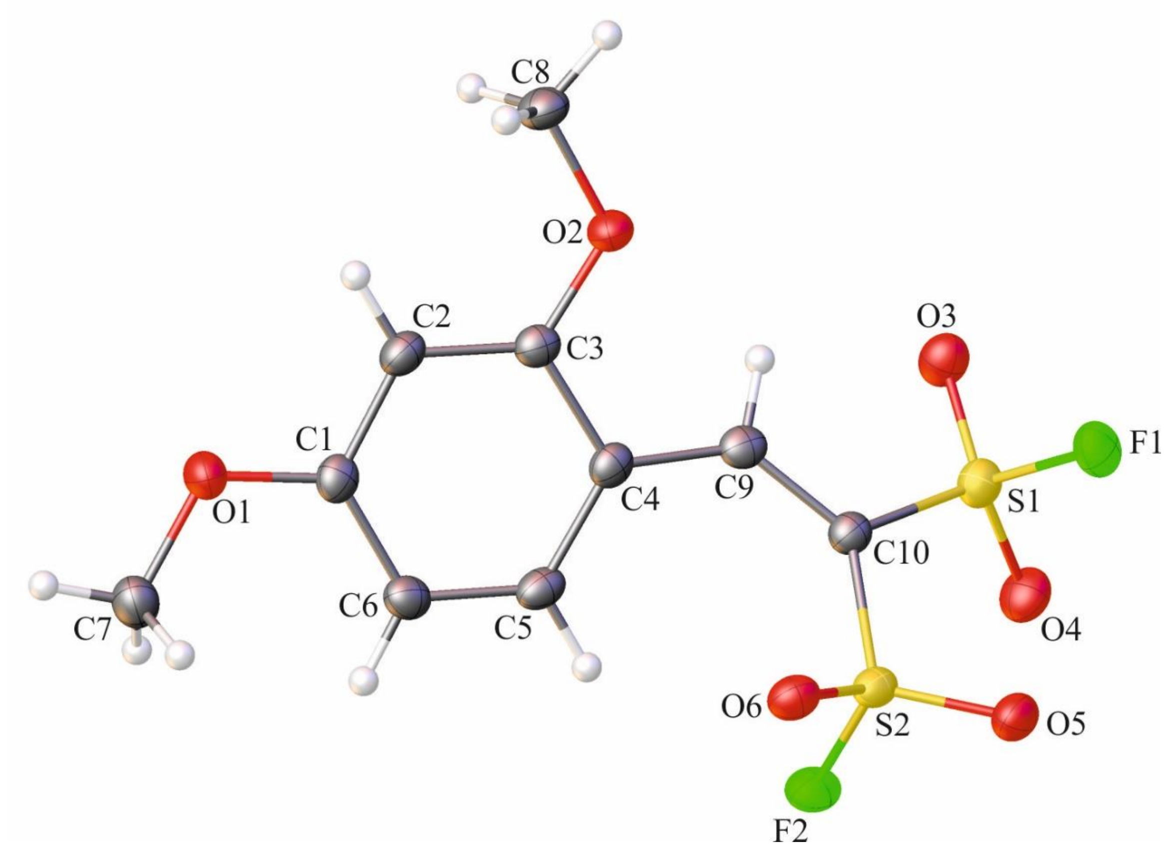

Figure 3. Asymmetric unit of the crystal lattice of **4o** with the crystallographic atom numbering. Displacement ellipsoids are drawn at the 50% probability level and the H atoms are shown as small spheres of arbitrary radius.

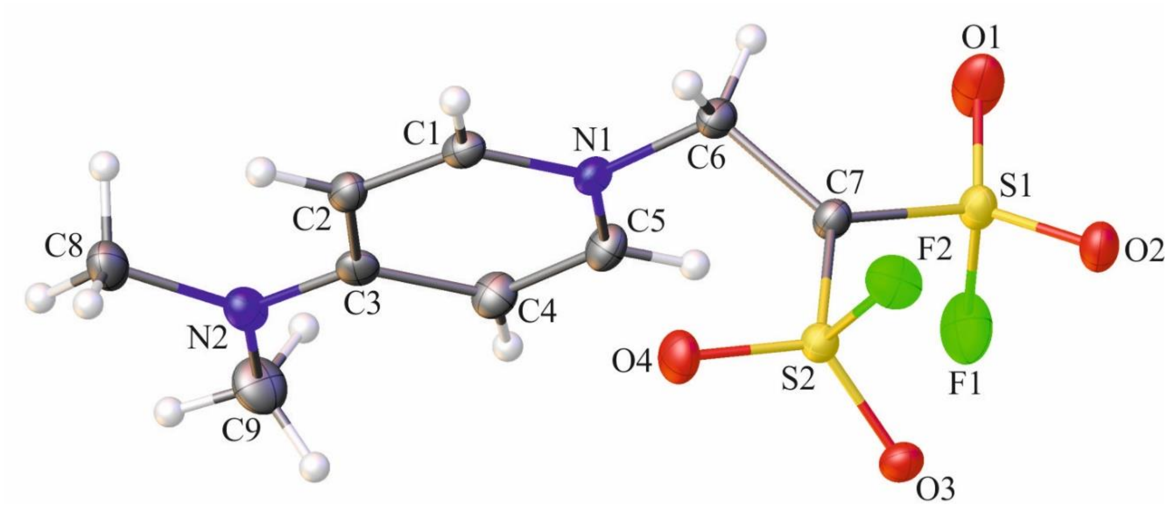

Figure 4. Asymmetric unit of the crystal lattice of **5** with the crystallographic atom numbering. Displacement ellipsoids are drawn at the 50% probability level and the H atoms are shown as small spheres of arbitrary radius.

9. ^{1}H, ^{13}C and ^{19}F NMR Spectra of Characterized Compounds

^{1}H NMR (400 MHz, $CDCl_3$):

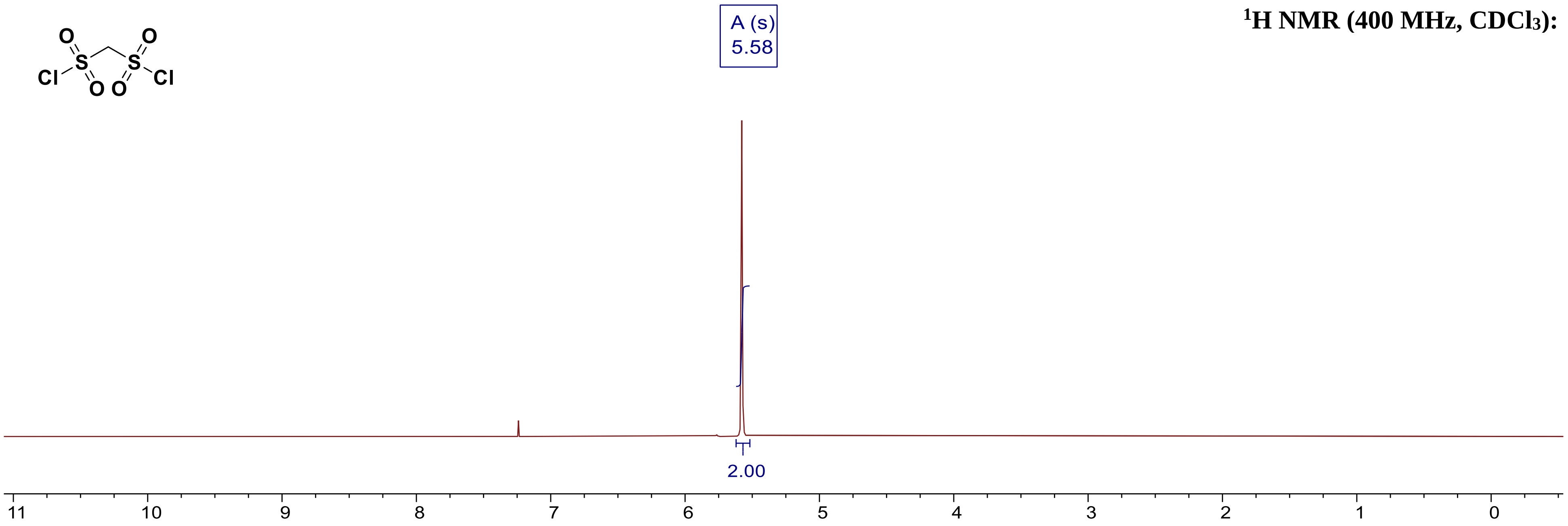

^{13}C NMR (100 MHz, $CDCl_3$):

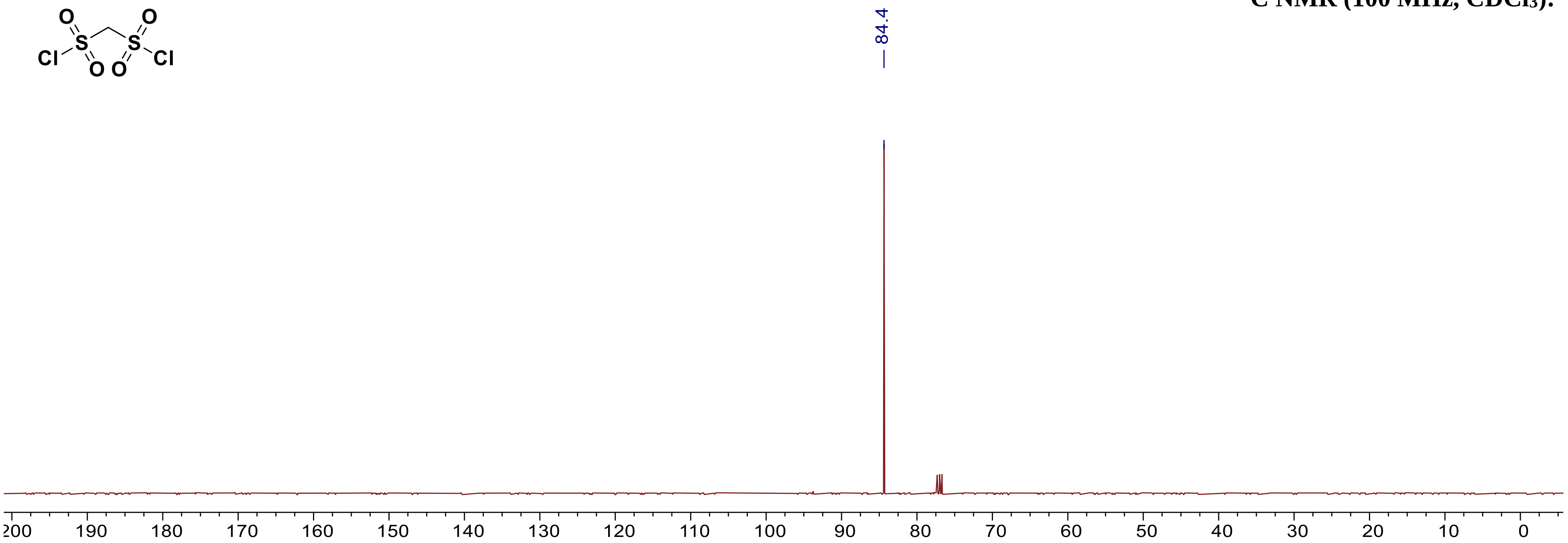

^{1}H NMR (400 MHz, $CDCl_3$):

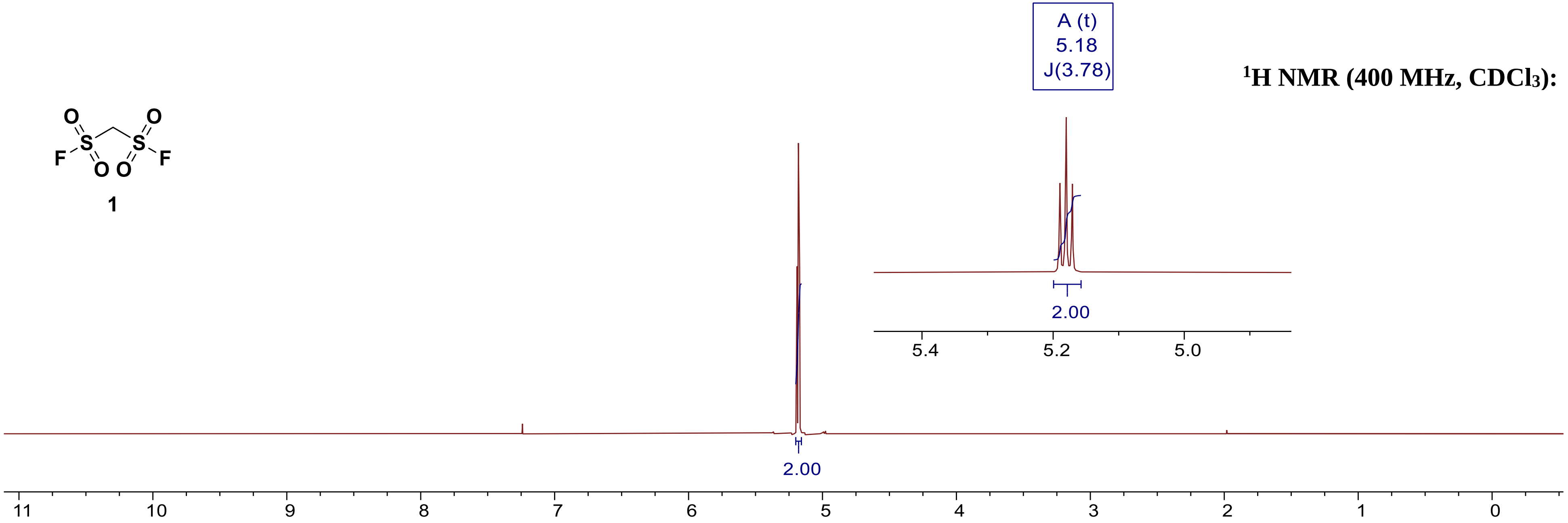

^{13}C NMR (100 MHz, $CDCl_3$):

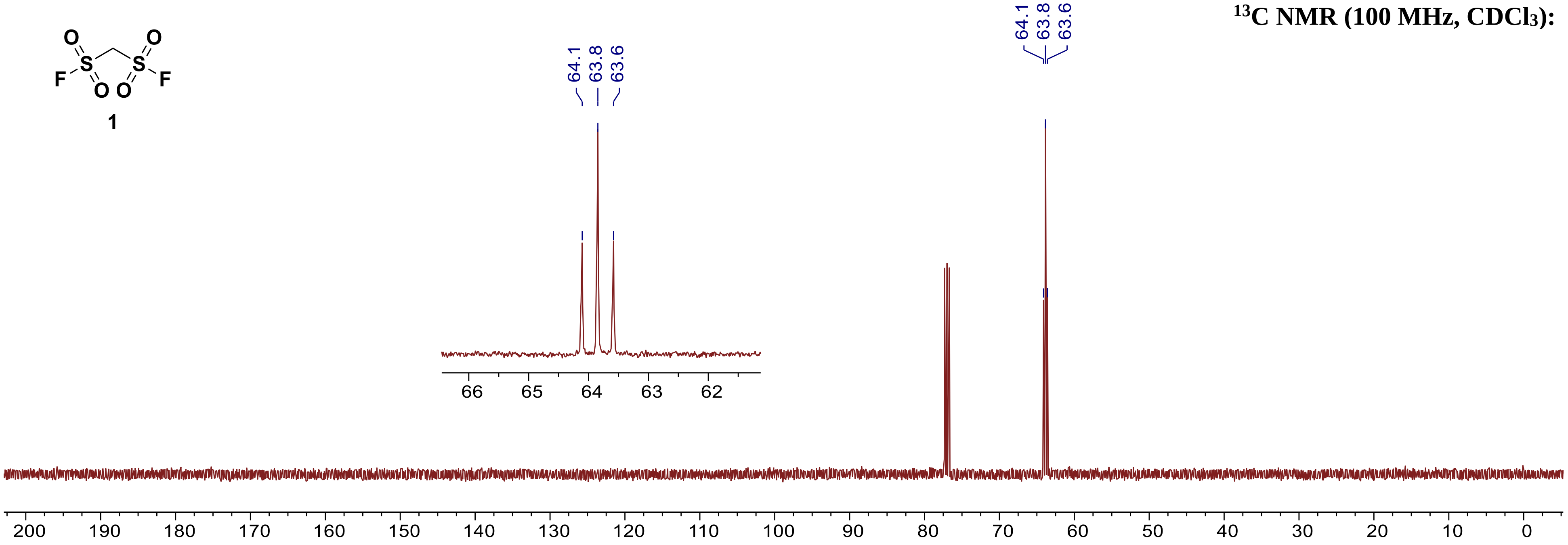

^{19}F NMR (376 MHz, CDCl3):

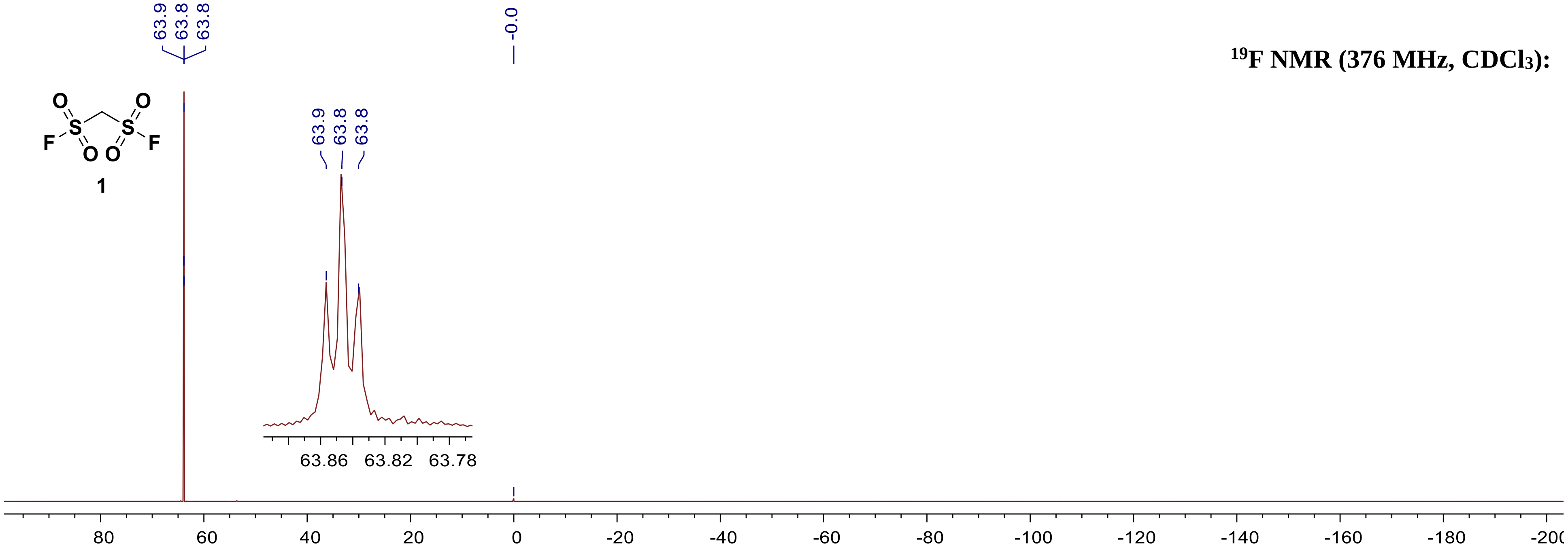

^{1}H NMR (400 MHz, CDCl$_3$):

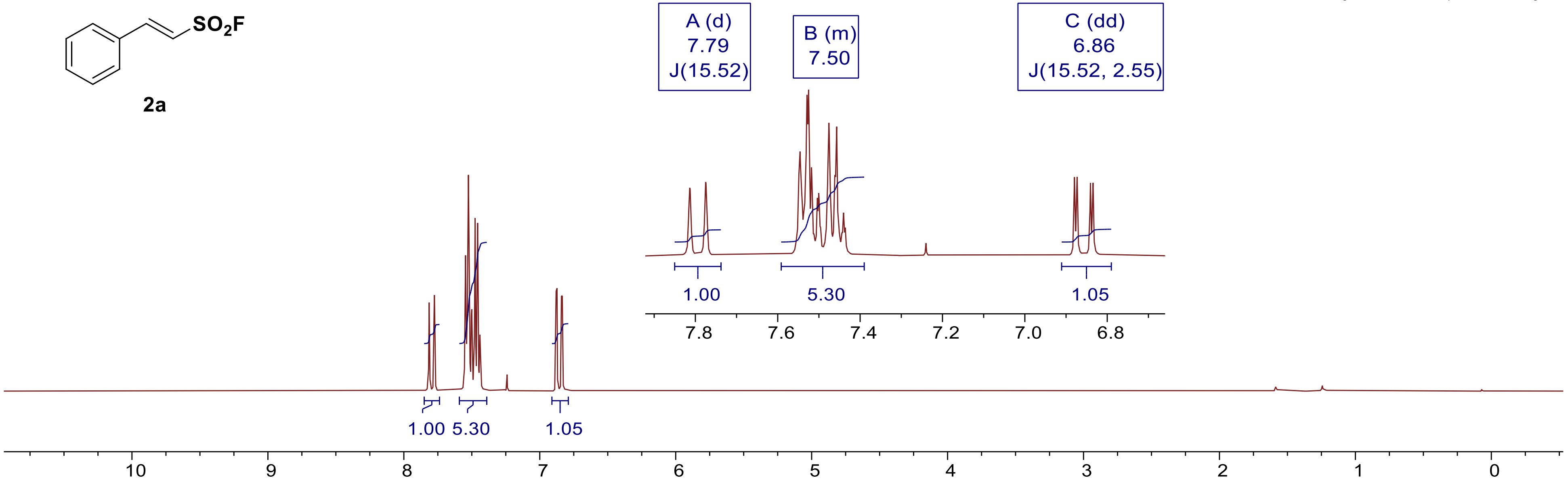

^{13}C NMR (100 MHz, CDCl$_3$):

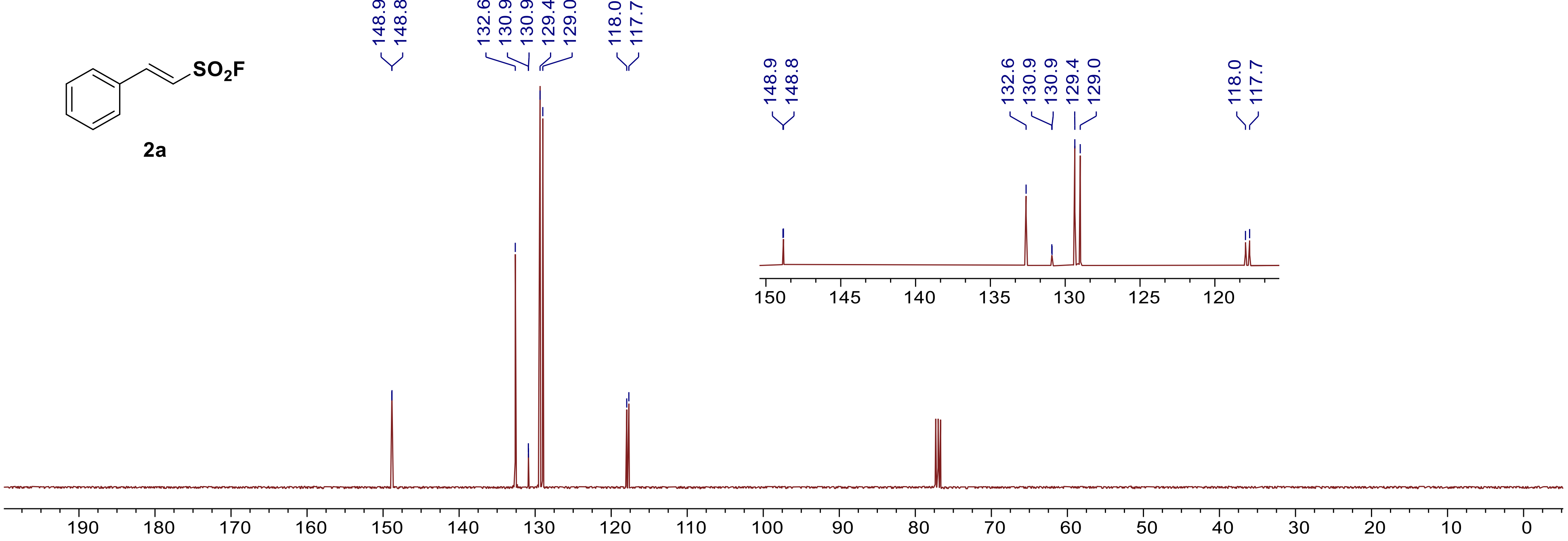

^{19}F NMR (376 MHz, CDCl3):

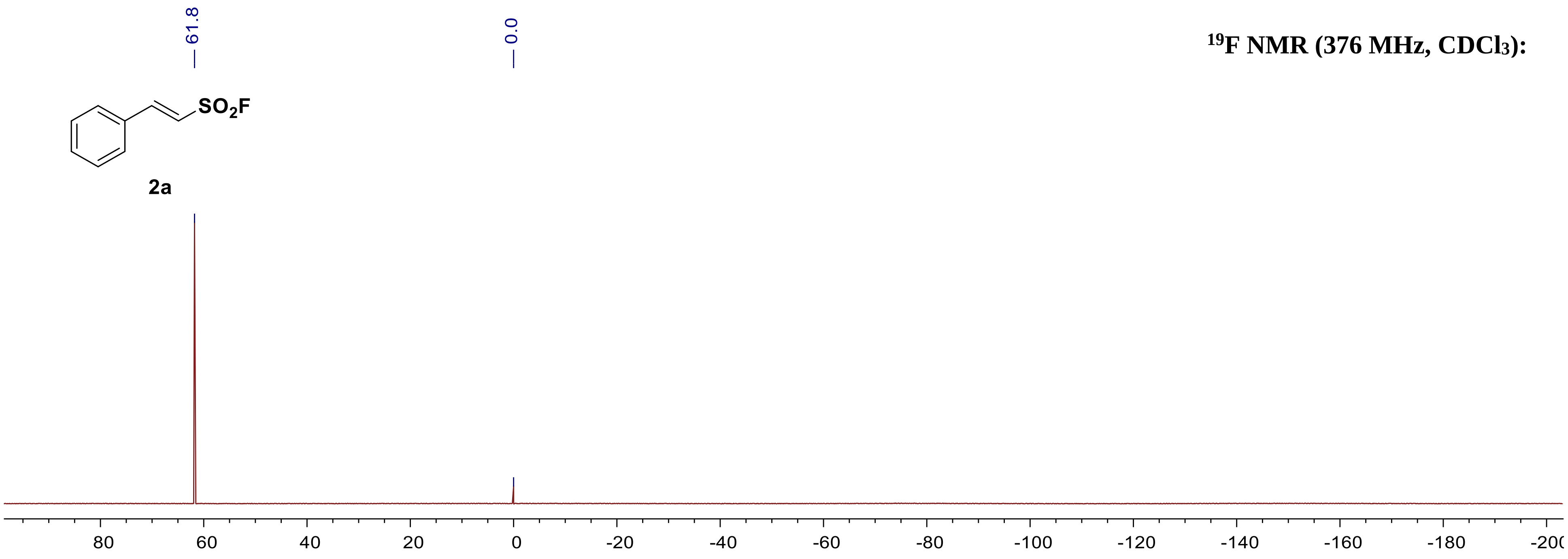

^{1}H NMR (400 MHz, $CDCl_3$):

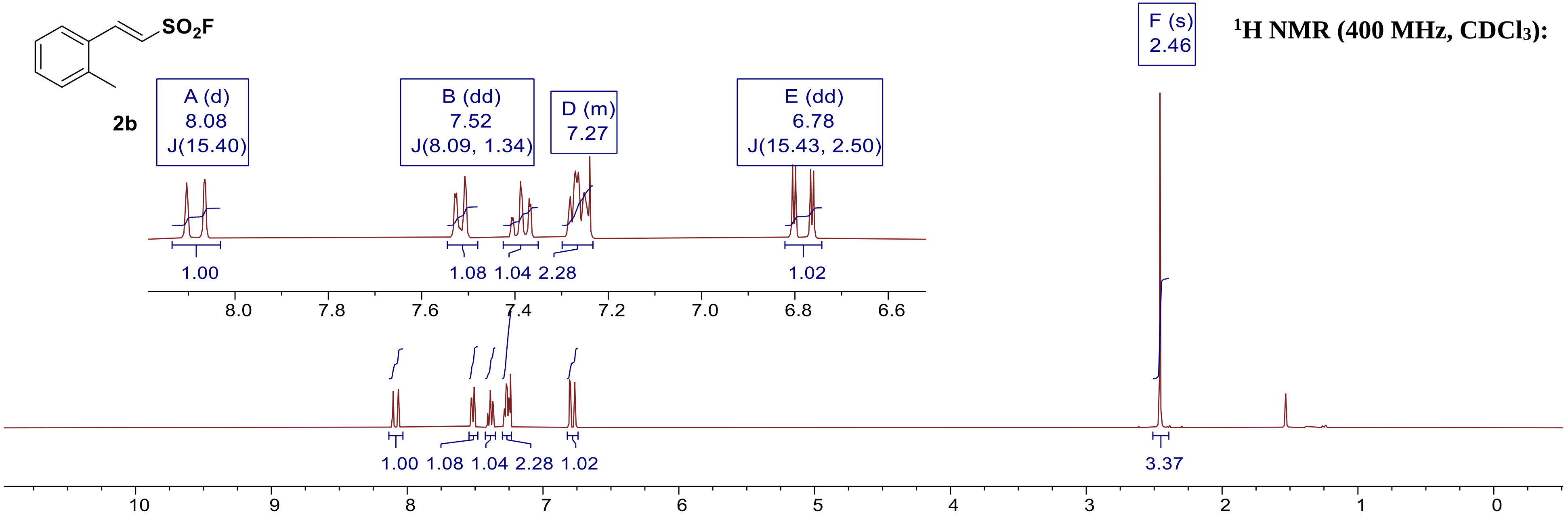

^{13}C NMR (100 MHz, $CDCl_3$):

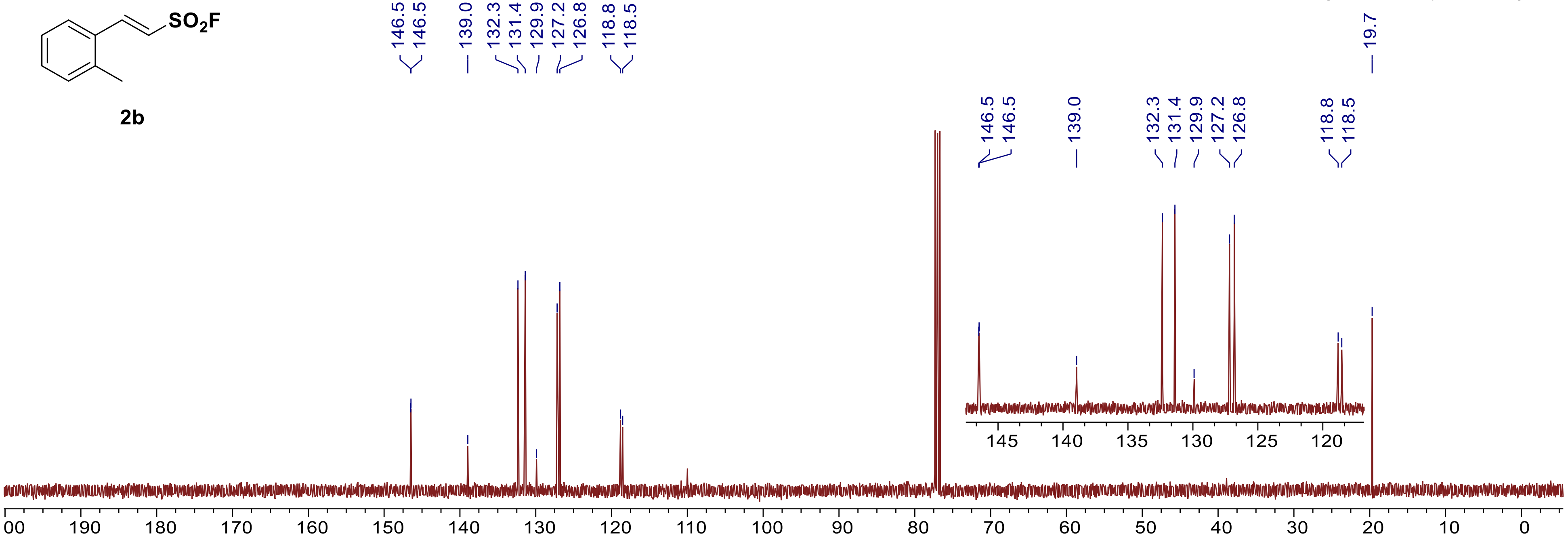

^{19}F NMR (376 MHz, $CDCl_3$):

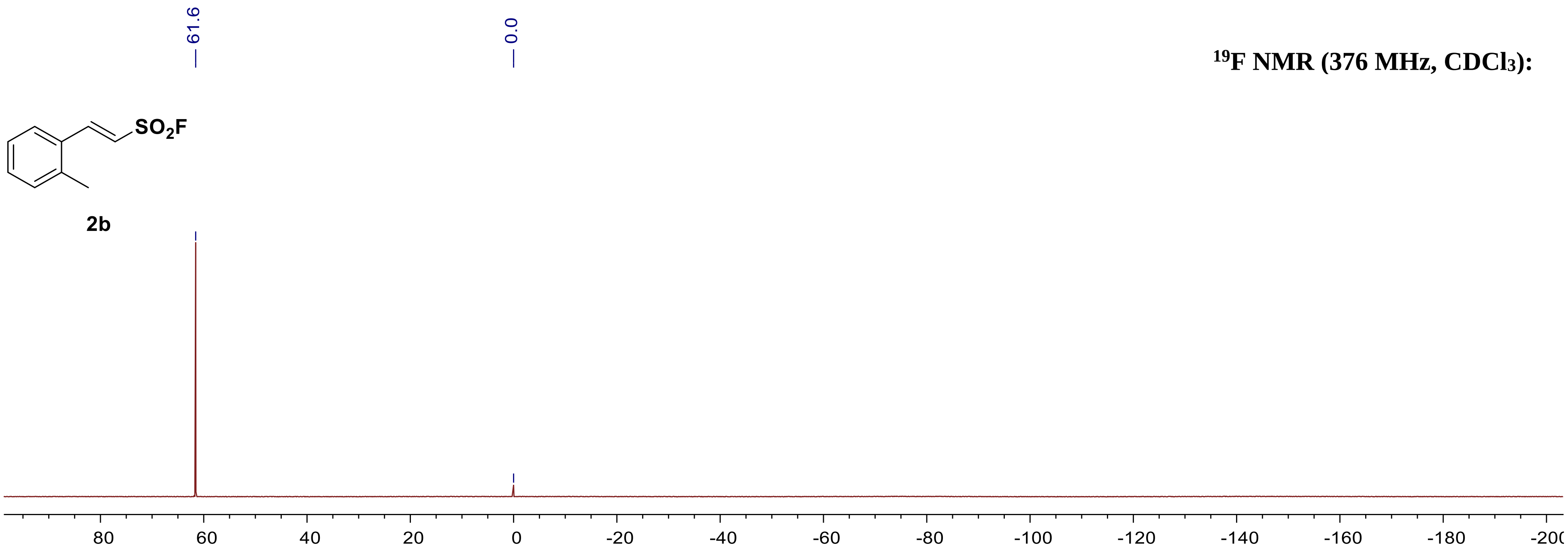

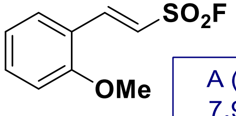

2c

¹H NMR (400 MHz, CDCl₃):

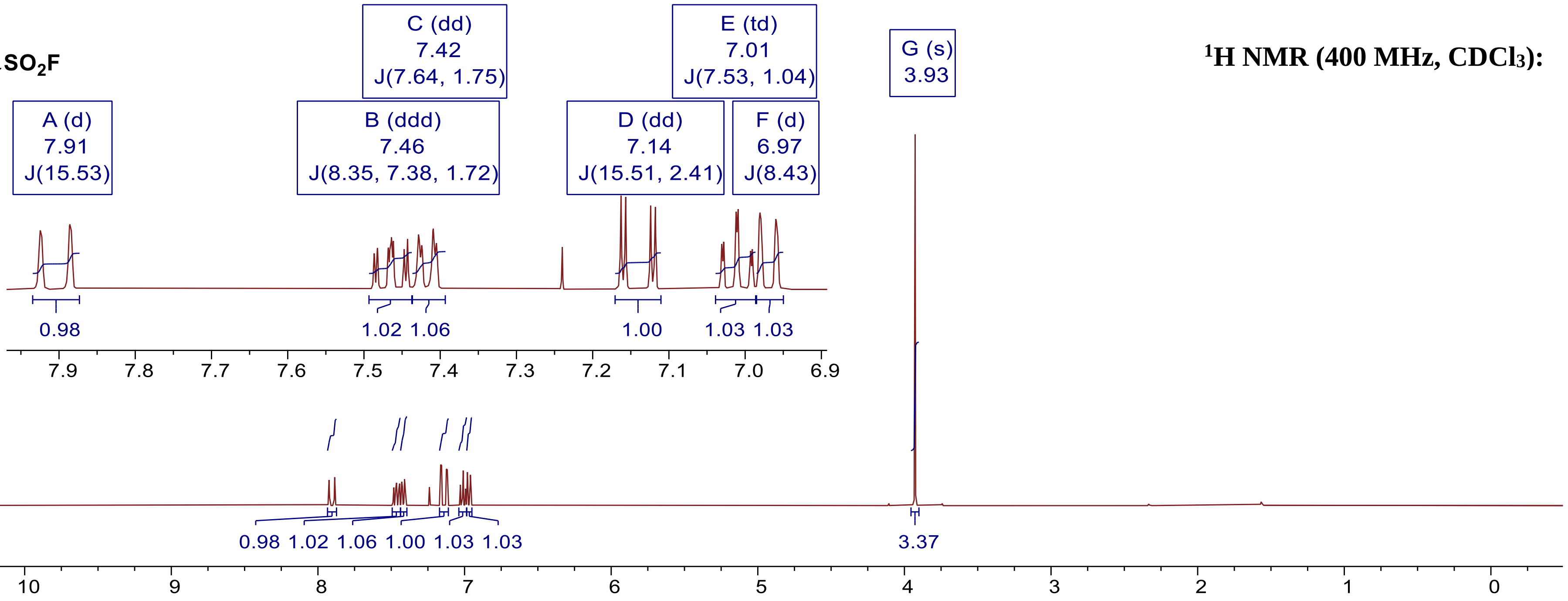

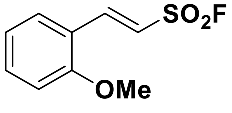

2c

¹³C NMR (100 MHz, CDCl₃):

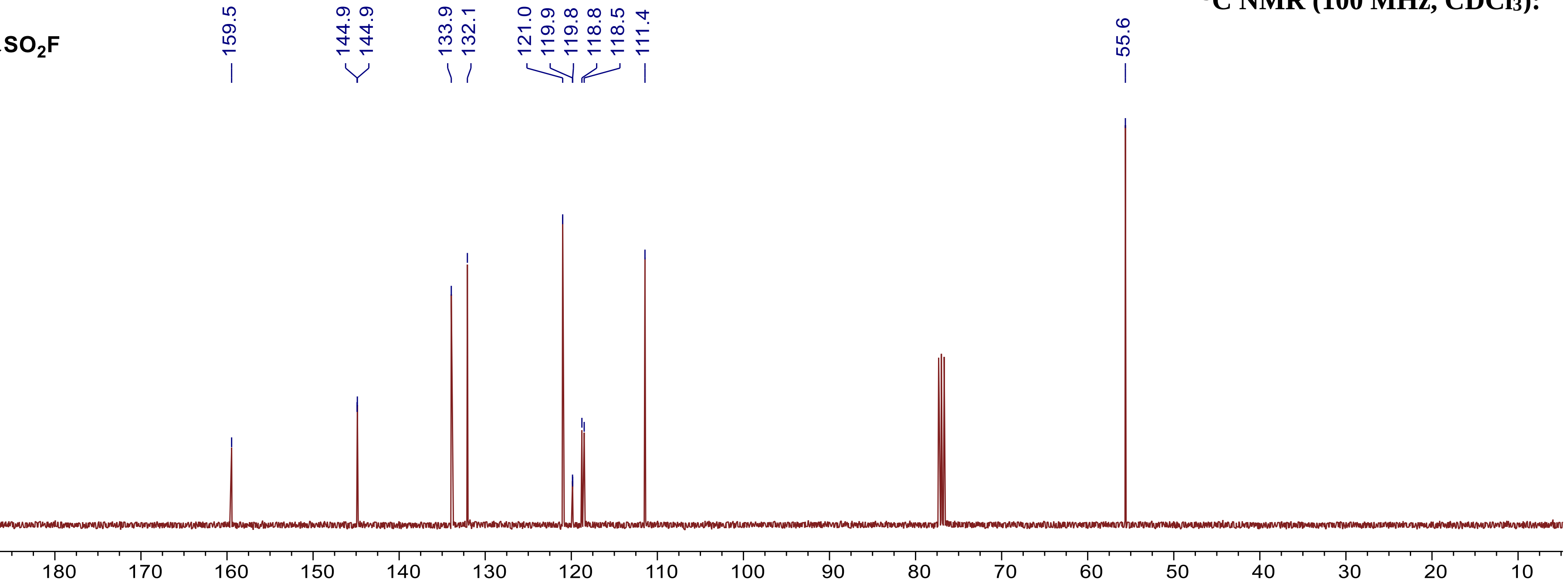

^{19}F NMR (376 MHz, CDCl$_3$):

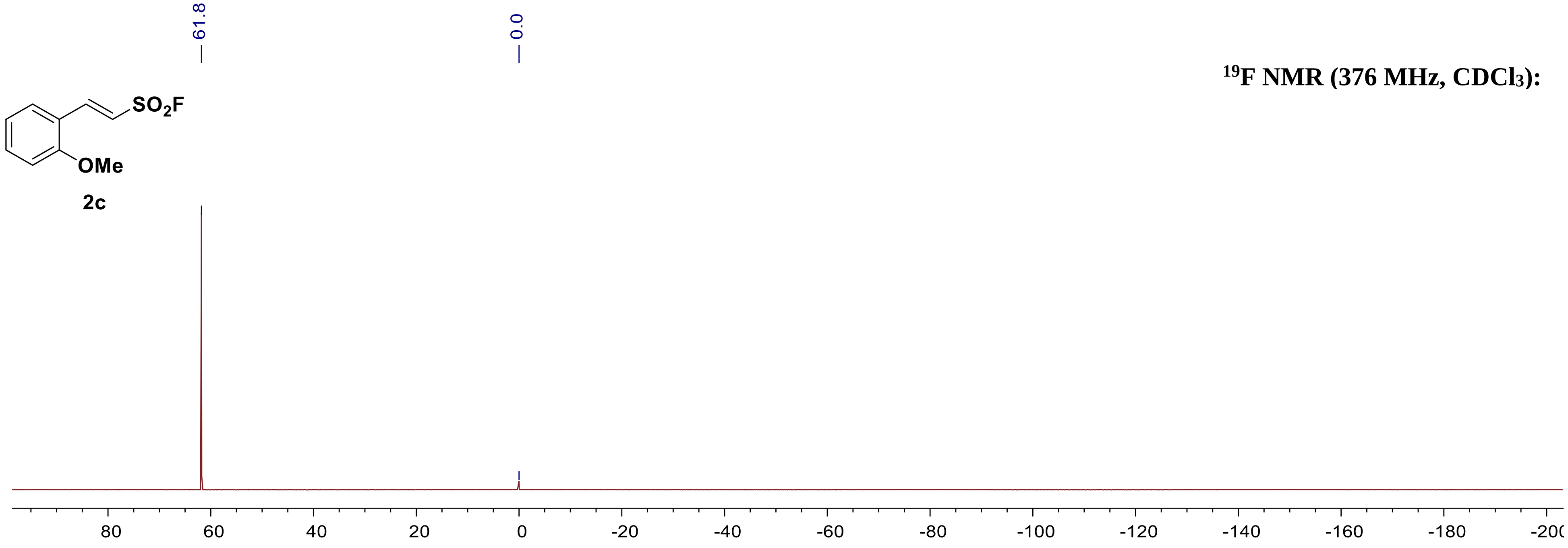

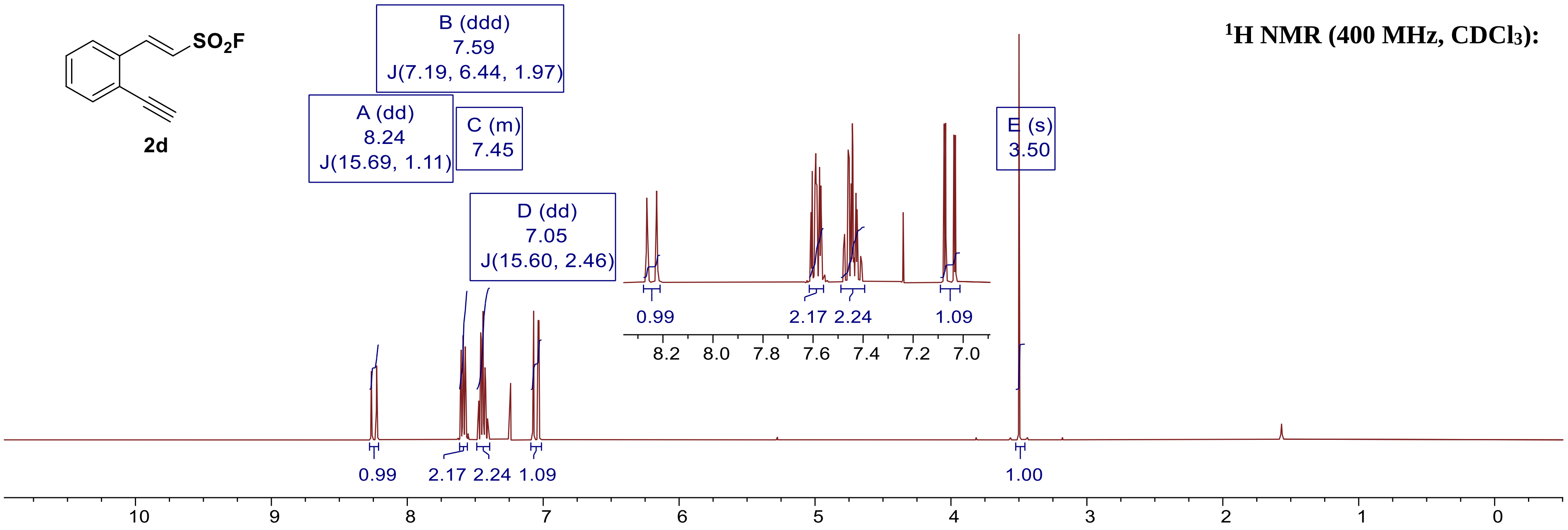

^{1}H NMR (400 MHz, $CDCl_3$):
2d
A (dd) 8.24 J(15.69, 1.11)
B (ddd) 7.59 J(7.19, 6.44, 1.97)
C (m) 7.45
D (dd) 7.05 J(15.60, 2.46)
E (s) 3.50
0.99 2.17 2.24 1.09 1.00

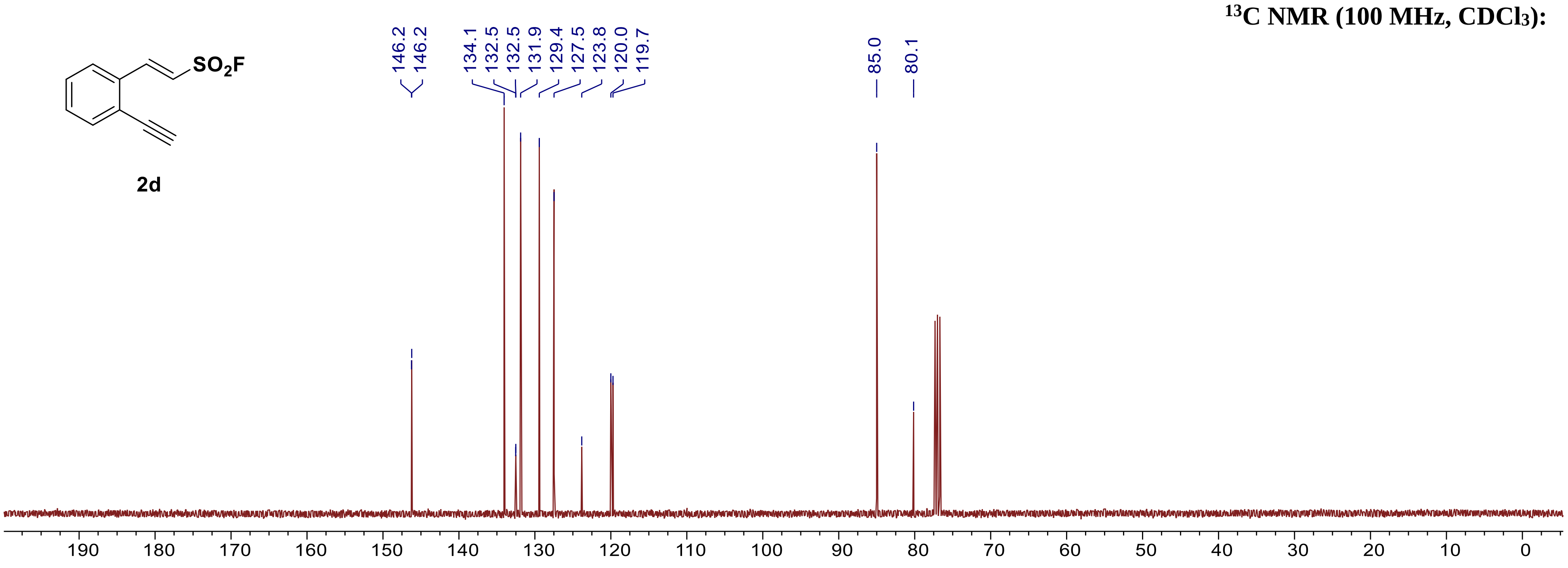

^{13}C NMR (100 MHz, $CDCl_3$):
2d
146.2 146.2 134.1 132.5 132.5 131.9 129.4 127.5 123.8 120.0 119.7 85.0 80.1

^{19}F NMR (376 MHz, $CDCl_3$):

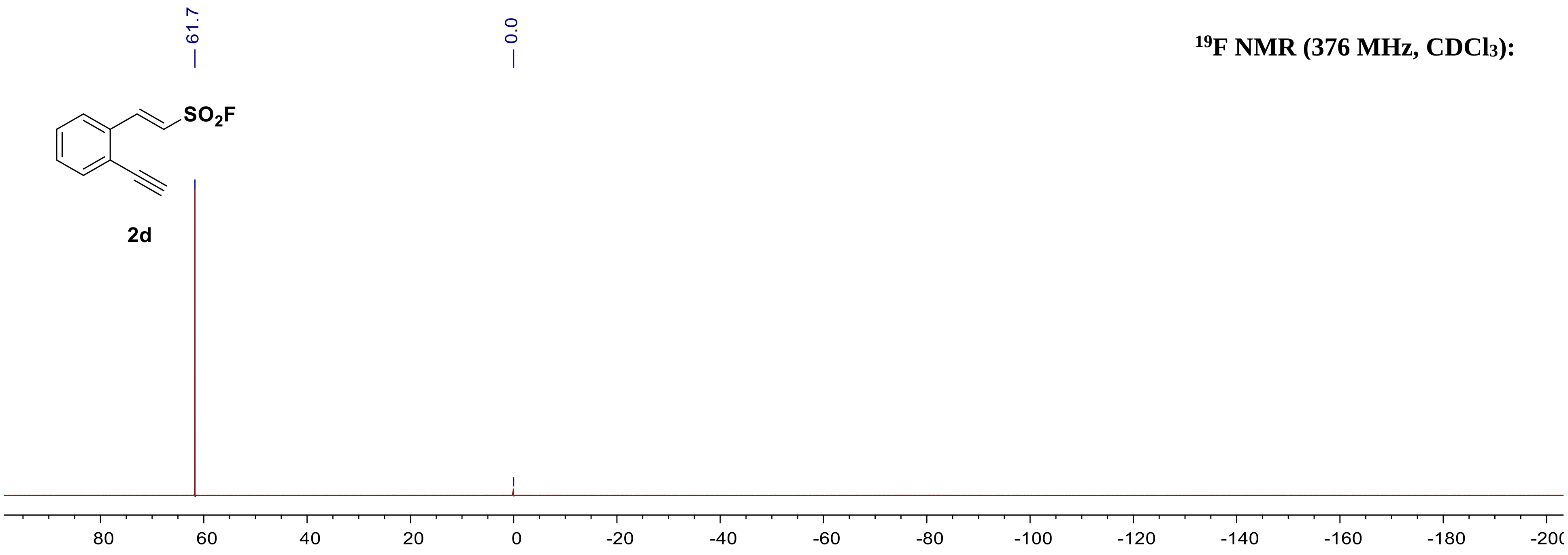

^{1}H NMR (400 MHz, $CDCl_3$):

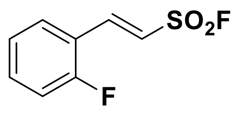

2e

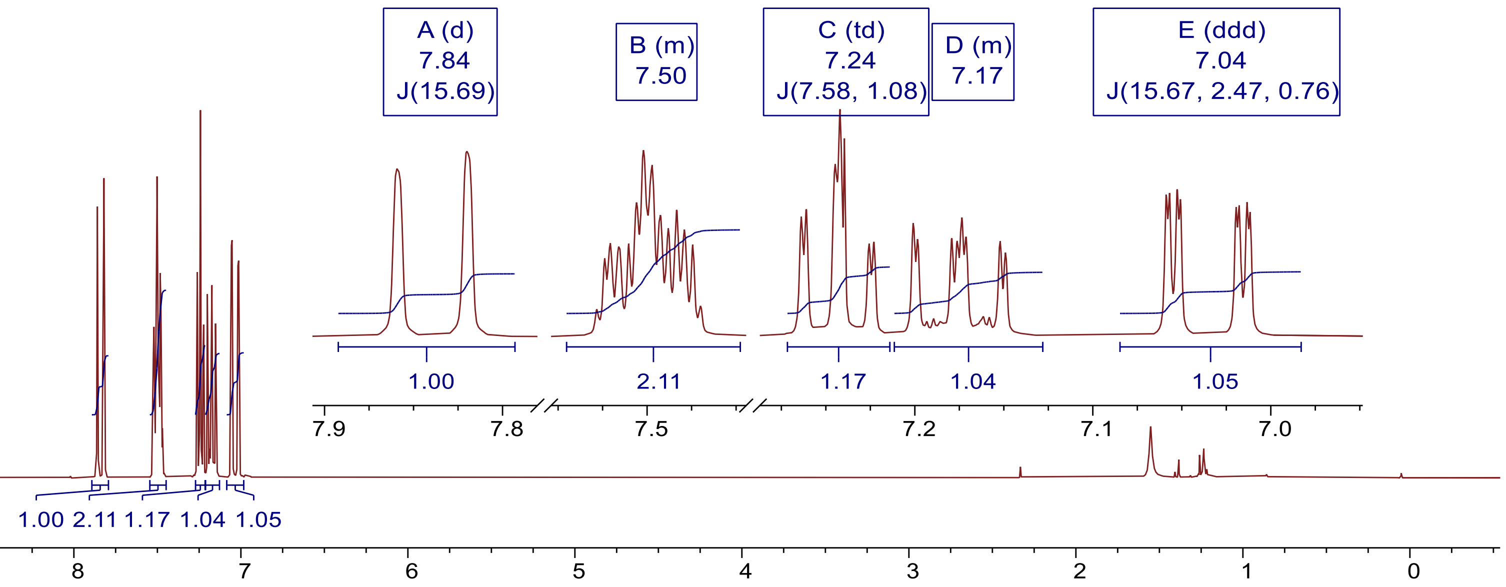

^{13}C NMR (100 MHz, $CDCl_3$):

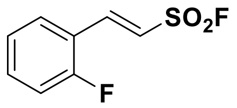

2e

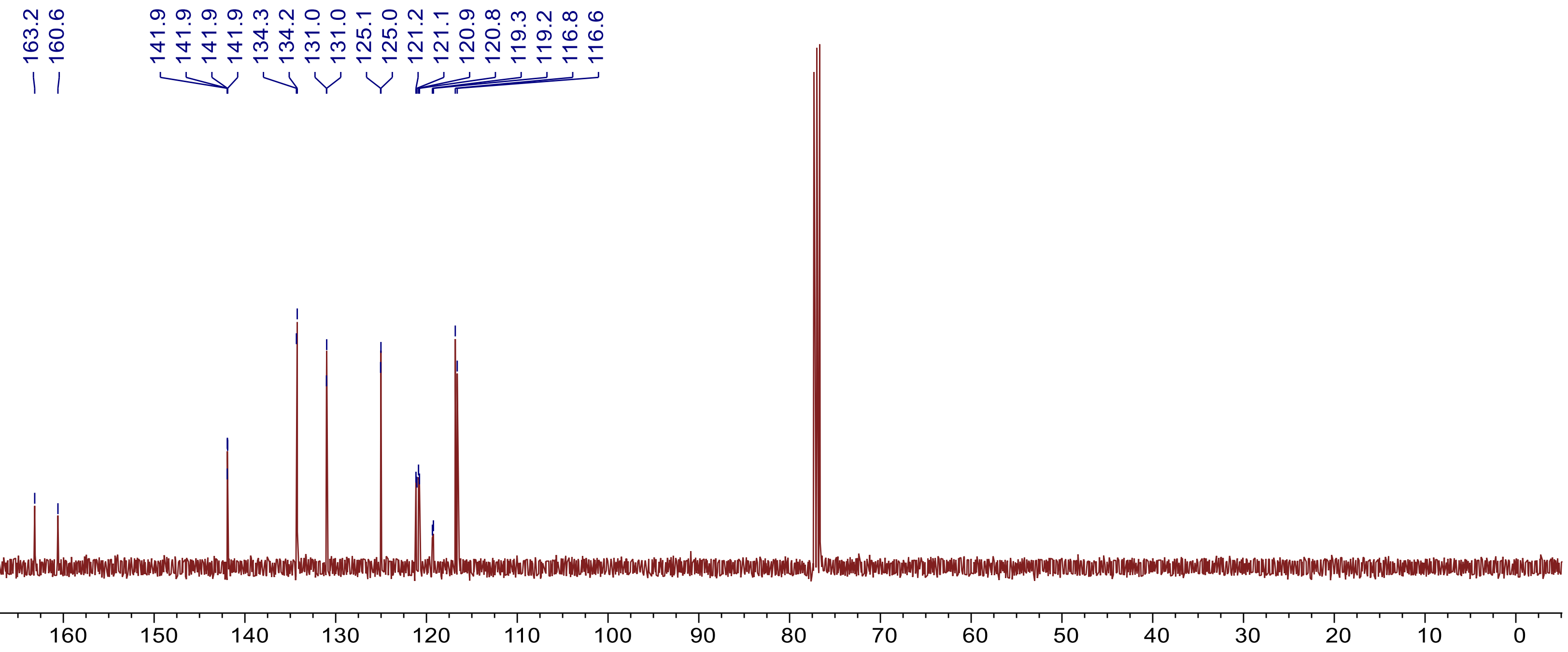

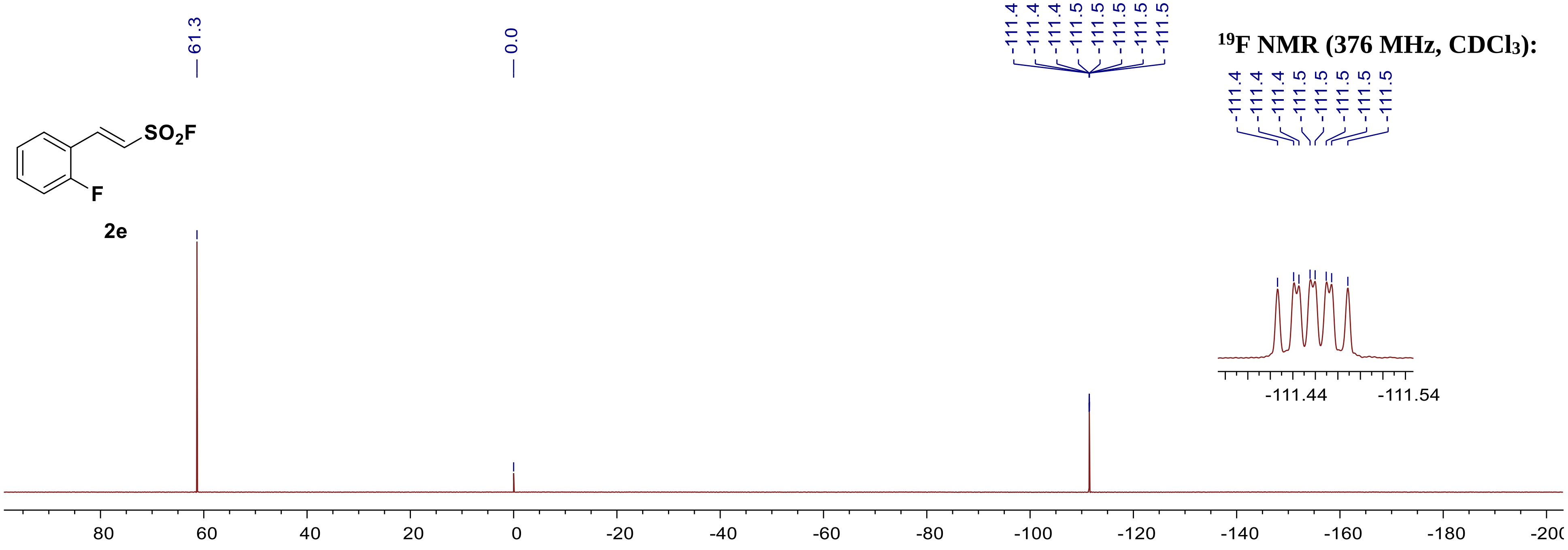

¹⁹F NMR (376 MHz, CDCl₃):
2e
SO2F
F
61.3
0.0
-111.4
-111.4
-111.4
-111.5
-111.5
-111.5
-111.5
-111.5
-111.44
-111.54
80
60
40
20
0
-20
-40
-60
-80
-100
-120
-140
-160
-180
-200

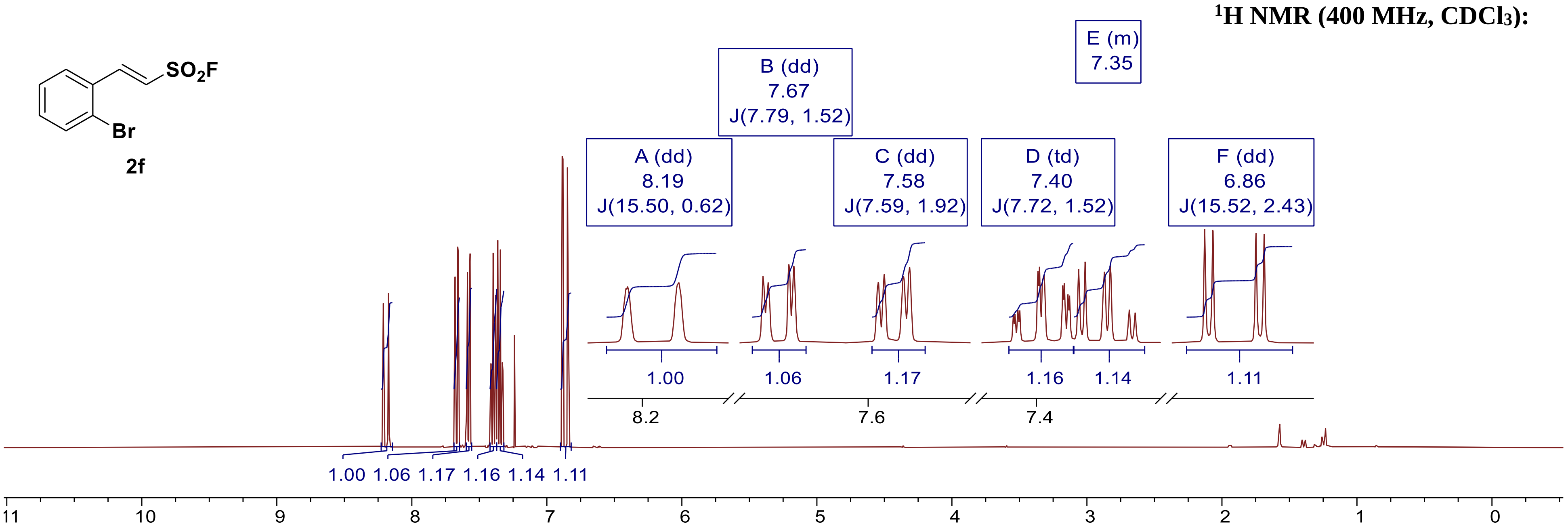

^{1}H NMR (400 MHz, CDCl3):
SO2F
Br
2f
A (dd) 8.19 J(15.50, 0.62)
B (dd) 7.67 J(7.79, 1.52)
C (dd) 7.58 J(7.59, 1.92)
D (td) 7.40 J(7.72, 1.52)
E (m) 7.35
F (dd) 6.86 J(15.52, 2.43)
1.00 1.06 1.17 1.16 1.14 1.11
8.2 7.6 7.4
11 10 9 8 7 6 5 4 3 2 1 0

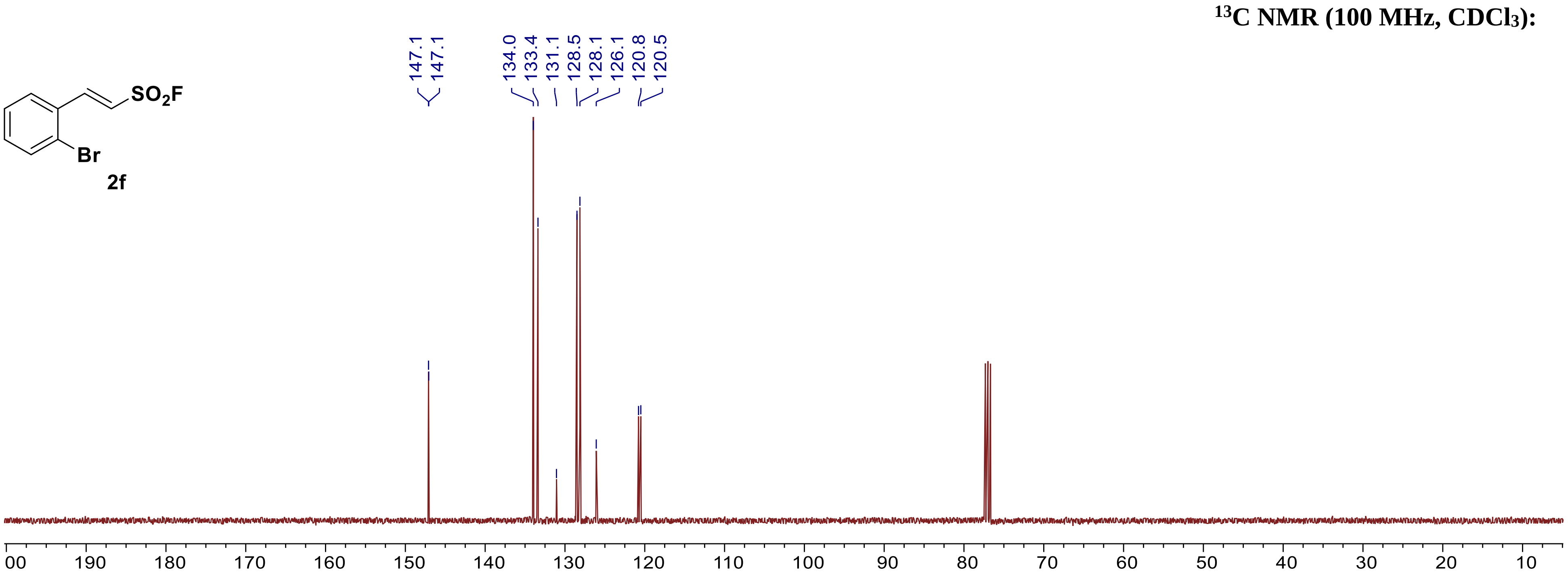

^{13}C NMR (100 MHz, CDCl3):
SO2F
Br
2f
147.1 147.1 134.0 133.4 131.1 128.5 128.1 126.1 120.8 120.5
00 190 180 170 160 150 140 130 120 110 100 90 80 70 60 50 40 30 20 10

^{19}F NMR (376 MHz, $CDCl_3$):

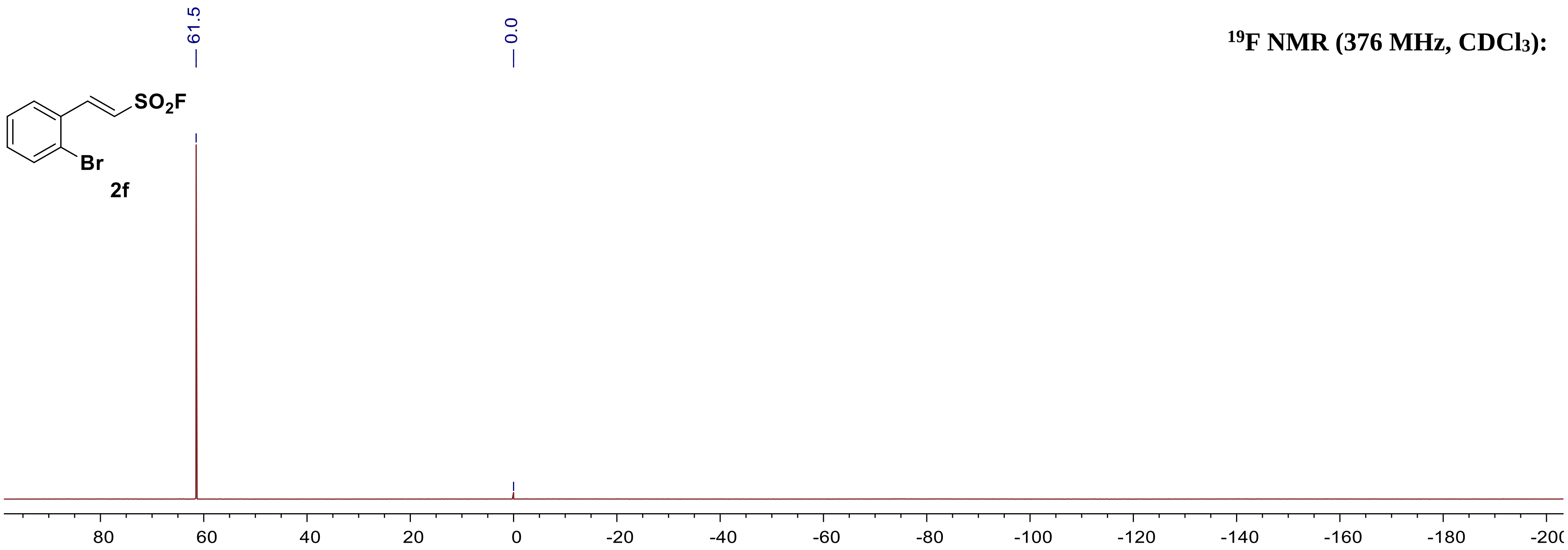

^{1}H NMR (400 MHz, CDCl$_3$):

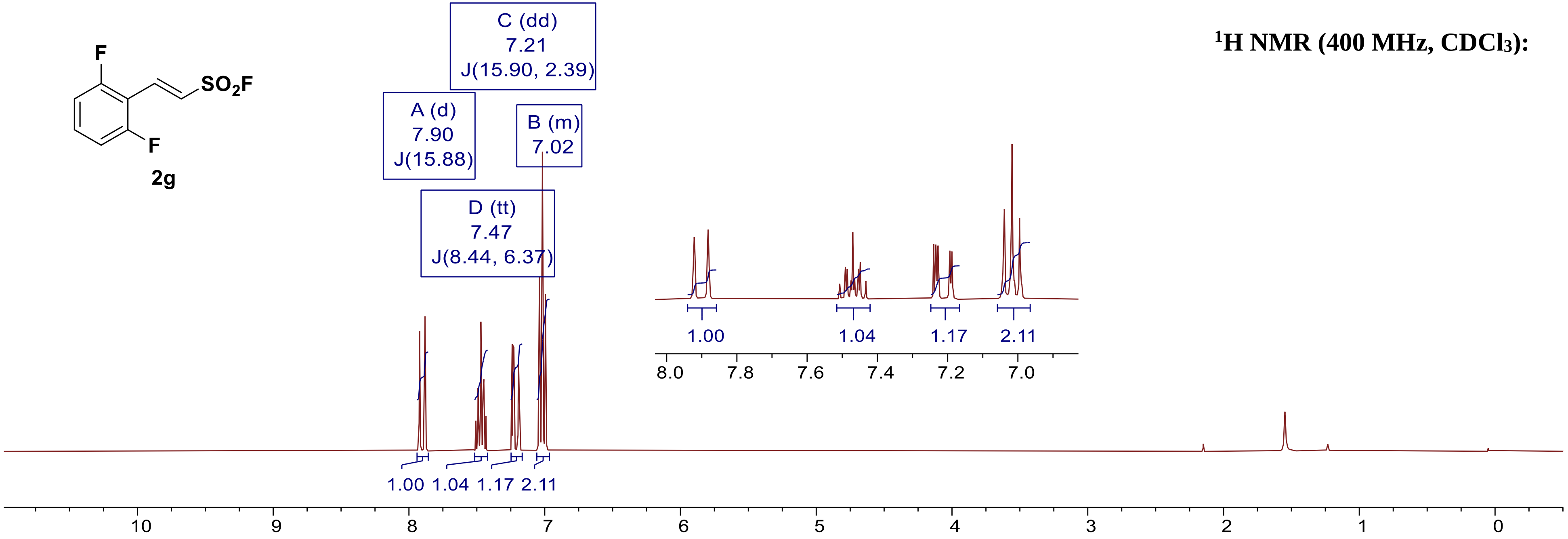

^{13}C NMR (100 MHz, CDCl$_3$):

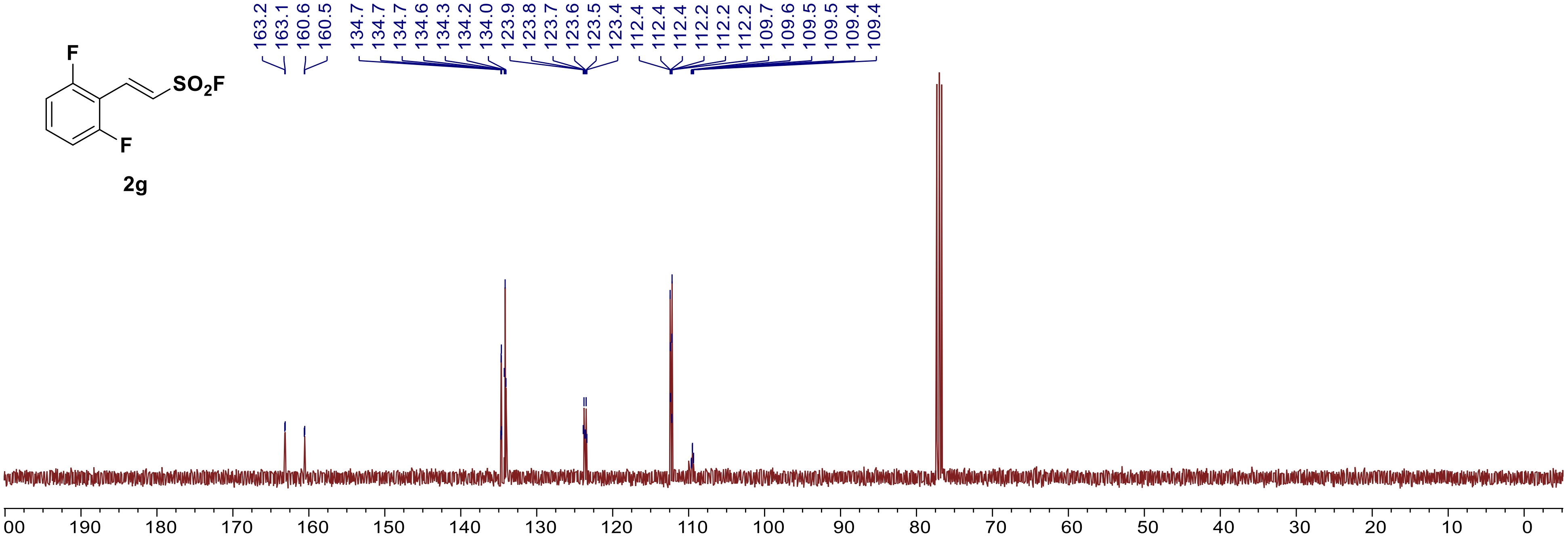

^{19}F NMR (376 MHz, CDCl$_3$):

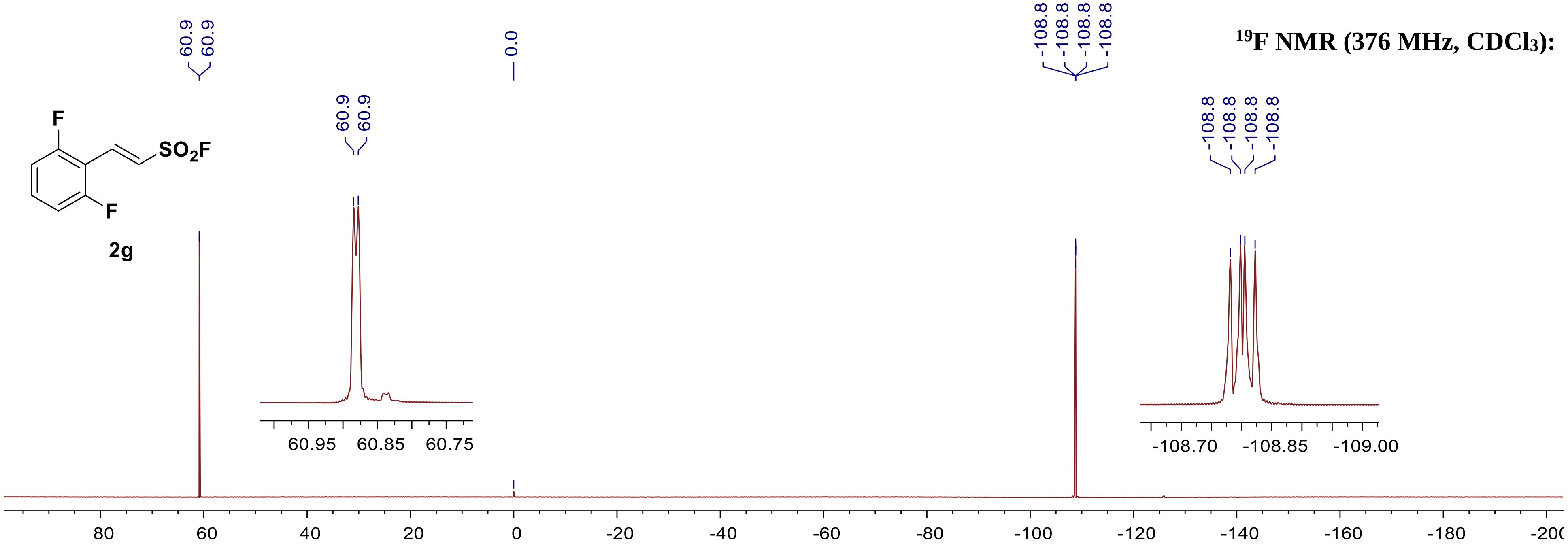

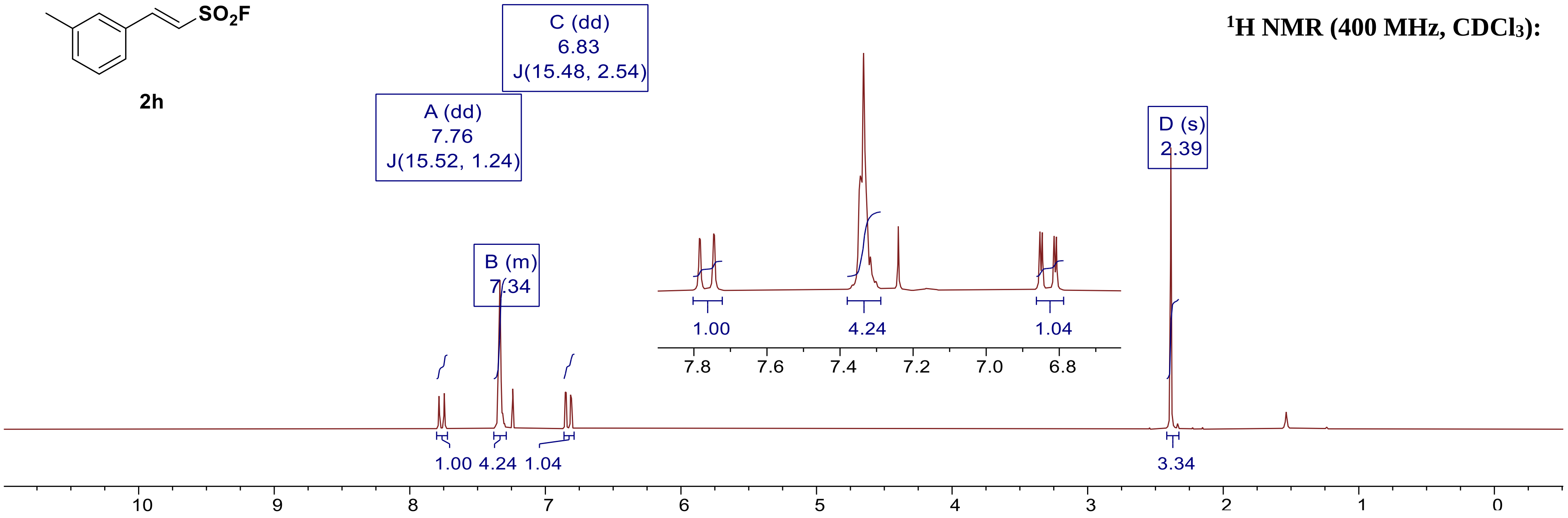
SO2F
2h
1H NMR (400 MHz, CDCl3):
A (dd)
7.76
J(15.52, 1.24)
B (m)
7.34
C (dd)
6.83
J(15.48, 2.54)
D (s)
2.39
1.00
4.24
1.04
3.34

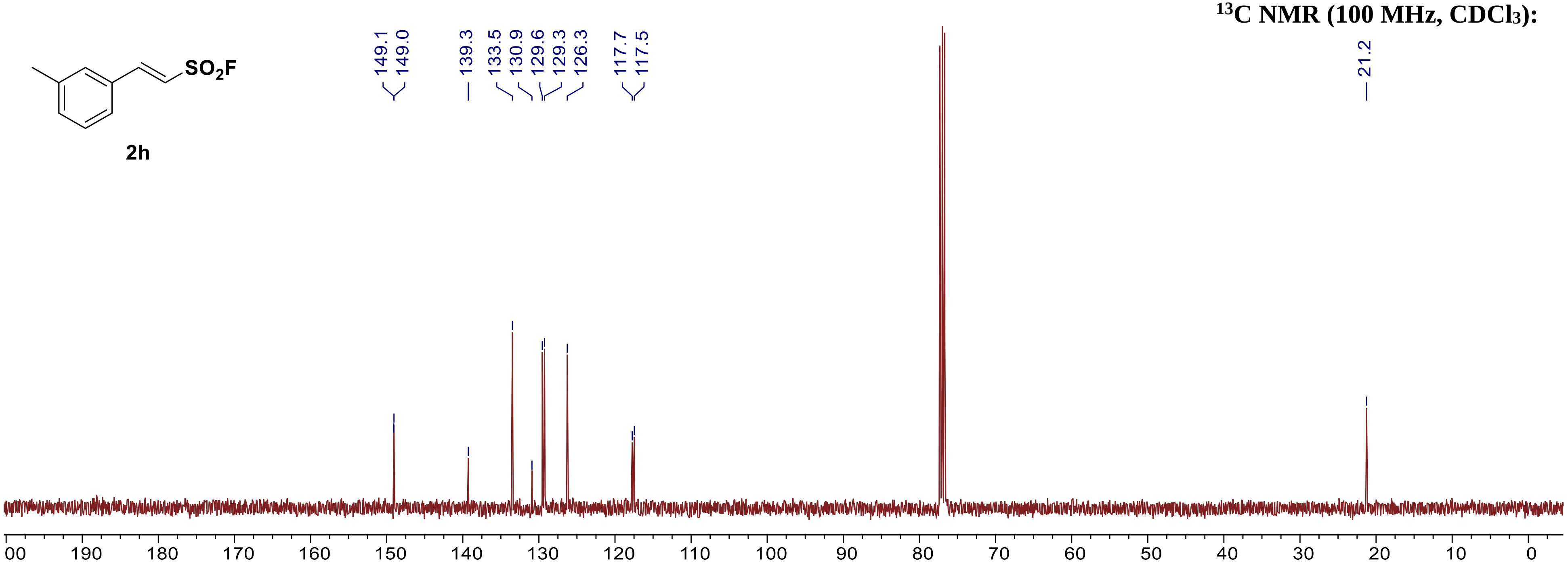
SO2F
2h
13C NMR (100 MHz, CDCl3):
149.1
149.0
139.3
133.5
130.9
129.6
129.3
126.3
117.7
117.5
21.2

^{19}F NMR (376 MHz, CDCl$_3$):

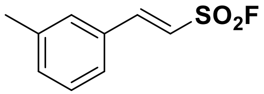

2h

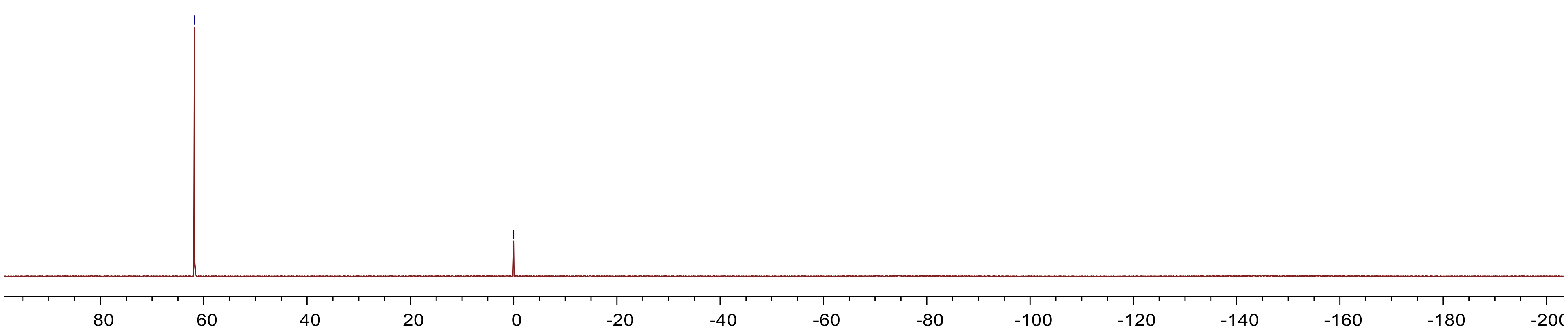

^{1}H NMR (400 MHz, $CDCl_3$):

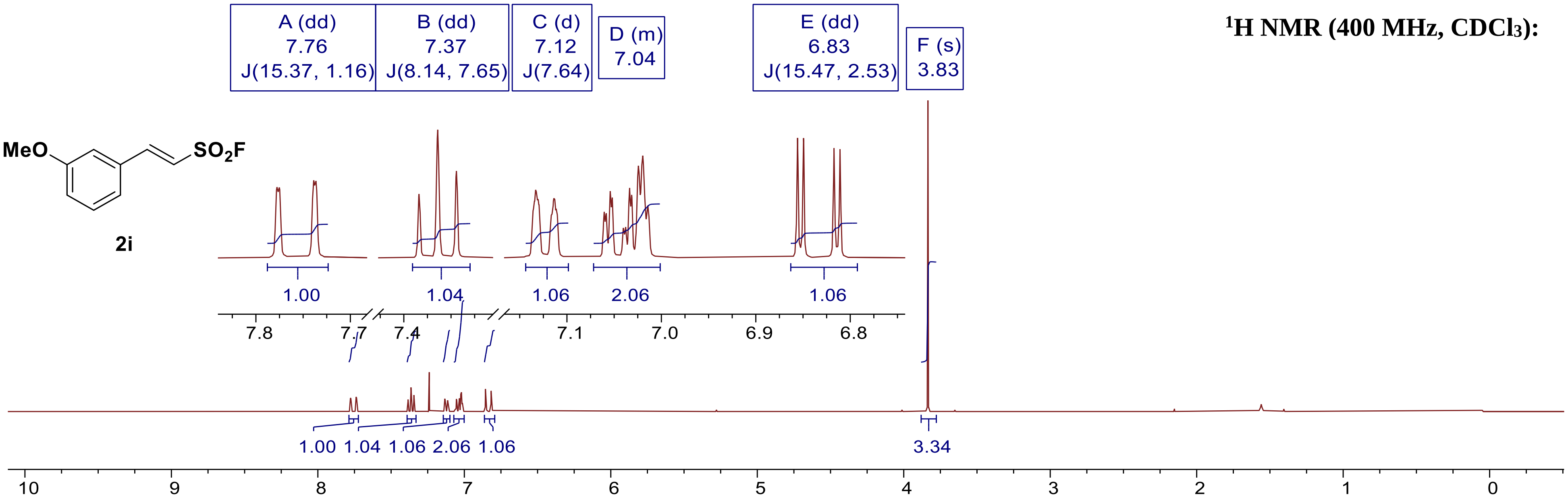

^{13}C NMR (100 MHz, $CDCl_3$):

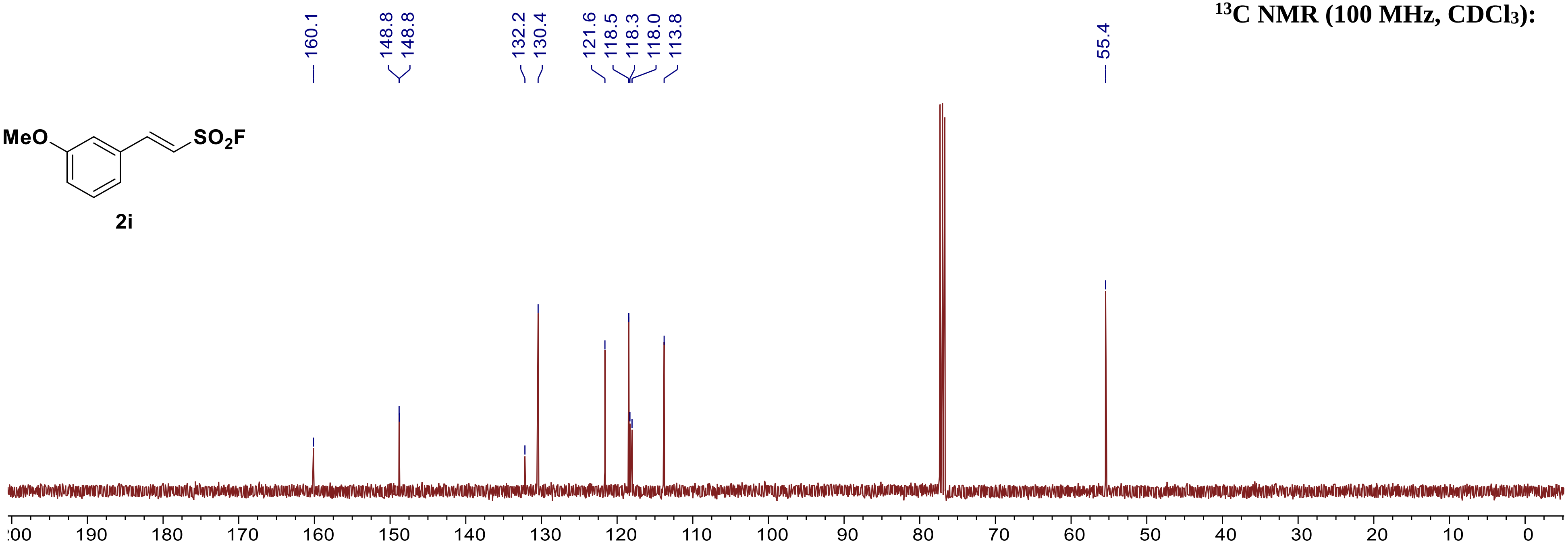

^{19}F NMR (376 MHz, $CDCl_3$):

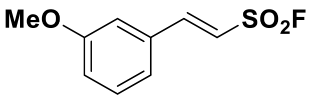

2i

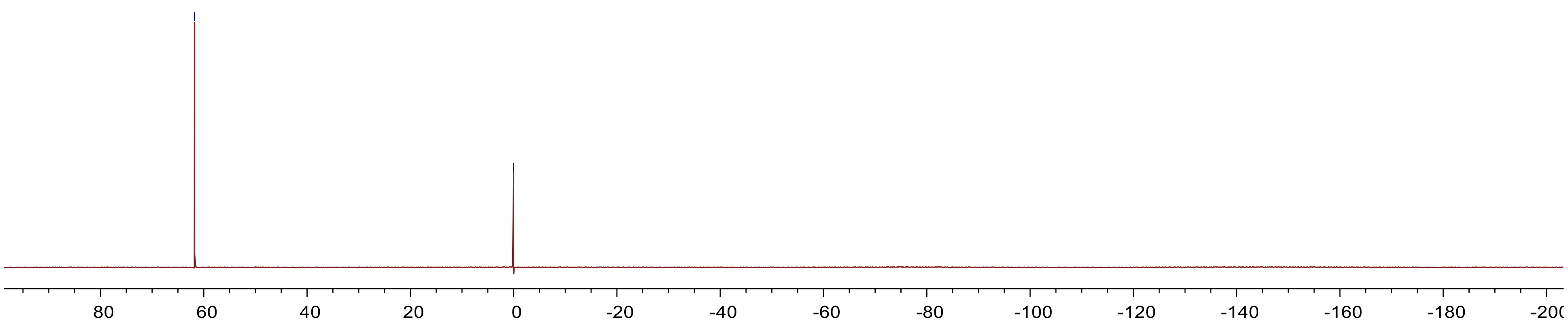

^{1}H NMR (400 MHz, CDCl₃):

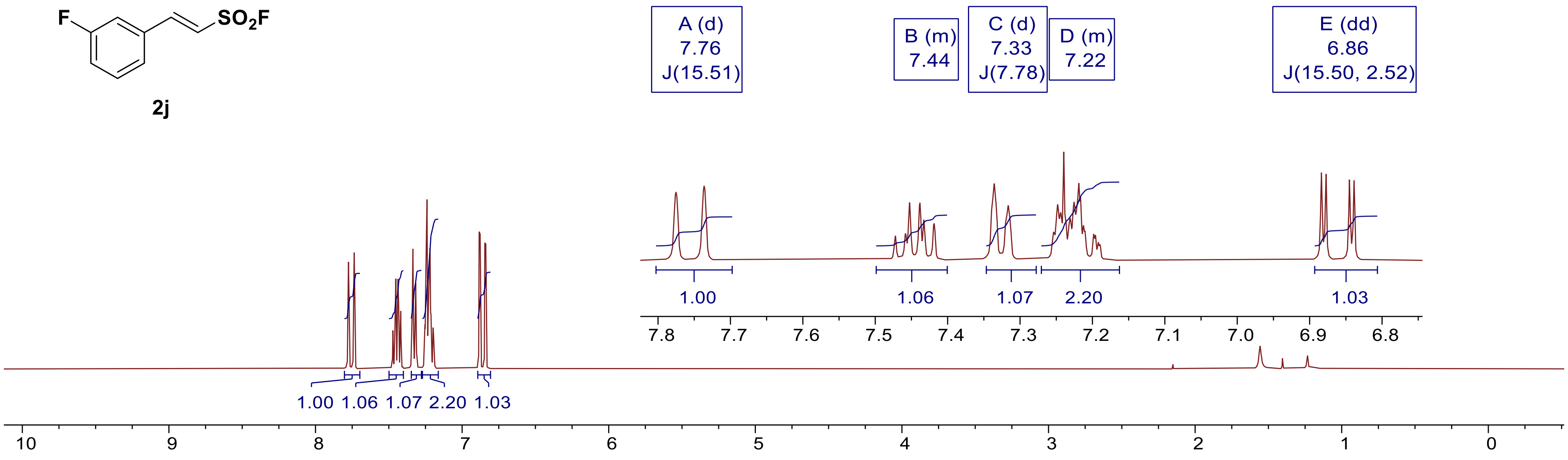

^{13}C NMR (100 MHz, CDCl₃):

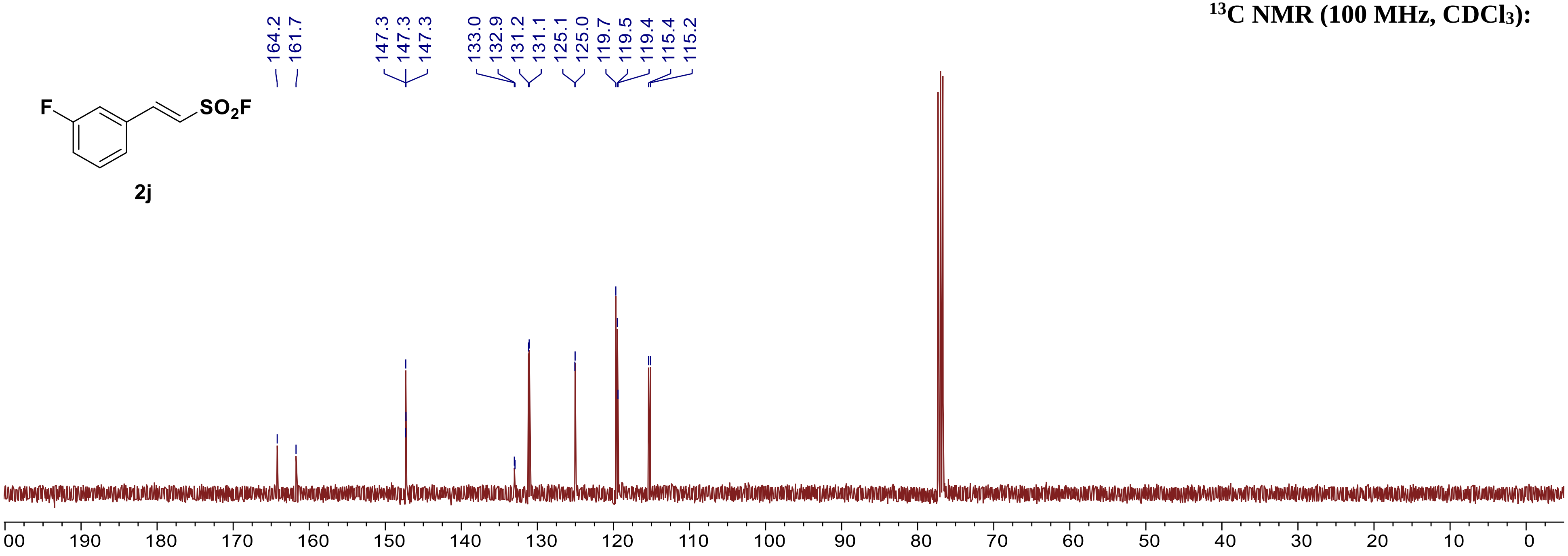

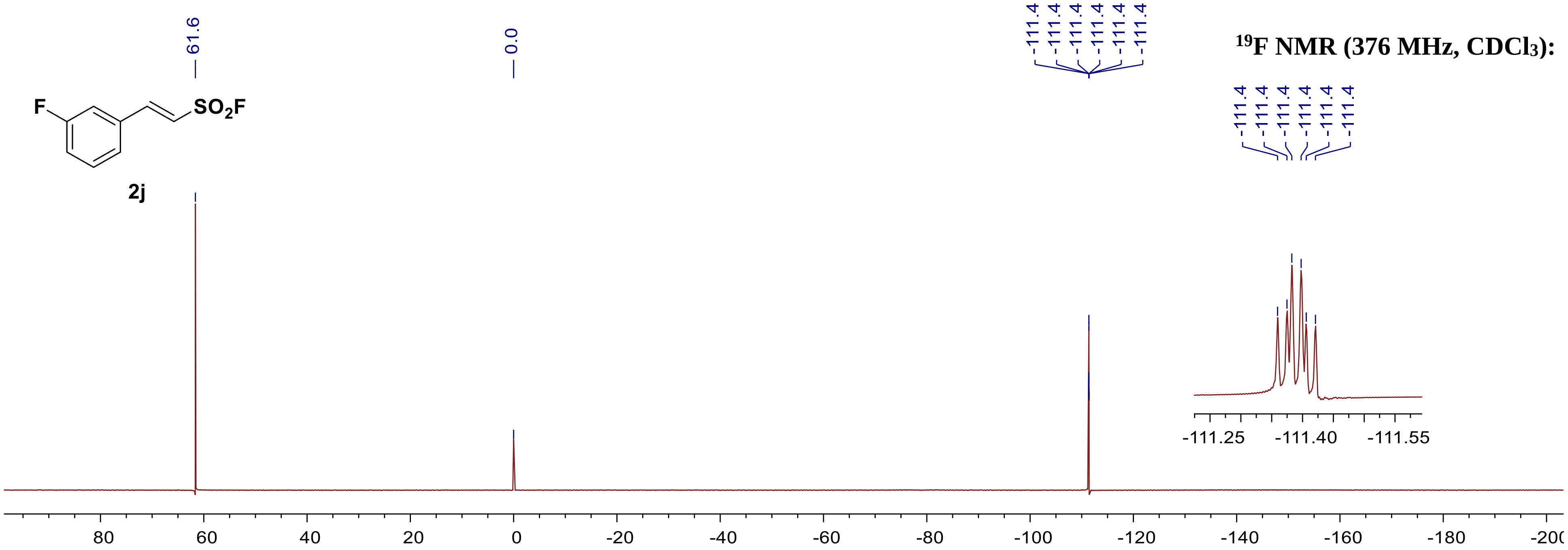

19F NMR (376 MHz, CDCl3):
2j
F
SO2F
61.6
0.0
-111.4
-111.4
-111.4
-111.4
-111.4
-111.4
-111.25
-111.40
-111.55
80
60
40
20
0
-20
-40
-60
-80
-100
-120
-140
-160
-180

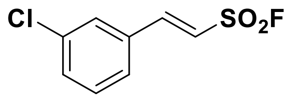

2k

¹H NMR (400 MHz, CDCl₃):

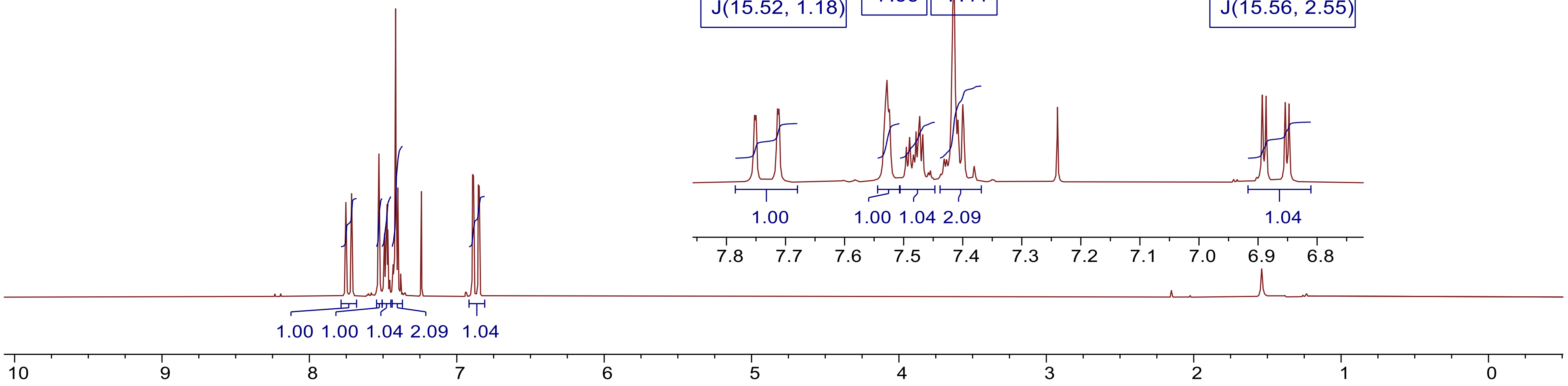

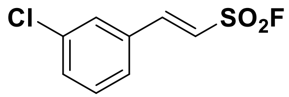

2k

¹³C NMR (100 MHz, CDCl₃):

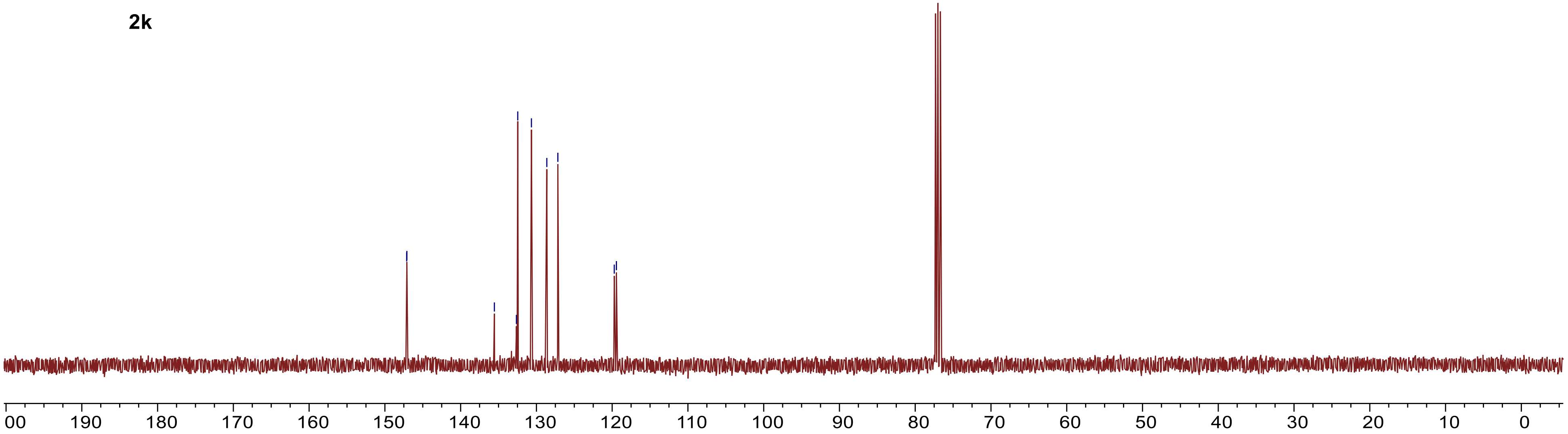

^{19}F NMR (376 MHz, $CDCl_3$):

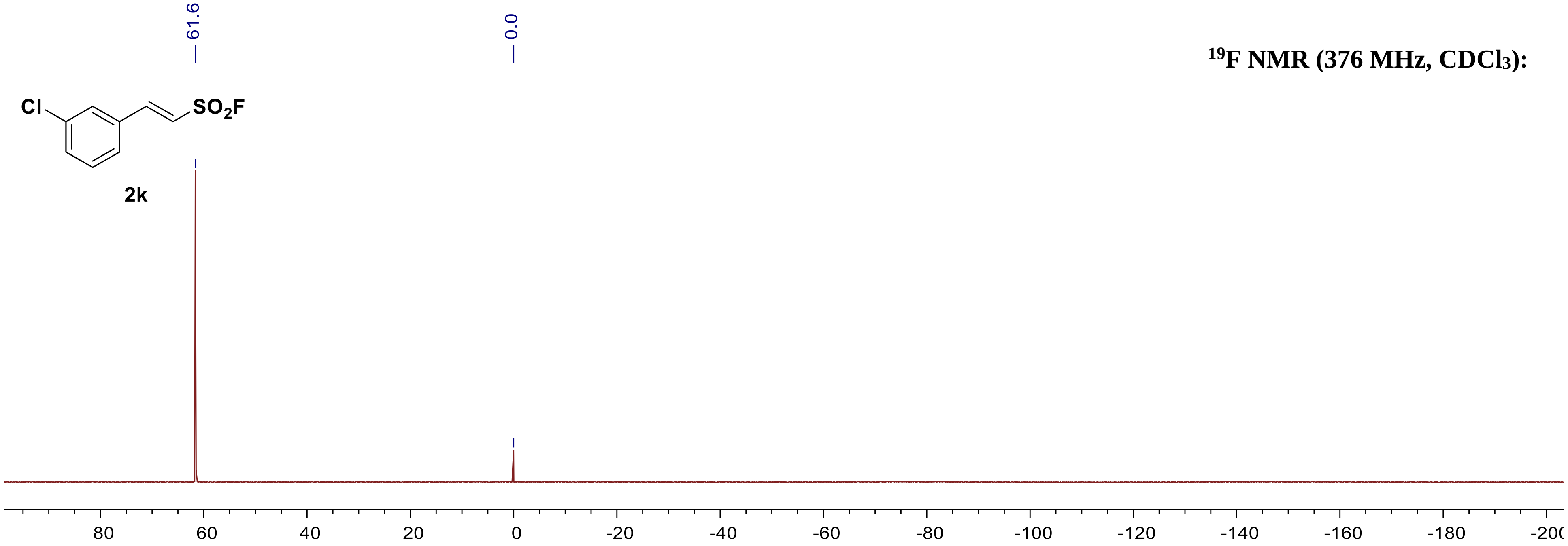

^{1}H NMR (400 MHz, CDCl3):

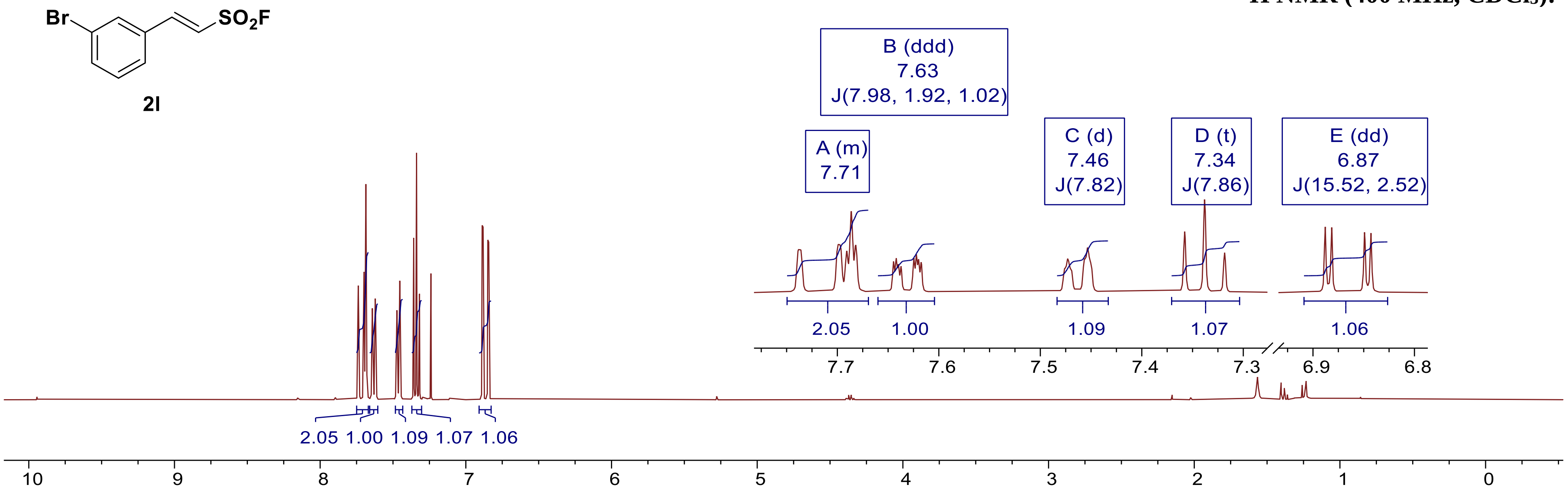

^{13}C NMR (100 MHz, CDCl3):

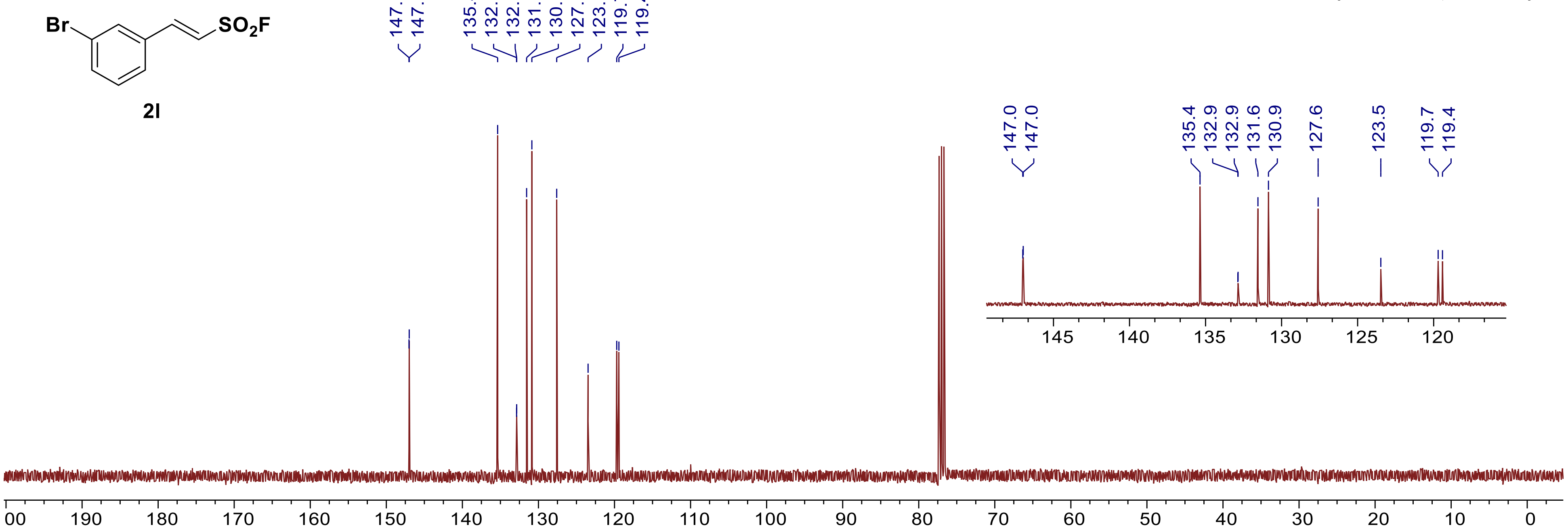

^{19}F NMR (376 MHz, CDCl$_3$):

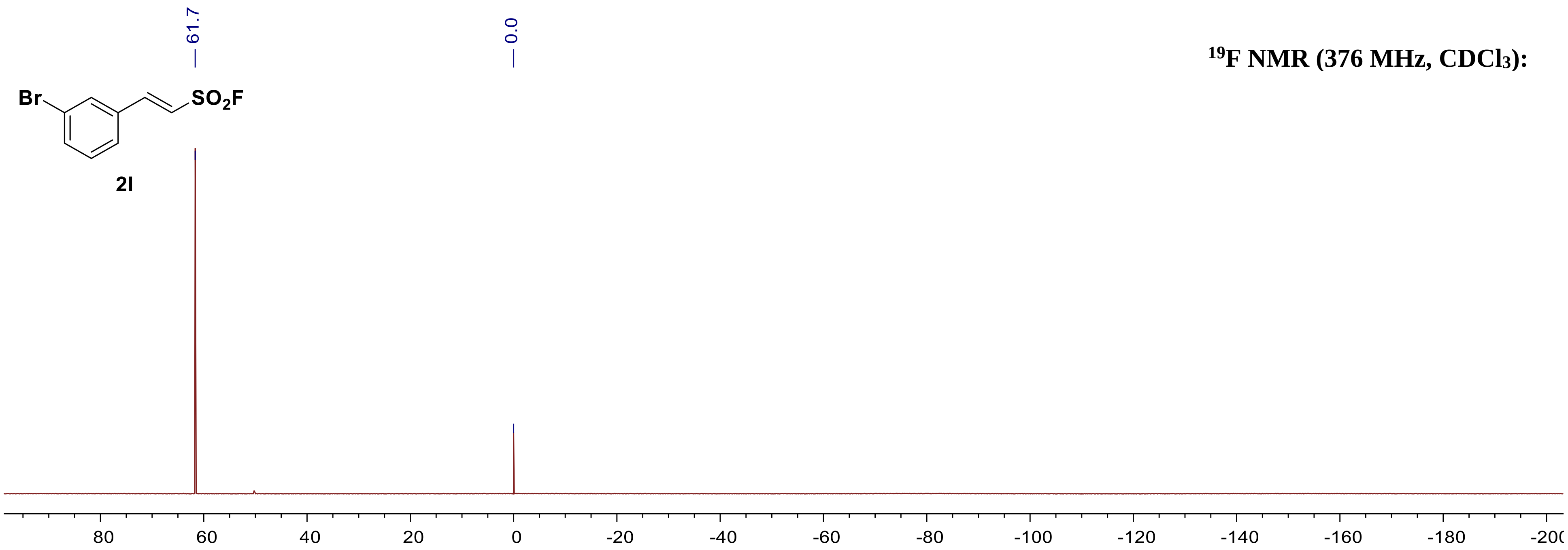

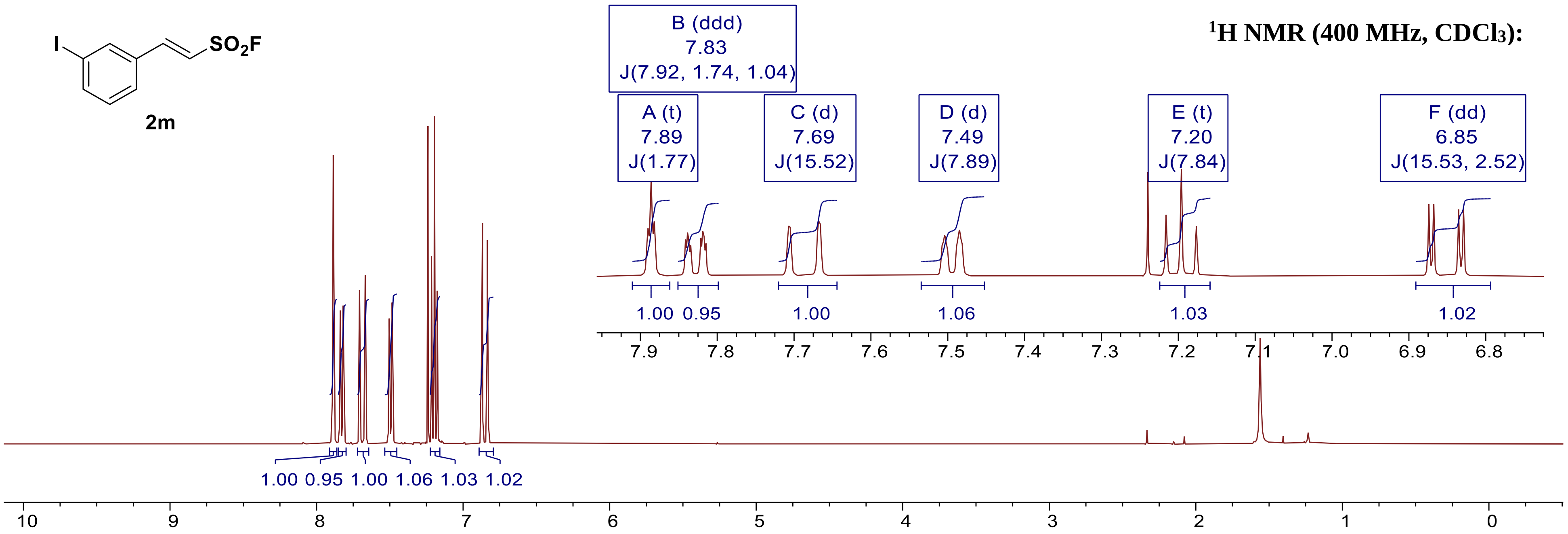

2m
1H NMR (400 MHz, CDCl3):
B (ddd)
7.83
J(7.92, 1.74, 1.04)
A (t)
7.89
J(1.77)
C (d)
7.69
J(15.52)
D (d)
7.49
J(7.89)
E (t)
7.20
J(7.84)
F (dd)
6.85
J(15.53, 2.52)
1.00 0.95 1.00 1.06 1.03 1.02

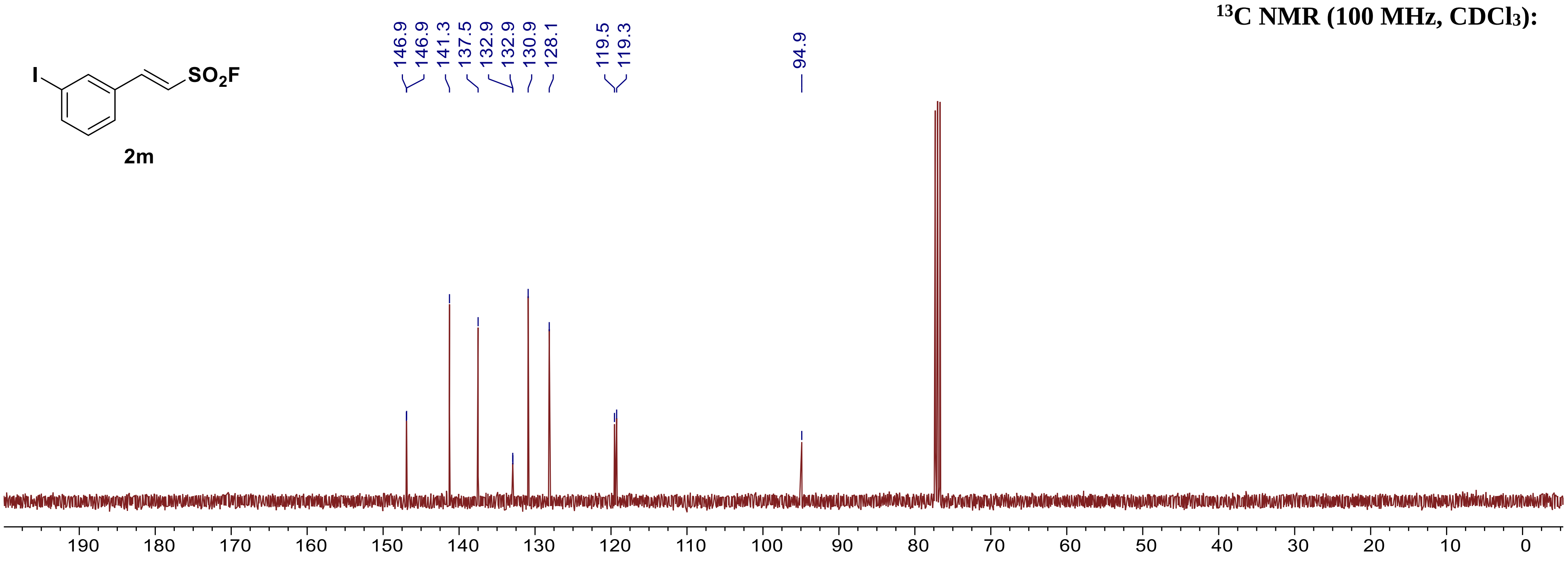

13C NMR (100 MHz, CDCl3):
146.9
146.9
141.3
137.5
132.9
132.9
130.9
128.1
119.5
119.3
94.9
2m

^{19}F NMR (376 MHz, $CDCl_3$):

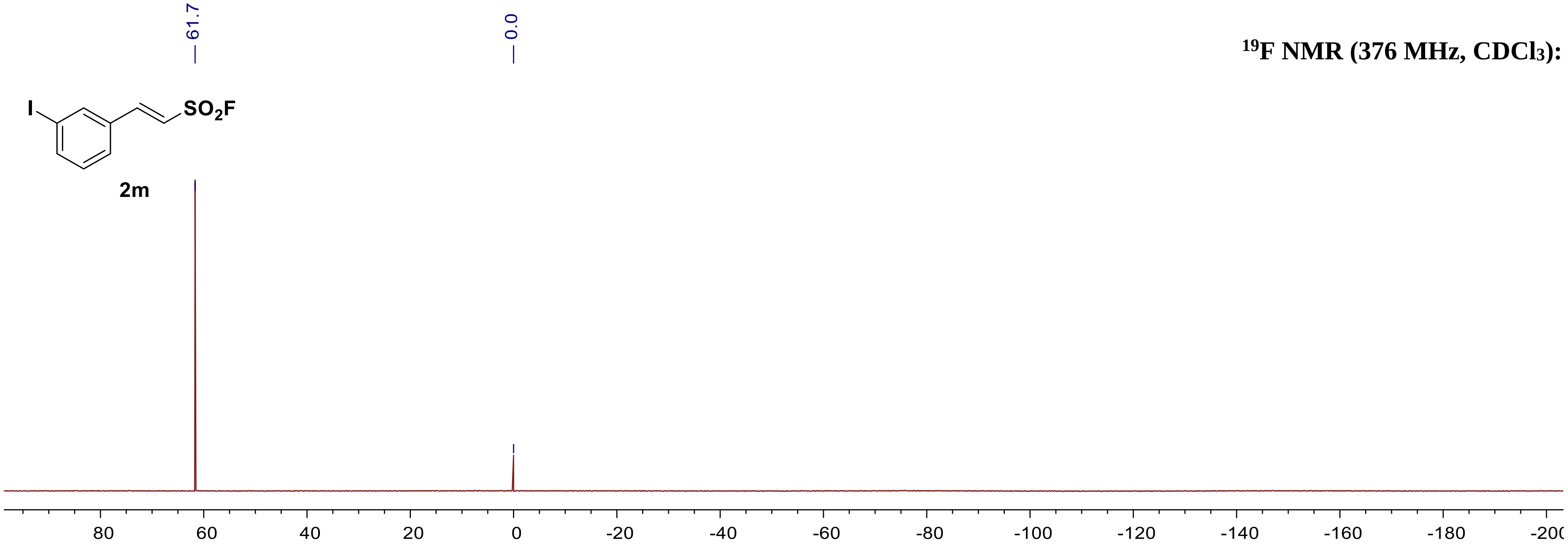

^{1}H NMR (400 MHz, $CDCl_3$):

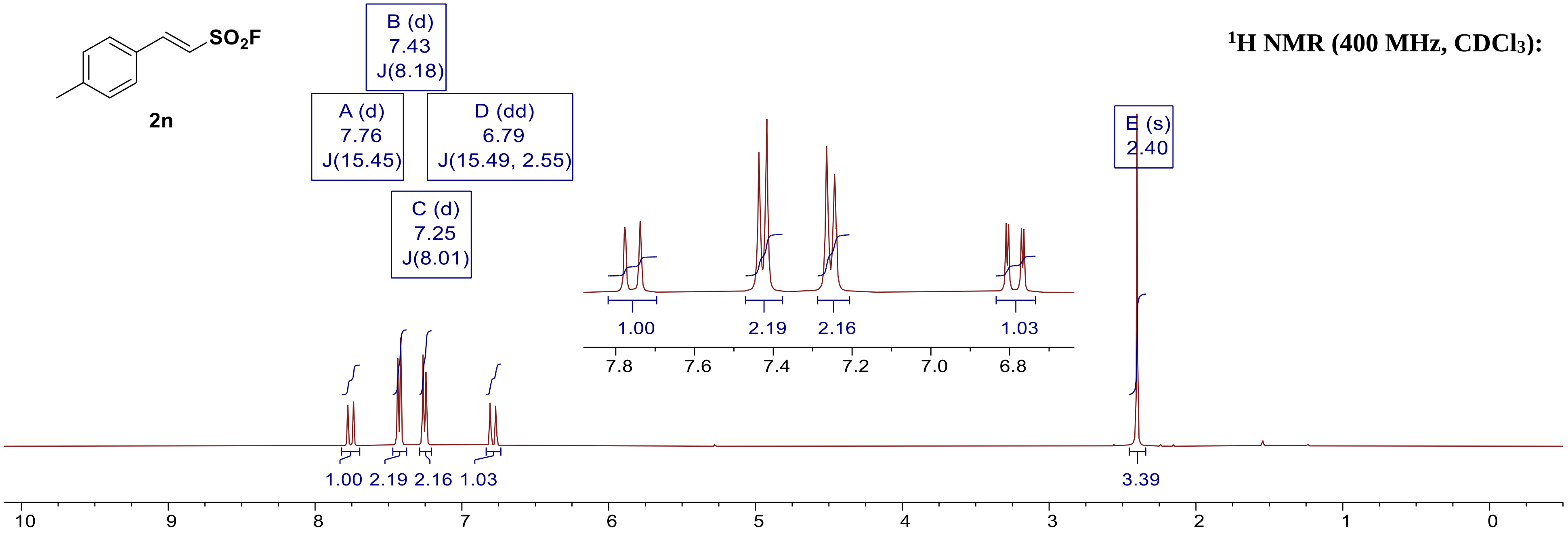

^{13}C NMR (100 MHz, $CDCl_3$):

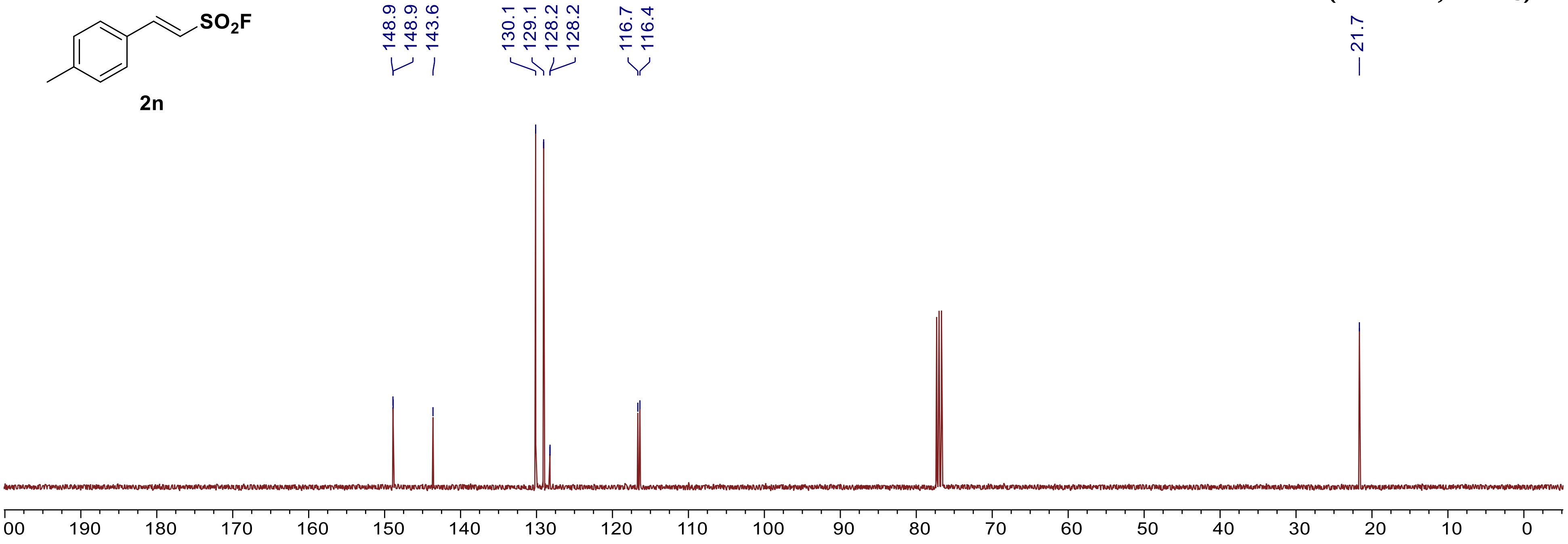

^{19}F NMR (376 MHz, $CDCl_3$):

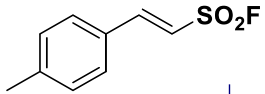

2n

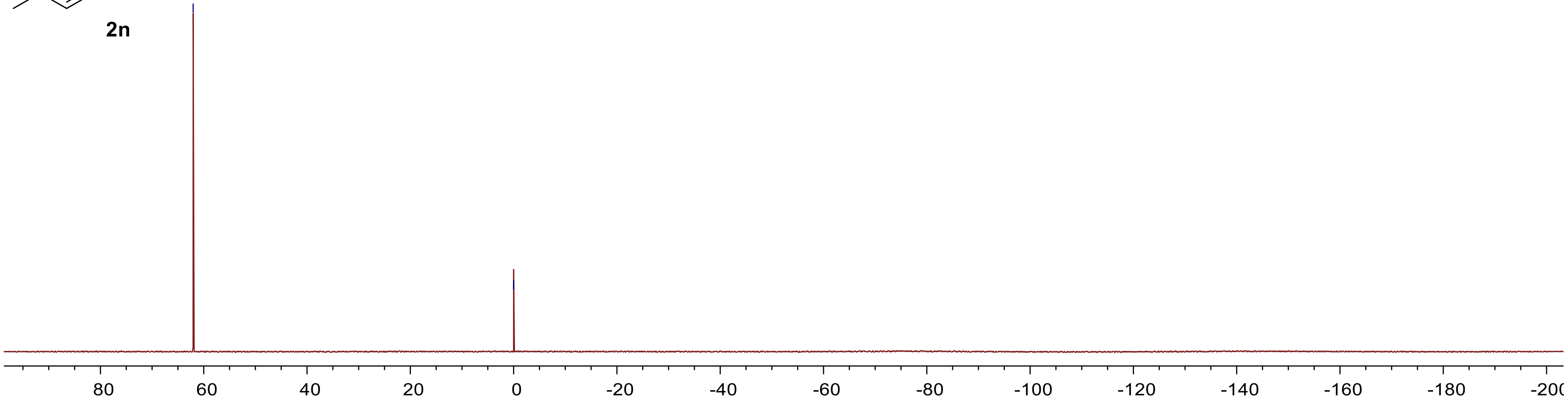

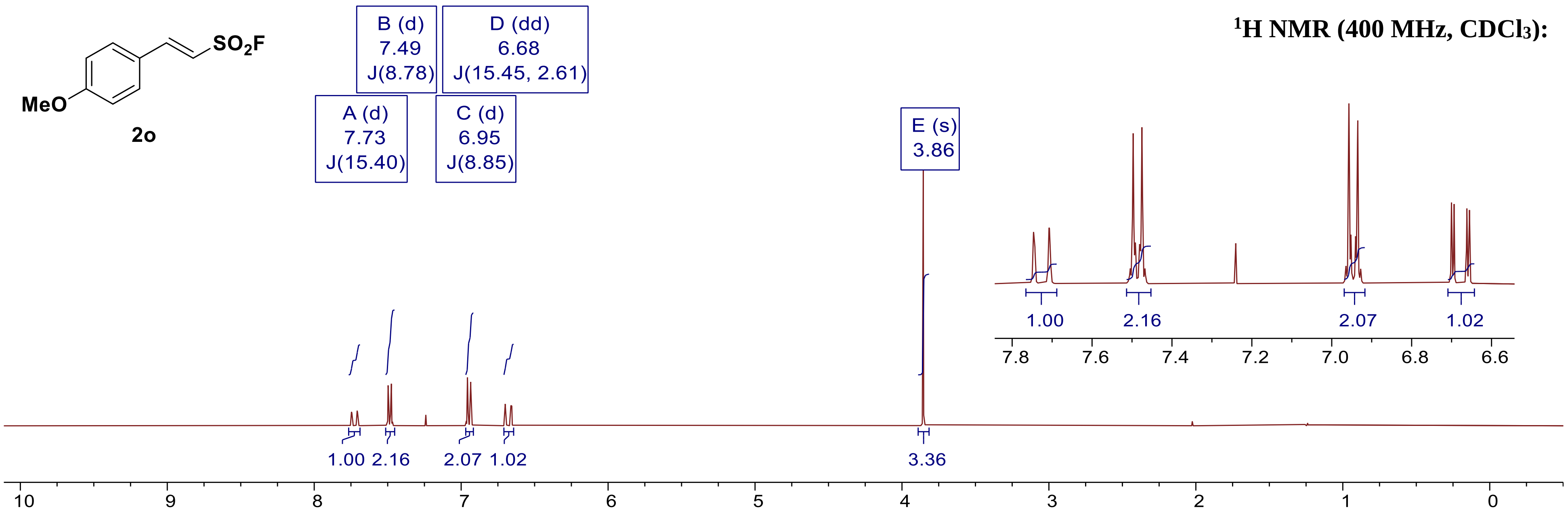
1H NMR (400 MHz, CDCl3):
SO2F
MeO
2o
A (d) 7.73 J(15.40)
B (d) 7.49 J(8.78)
C (d) 6.95 J(8.85)
D (dd) 6.68 J(15.45, 2.61)
E (s) 3.86
1.00 2.16 2.07 1.02 3.36
10 9 8 7 6 5 4 3 2 1 0
1.00 2.16 2.07 1.02
7.8 7.6 7.4 7.2 7.0 6.8 6.6

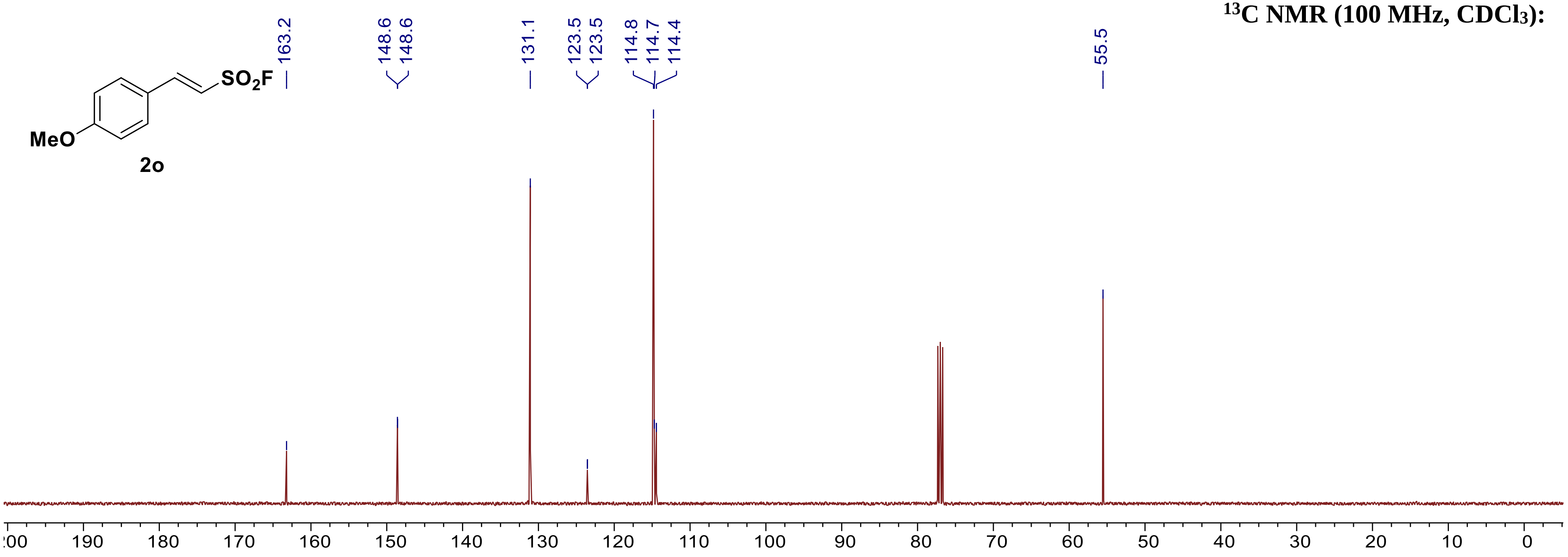
13C NMR (100 MHz, CDCl3):
SO2F
MeO
2o
163.2
148.6
148.6
131.1
123.5
123.5
114.8
114.7
114.4
55.5
200 190 180 170 160 150 140 130 120 110 100 90 80 70 60 50 40 30 20 10 0

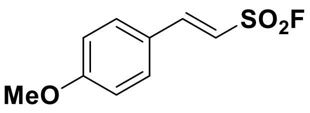

^{19}F NMR (376 MHz, $CDCl_3$):

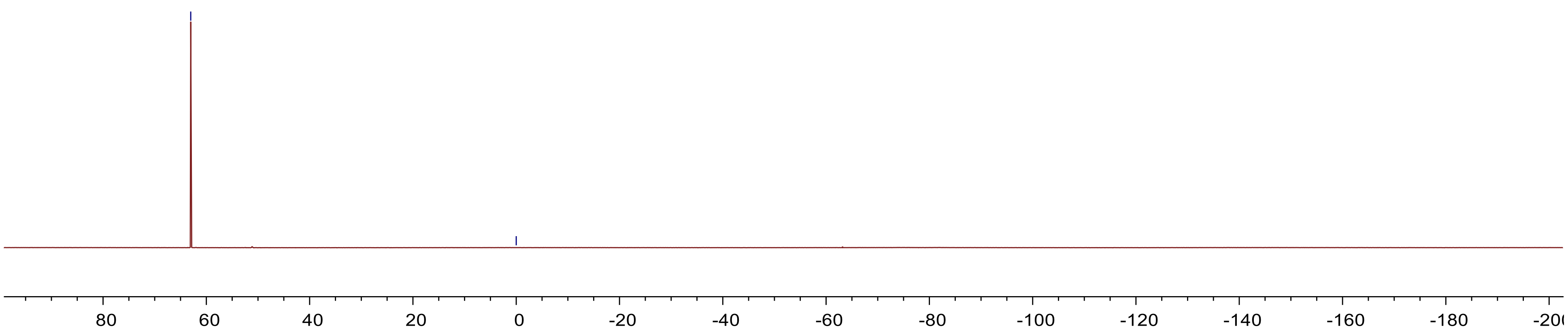

^{1}H NMR (400 MHz, $CDCl_3$):

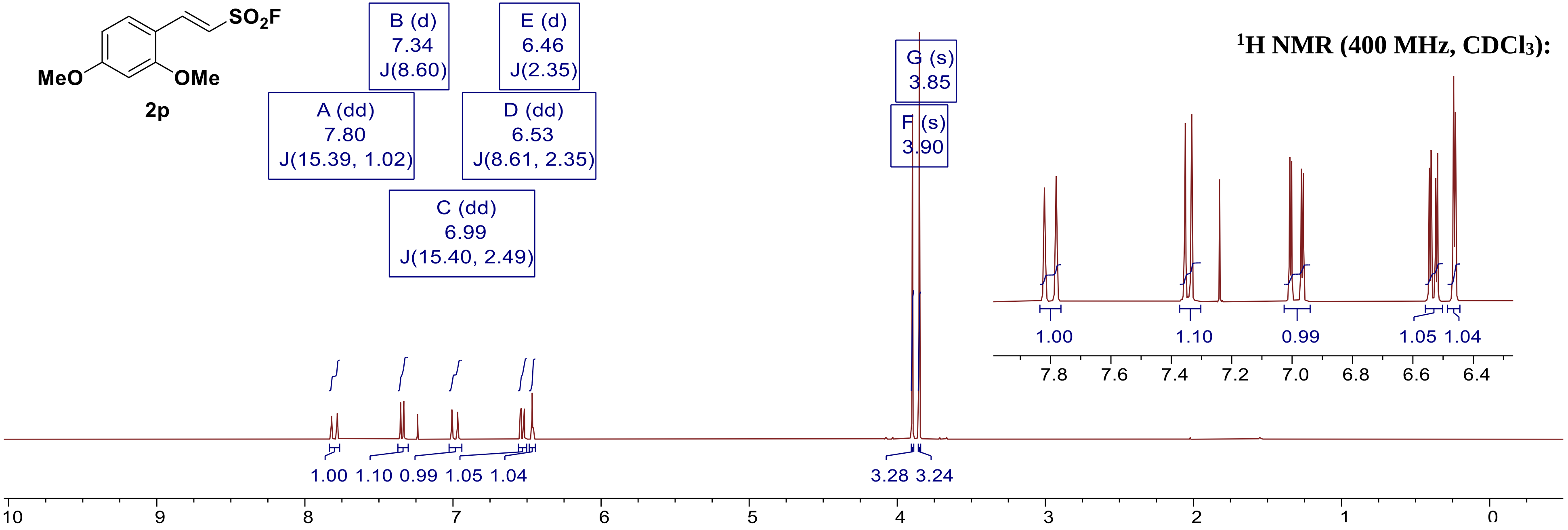

^{13}C NMR (100 MHz, $CDCl_3$):

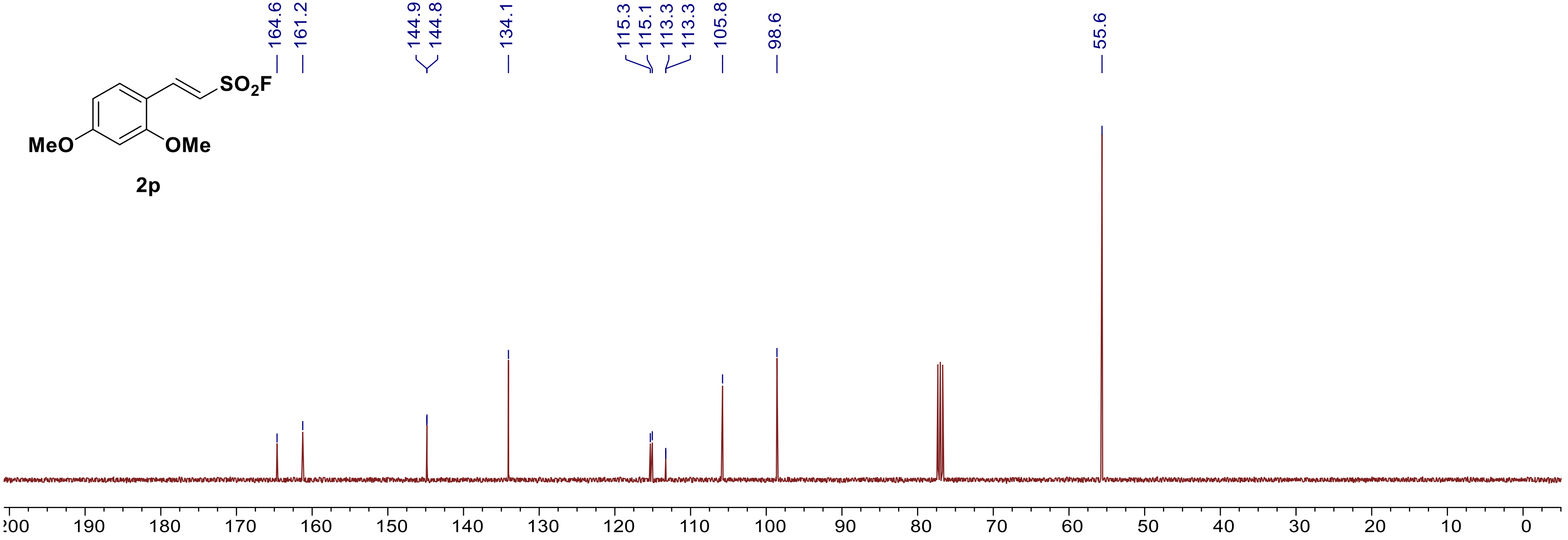

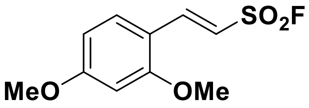

2p

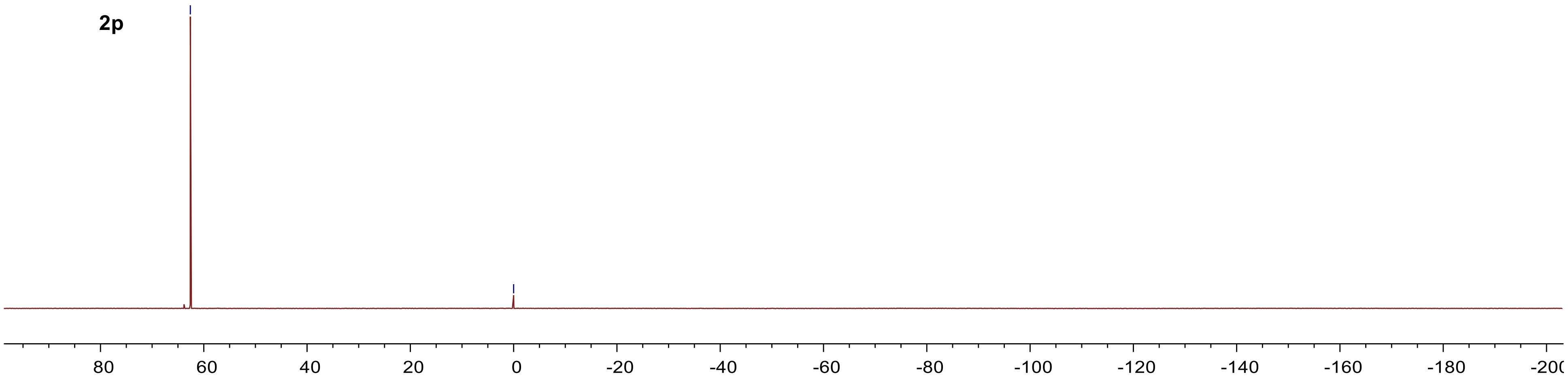

^{19}F NMR (376 MHz, $CDCl_3$):

^{1}H NMR (400 MHz, $CDCl_3$):

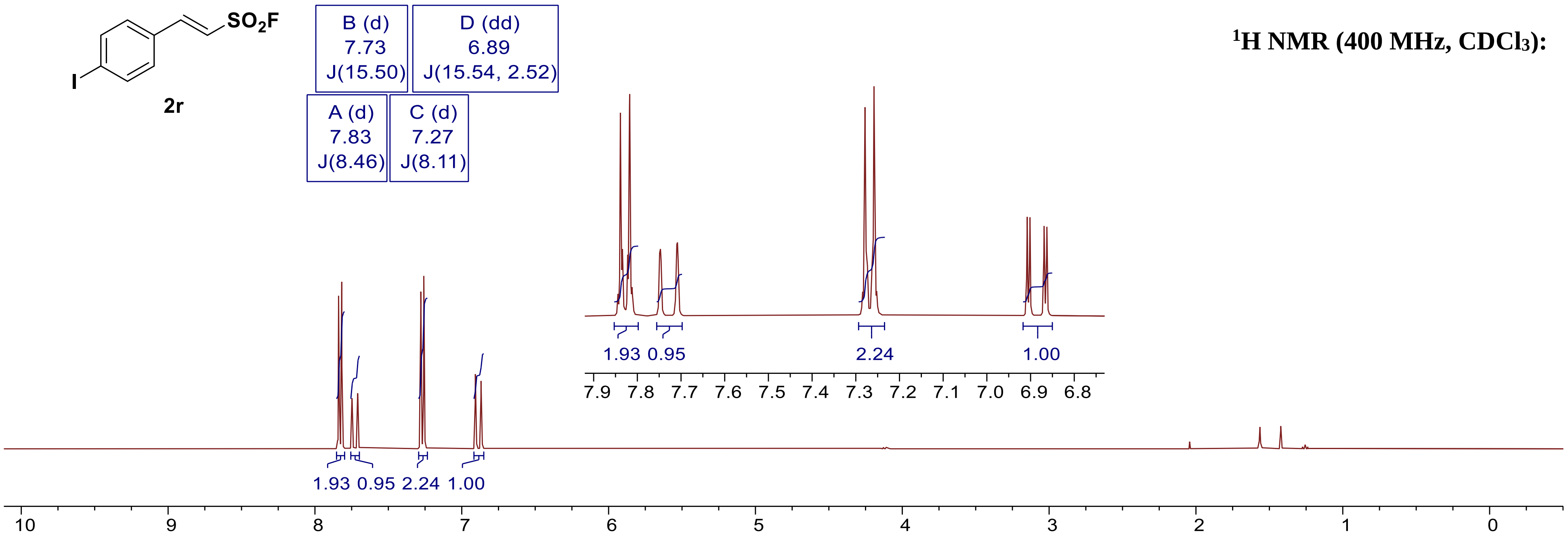

^{13}C NMR (100 MHz, $CDCl_3$):

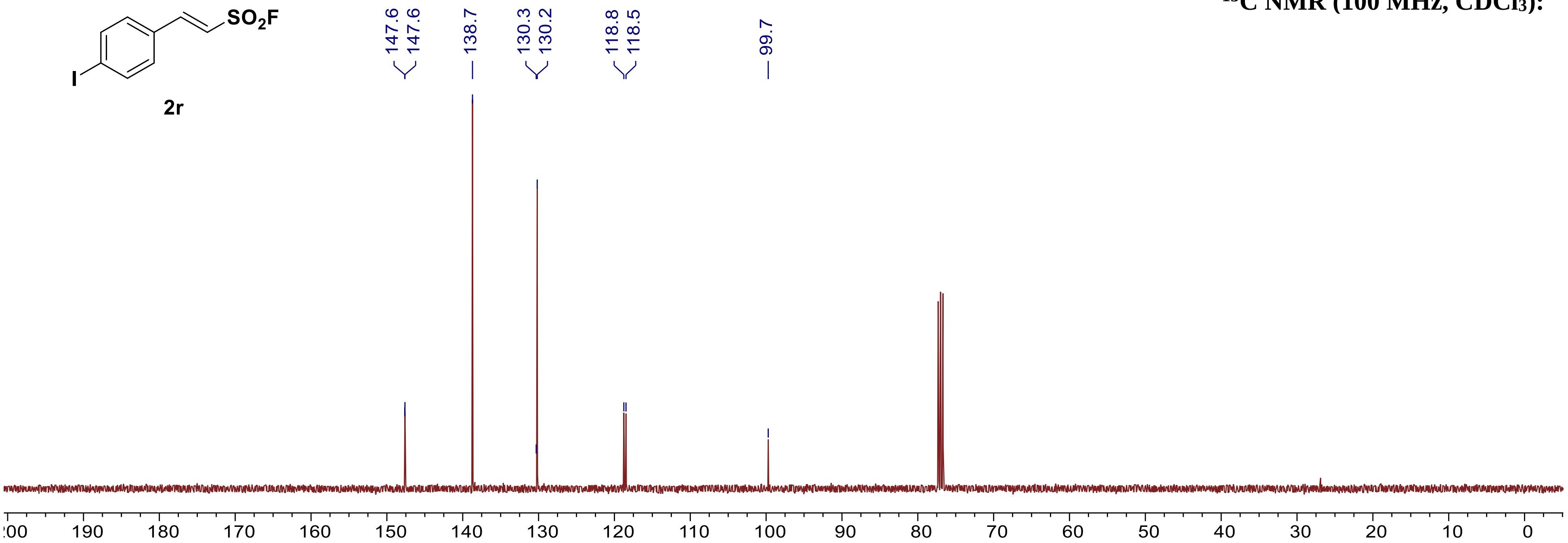

^{19}F NMR (376 MHz, $CDCl_3$):

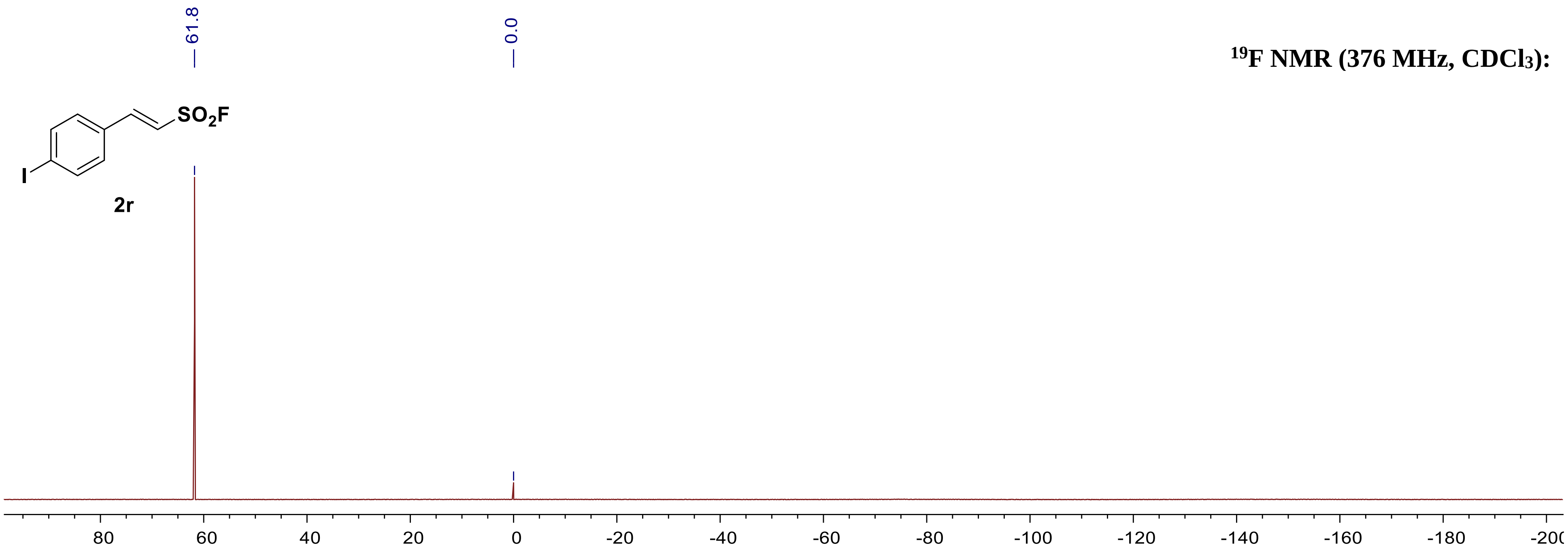

^{1}H NMR (400 MHz, $CDCl_3$):

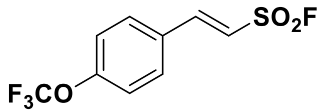

2s

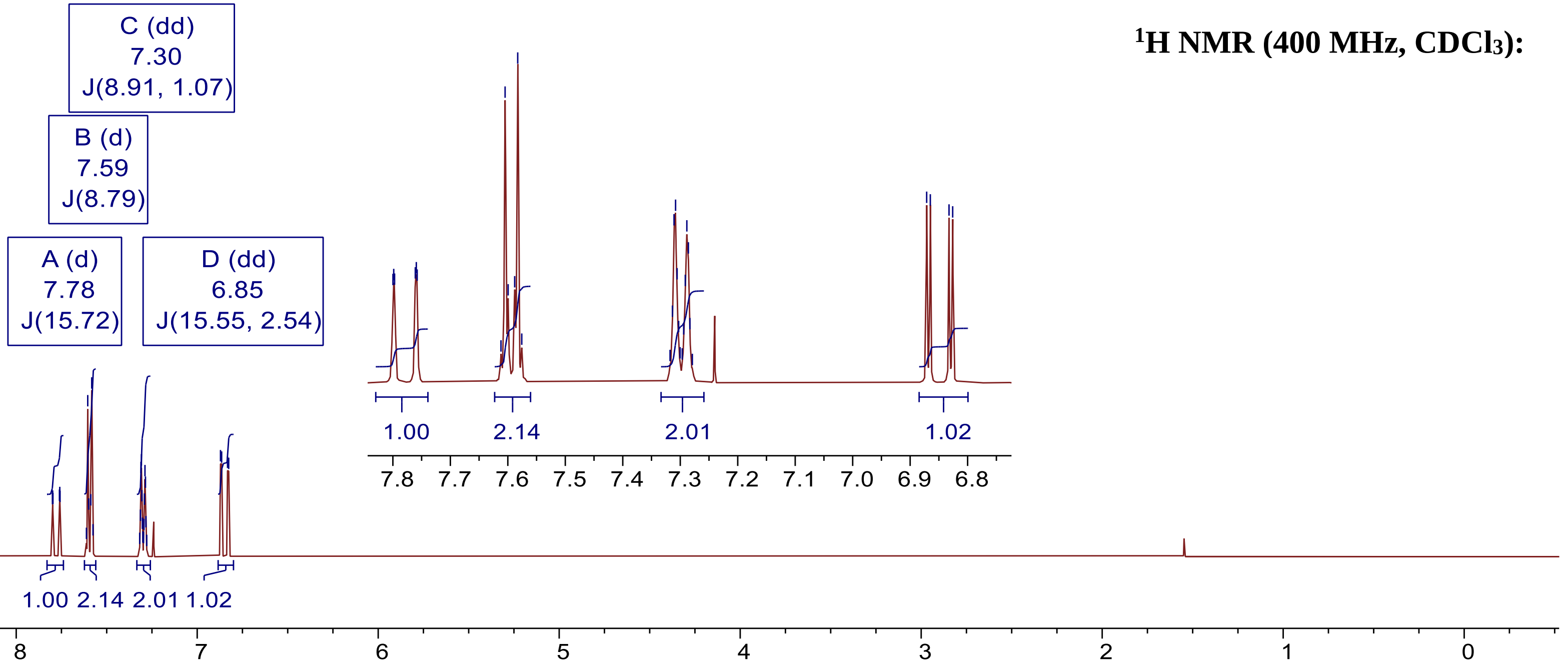

^{13}C NMR (100 MHz, $CDCl_3$):

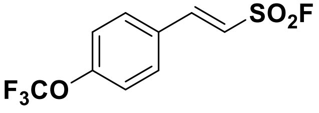

2s

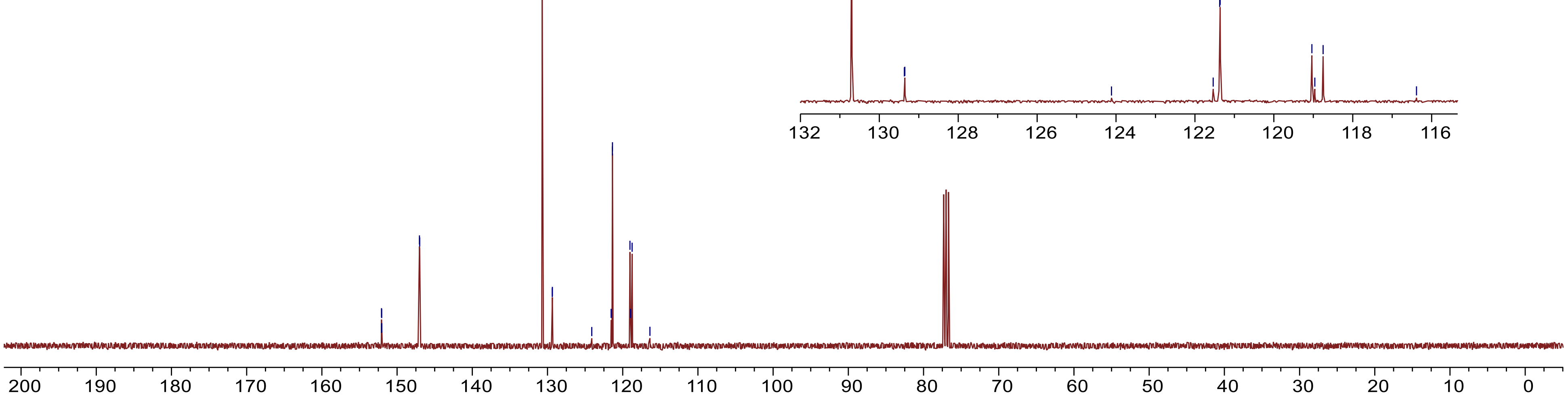

^{19}F NMR (376 MHz, CDCl3):

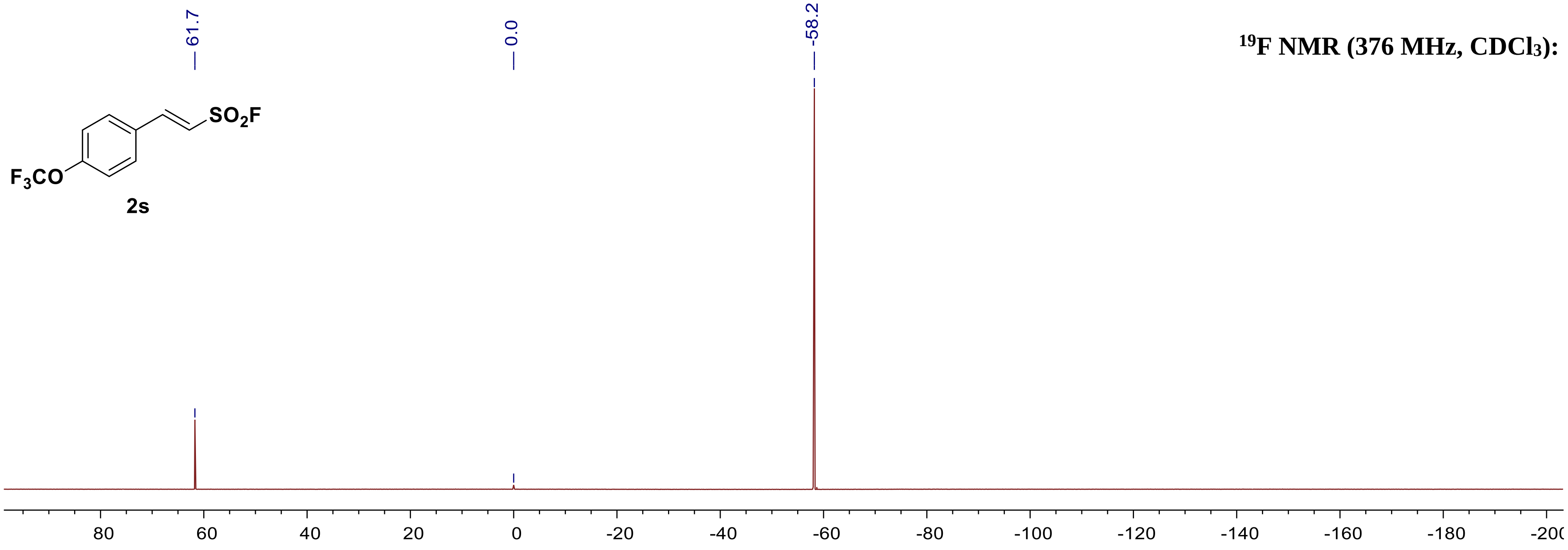

2s

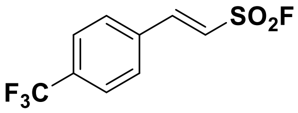

2t

^{1}H NMR (400 MHz, CDCl$_3$):

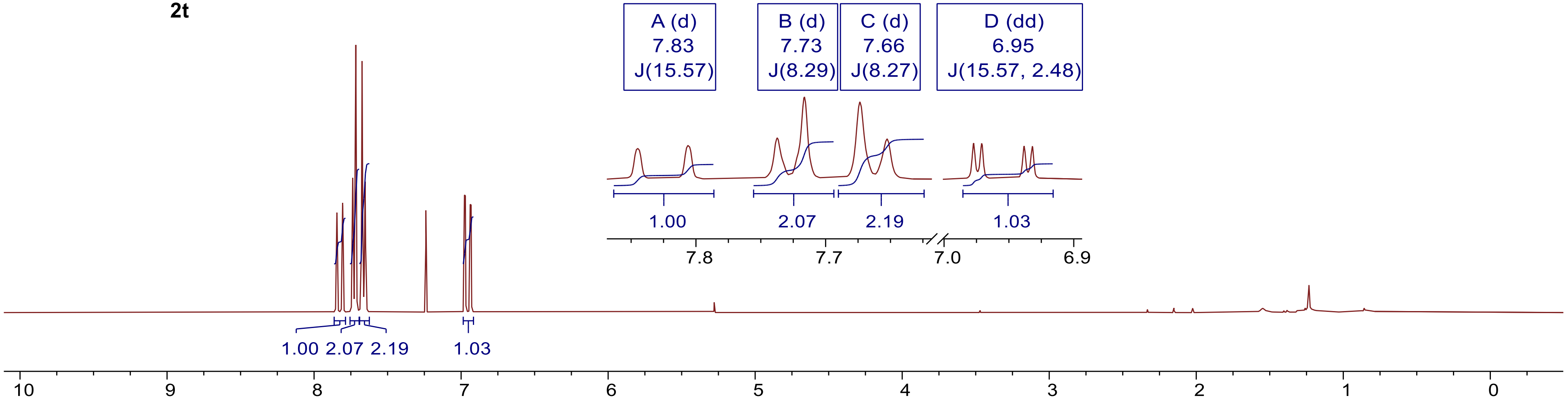

^{13}C NMR (100 MHz, CDCl$_3$):

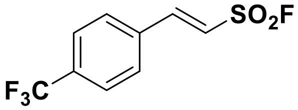

2t

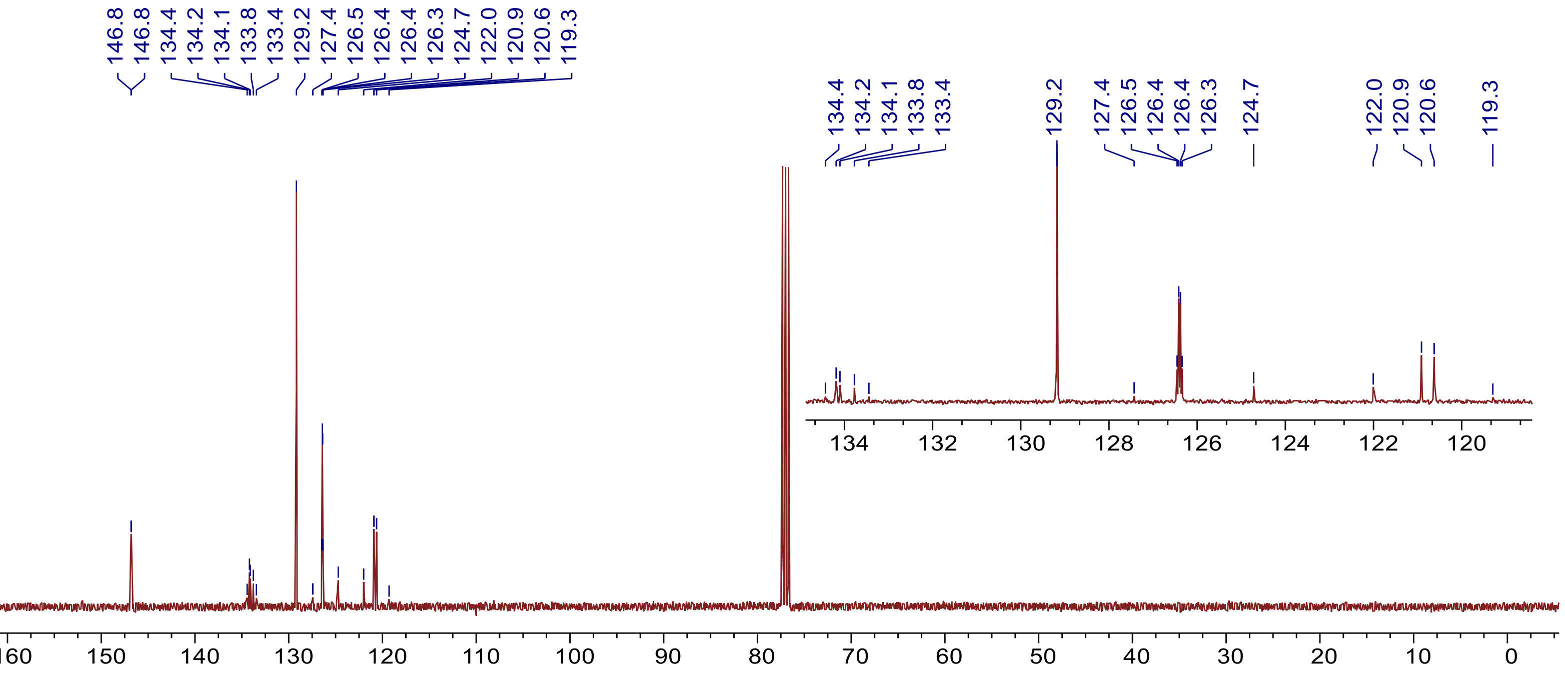

^{19}F NMR (376 MHz, $CDCl_3$):

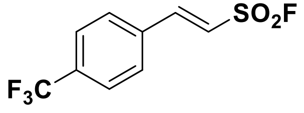

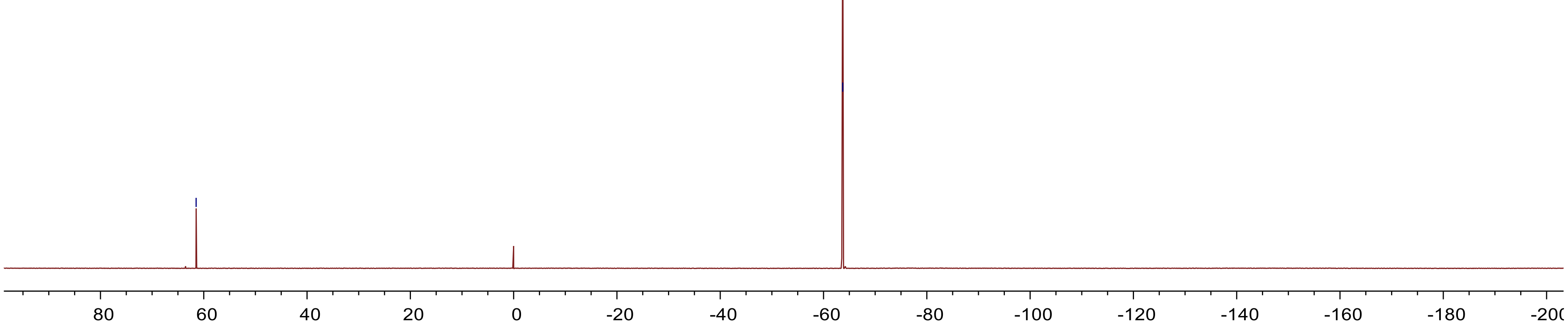

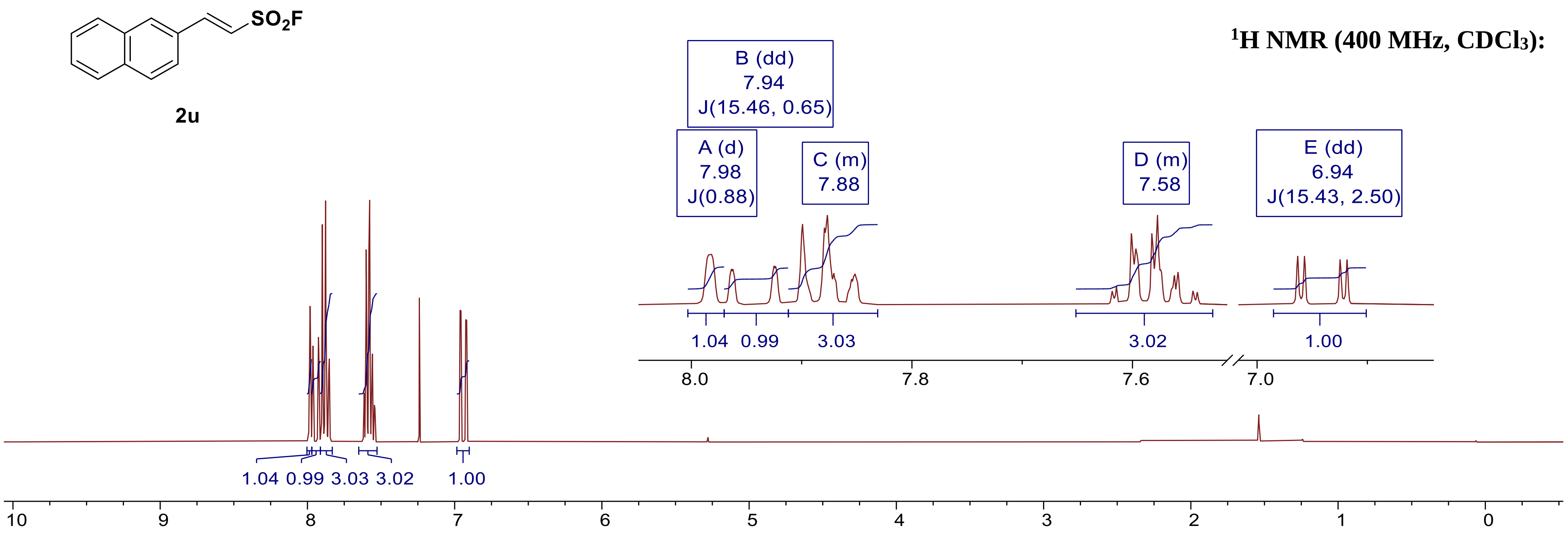
SO2F
2u
1H NMR (400 MHz, CDCl3):
A (d) 7.98 J(0.88)
B (dd) 7.94 J(15.46, 0.65)
C (m) 7.88
D (m) 7.58
E (dd) 6.94 J(15.43, 2.50)
1.04 0.99 3.03 3.02 1.00
8.0 7.8 7.6 7.0
10 9 8 7 6 5 4 3 2 1 0

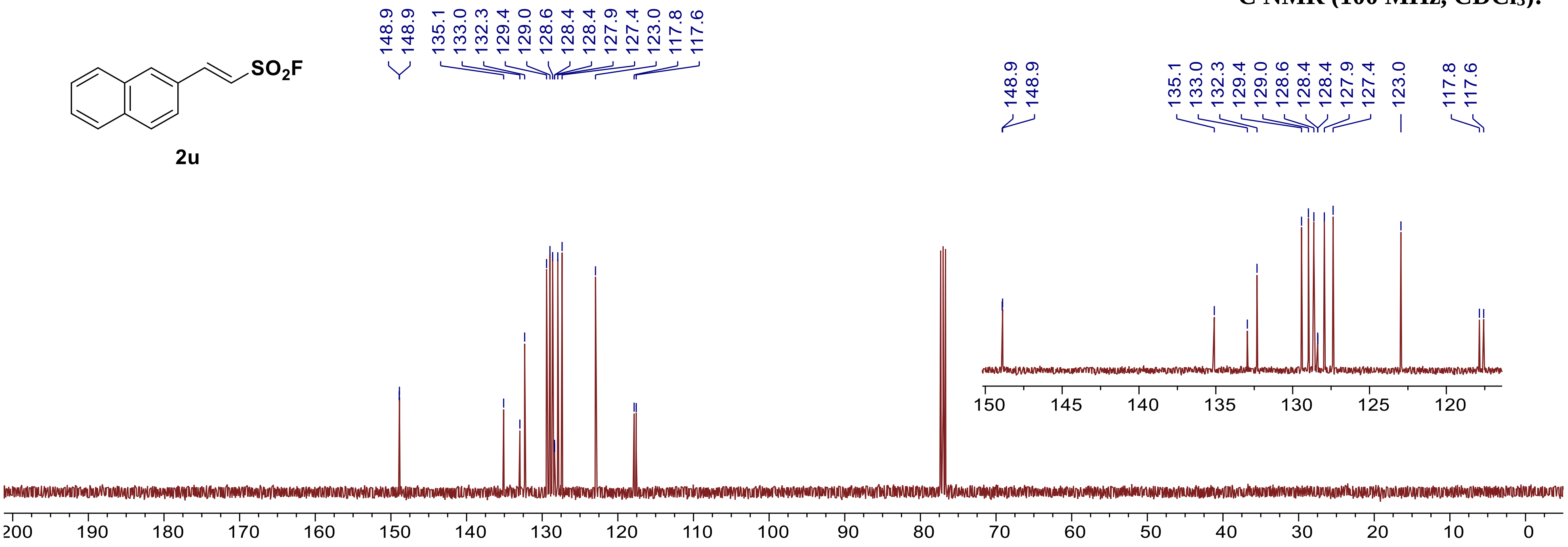
SO2F
2u
13C NMR (100 MHz, CDCl3):
148.9 148.9 135.1 133.0 132.3 129.4 129.0 128.6 128.4 128.4 127.9 127.4 123.0 117.8 117.6
150 145 140 135 130 125 120
200 190 180 170 160 150 140 130 120 110 100 90 80 70 60 50 40 30 20 10 0

^{19}F NMR (376 MHz, CDCl3):

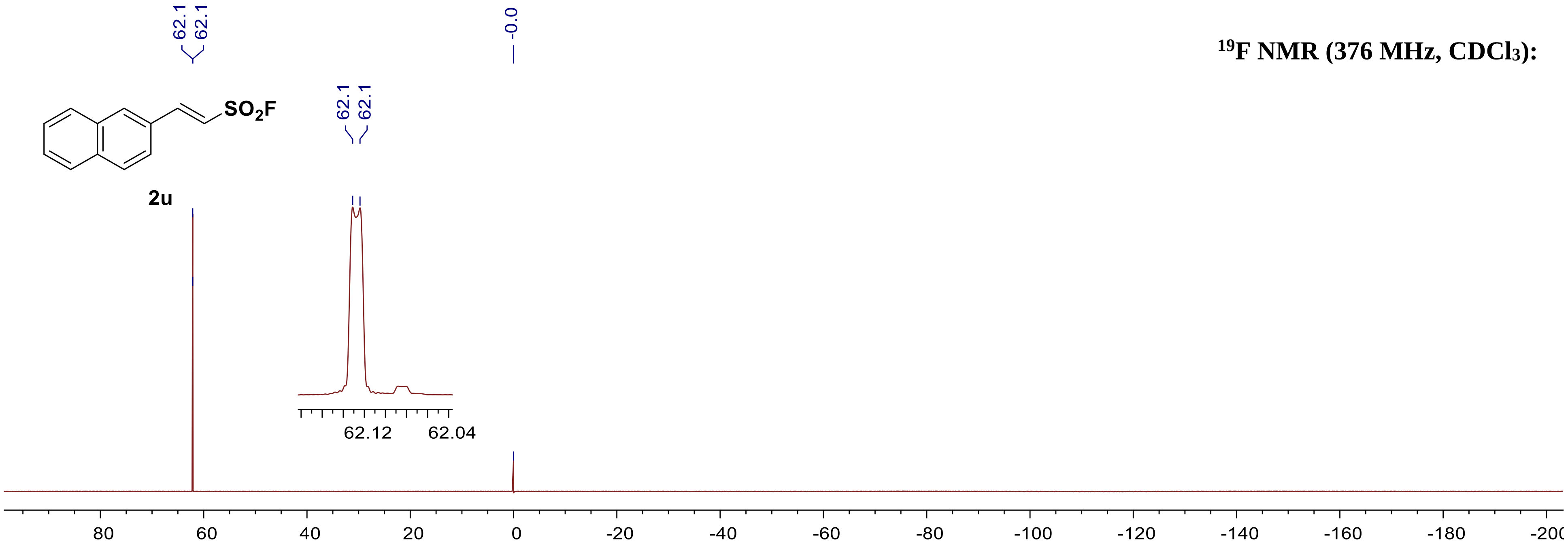

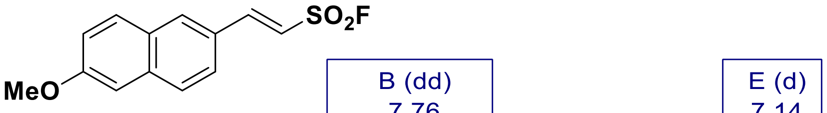

2v

^{1}H NMR (400 MHz, CDCl3):

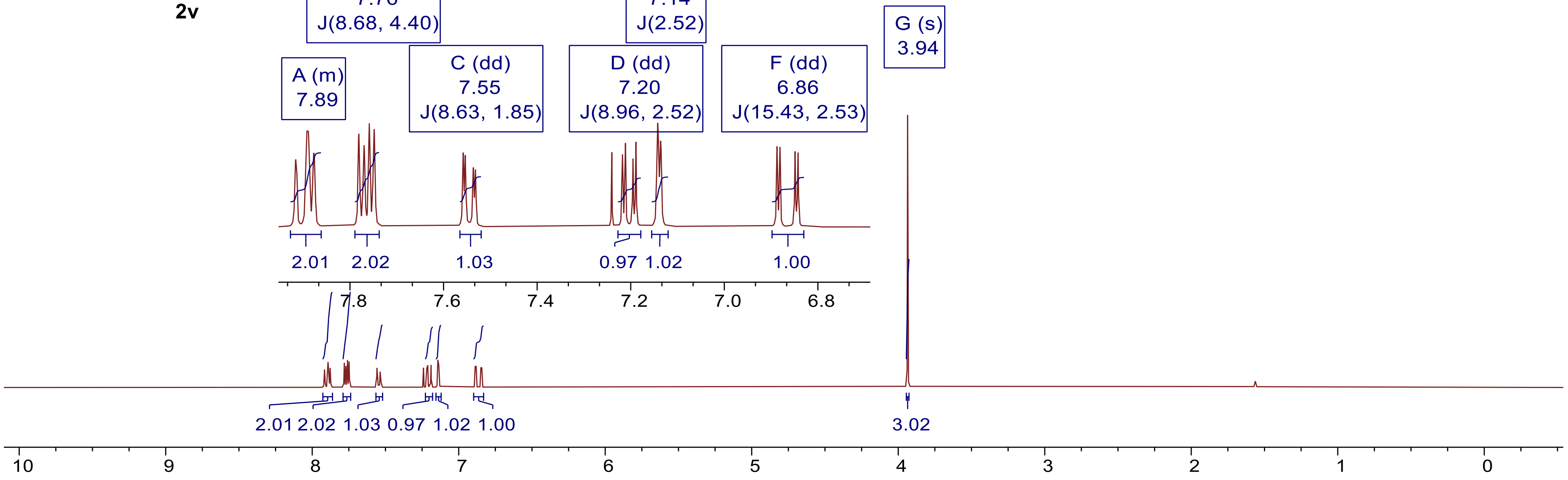

^{13}C NMR (100 MHz, CDCl3):

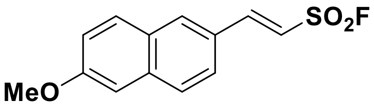

2v

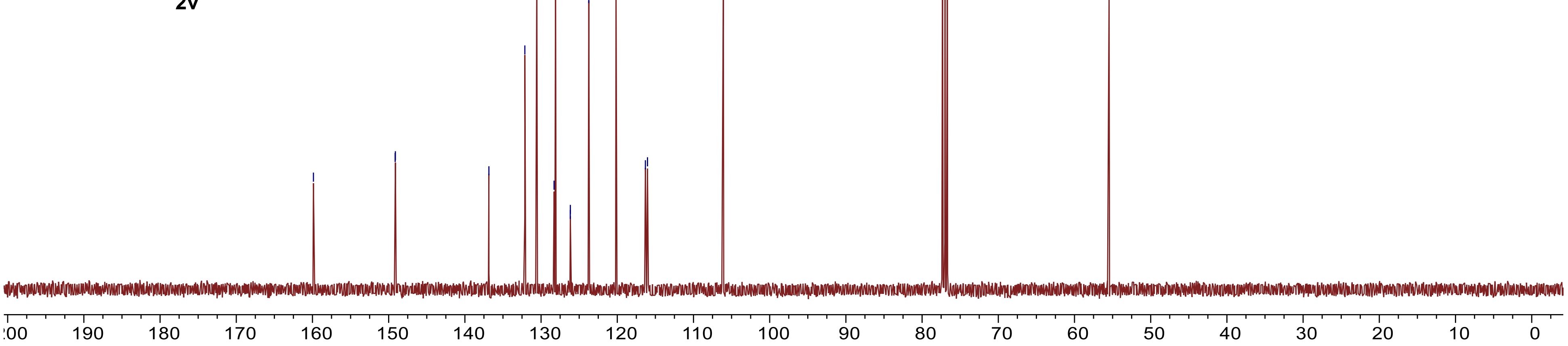

^{19}F NMR (376 MHz, $CDCl_3$):

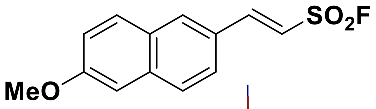

2v

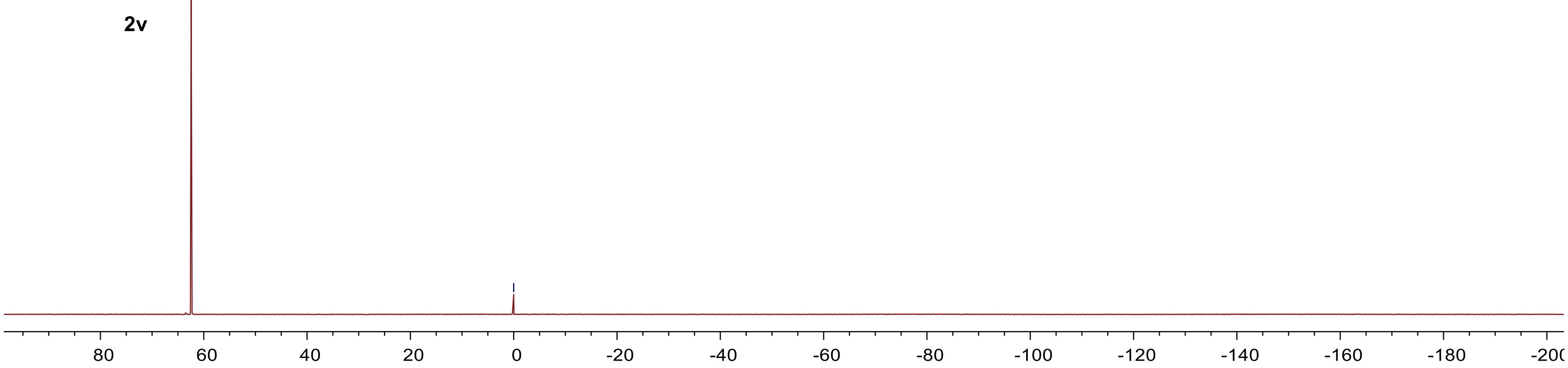

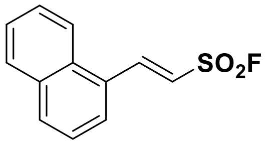

2w

^{1}H NMR (400 MHz, $CDCl_3$):

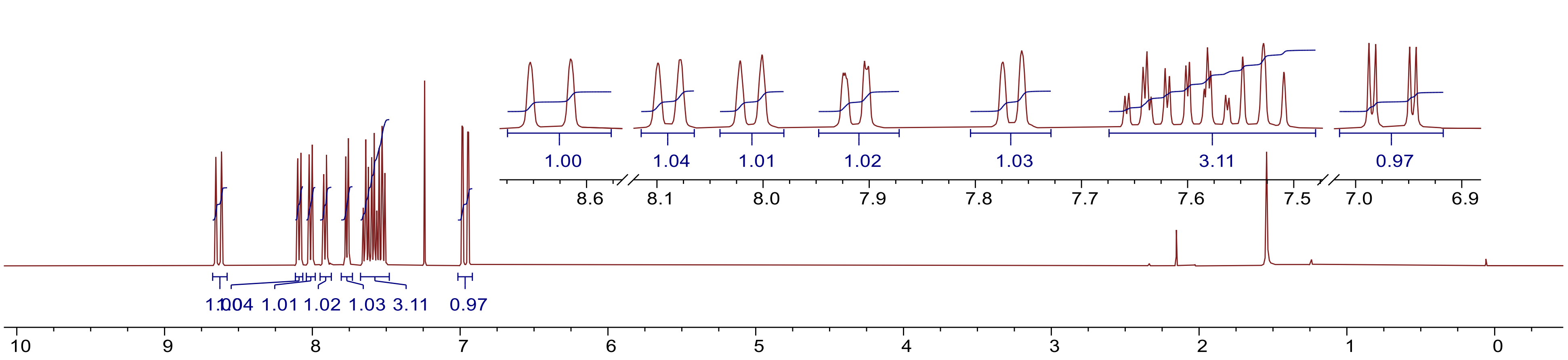

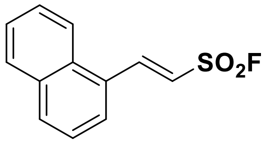

2w

^{13}C NMR (100 MHz, $CDCl_3$):

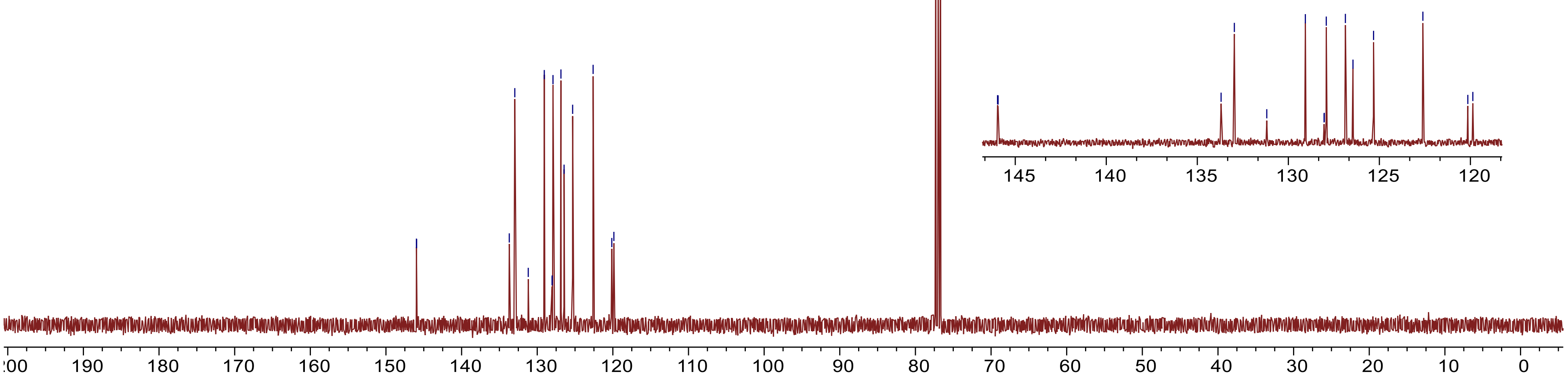

^{19}F NMR (376 MHz, CDCl$_3$):

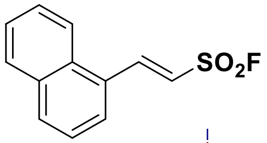

2w

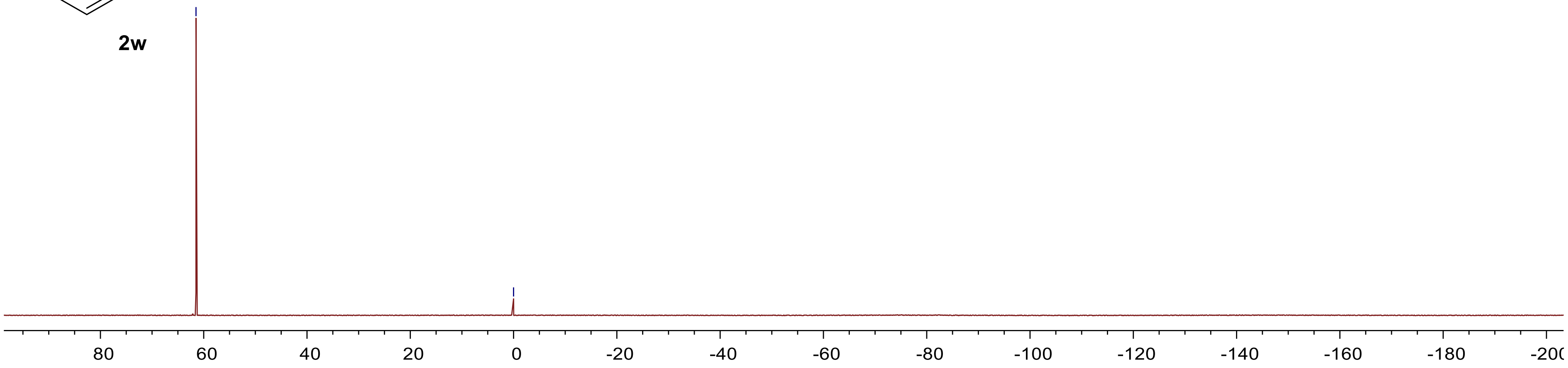

^{1}H NMR (400 MHz, $CDCl_3$):

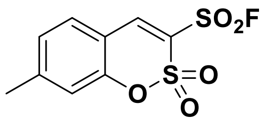

3a

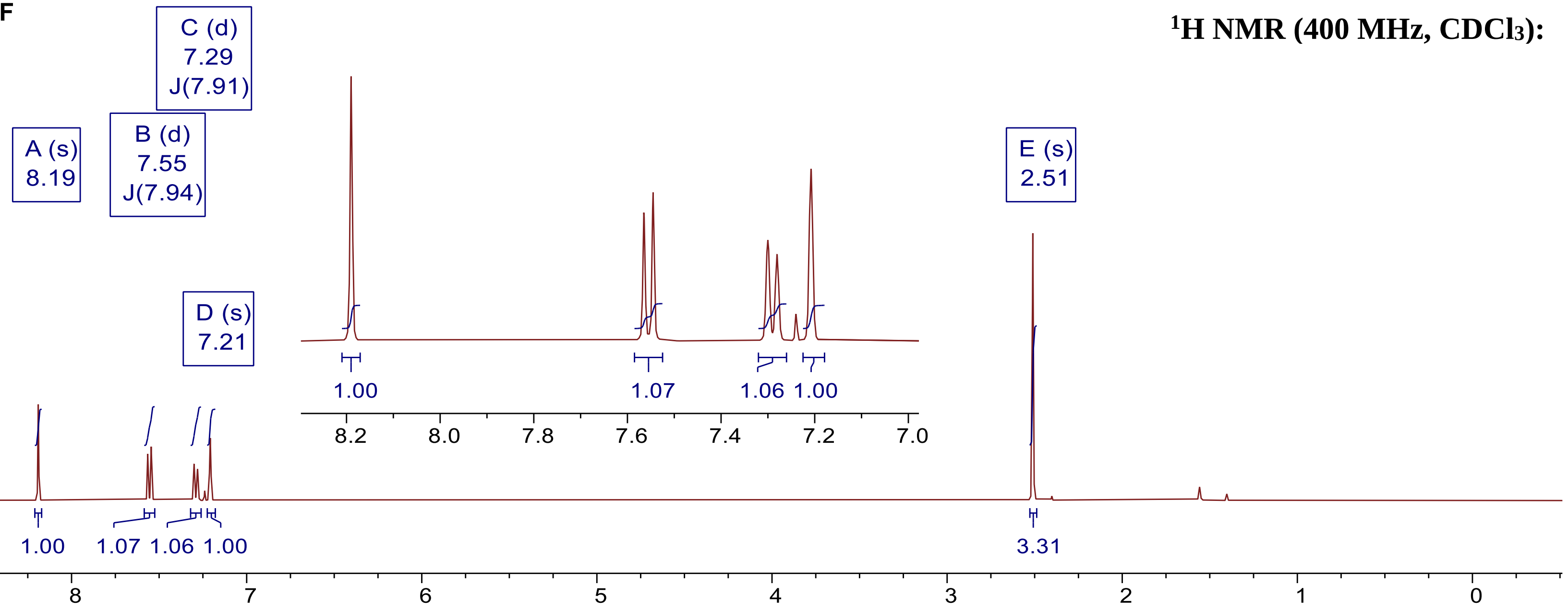

^{13}C NMR (100 MHz, $CDCl_3$):

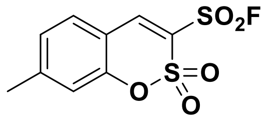

3a

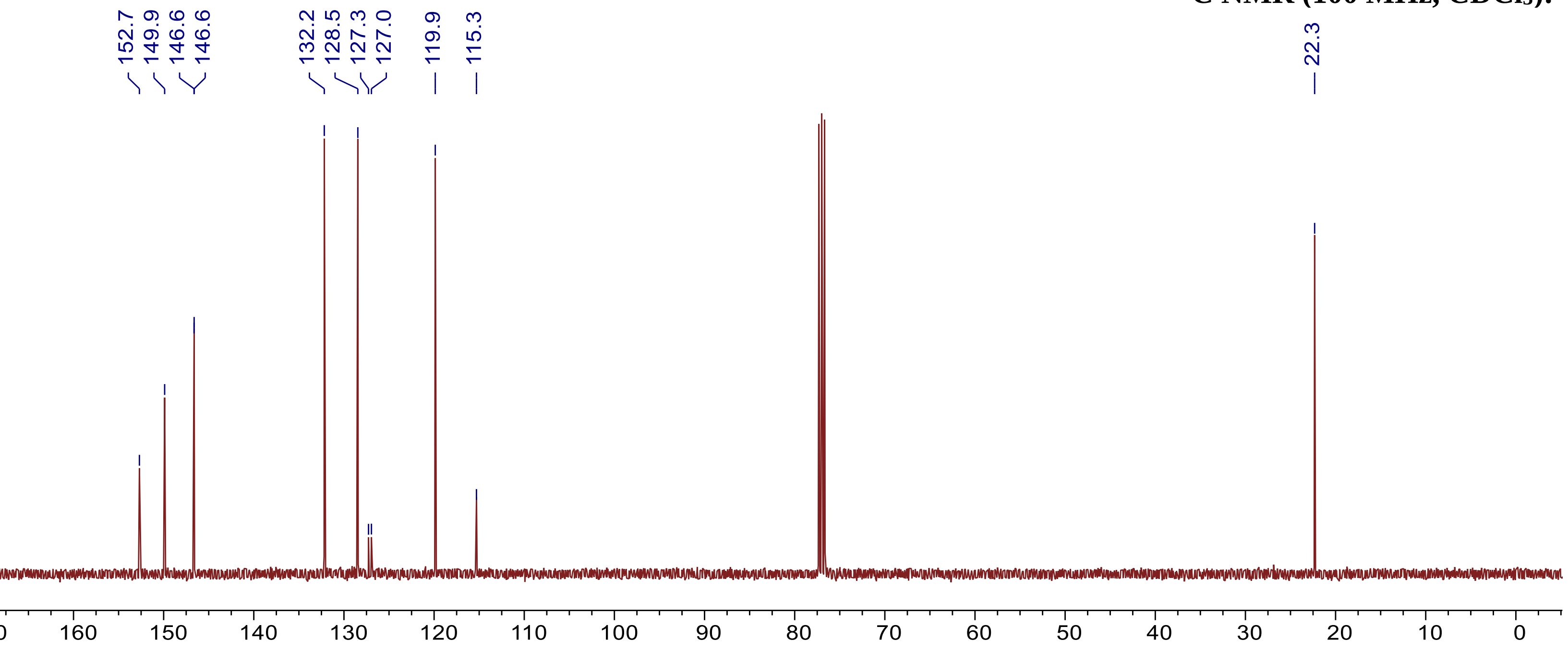

^{19}F NMR (376 MHz, CDCl3):

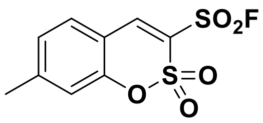

3a

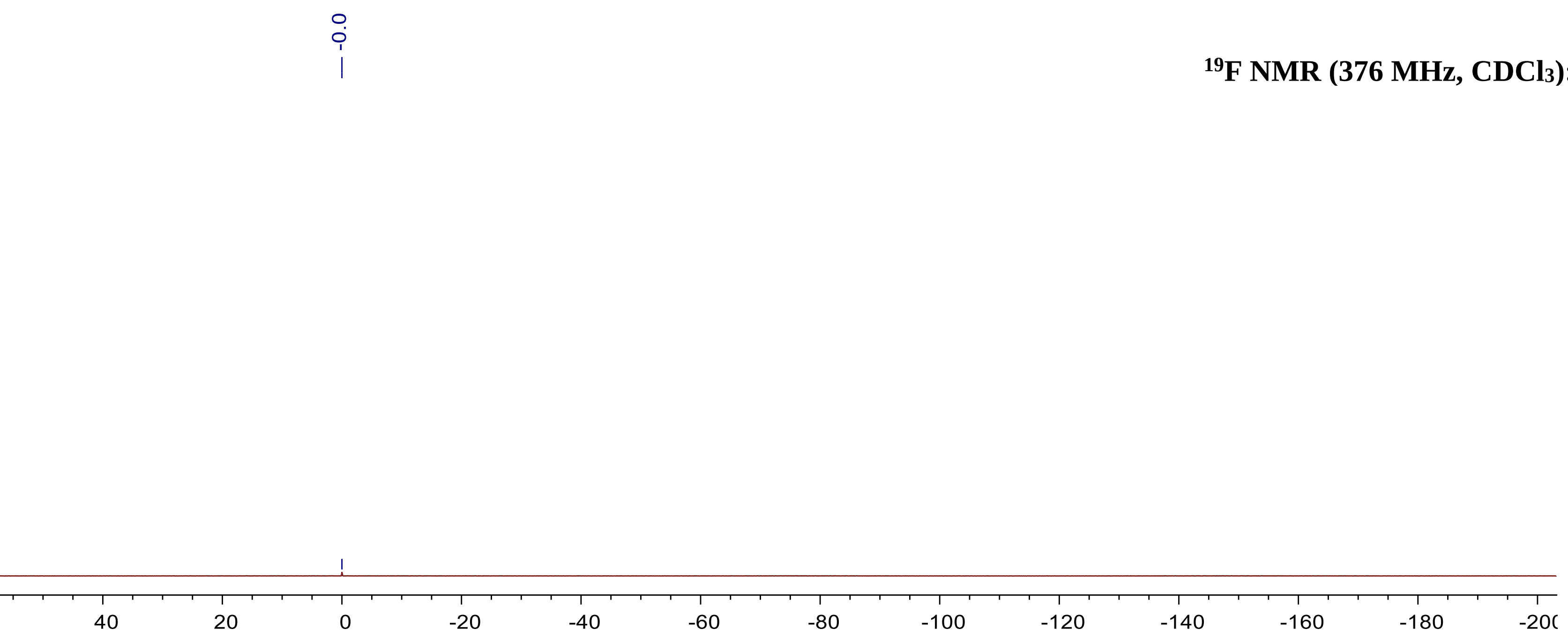

^{1}H NMR (400 MHz, CDCl3):

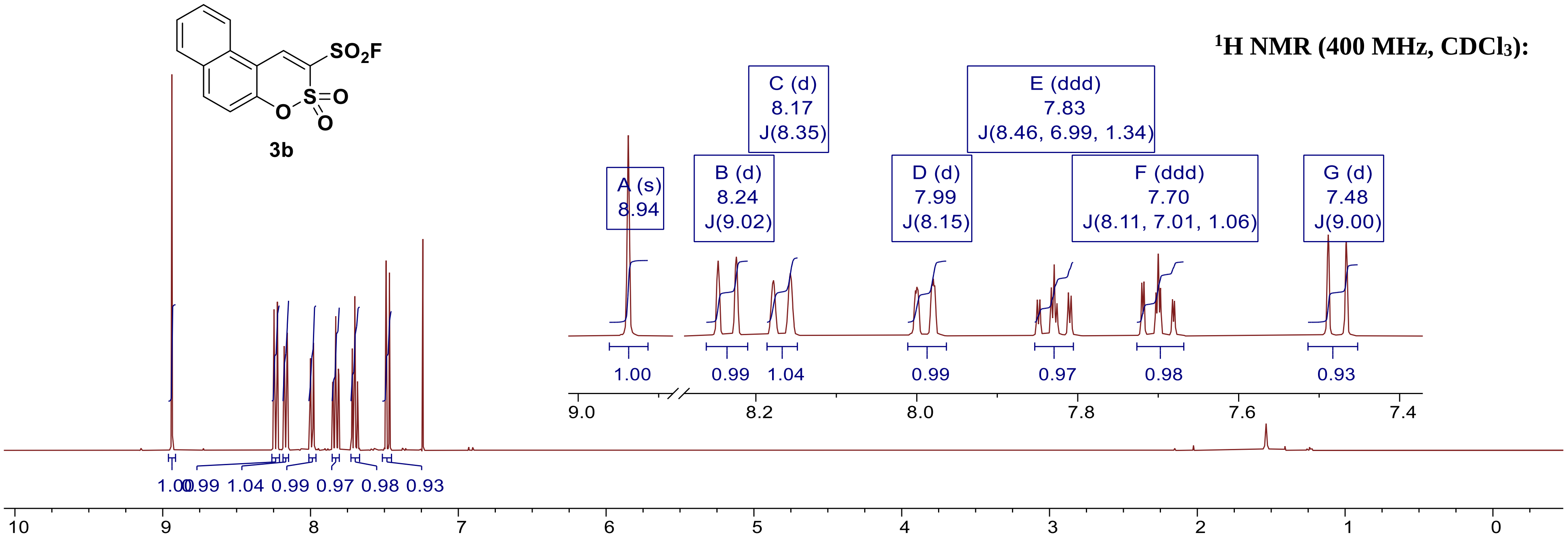

^{13}C NMR (100 MHz, CDCl3):

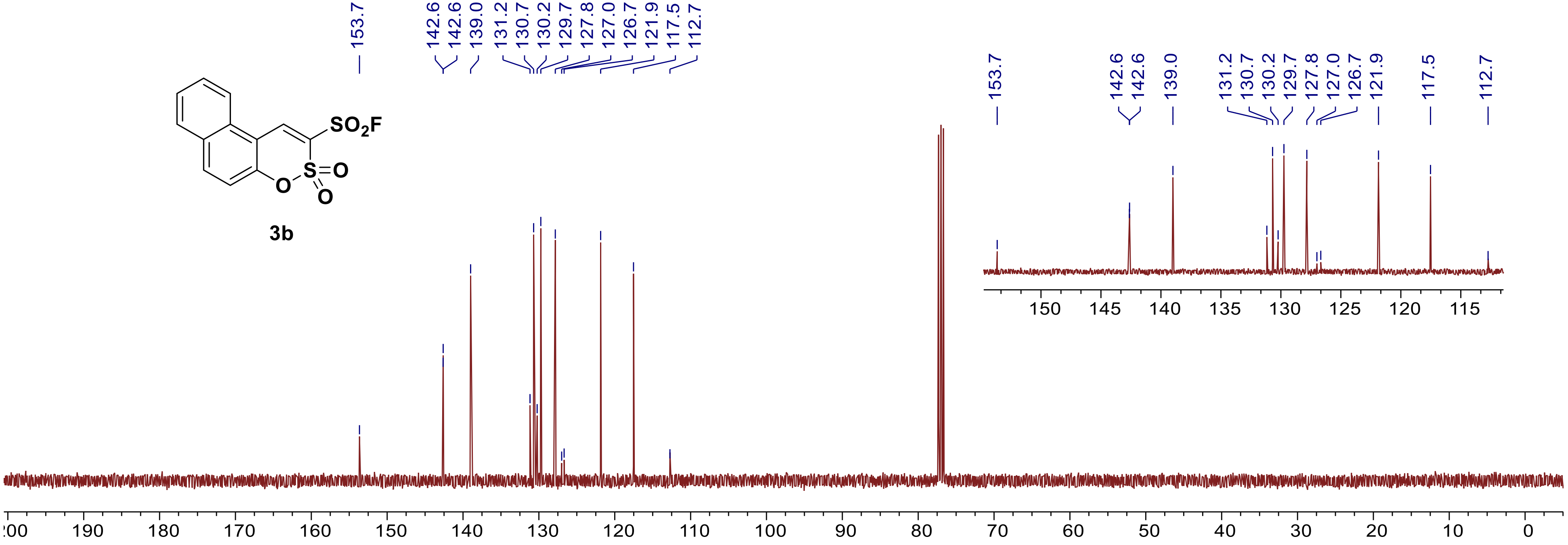

^{19}F NMR (376 MHz, $CDCl_3$):

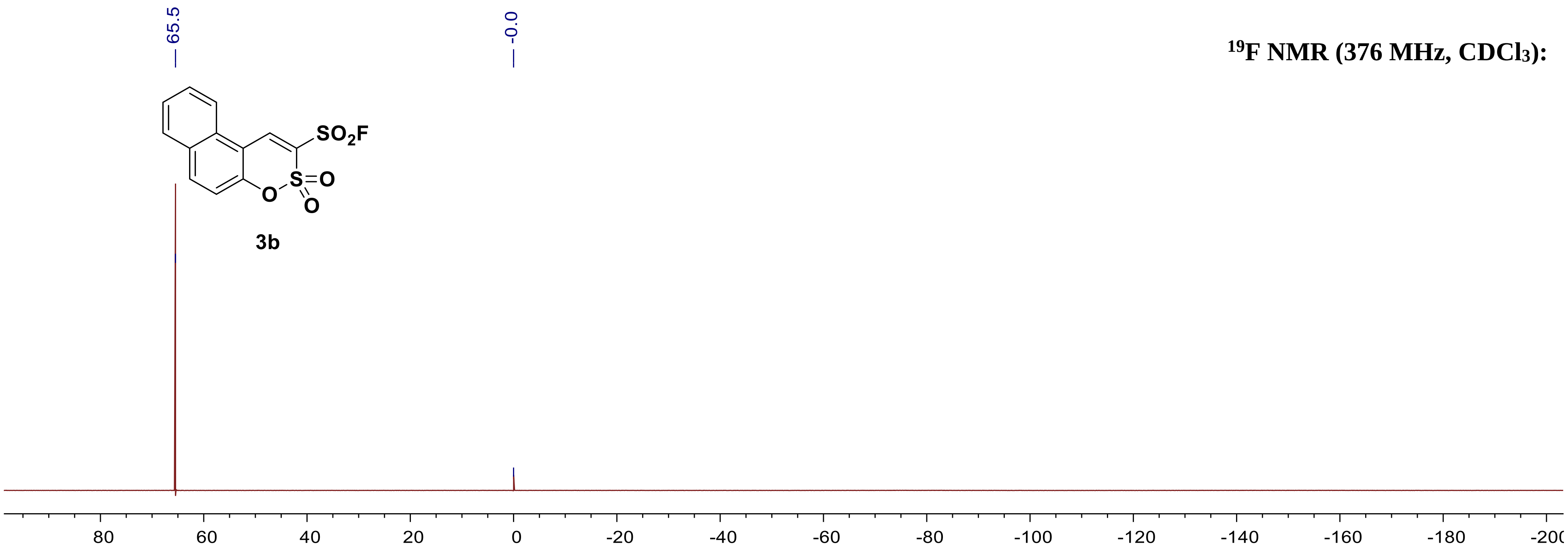

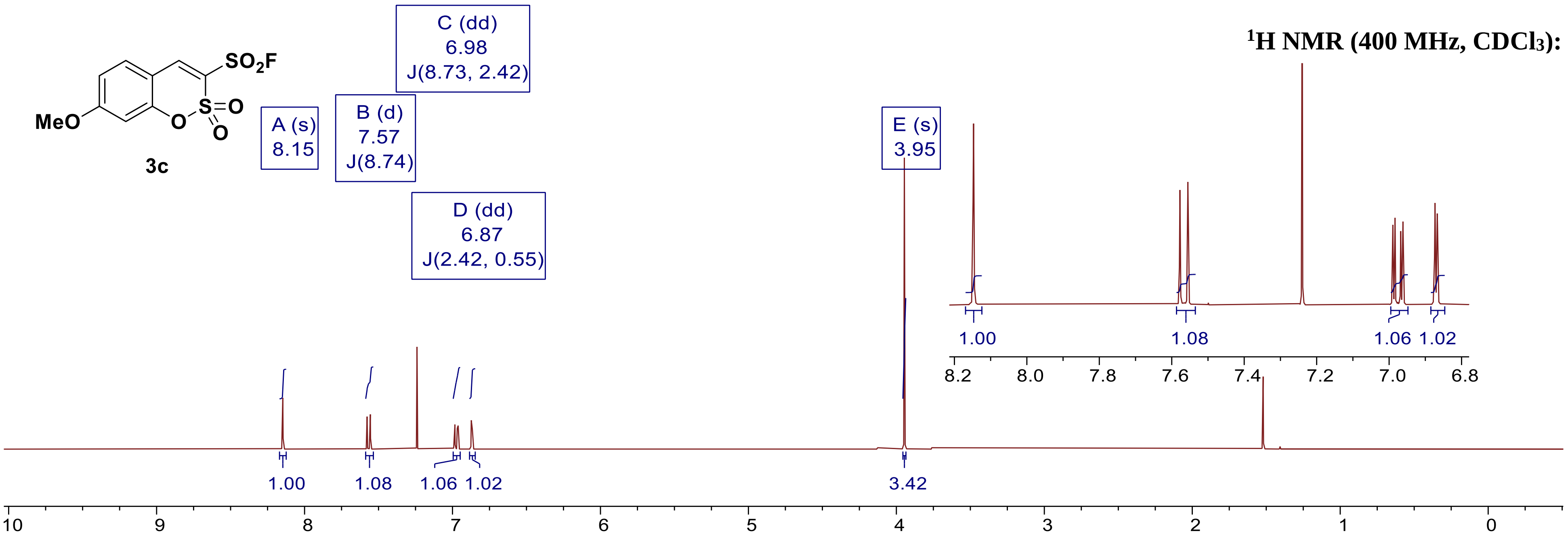

MeO
SO2F
S=O
O
O
3c
A (s)
8.15
B (d)
7.57
J(8.74)
C (dd)
6.98
J(8.73, 2.42)
D (dd)
6.87
J(2.42, 0.55)
E (s)
3.95
1H NMR (400 MHz, CDCl3):
1.00
1.08
1.06 1.02
3.42

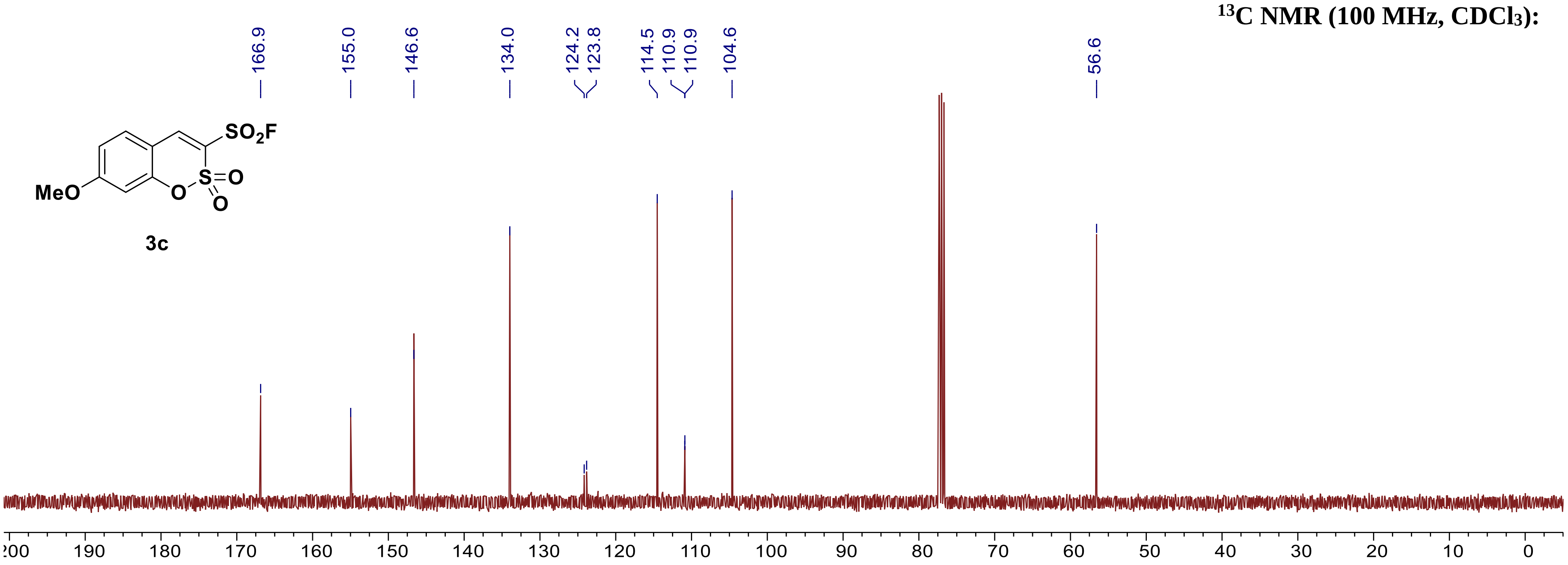

13C NMR (100 MHz, CDCl3):
166.9
155.0
146.6
134.0
124.2
123.8
114.5
110.9
110.9
104.6
56.6
MeO
SO2F
S=O
O
O
3c

^{19}F NMR (376 MHz, $CDCl_3$):

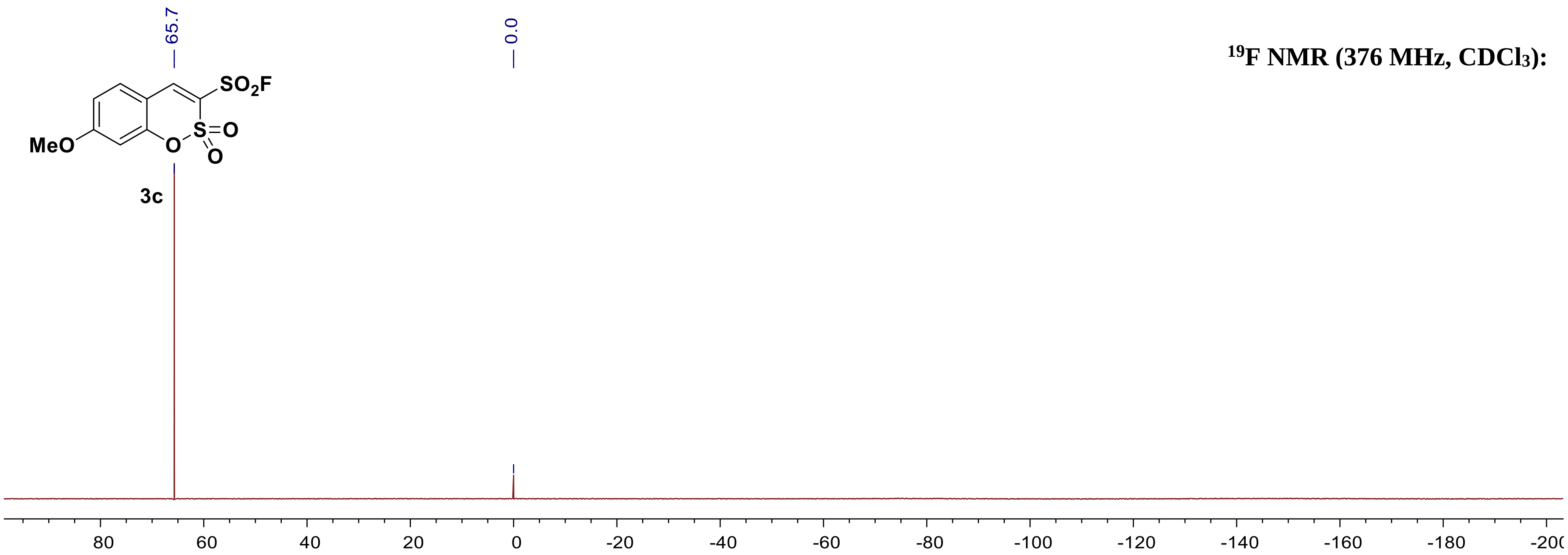

^{1}H NMR (400 MHz, $CDCl_3$):

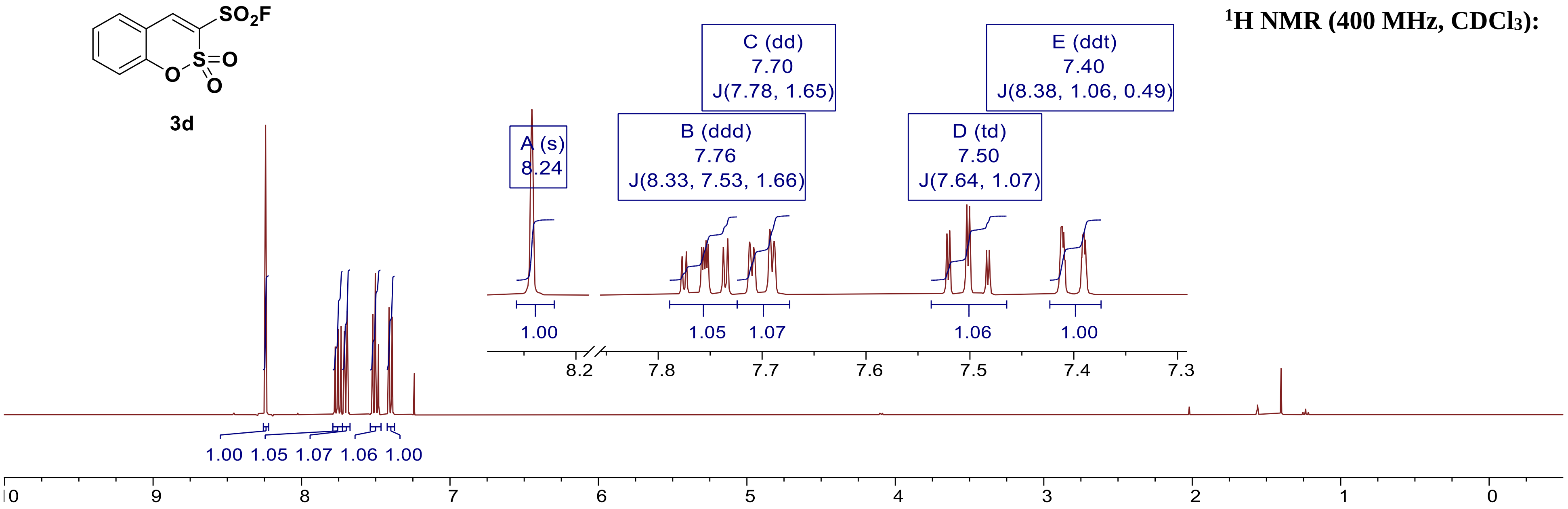

^{13}C NMR (100 MHz, $CDCl_3$):

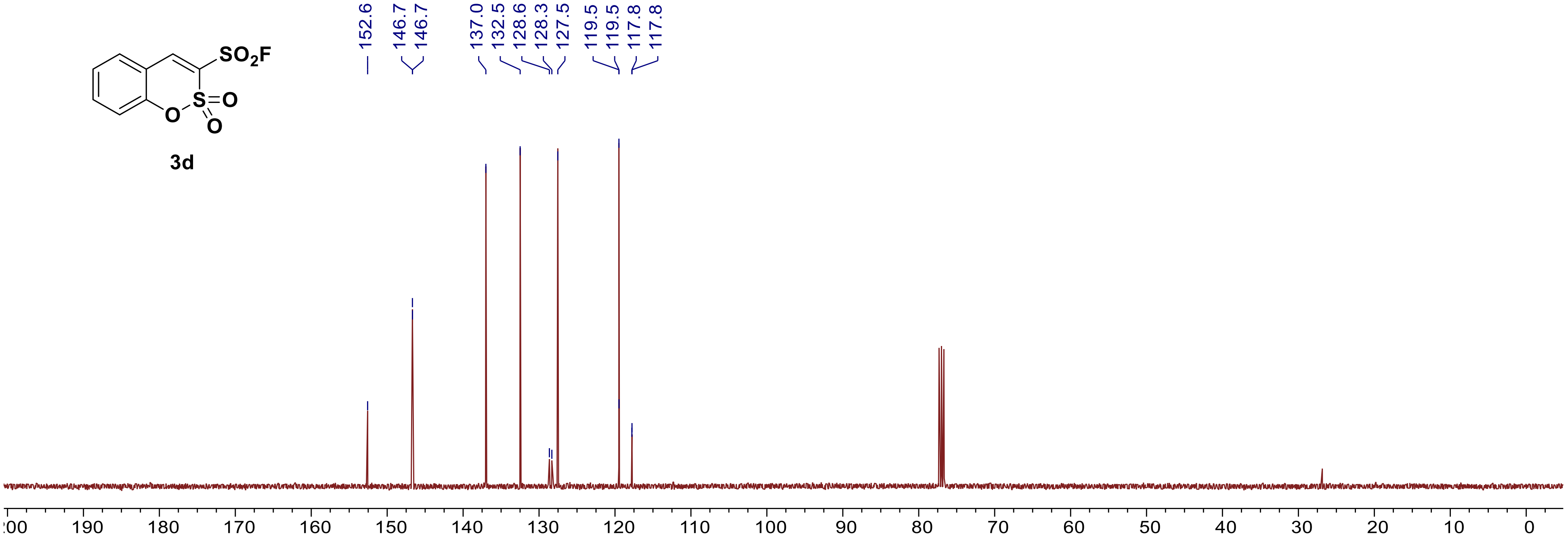

^{19}F NMR (376 MHz, $CDCl_3$):

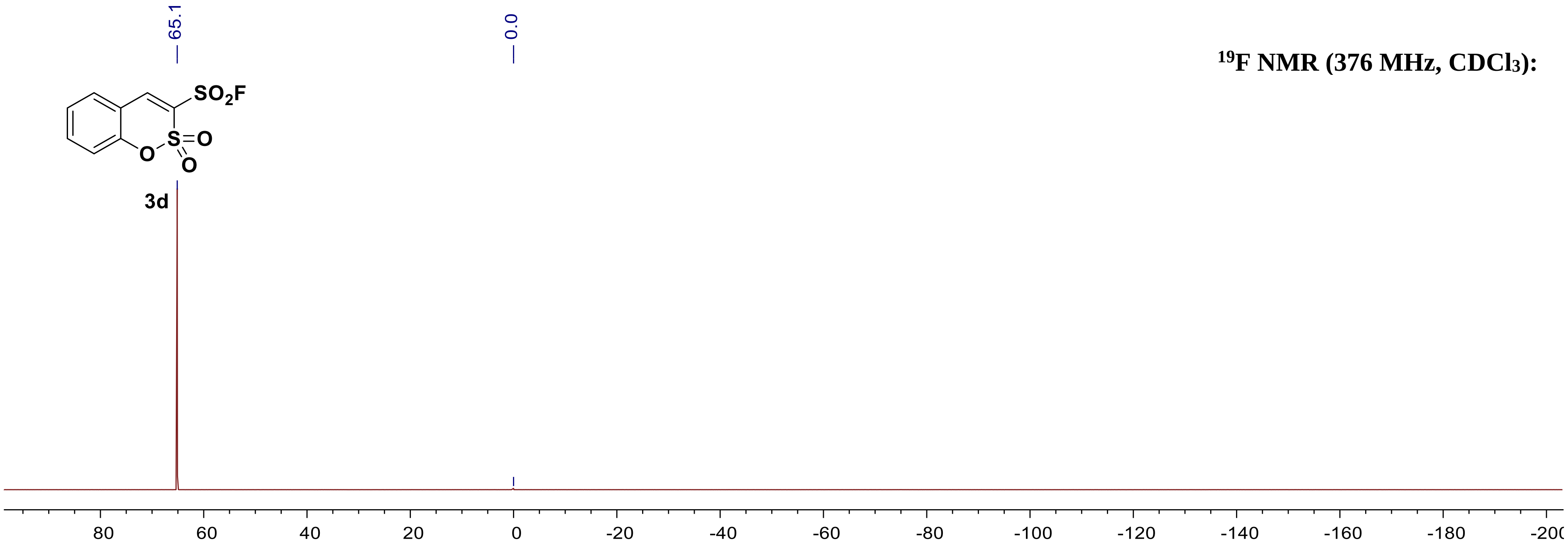

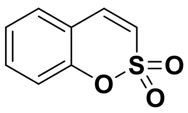

3d'

^{1}H NMR (400 MHz, CDCl$_3$):

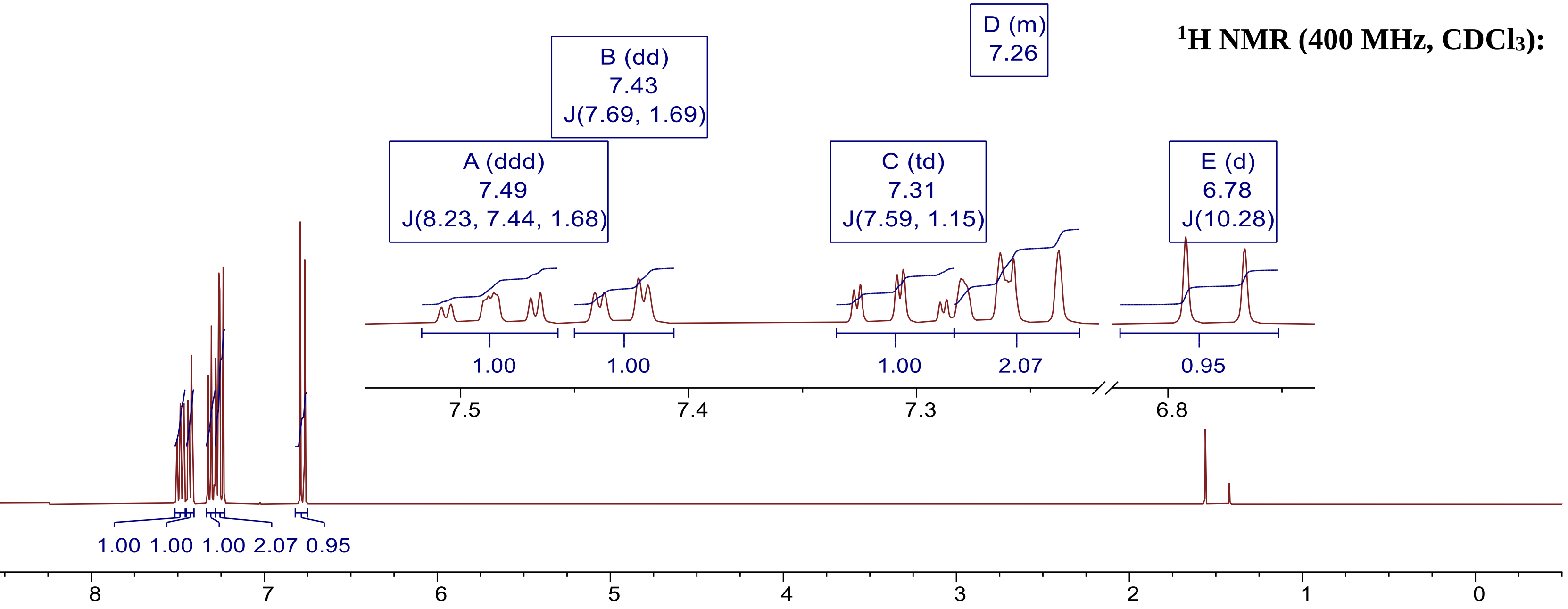

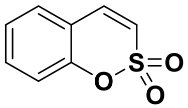

3d'

^{13}C NMR (100 MHz, CDCl$_3$):

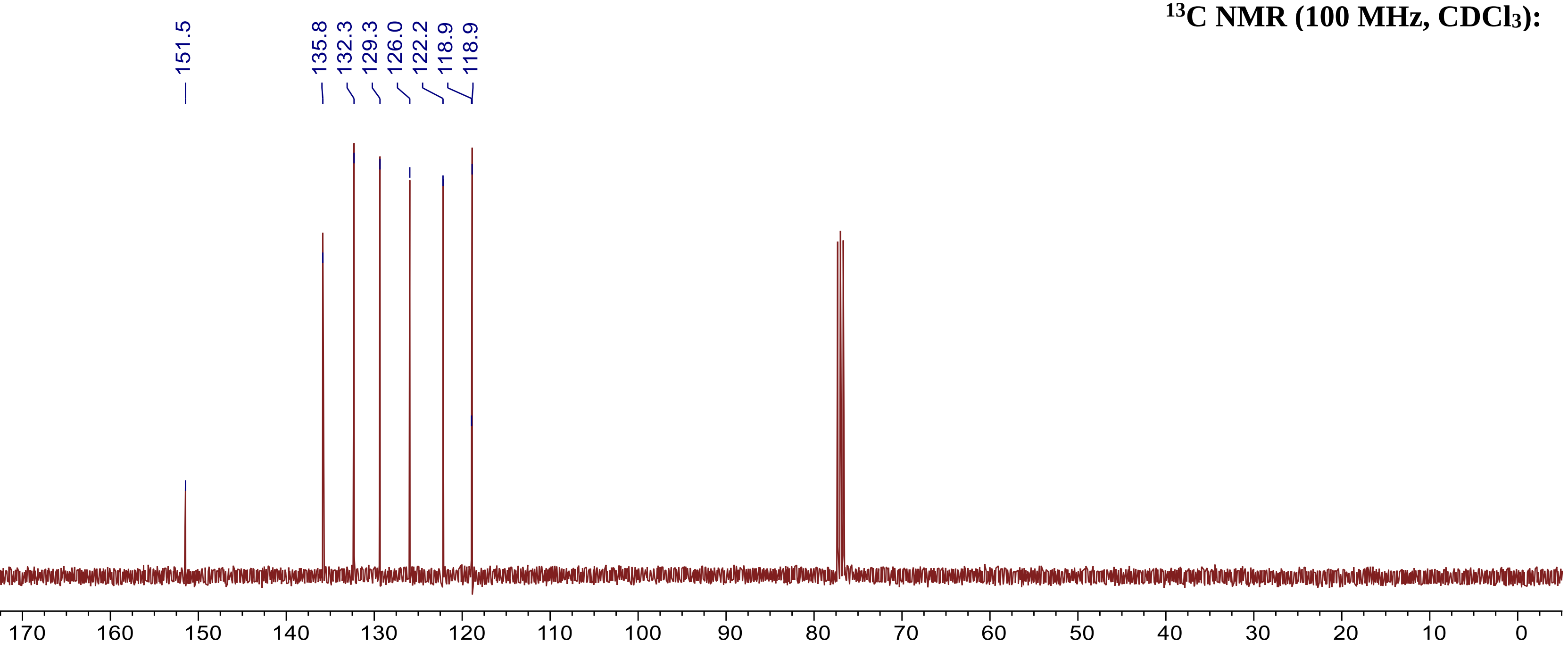

^{1}H NMR (400 MHz, $CDCl_3$):

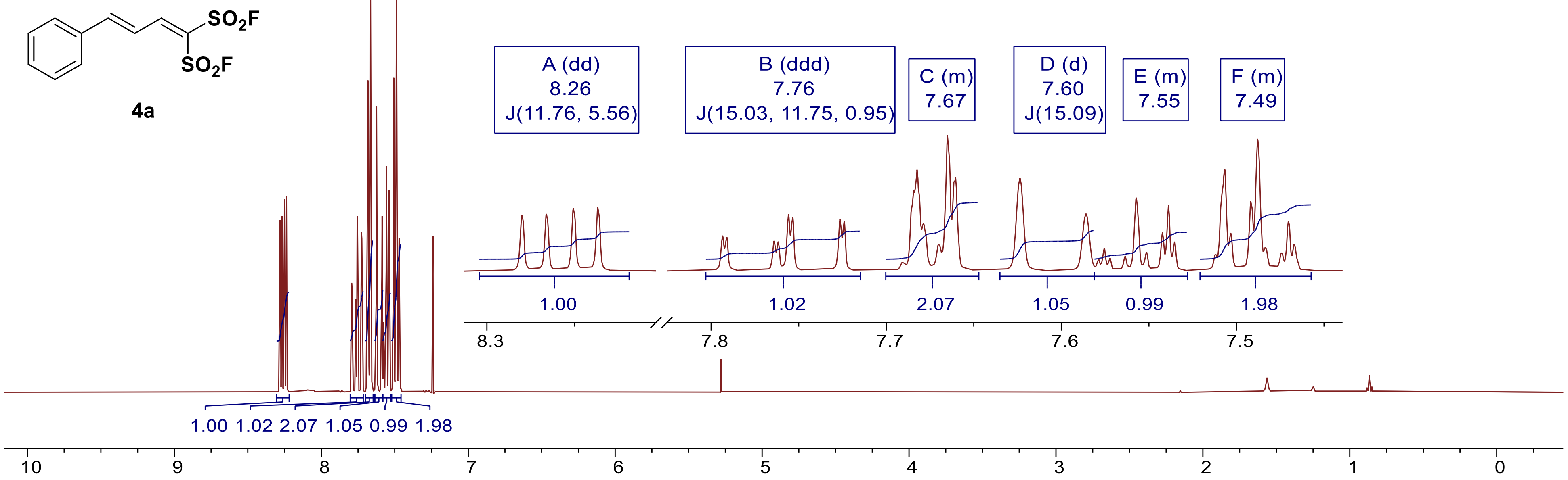

^{13}C NMR (100 MHz, $CDCl_3$):

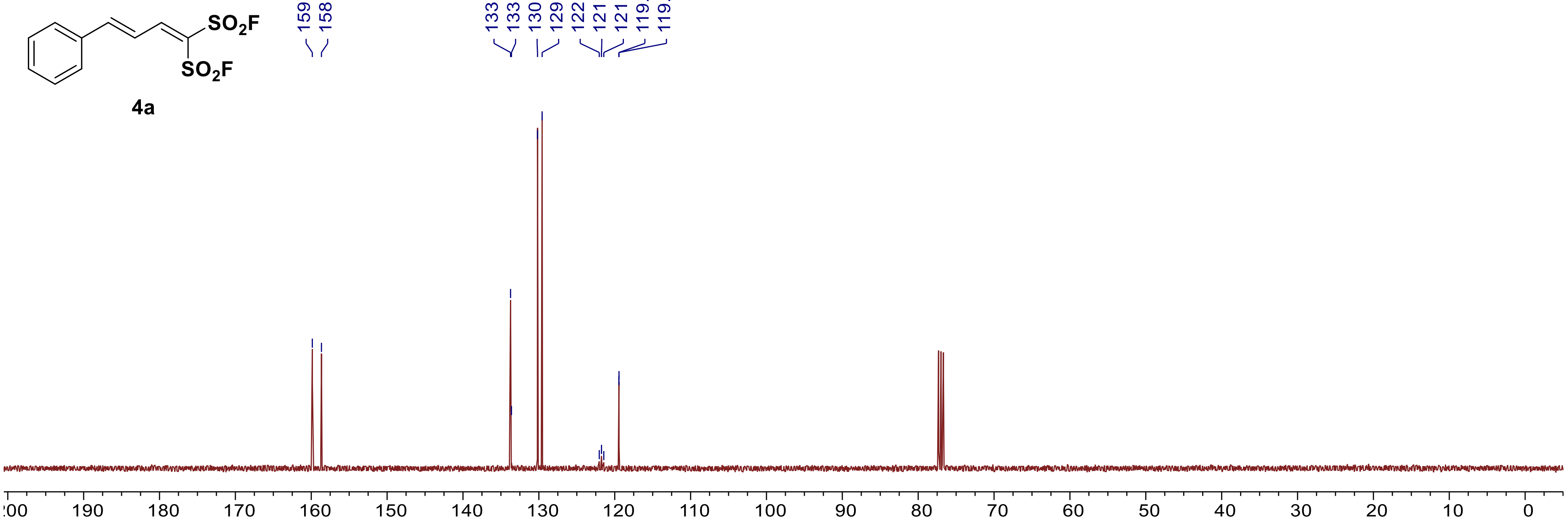

^{19}F NMR (376 MHz, $CDCl_3$):

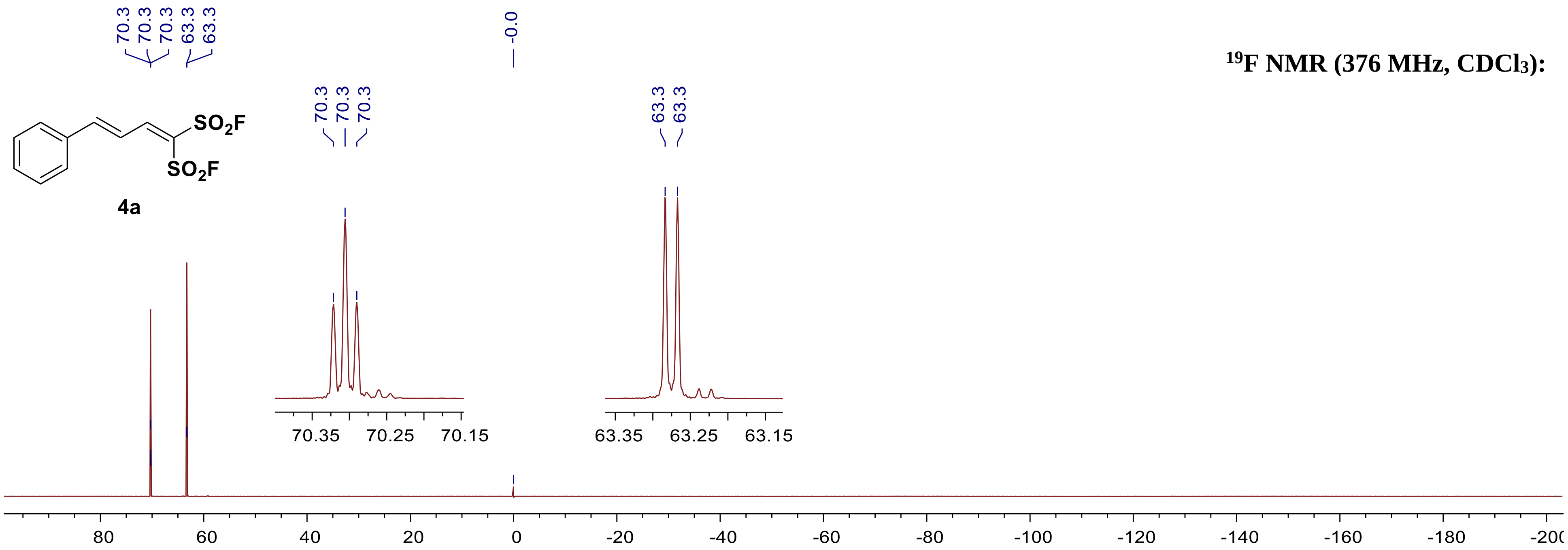

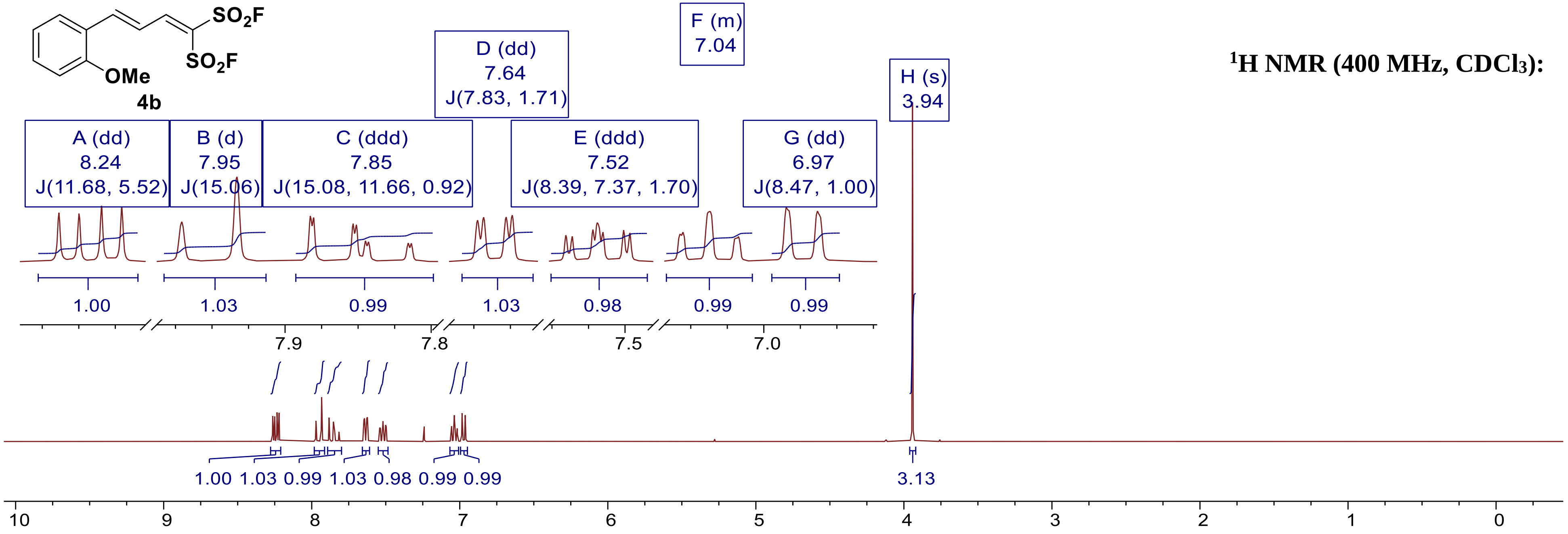
SO2F
SO2F
OMe
4b
1H NMR (400 MHz, CDCl3):
A (dd) 8.24 J(11.68, 5.52)
B (d) 7.95 J(15.06)
C (ddd) 7.85 J(15.08, 11.66, 0.92)
D (dd) 7.64 J(7.83, 1.71)
E (ddd) 7.52 J(8.39, 7.37, 1.70)
F (m) 7.04
G (dd) 6.97 J(8.47, 1.00)
H (s) 3.94
1.00 1.03 0.99 1.03 0.98 0.99 0.99
3.13

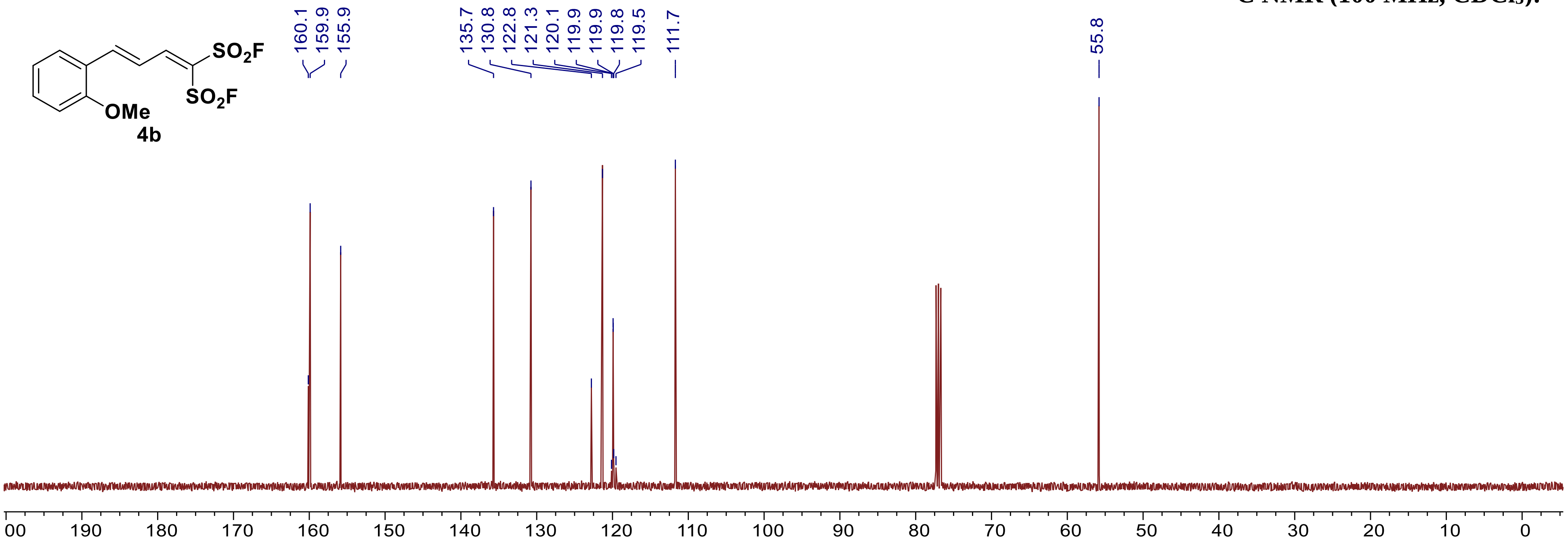
13C NMR (100 MHz, CDCl3):
160.1
159.9
155.9
135.7
130.8
122.8
121.3
120.1
119.9
119.9
119.8
119.5
111.7
55.8
SO2F
SO2F
OMe
4b

^{19}F NMR (376 MHz, $CDCl_3$):

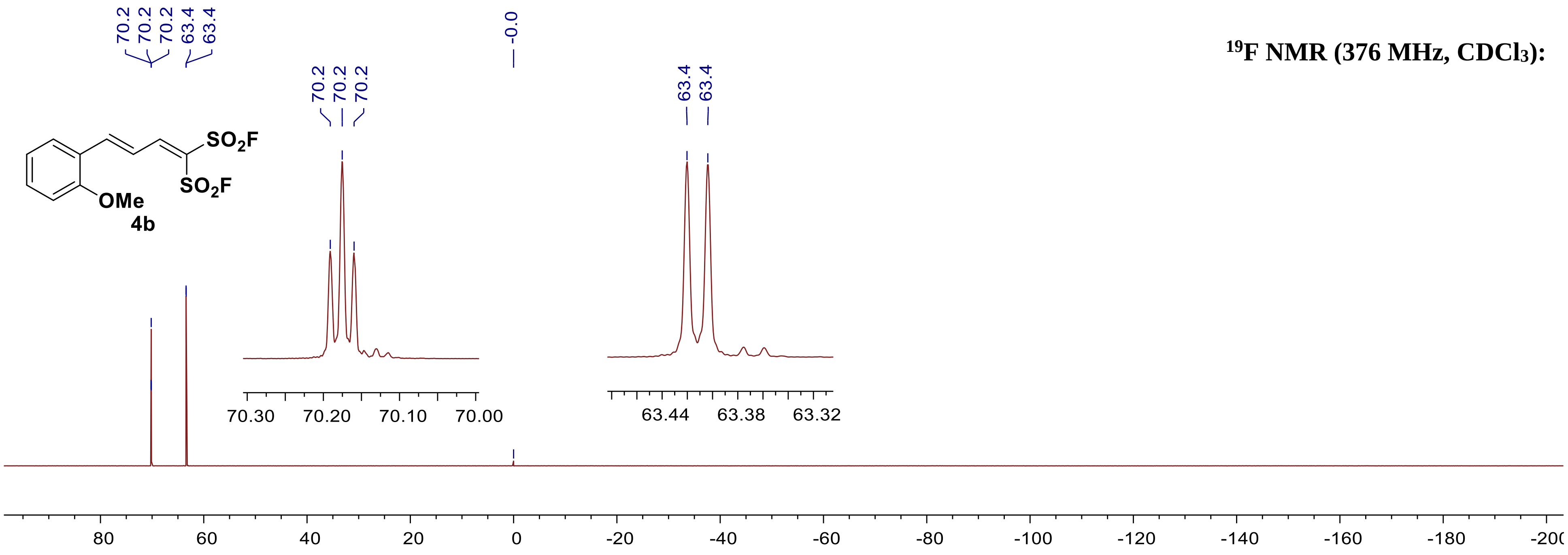

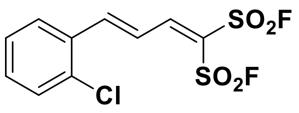

4c

^{1}H NMR (400 MHz, CDCl3):

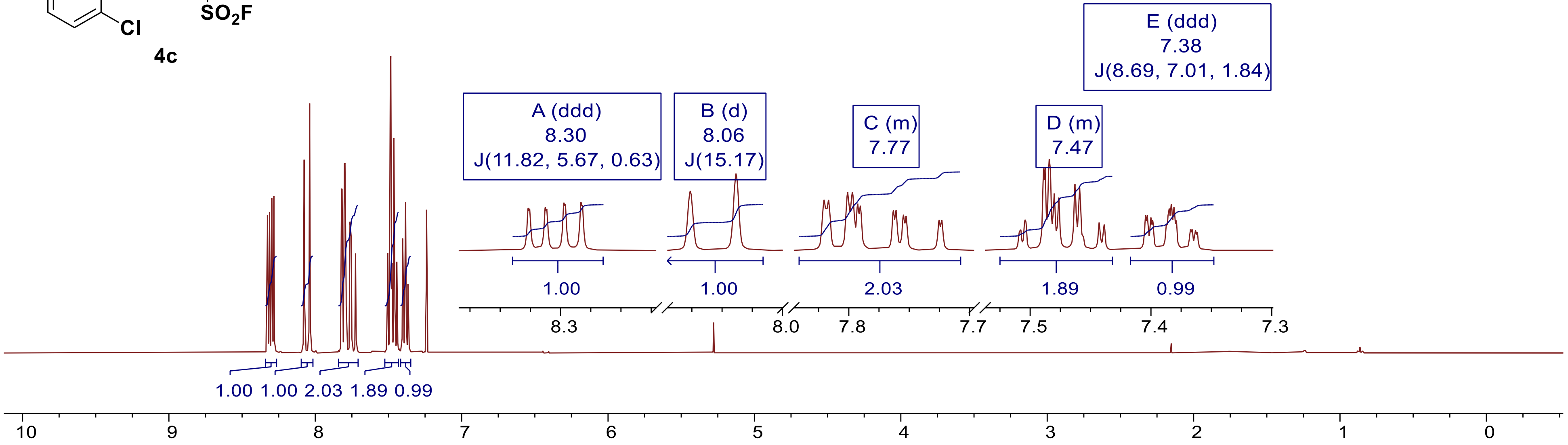

^{13}C NMR (100 MHz, CDCl3):

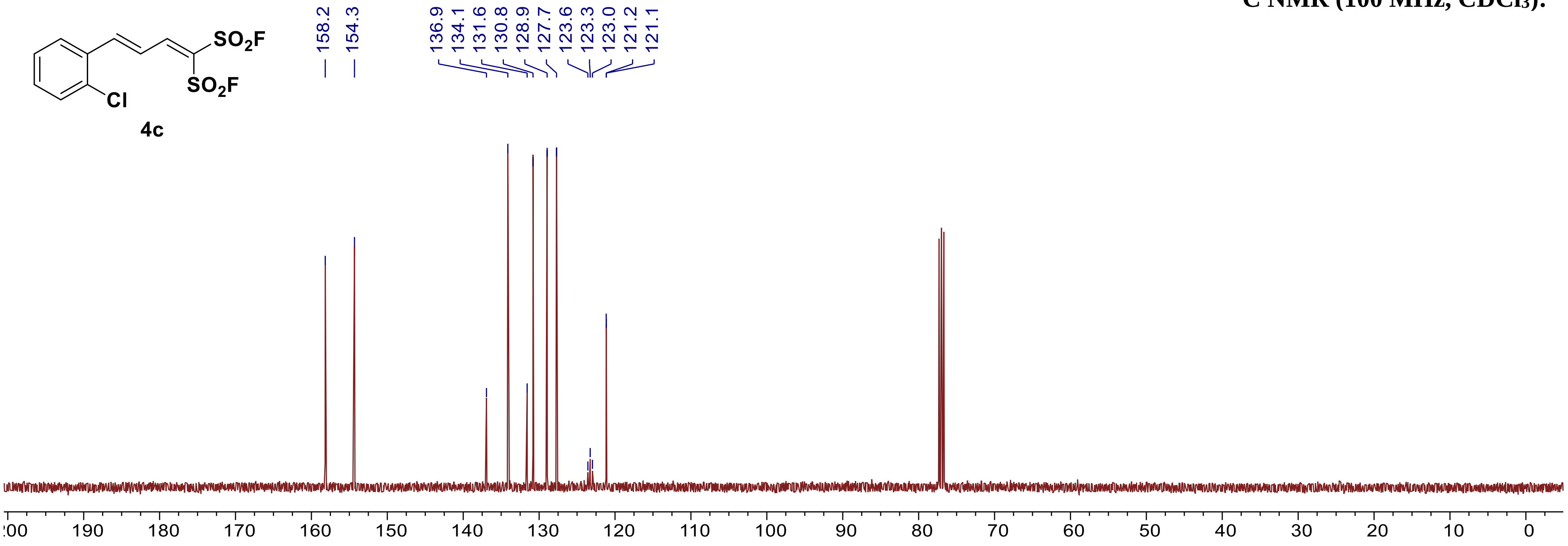

4c

^{19}F NMR (376 MHz, CDCl₃):

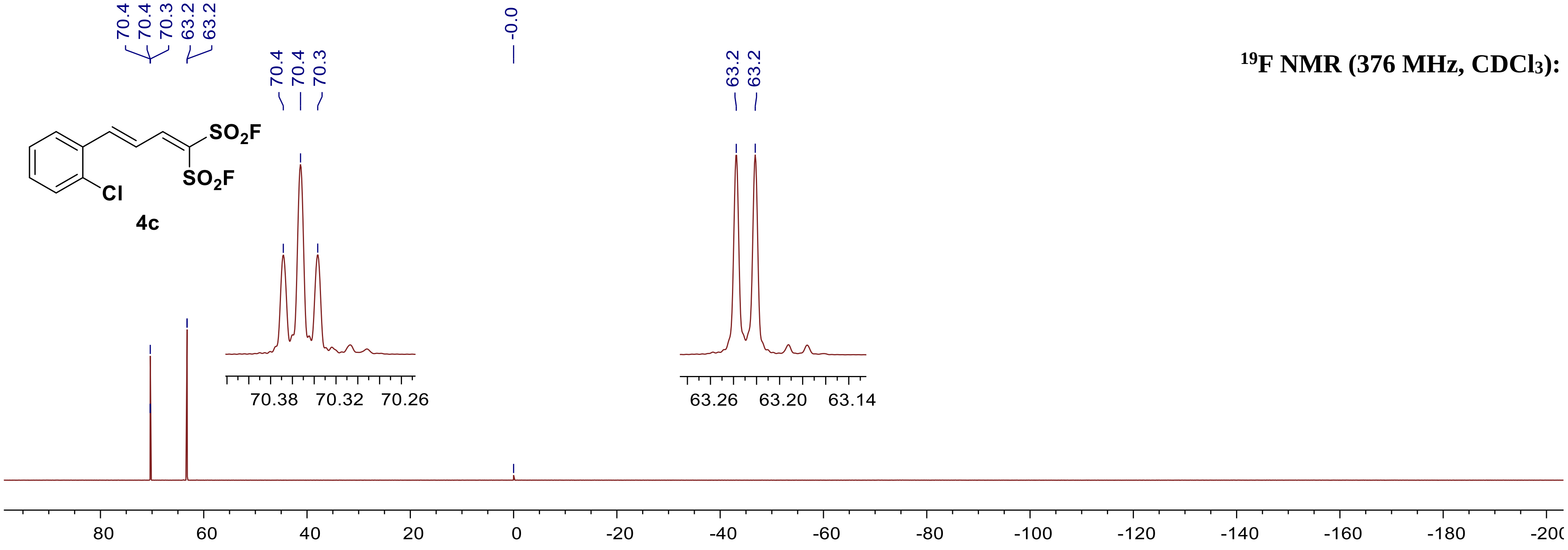

^{1}H NMR (400 MHz, $CDCl_3$):

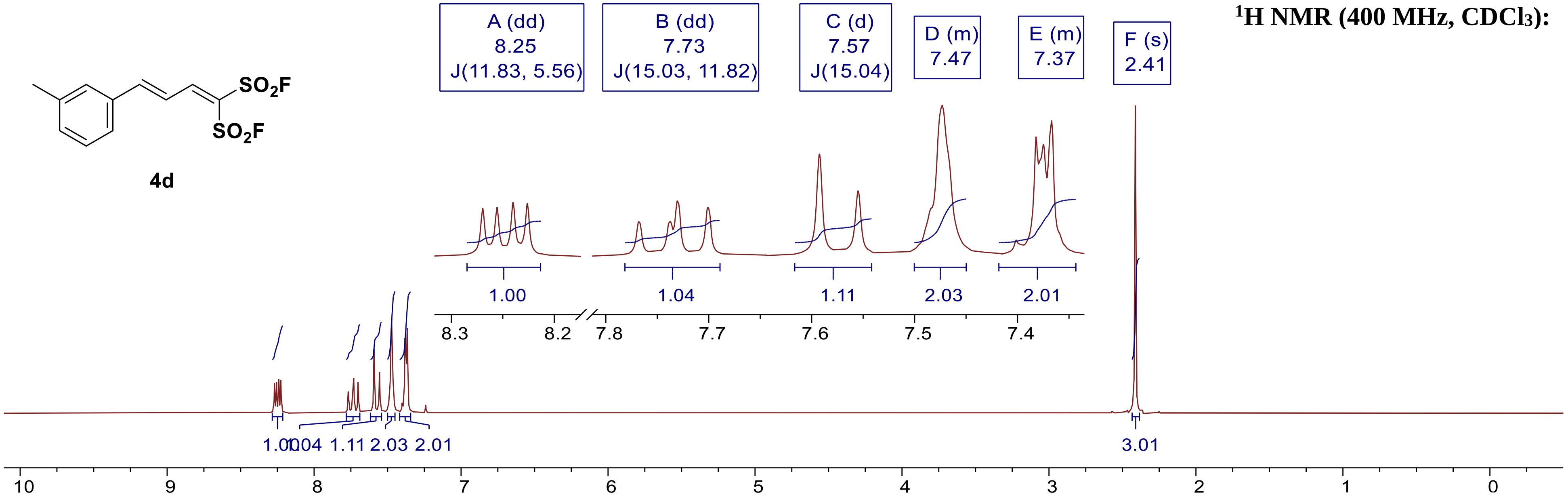

^{13}C NMR (100 MHz, $CDCl_3$):

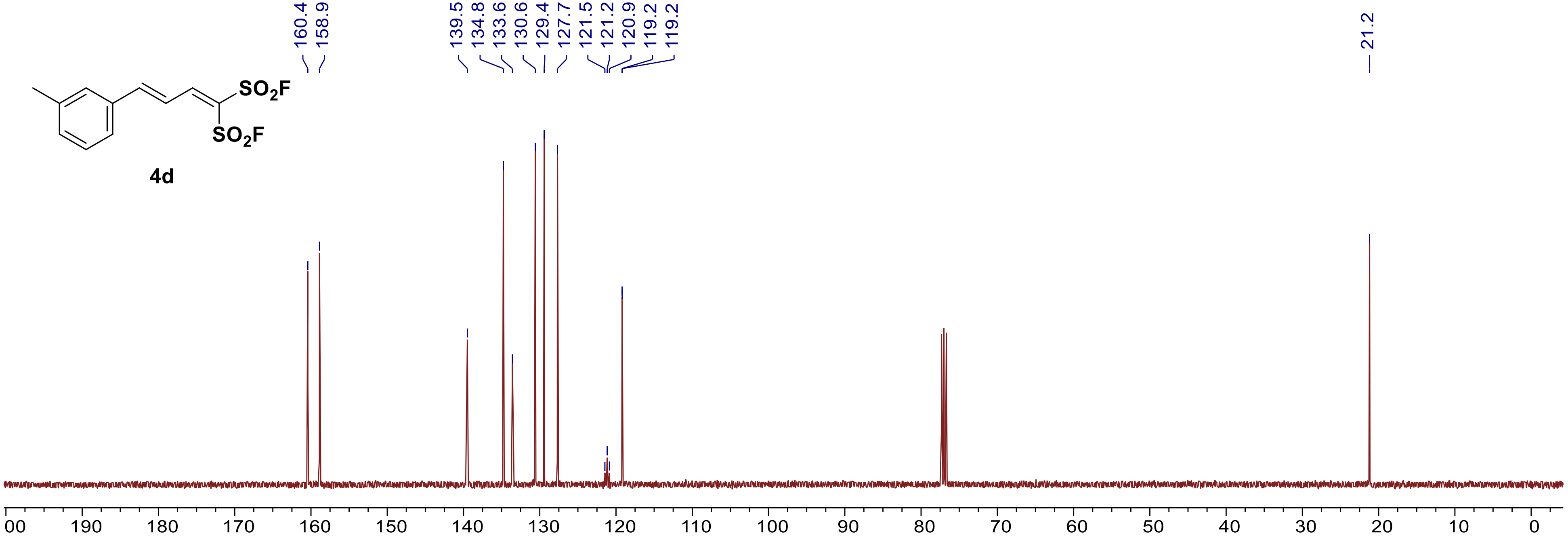

^{19}F NMR (376 MHz, CDCl$_3$):

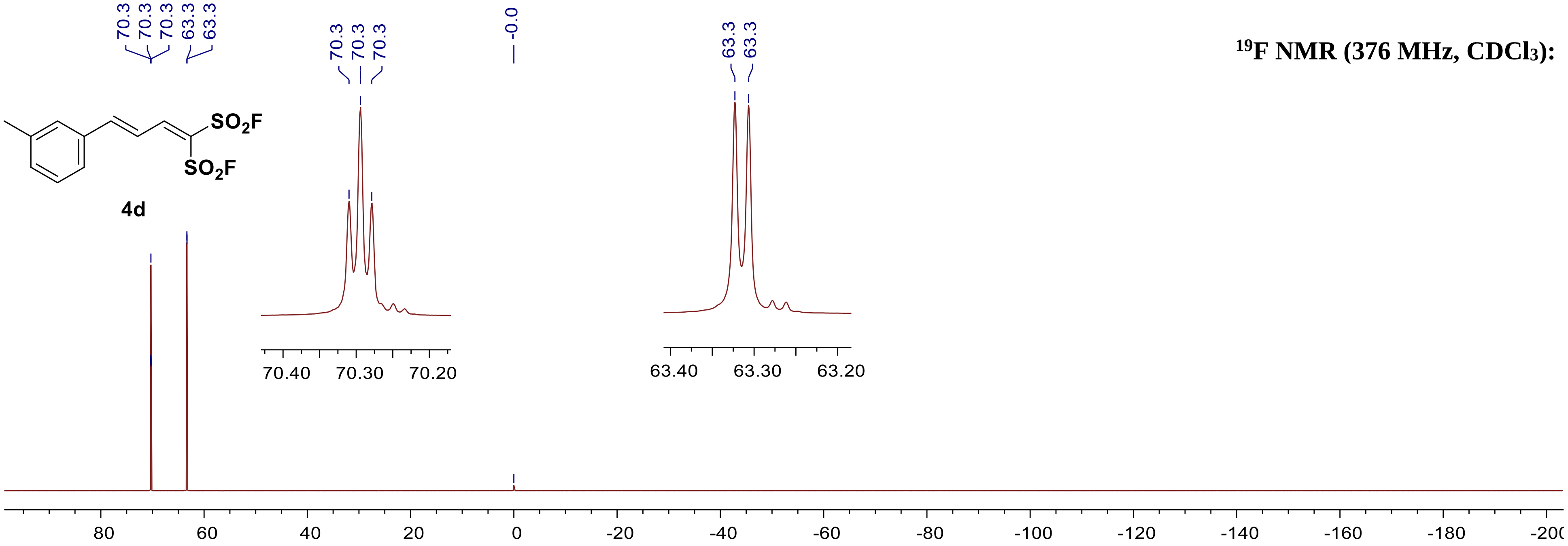

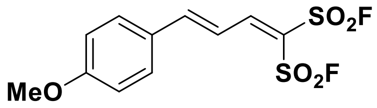

4e

^{1}H NMR (400 MHz, CDCl3):

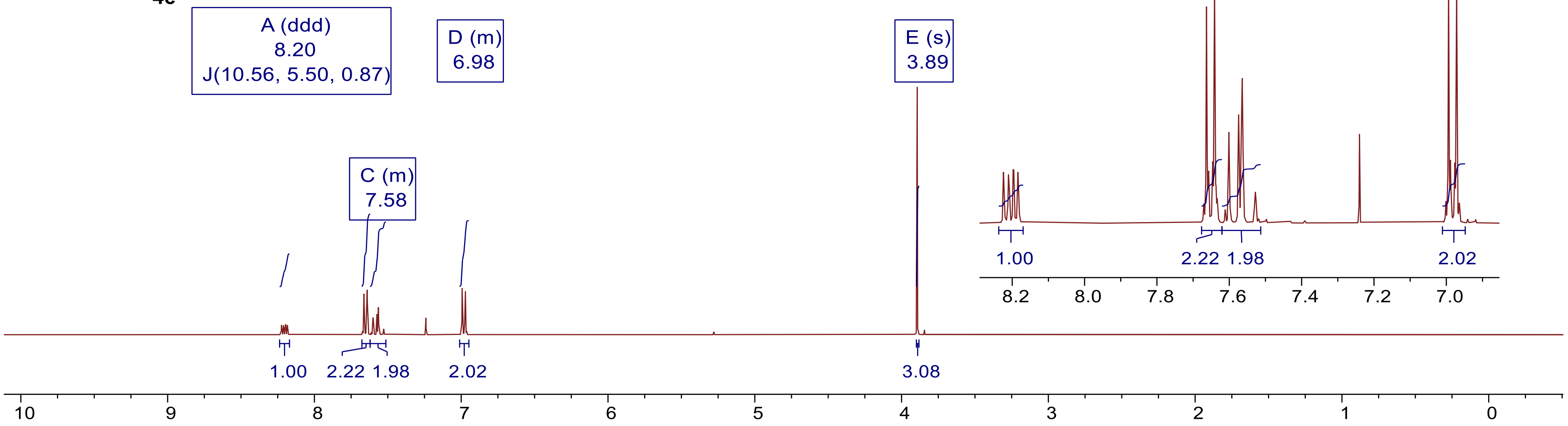

4e

^{13}C NMR (100 MHz, CDCl3):

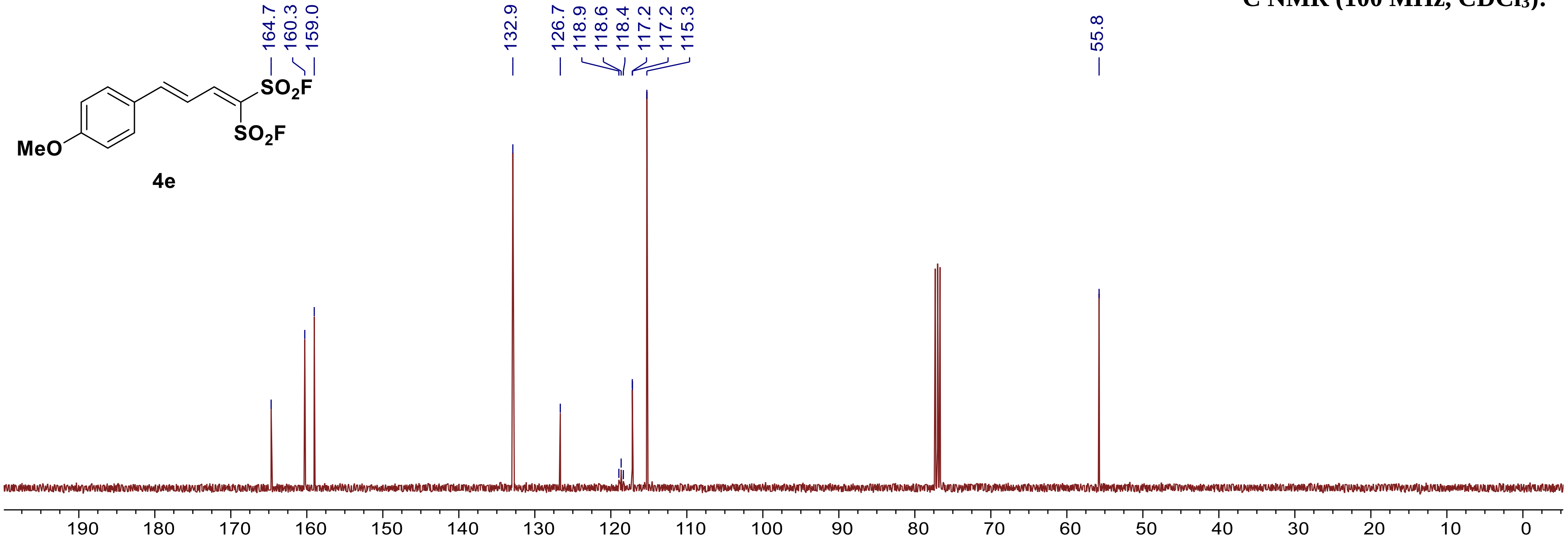

^{19}F NMR (376 MHz, CDCl$_3$):

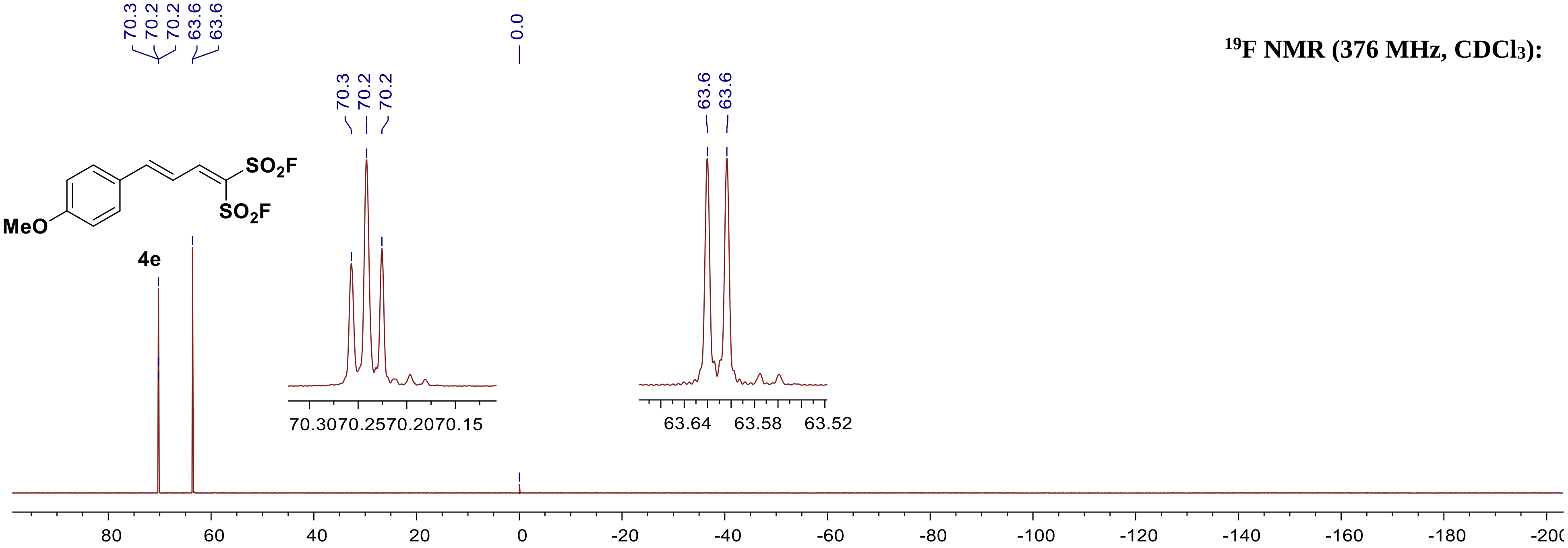

^{1}H NMR (400 MHz, acetone-d_3):

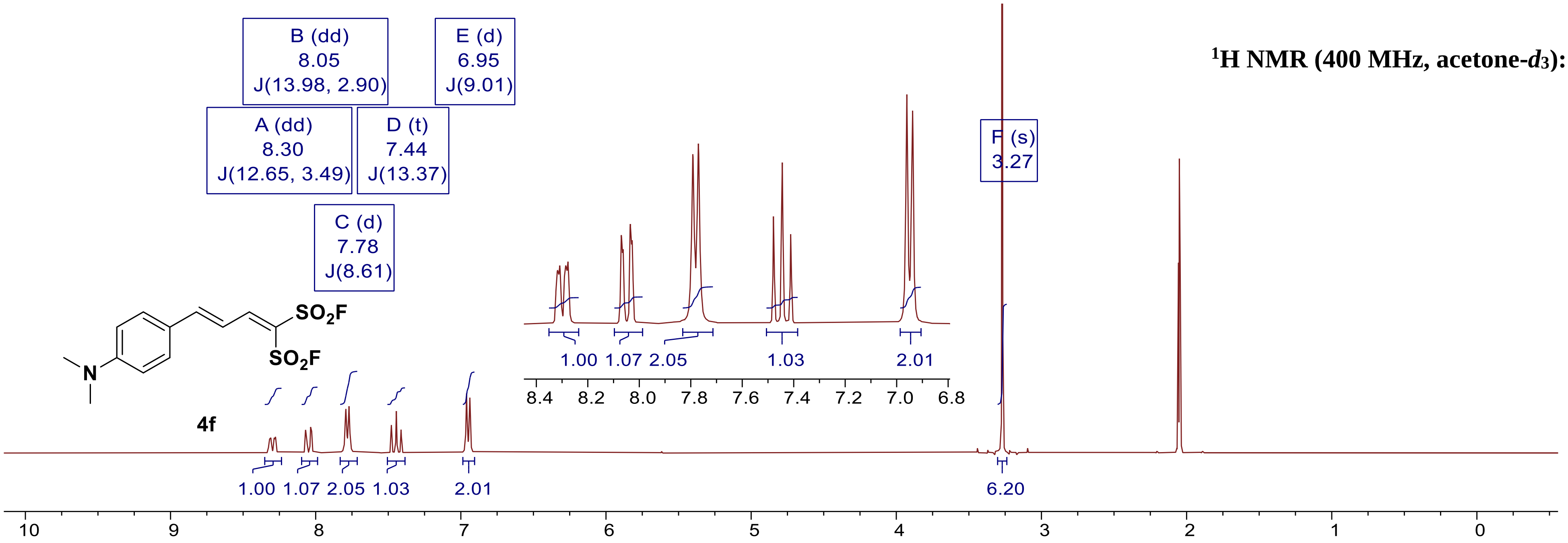

^{13}C NMR (100 MHz, acetone-d_3):

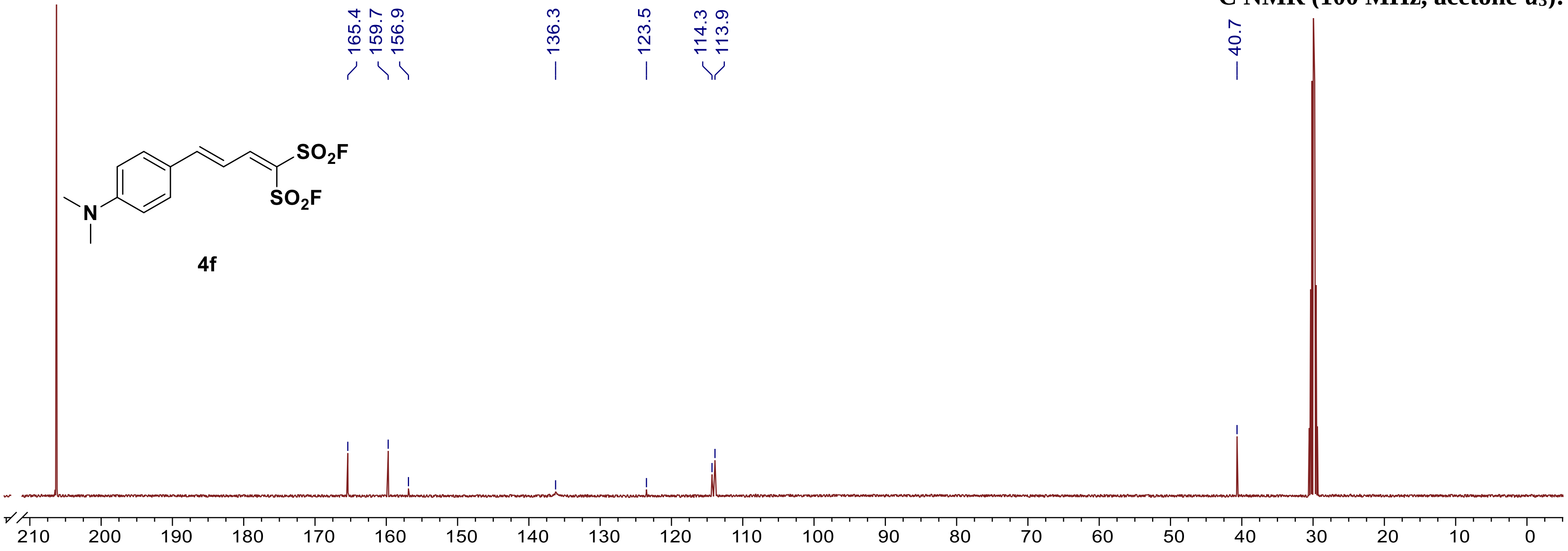

^{19}F NMR (376 MHz, acetone-d_3):

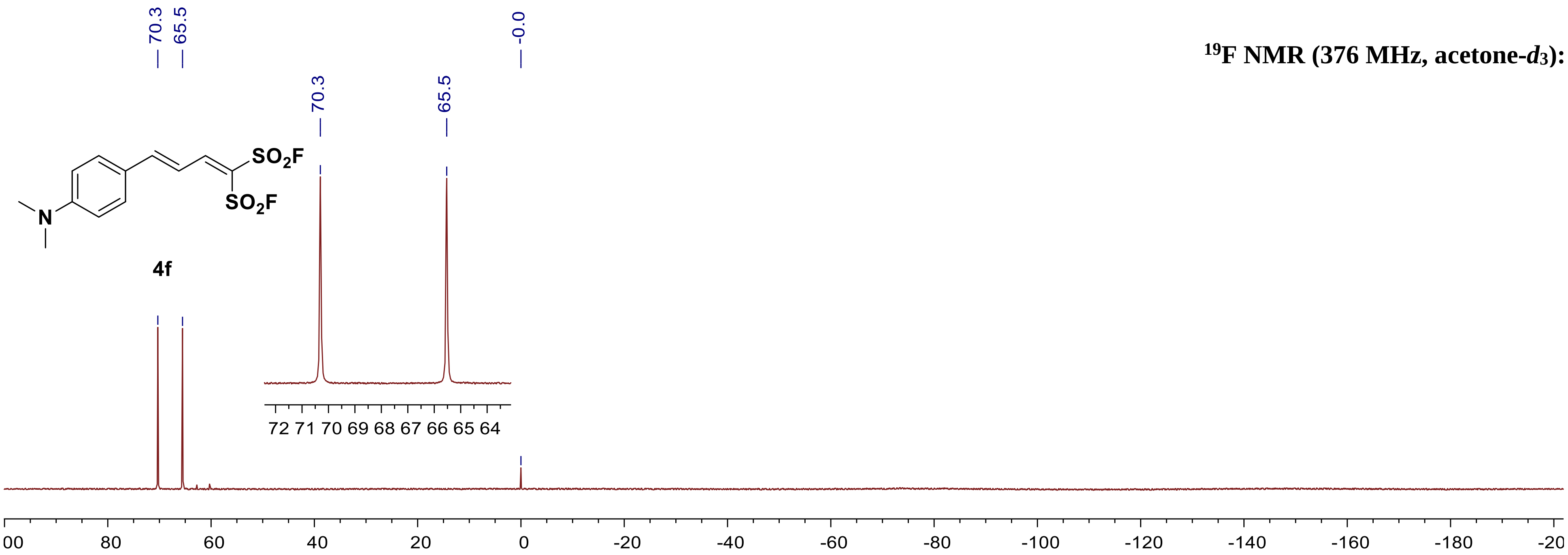

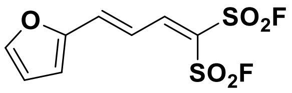

4g

^{1}H NMR (400 MHz, CDCl3):

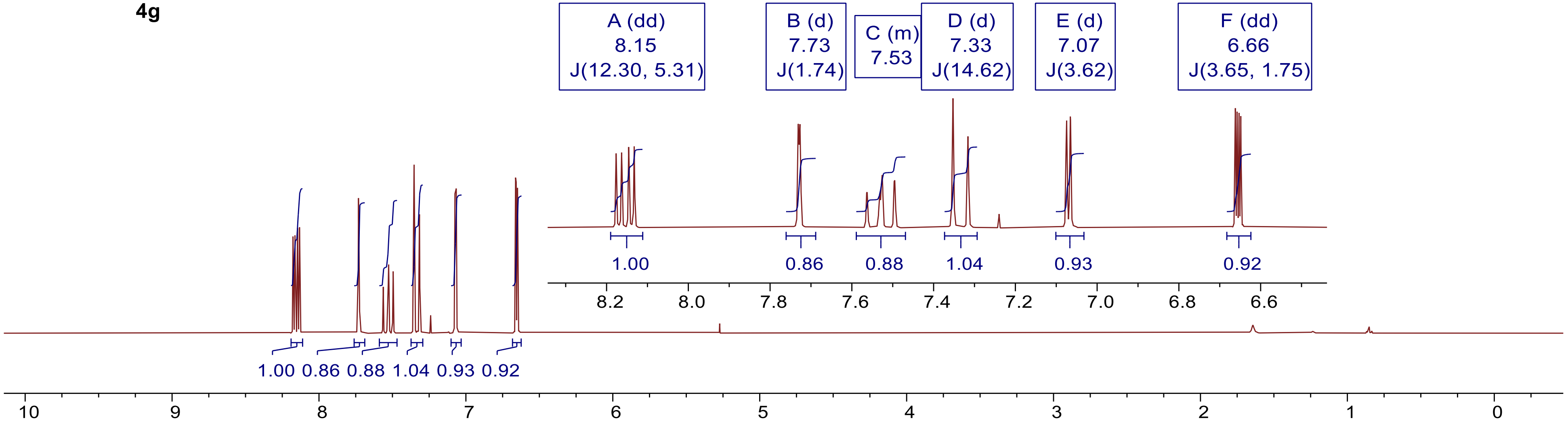

^{13}C NMR (100 MHz, CDCl3):

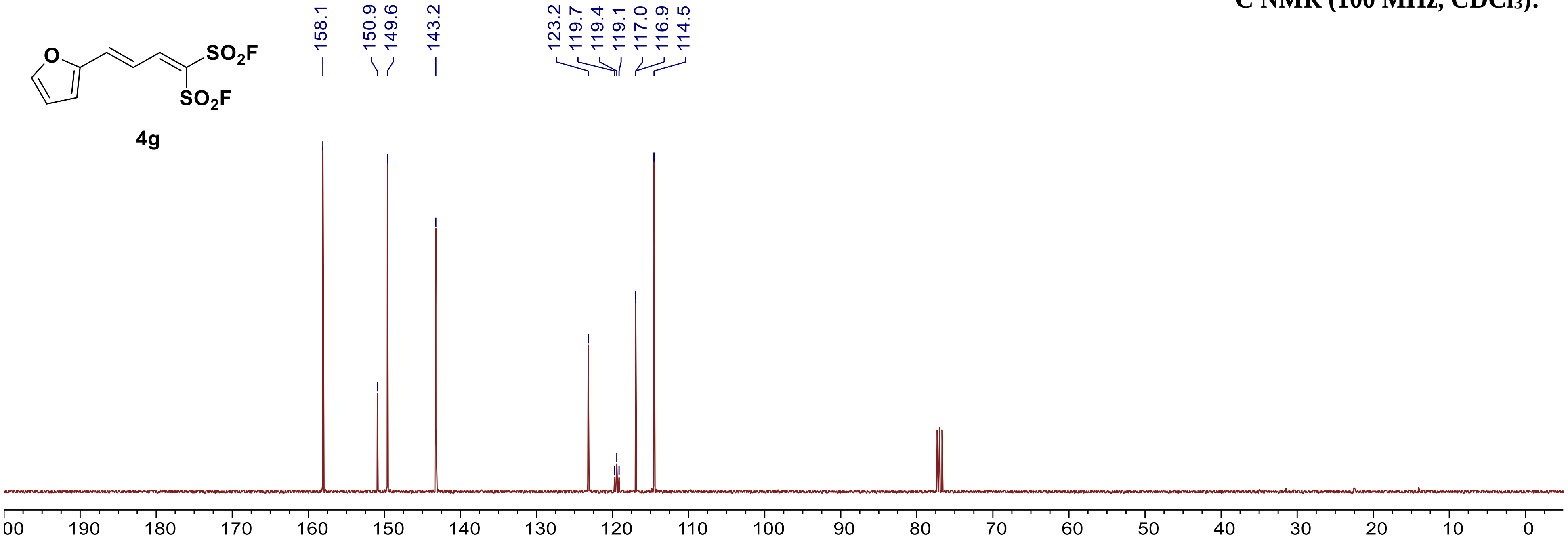

^{19}**F NMR (376 MHz, CDCl$_3$):**

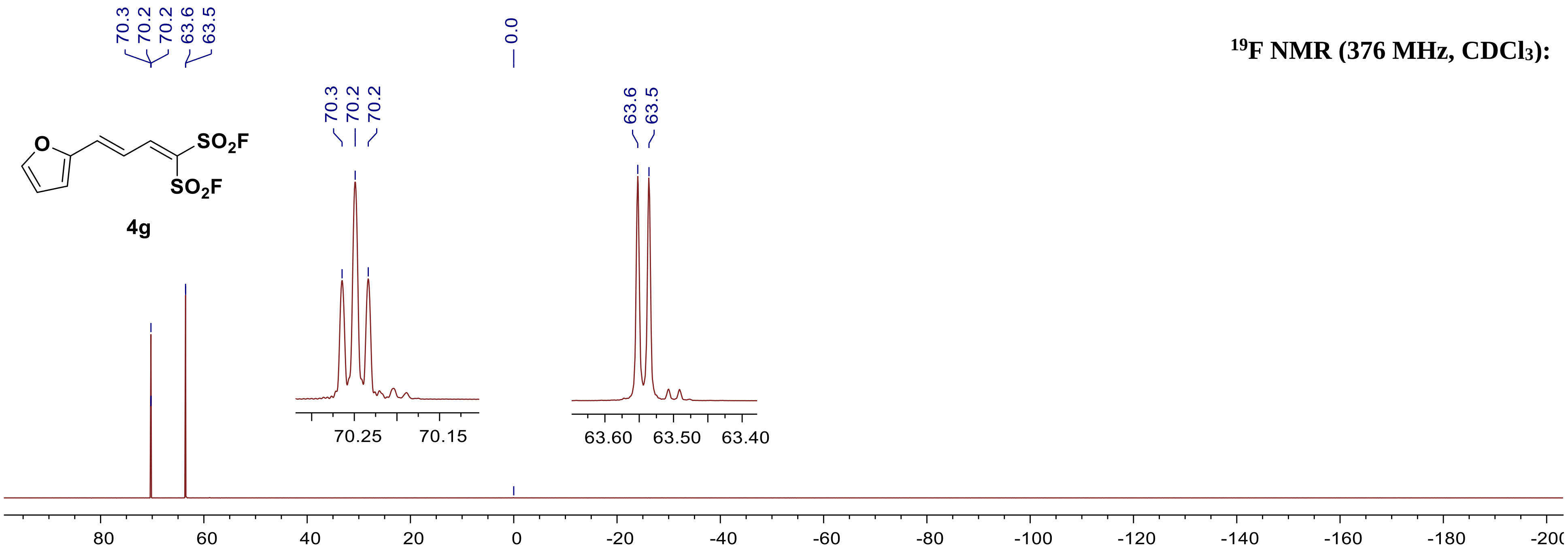

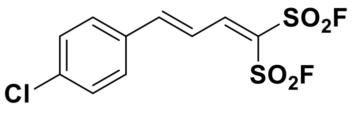

4h

^{1}H NMR (400 MHz, CDCl3):

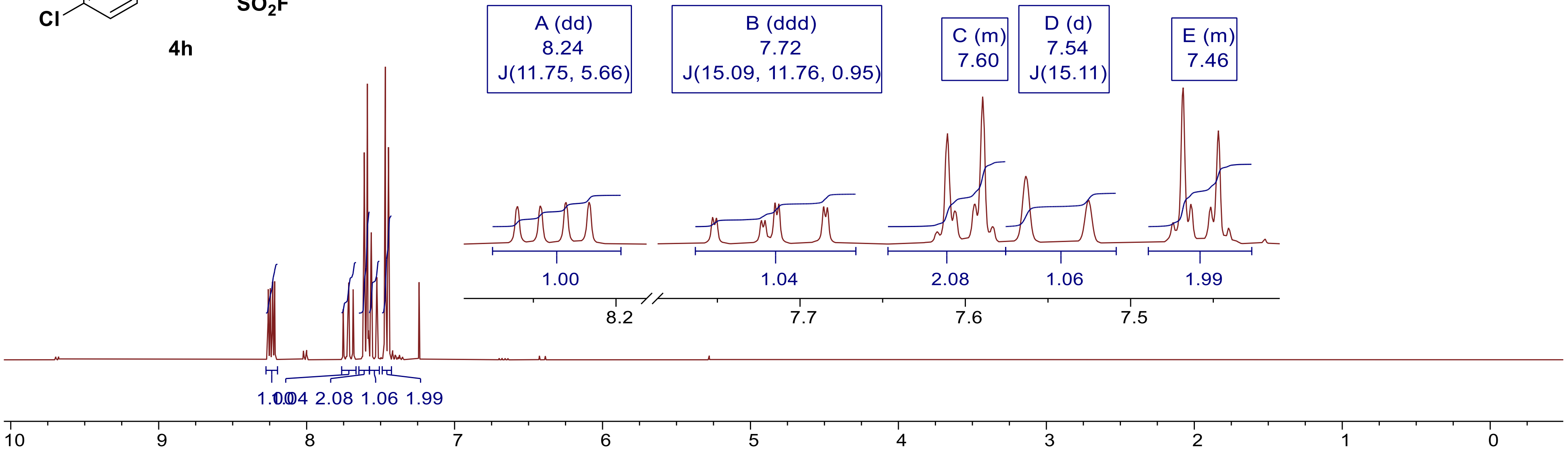

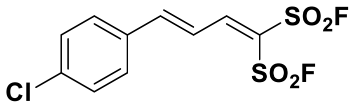

4h

^{13}C NMR (100 MHz, CDCl3):

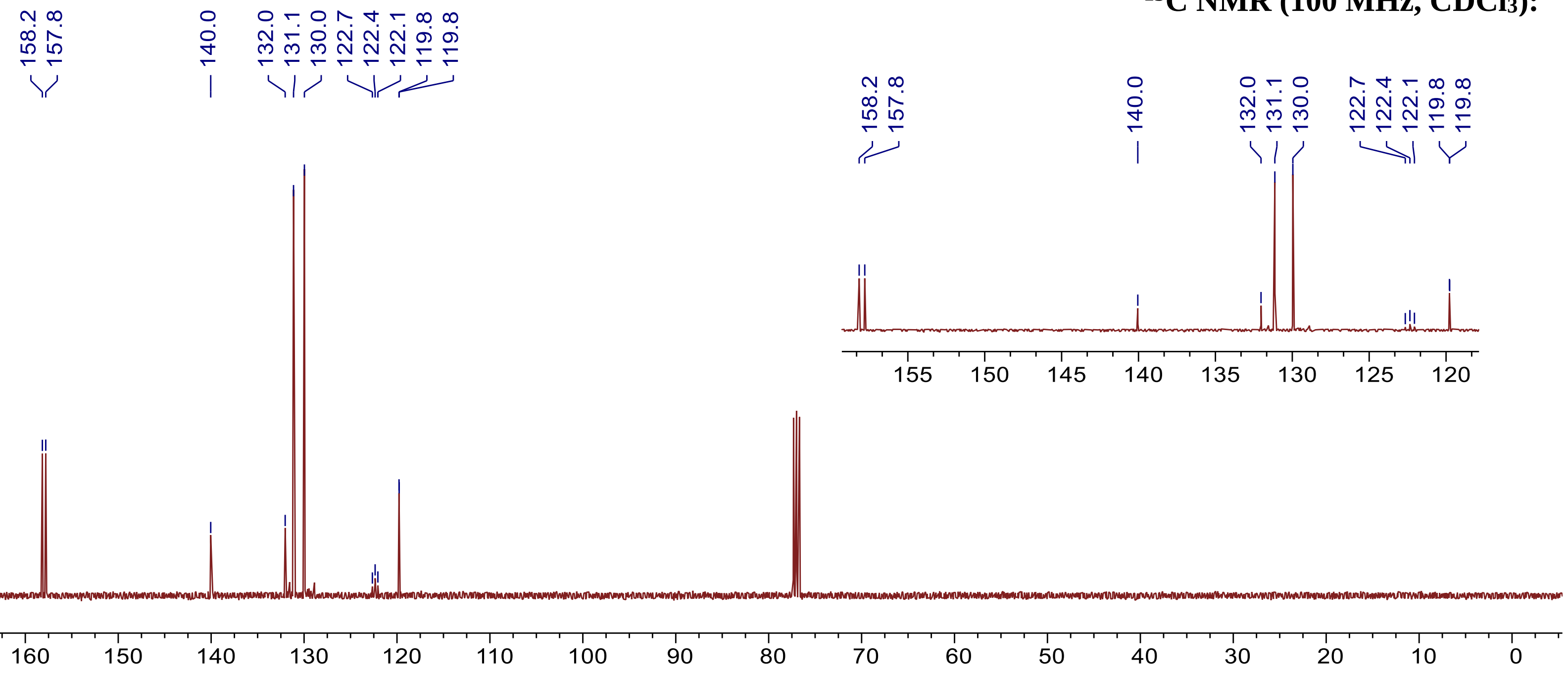

^{19}F NMR (376 MHz, $CDCl_3$):

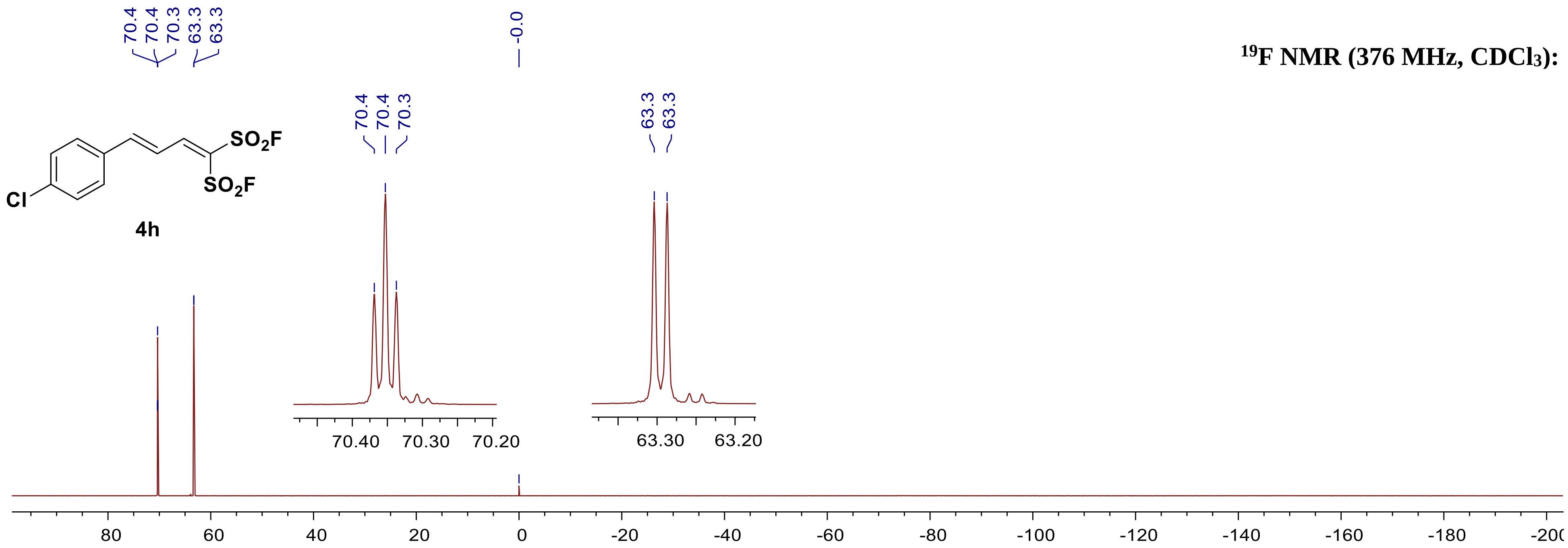

^{1}H NMR (400 MHz, CDCl₃):

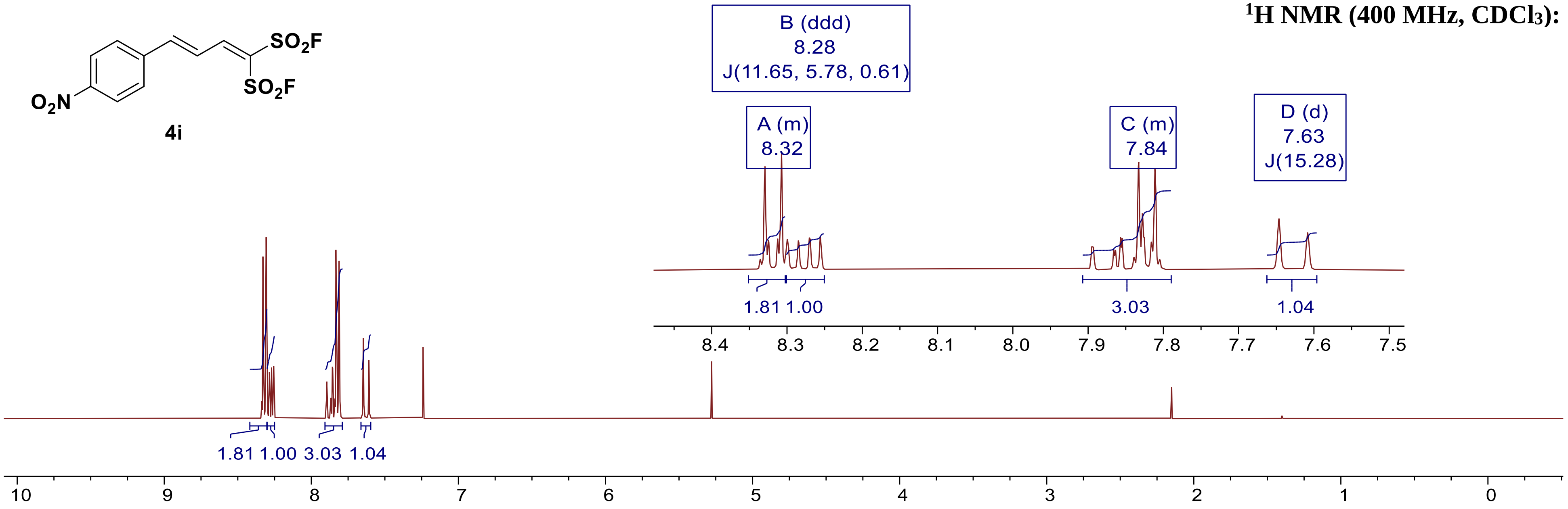

^{13}C NMR (100 MHz, CDCl₃):

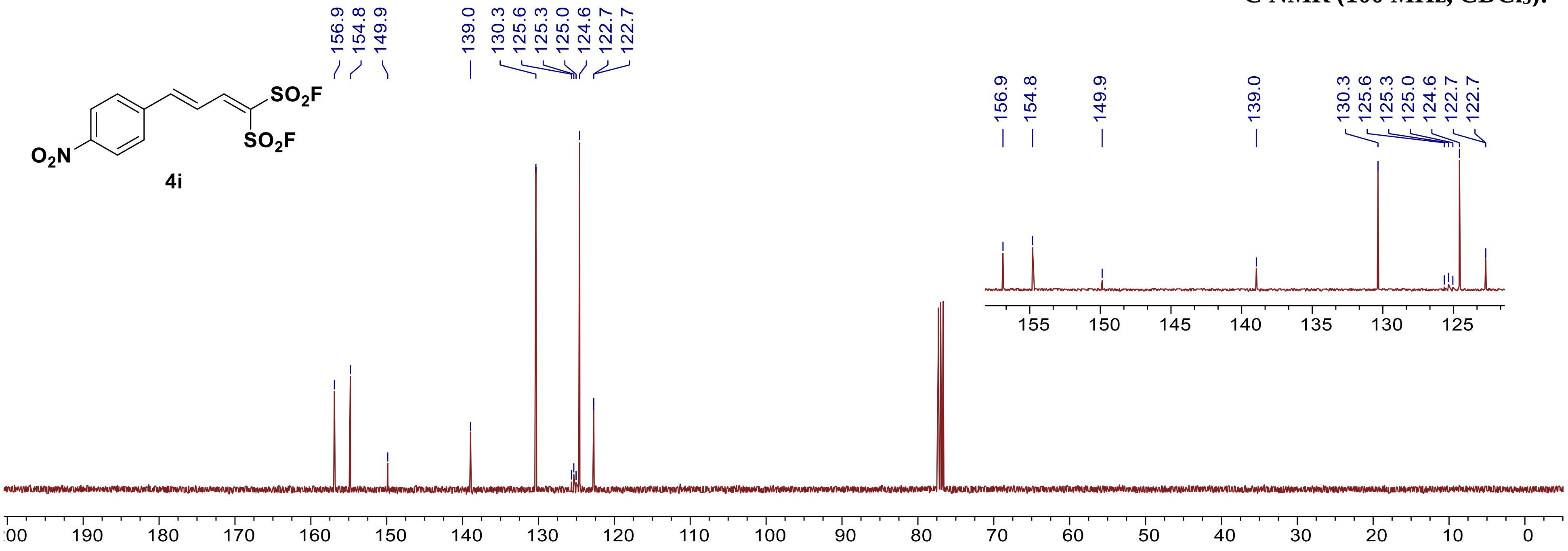

^{19}F NMR (376 MHz, CDCl$_3$):

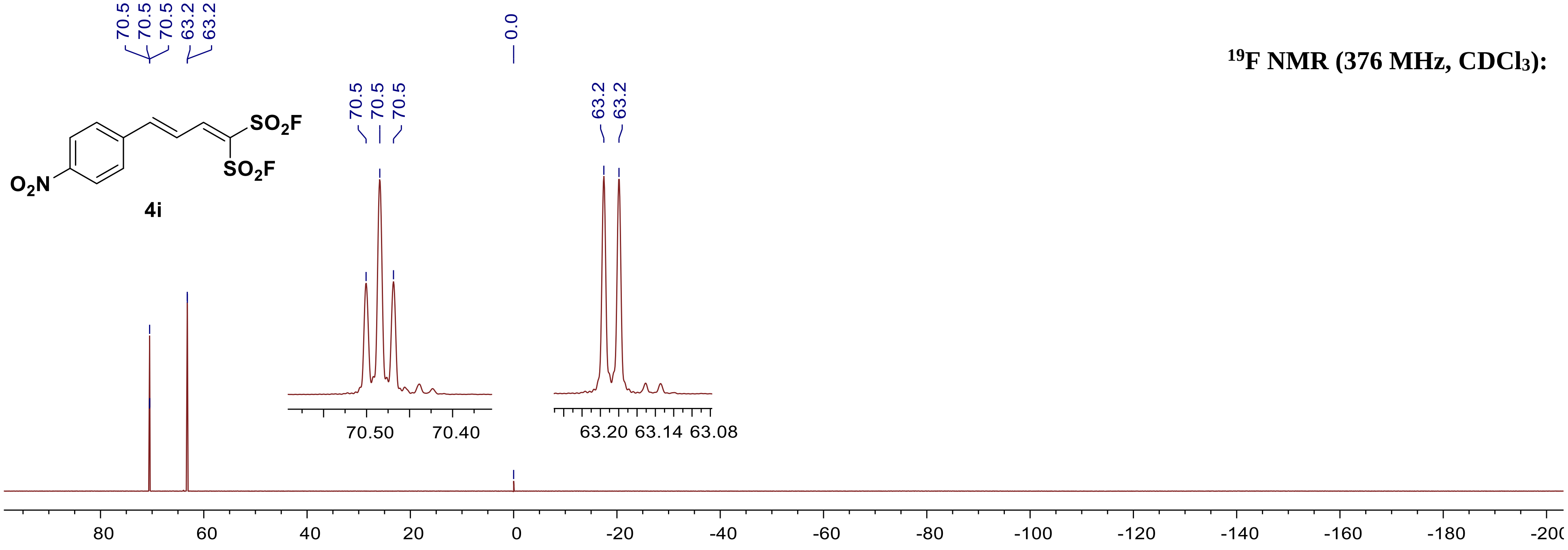

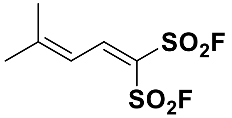

4j

^{1}H NMR (400 MHz, CDCl$_3$):

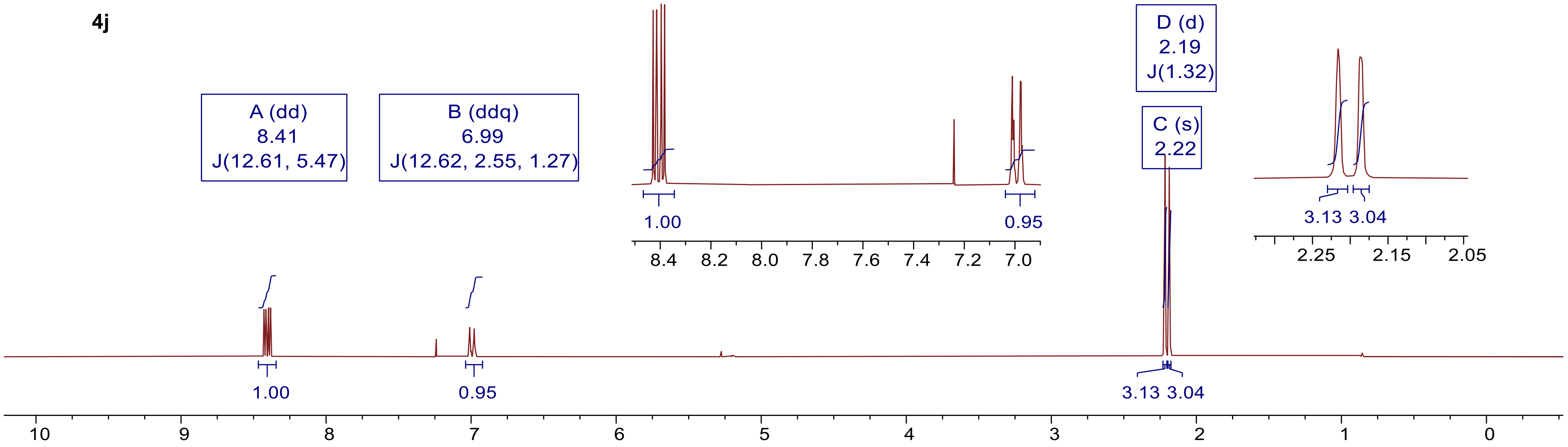

^{13}C NMR (100 MHz, CDCl$_3$):

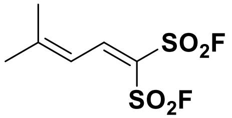

4j

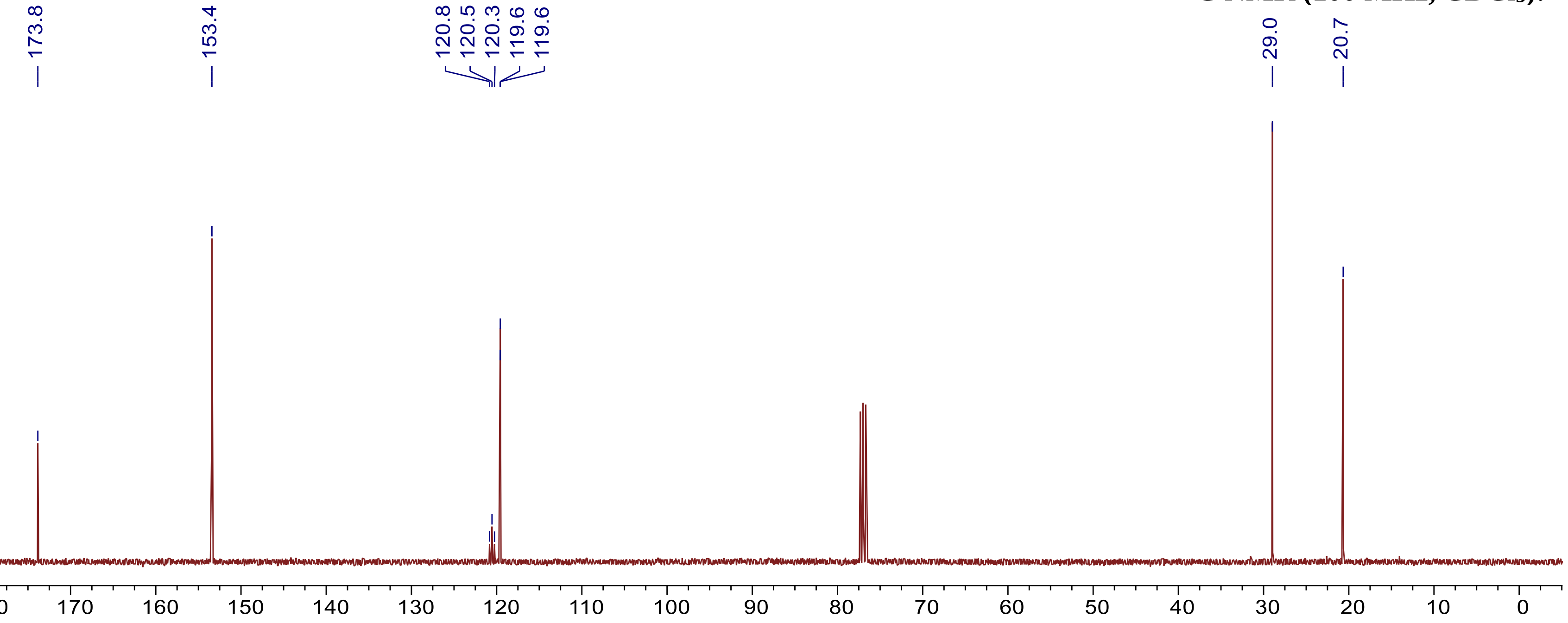

^{19}F NMR (376 MHz, CDCl$_3$):

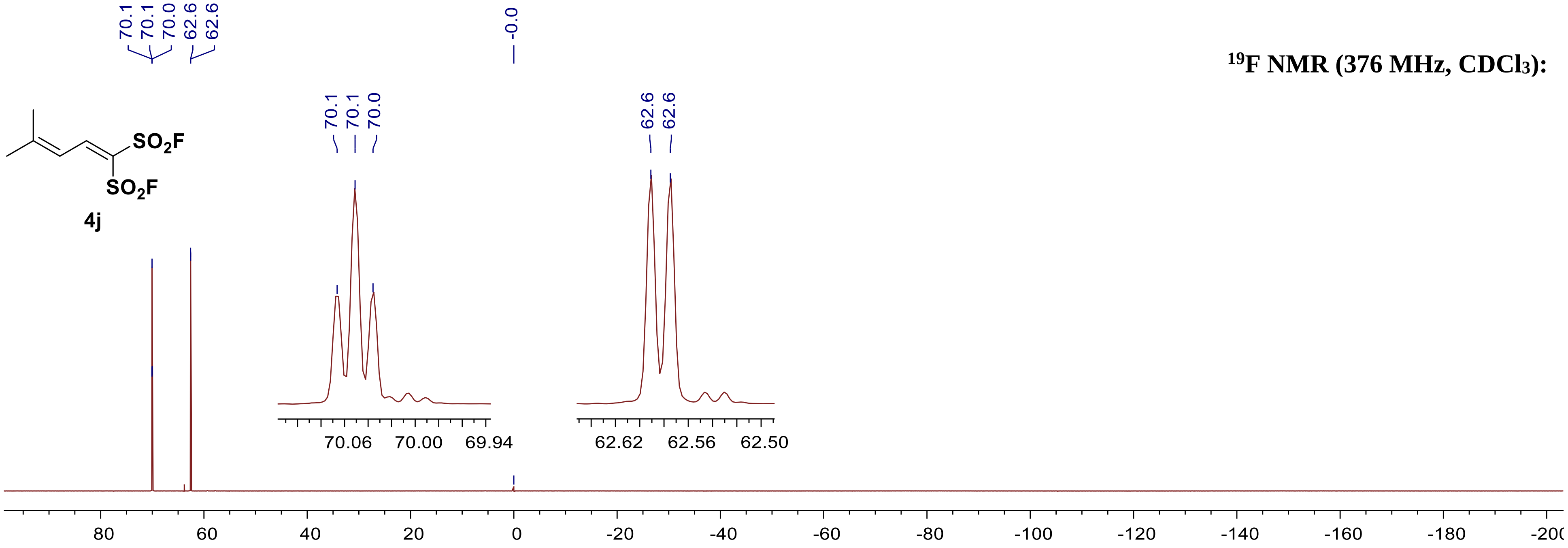

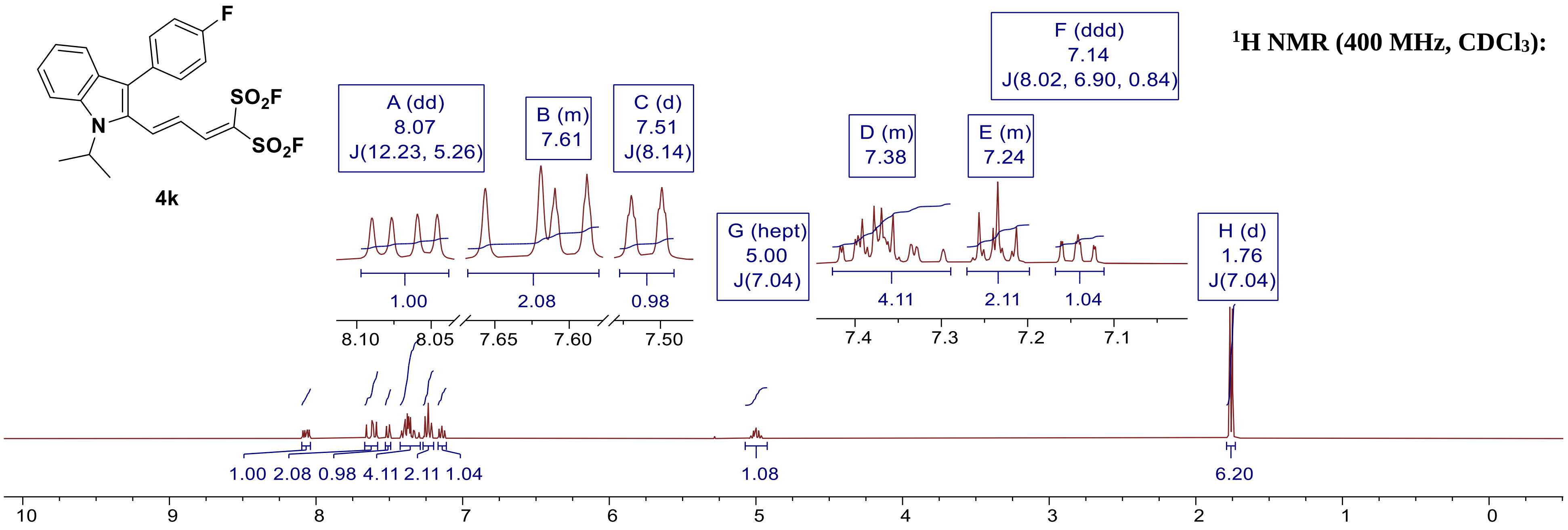

F
SO_2F
N
SO_2F
4k
^{1}H NMR (400 MHz, $CDCl_3$):
A (dd) 8.07 J(12.23, 5.26)
B (m) 7.61
C (d) 7.51 J(8.14)
D (m) 7.38
E (m) 7.24
F (ddd) 7.14 J(8.02, 6.90, 0.84)
G (hept) 5.00 J(7.04)
H (d) 1.76 J(7.04)
1.00
2.08
0.98
4.11
2.11
1.04
8.10
8.05
7.65
7.60
7.50
7.4
7.3
7.2
7.1
1.00 2.08 0.98 4.11 2.11 1.04
1.08
6.20
10
9
8
7
6
5
4
3
2
1
0

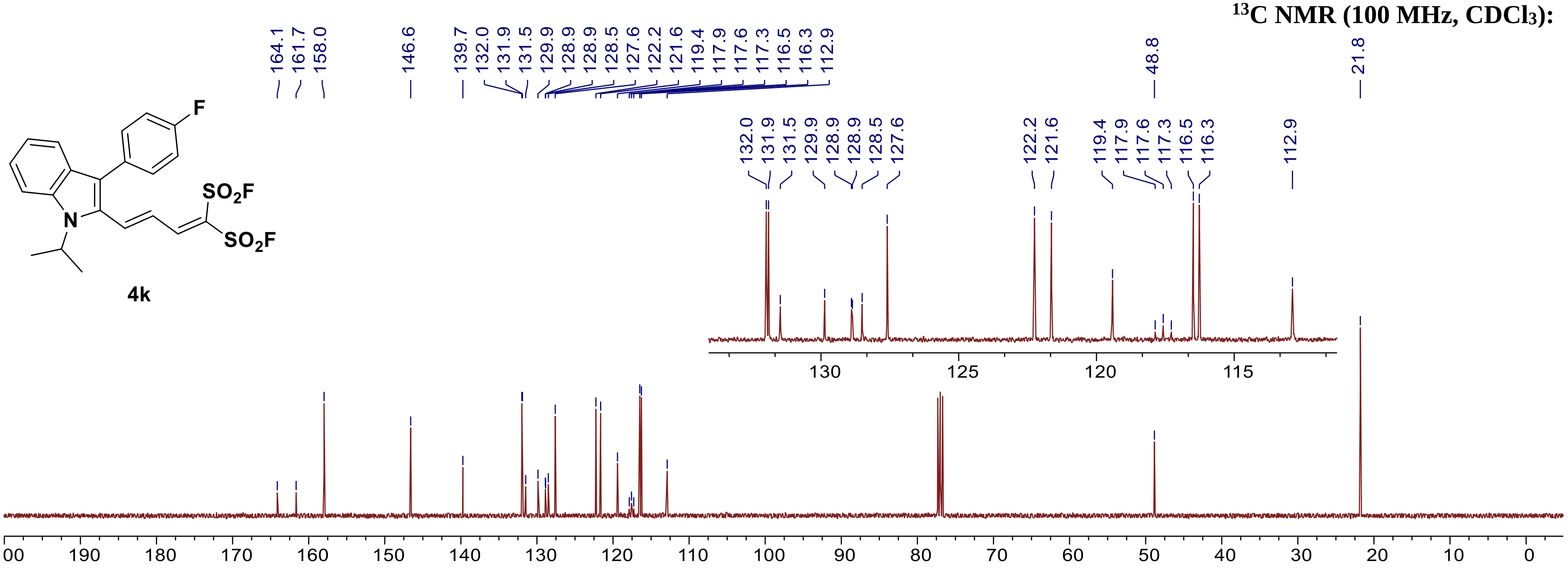

^{13}C NMR (100 MHz, $CDCl_3$):
164.1
161.7
158.0
146.6
139.7
132.0
131.9
131.5
129.9
128.9
128.9
128.5
127.6
122.2
121.6
119.4
117.9
117.6
117.3
116.5
116.3
112.9
48.8
21.8
F
SO_2F
N
SO_2F
4k
130
125
120
115
00
190
180
170
160
150
140
130
120
110
100
90
80
70
60
50
40
30
20
10
0

^{19}F NMR (376 MHz, $CDCl_3$):

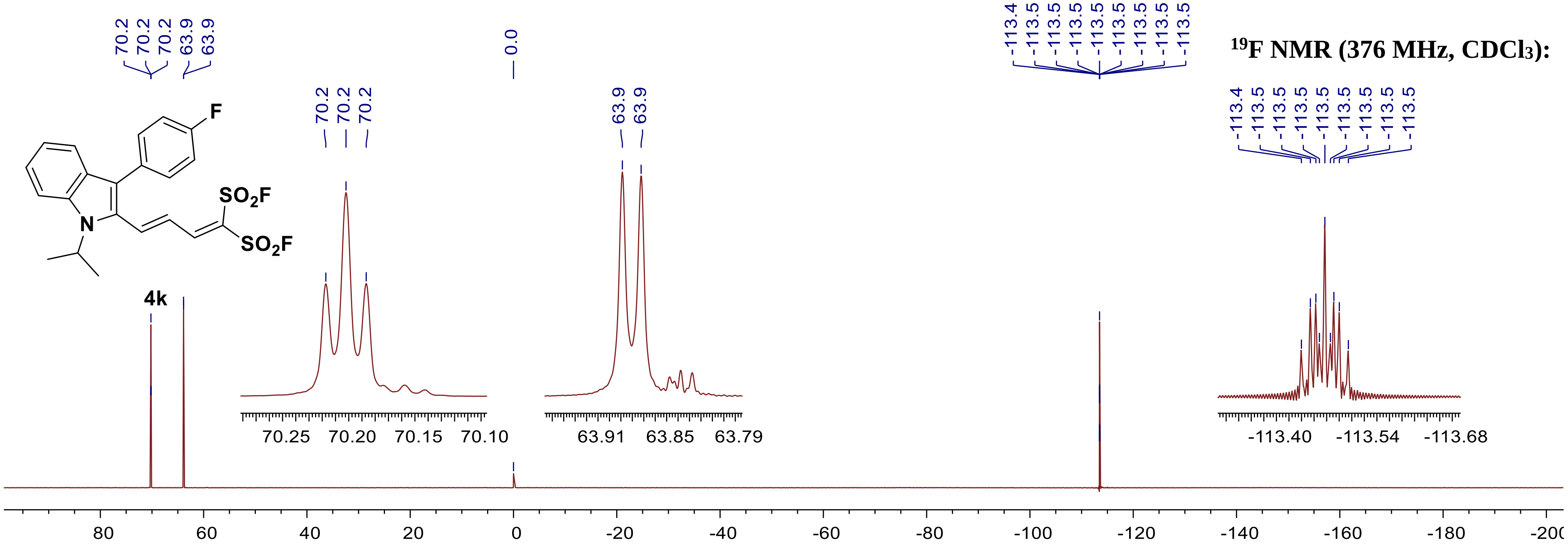

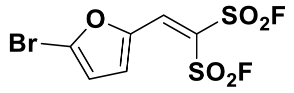

4l

^{1}H NMR (400 MHz, CDCl$_3$):

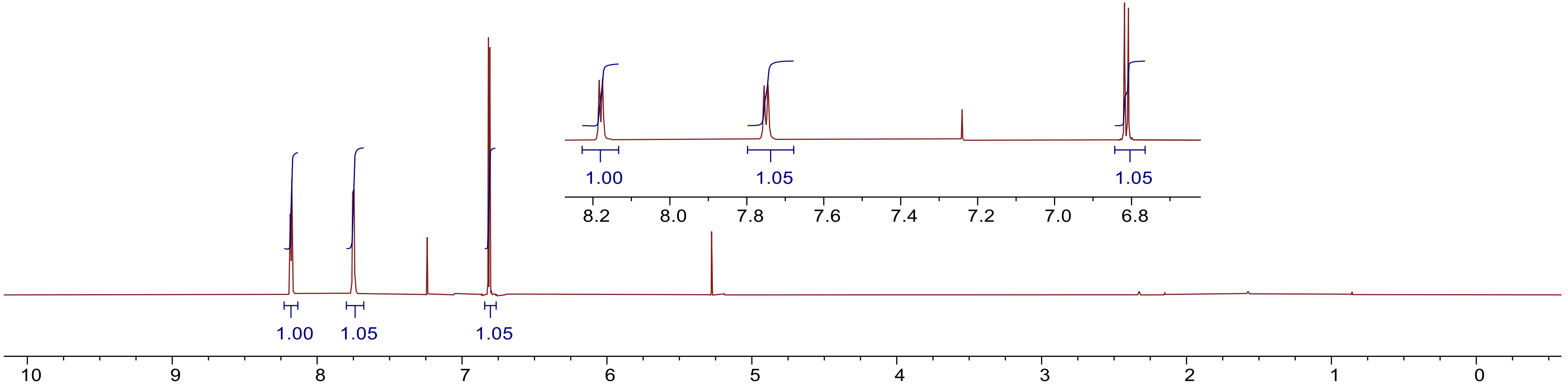

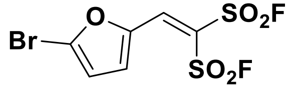

4l

^{13}C NMR (100 MHz, CDCl$_3$):

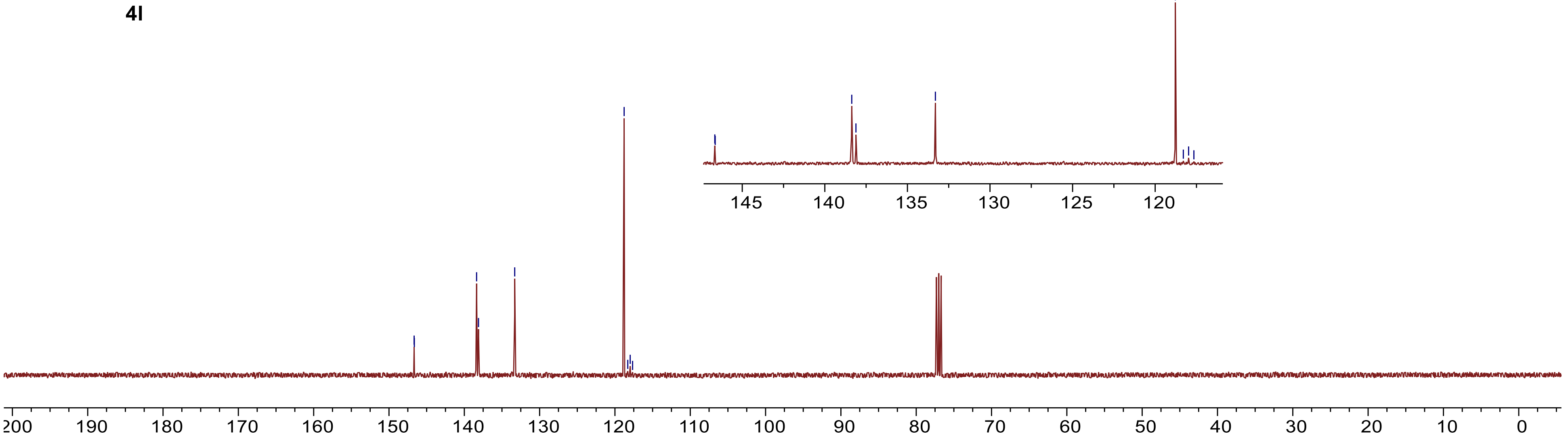

^{19}F NMR (376 MHz, $CDCl_3$):

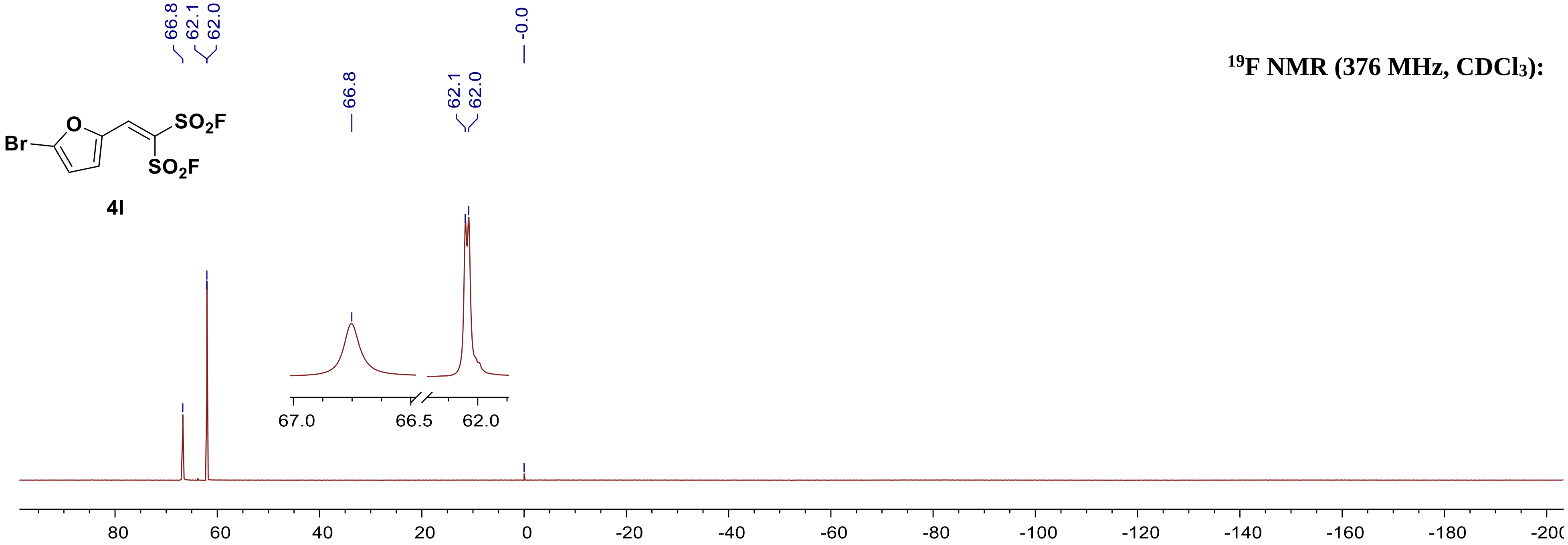

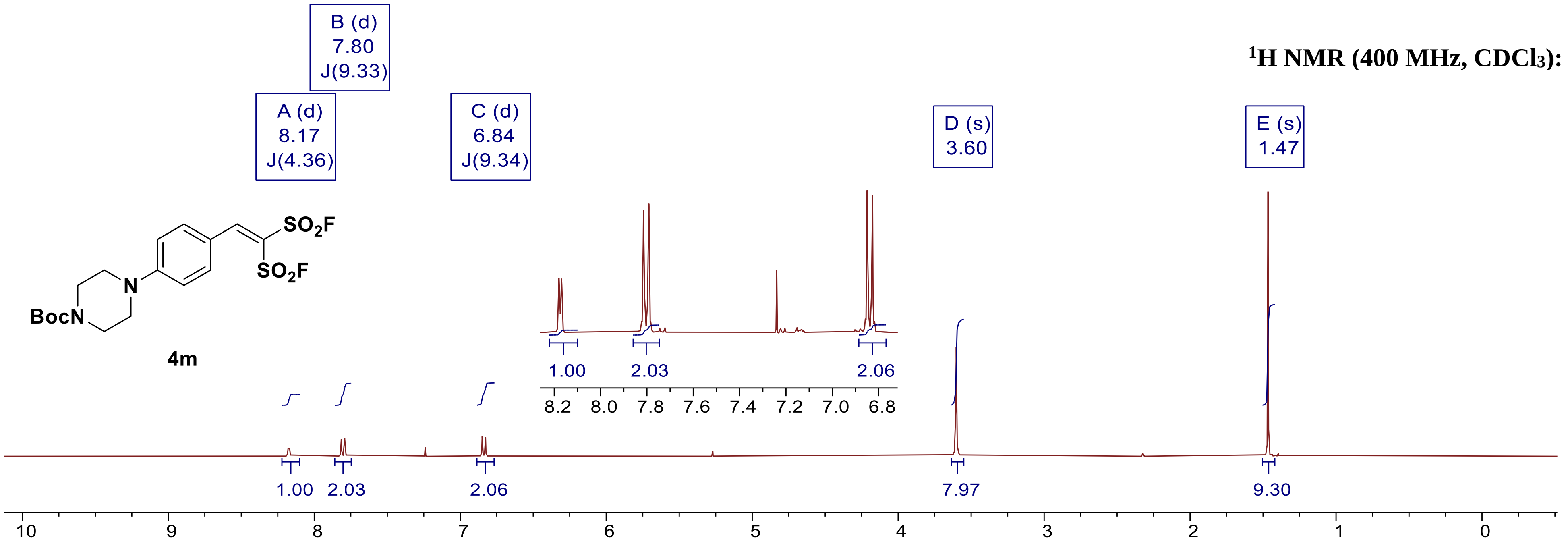

¹H NMR (400 MHz, CDCl₃):
A (d)
8.17
J(4.36)
B (d)
7.80
J(9.33)
C (d)
6.84
J(9.34)
D (s)
3.60
E (s)
1.47
SO2F
SO2F
N
BocN
4m
1.00
2.03
2.06
7.97
9.30

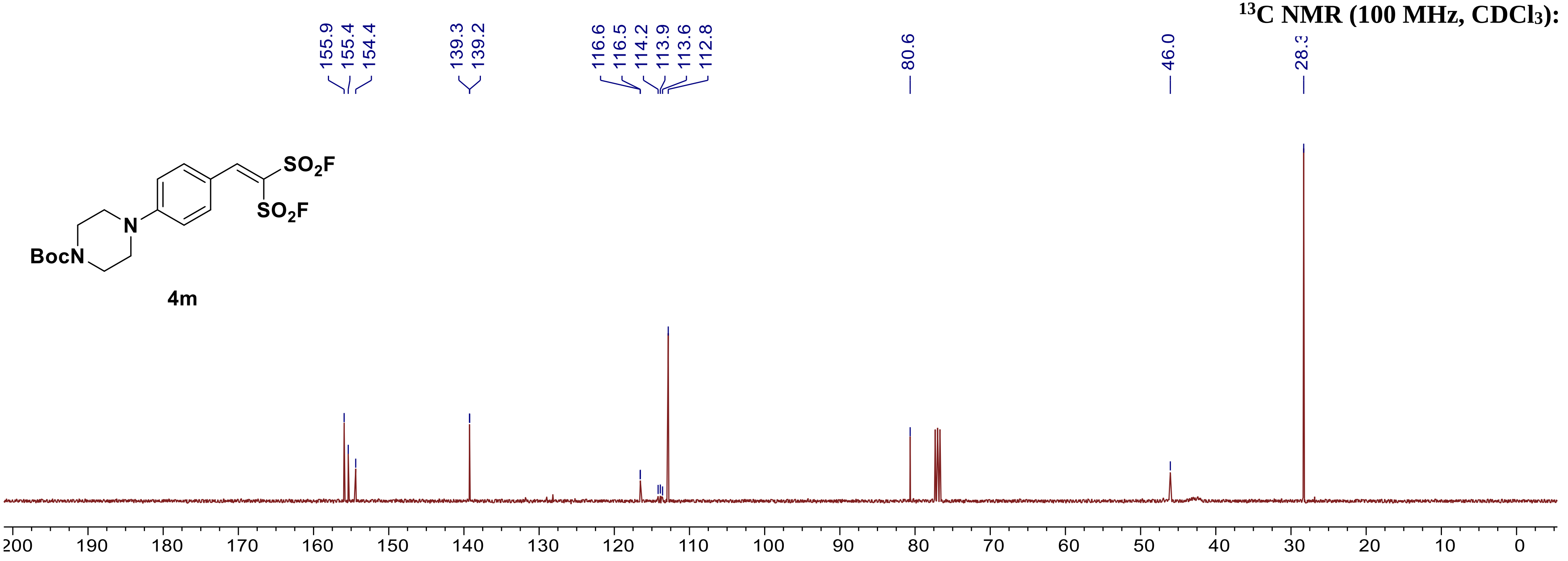

¹³C NMR (100 MHz, CDCl₃):
155.9
155.4
154.4
139.3
139.2
116.6
116.5
114.2
113.9
113.6
112.8
80.6
46.0
SO2F
SO2F
N
BocN
4m

^{19}F NMR (376 MHz, $CDCl_3$):

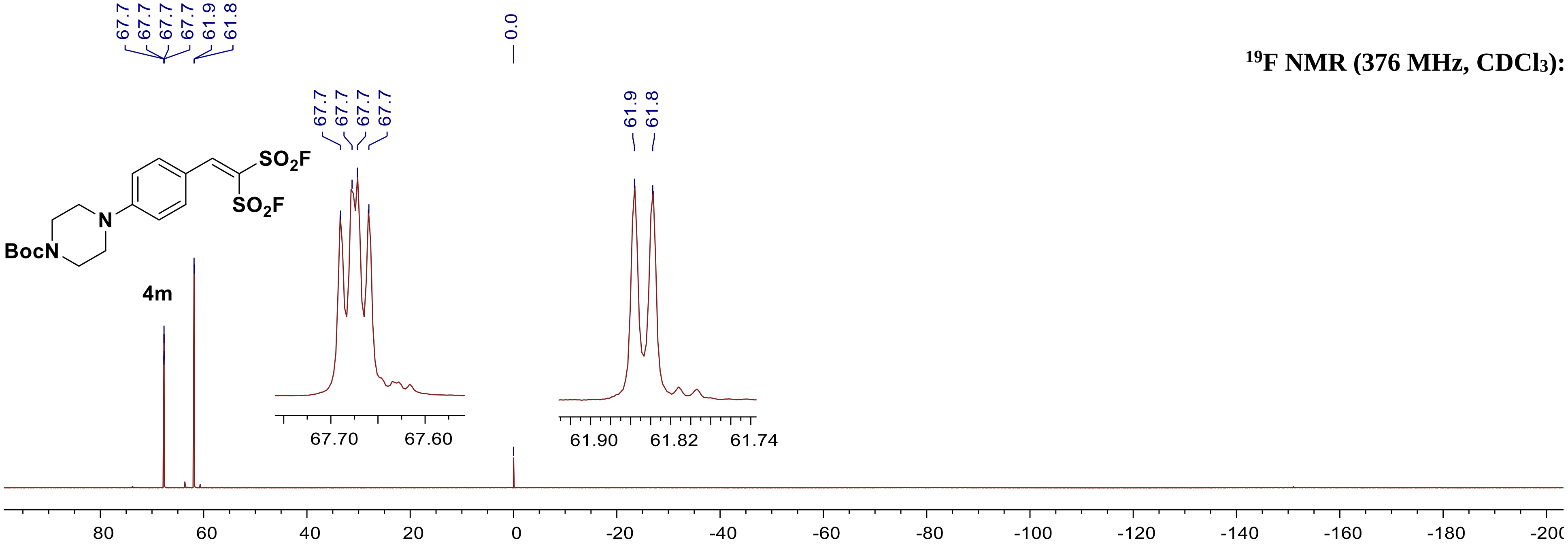

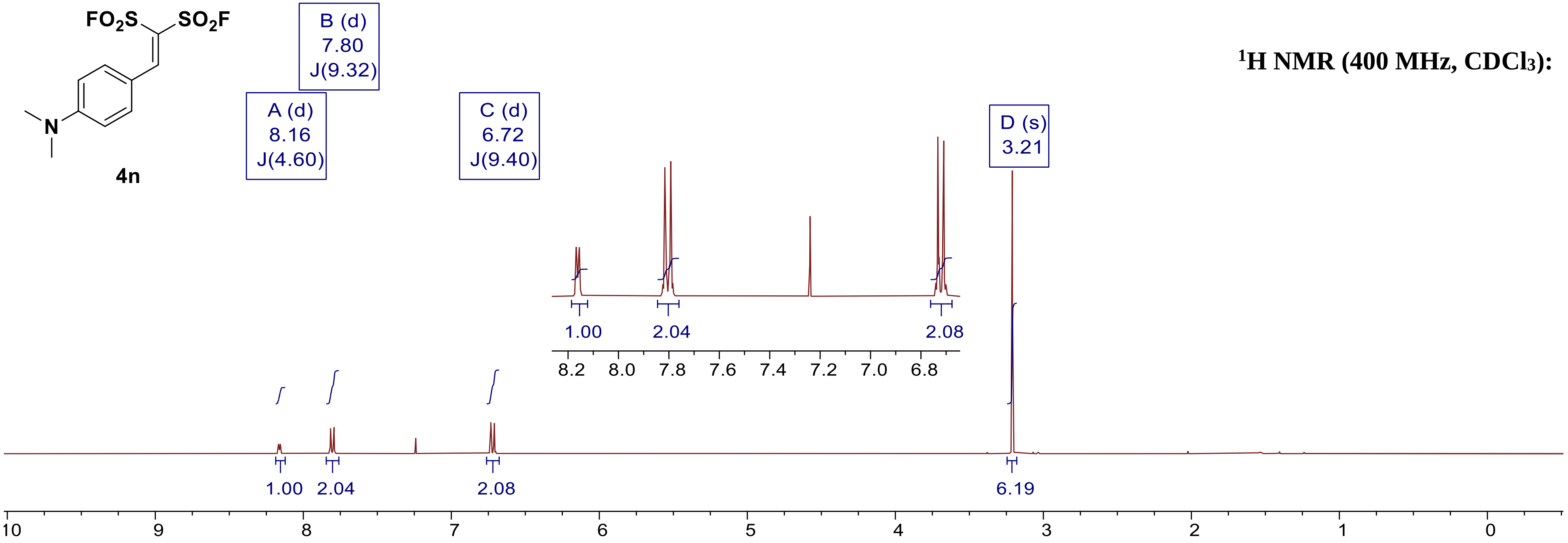

FO2S
SO2F
N
4n
B (d)
7.80
J(9.32)
A (d)
8.16
J(4.60)
C (d)
6.72
J(9.40)
D (s)
3.21
¹H NMR (400 MHz, CDCl3):
1.00
2.04
2.08
6.19

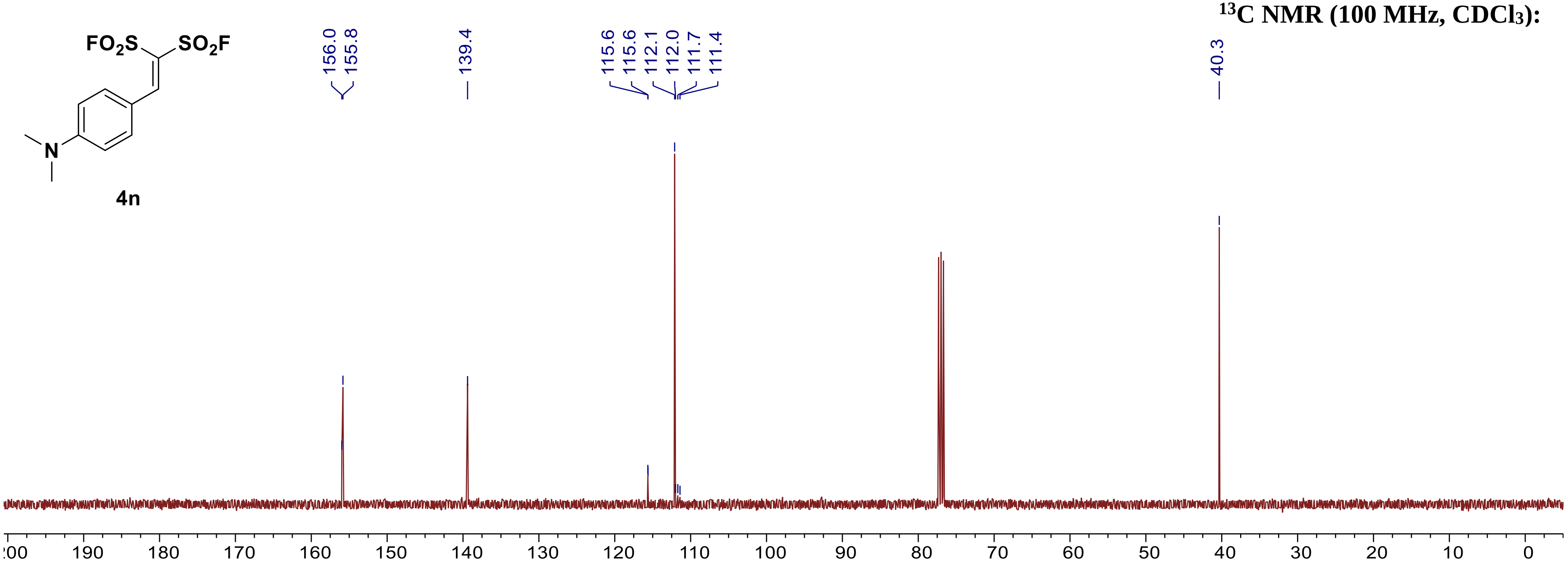

¹³C NMR (100 MHz, CDCl3):
FO2S
SO2F
N
4n
156.0
155.8
139.4
115.6
115.6
112.1
112.0
111.7
111.4
40.3

^{19}F NMR (376 MHz, CDCl3):

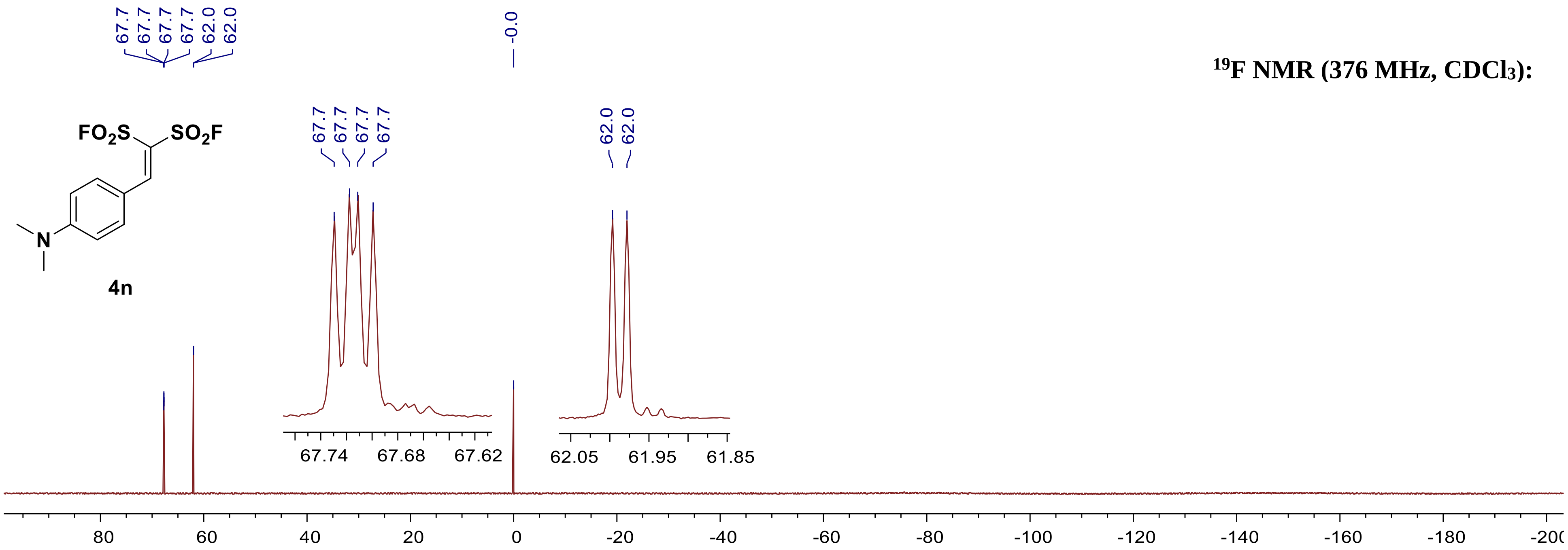

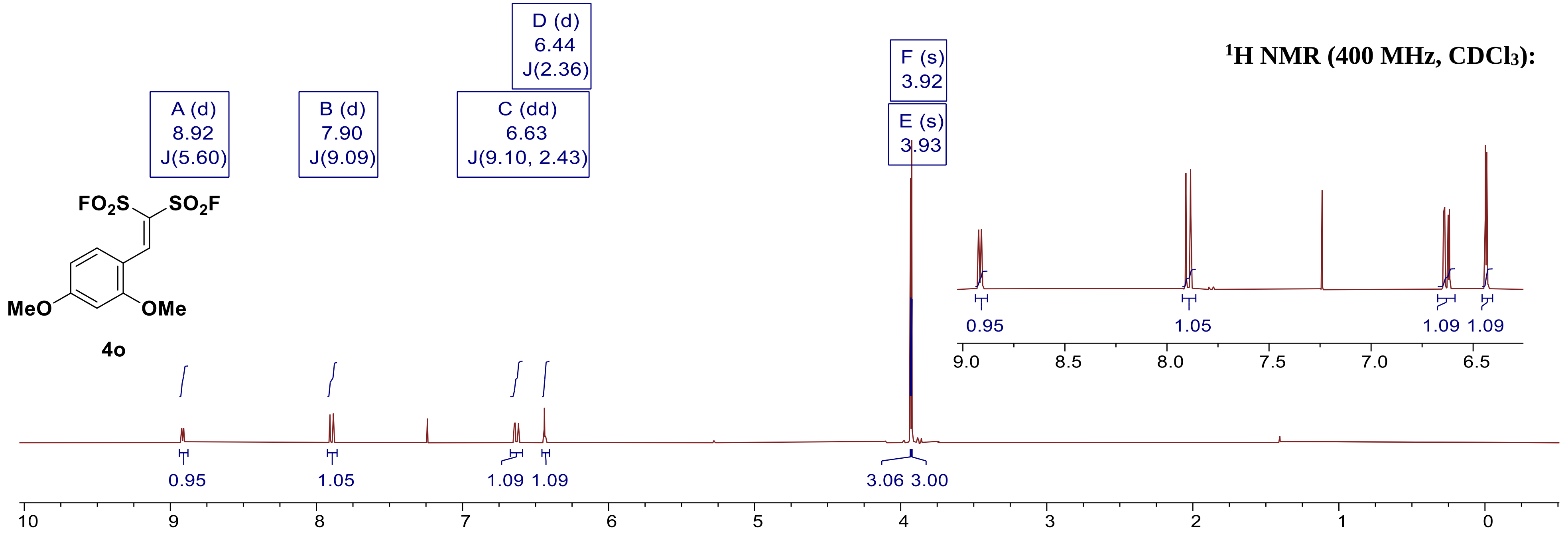

¹H NMR (400 MHz, CDCl3):
A (d)
8.92
J(5.60)
B (d)
7.90
J(9.09)
C (dd)
6.63
J(9.10, 2.43)
D (d)
6.44
J(2.36)
E (s)
3.93
F (s)
3.92
FO2S
SO2F
MeO
OMe
4o
0.95
1.05
1.09 1.09
3.06 3.00

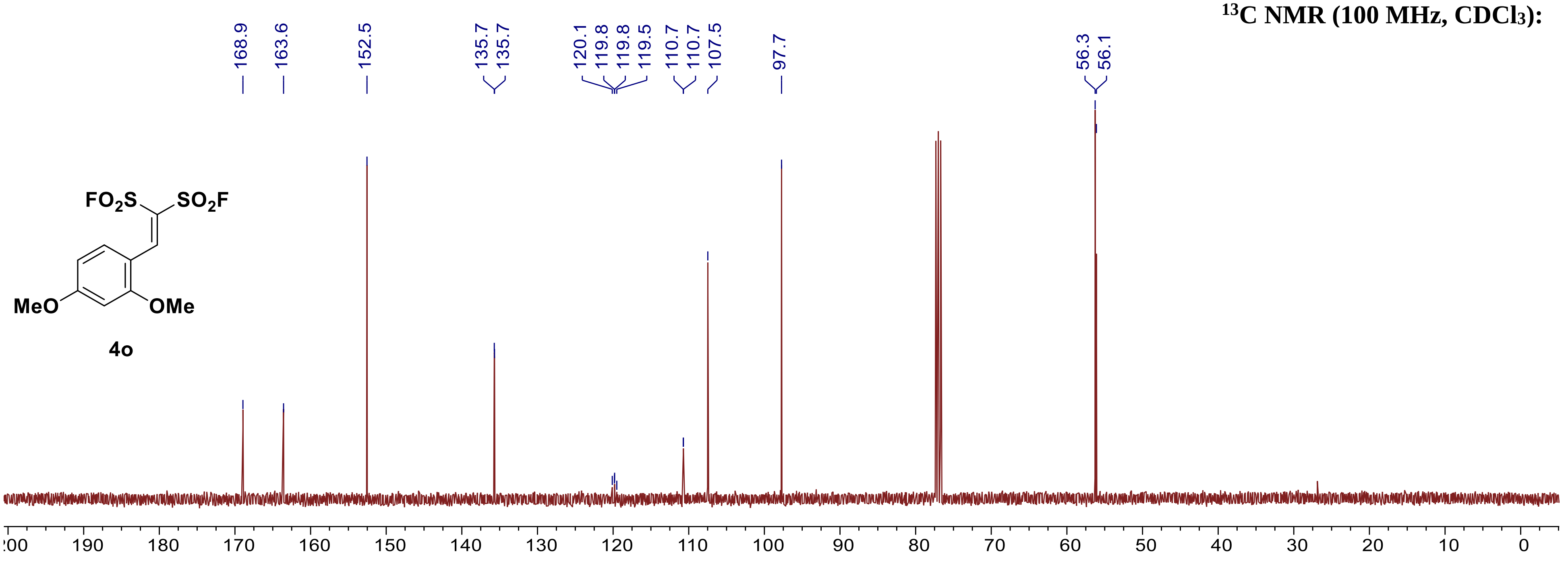

13C NMR (100 MHz, CDCl3):
168.9
163.6
152.5
135.7
135.7
120.1
119.8
119.8
119.5
110.7
110.7
107.5
97.7
56.3
56.1
FO2S
SO2F
MeO
OMe
4o

^{19}F NMR (376 MHz, $CDCl_3$):

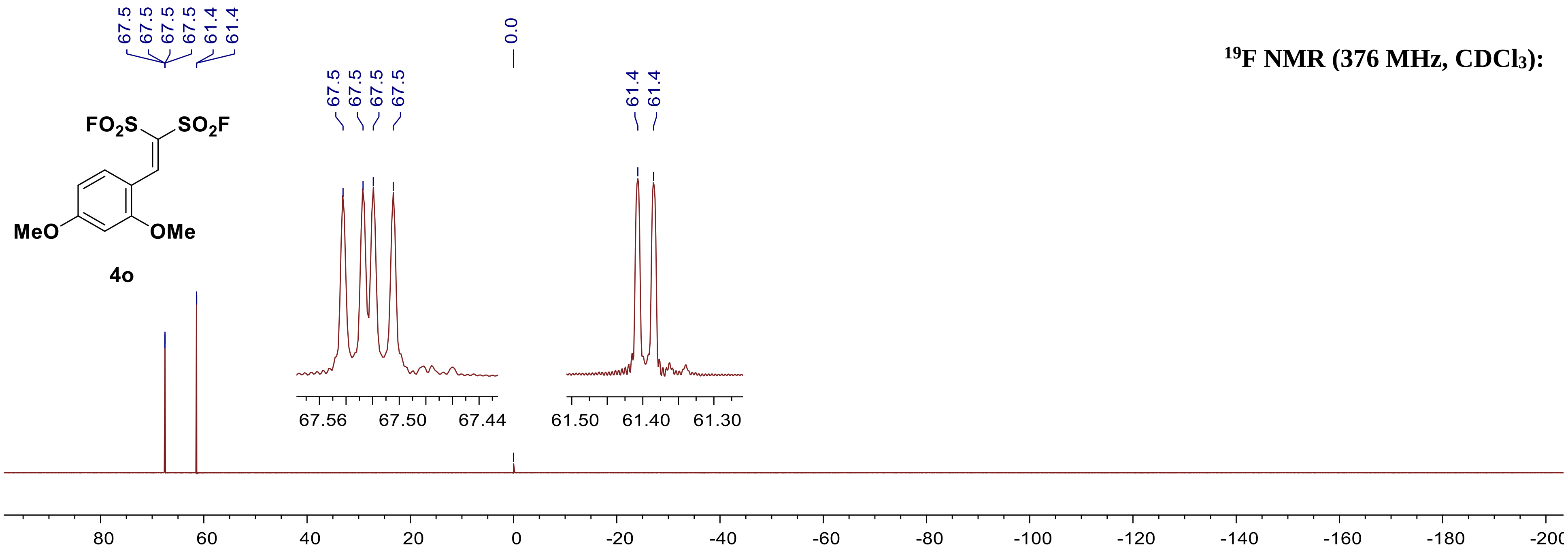

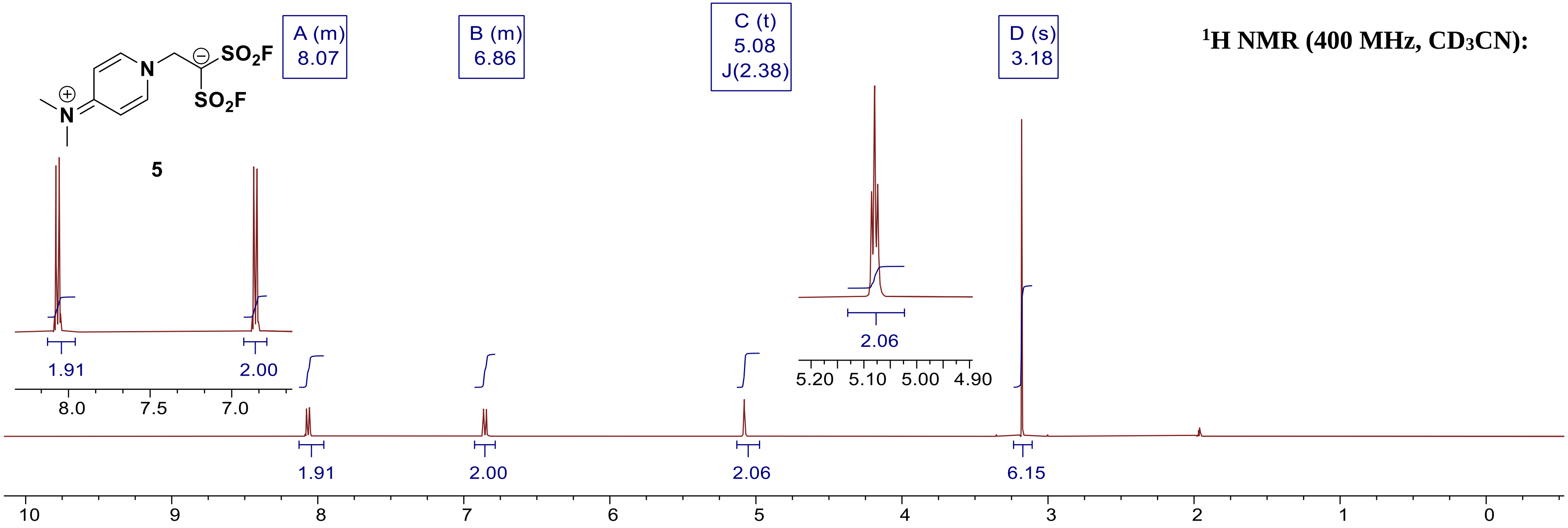

^{1}H NMR (400 MHz, CD$_3$CN):
5
A (m) 8.07
B (m) 6.86
C (t) 5.08 J(2.38)
D (s) 3.18
1.91
2.00
2.06
6.15

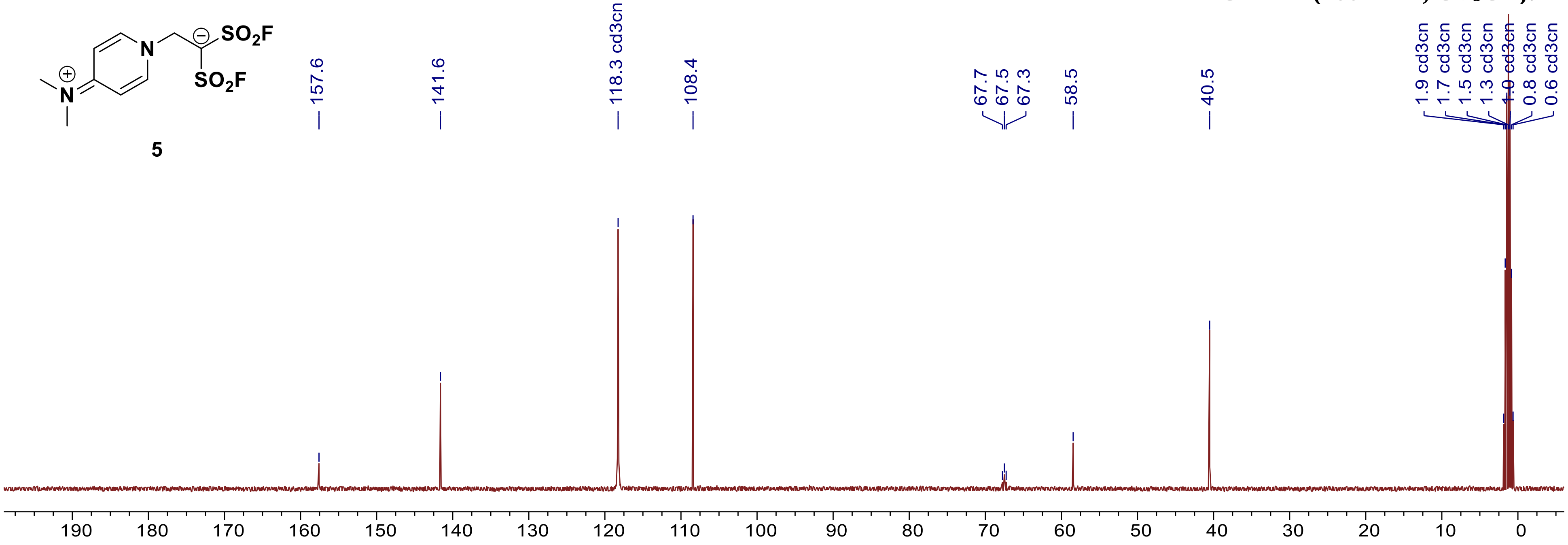

^{13}C NMR (100 MHz, CD$_3$CN):
5
157.6
141.6
118.3 cd3cn
108.4
67.7
67.5
67.3
58.5
40.5
1.9 cd3cn
1.7 cd3cn
1.5 cd3cn
1.3 cd3cn
1.0 cd3cn
0.8 cd3cn
0.6 cd3cn

^{19}F NMR (376 MHz, CD_3CN):

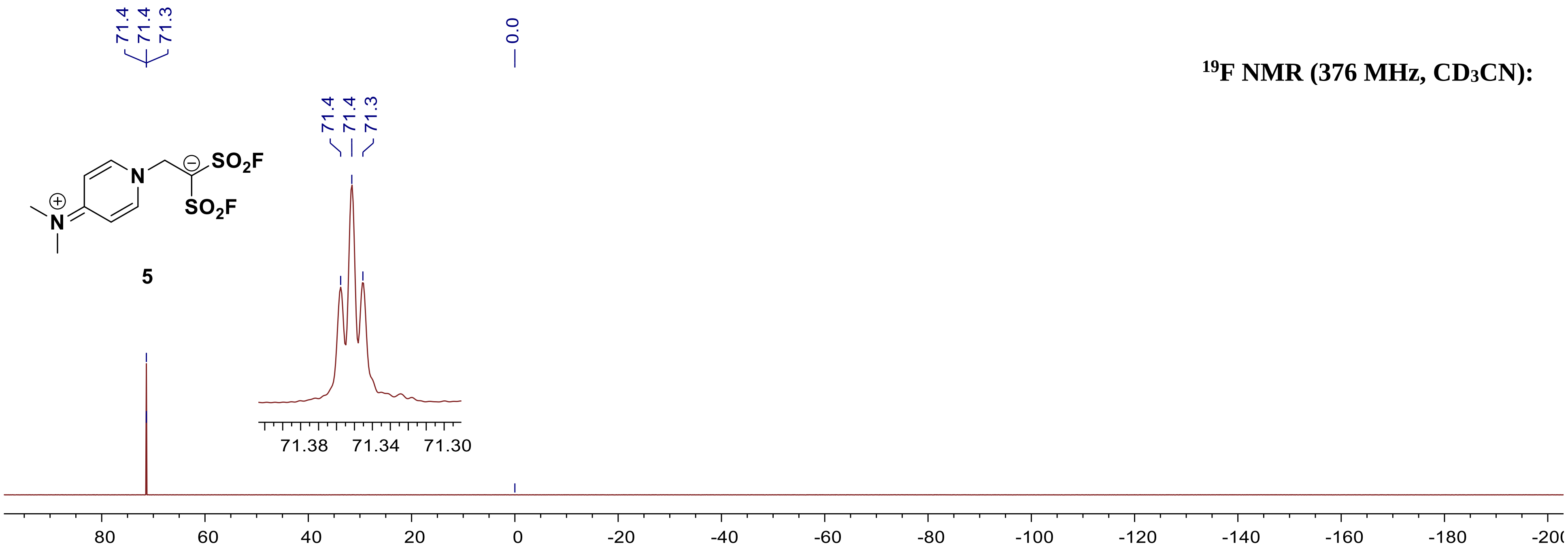

5
